# 2- and 3-substituted imidazo[1,2-*a*]pyrazines as inhibitors of bacterial type IV secretion

**DOI:** 10.1016/j.bmc.2014.09.036

**Published:** 2014-11-15

**Authors:** James R. Sayer, Karin Walldén, Thomas Pesnot, Frederick Campbell, Paul J. Gane, Michela Simone, Hans Koss, Floris Buelens, Timothy P. Boyle, David L. Selwood, Gabriel Waksman, Alethea B. Tabor

**Affiliations:** a Department of Chemistry, UCL, 20, Gordon Street, London WC1H 0AJ, UK; b Institute of Structural and Molecular Biology, UCL and Birkbeck, Malet Street, London WC1E 7HX, UK; c Wolfson Institute for Biomedical Research, UCL, The Cruciform Building, Gower Street, London WC1E 6BT, UK

**Keywords:** 8-Amino imidazo[1,2-*a*]pyrazine, Bacterial type IV secretion system, HP0525, ATPase inhibitor

## Abstract

A novel series of 8-amino imidazo[1,2-*a*]pyrazine derivatives has been developed as inhibitors of the VirB11 ATPase HP0525, a key component of the bacterial type IV secretion system. A flexible synthetic route to both 2- and 3-aryl substituted regioisomers has been developed. The resulting series of imidazo[1,2-*a*]pyrazines has been used to probe the structure–activity relationships of these inhibitors, which show potential as antibacterial agents.

## Graphical abstract

**Figure:**
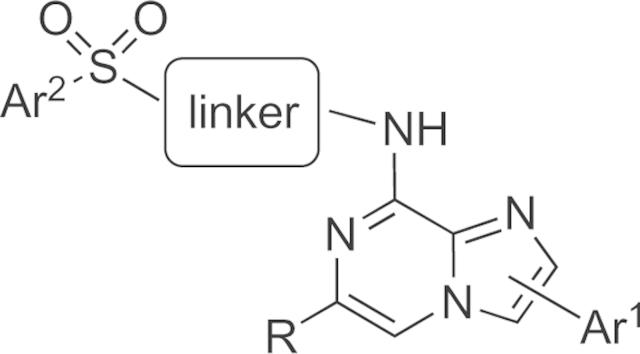

## 1 Introduction

Microorganisms have evolved a number of macromolecular secretion machineries to translocate proteins and nucleoprotein complexes from the bacterial cytosol to the host cell. Seven types of secretion systems (I–VII) have so far been identified, with a diverse range of functions including: the transfer of plasmid DNA from one cell to another (the major mechanism for the spread of antibiotic resistance genes between pathogenic bacteria); the secretion of proteins toxic to host cells; and the secretion of effector molecules required for the propagation of the microorganism within the host cell.^1^

Bacterial secretion systems represent attractive targets for the development of novel antibacterial agents.^2^ As these systems are not required for bacterial growth, it is believed that bacteria would be slow to develop resistance to drugs targeting these system. Several groups have developed promising small molecule inhibitors that are effective against the type III secretion systems (T3SS) found in Gram-negative pathogens such as *Yersinia*, *Salmonella* and *Chlamydia*.^2^

Type IV secretion systems (T4SS) are vital for the pathogenicity of a number of important Gram-negative bacteria, such as *Helicobacter pylori*, *Legionella pneumophilia* and *Bordetella pertussis*, which cause serious infections in both animals and plants.^3^, ^4^ *H. pylori* utilizes the type IV secretion system to translocate the toxic protein CagA into gastric epithelial cells, and in doing so induces a number of changes in the host cell.^5^, ^6^ To date, little attention has been paid to T4SS as targets for antibacterial agents, although a VirB8 dimerisation inhibitor has recently been described as a T4SS inhibitor.^7^ Type IV secretion systems require ATP as an energy source to drive this transport and therefore require a class of ATPases known as VirB11 ATPases, which are associated with the inner membrane. The crystal structure of the VirB11 ATPase HP0525 has been solved, with the *apo*-,^8^ ADP bound^9^ and ATPγS bound^8^ forms of HP0525 being studied. The HP0525 forms double hexameric ring structures where each subunit monomer consists of 328 amino acid residues comprising the N-terminal domain (NTD) and C-terminal domain (CTD). Each domain forms a hexameric ring with the CTDs forming a closed ring mounted onto the dynamic open hexameric ring formed by the NTDs. The result is a dome-like structure, open at the NTD end and closed at the CTD end, which is large enough to accommodate a macromolecule such as CagA. The nucleotide-free (*apo*-HP0525) form exists as an asymmetric hexamer with the NTDs displaying mobility and the CTDs maintaining the scaffold. When three molecules of ATP bind, three of the subunits are locked into rigid conformations. Hydrolysis of these three ATP molecules to give ADP, together with binding of a further three ATP molecules to the remaining three nucleotide-free subunits results in a perfect hexameric rigid form. When all ATP molecules are hydrolysed and released the symmetric hexameric ring returns to its asymmetric form. The structure of HP1451, an inhibitory factor of HP0525 which regulates Cag-mediated secretion, bound to HP0525, has also been studied.^10^

Whilst targeting the ATPase activity of HP0525 would represent an attractive approach to generating novel antibacterial agents,^11^ so far only one group have previously published a series of inhibitors of this enzyme.^12^ In this paper, we describe a novel series of imidazo[1,2-*a*]pyrazine derivatives which act as inhibitors of the HP0525 ATPase from *H. pylori*. In the course of this work we have developed a flexible synthetic route to the core heterocycle, which can deliver either 2-aryl or 3-aryl imidazo[1,2-*a*]pyrazines.

## 2 Results and discussion

### 2.1 Lead identification

**Figure 1:**
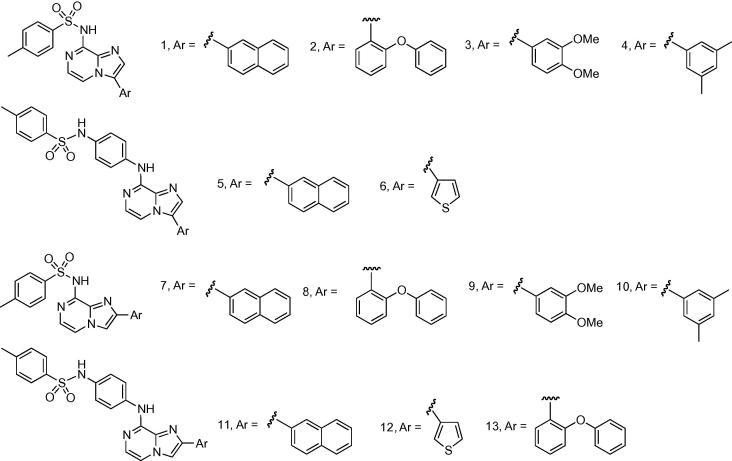
Initial lead structures of 2- and 3-substituted 8-amino imidazo[1,2-*a*]pyrazines.

Structures from the kinase-directed SoftFocus library (BioFocus) were modeled into ATP-binding site of HP0525 ATPase^7^ (1NLY) and scored using DOCK 6.^13^ A group of 3-aryl 8-amino imidazo[1,2-*a*]pyrazines **1**–**6** (Fig. 1) was identified among the best-scoring compounds. These were selected for experimental study, along with the regioisomeric 2-aryl 8-amino imidazo[1,2-*a*]pyrazines **7**–**13** (Fig. 1).

### 2.2 Synthesis of first generation imidazo[1,2-*a*]pyrazine inhibitors

The classical synthetic route to substituted imidazo[1,2-*a*]pyrazines involves a simple condensation between 2-amino pyrazine and chloroacetaldehyde. Previously reported methods^14^ using DMF as a solvent proved to be low yielding, but with methanol as solvent, the product was isolated in 98% yield. However, all subsequent attempts to functionalise the imidazo[1,2-*a*]pyrazine core via bromination^14^, ^15^ gave extremely poor yields and inseparable mixtures of dibrominated regioisomers. Furthermore, these could not be further transformed using telesubstitution with ammonia,^15^, ^16^ to give 8-amino imidazo[1,2-*a*]pyrazines as planned.

**Scheme 1:**
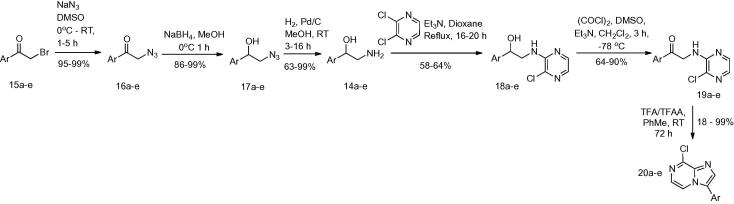
Synthesis of 3-aryl-8-chloroimidazo[1,2-*a*]pyrazines **20a**–**e**.

We therefore planned to prepare the core heterocycle with functional groups already installed at the 2- or 3-positions, and with a leaving group at the 8-position to allow amino or other functionality to be added. In order to access the 3-substituted heterocycles **1**–**6**, we adapted the procedure of MacCoss et al.^17^ to give 3-aryl substituted 8-chloroimidazo[1,2-*a*]pyrazines from 2-aryl-2-hydroxy amines **14a**–**e** (Scheme 1). In each case the synthesis of the amino alcohol was achieved via α-bromination^18^ of the aryl ketone with pyridinium tribromide to give **15a**–**e**. This was followed by substitution of the α-bromine with an azide,^19^ giving **16a**–**e**, which were then reduced^20^ to give alcohols **17a**–**e**. Hydrogenation^21^ to give amino alcohols **14a**–**e** was performed at atmospheric pressure, with the exception of the thiophene analogue which required 3 bar pressure^19^ to go to completion. Coupling with 2,3-dichloropyrazine in 1,4-dioxane afforded the pyrazinyl-amino alcohols **18a**–**e** in good yields. Swern oxidation^22^ to the ketones **19a**–**e** was followed by acid-induced cyclisation to form the 3-aryl-8-chloroimidazo[1,2-*a*]pyrazines **20a**–**e**.

**Scheme 2:**
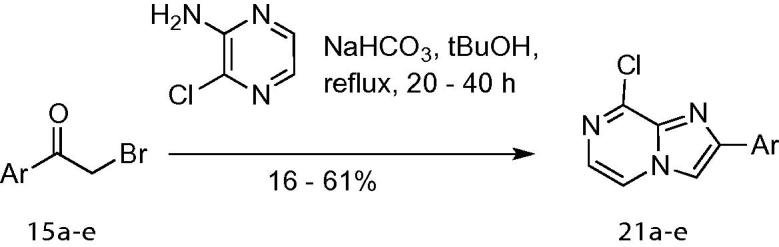
Synthesis of 2-aryl-8-chloroimidazo[1,2-*a*]pyrazines **21a**–**e**.

3-substituted imidazo[1,2-*a*]pyrazine derivatives have previously been reported as kinase inhibitors,^23^, ^24^, ^25^ and a range of strategies is available for synthesis of the key heterocyclic core. However, 2-aryl imidazo[1,2-*a*]pyrazine derivatives have been less frequently explored,^26^ and consequently fewer synthetic approaches to these compounds have been reported. Using the α-bromo aryl ketones **15a**–**e**, the key 2-aryl-8-chloroimidazo[1,2-*a*]pyrazine intermediates **21a**–**e** were readily prepared by condensation with 2-amino-3-chloropyrazine (Scheme 2).

To install the sulfonamido and sulfonamidoaniline groups, Buchwald–Hartwig coupling chemistry was employed.^27^ *t*Bu-XPhos^28^ has been previously used successfully in the Pd-catalysed amidation of aryl bromides with *tert*-butyl carbamate.^29^ The combination of *t*Bu-XPhos/Pd(dba)_2_/K_2_CO_3_/*t*BuOH gave low to moderate yields for all couplings with *p*-toluene sulfonamide **22** and *N*-(4-aminophenyl)-4-methylbenzenesulfonamide **23** (Table 1), with the exception of **21d**, for which Pd(dppf)Cl_2_ had to be used^30^ as an alternative. As the coupling of sulfonamides with aryl chlorides had been reported^31^ to give high yields using DavePhos^32^ a complete range of precatalyst, base, solvent and temperature was screened for the conversion of **21a** to **7** and **11** (Table S1, Supporting information). The combination of Pd_2_(dba)_3_/DavePhos/NaO*^t^*Bu/toluene gave **7** in excellent yield, however this reaction is clearly highly substrate-dependent and these optimised coupling conditions did not give such good results with other 2- and 3-aryl-8-chloroimidazo[1,2-*a*]pyrazines. Likewise, conditions for microwave coupling were explored, which gave excellent yields of **7** but which were less successful for the synthesis of **11** (Table 1).

**Table 1:** Synthesis of 2- and 3-substituted 8-amino imidazo[1,2-*a*]pyrazines from **20a**–**e** and **21a**–**e**

**Figure:**
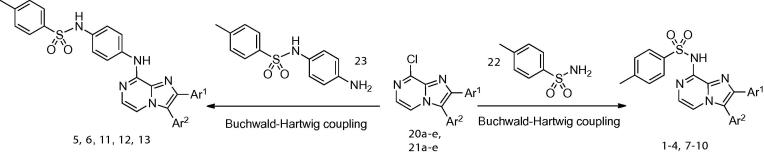

Starting Material	Ar^1^	Ar^2^	Ar^3^NH_2_	Conditions	Product (Yield)
**20a**	H		**22**	*^t^*Bu-XPhos (5 mol %) Pd(dba)_2_ (1 mol %) K_2_CO_3_ (1.2 equiv) *^t^*BuOH, reflux, 40 h	**1** (41%)
**20b**	H		**22**	*^t^*Bu-XPhos (5 mol %) Pd(dba)_2_ (1 mol %) K_2_CO_3_ (1.2 equiv) *^t^*BuOH, reflux, 40 h	**2** (26%)
**20c**	H		**22**	*^t^*Bu-XPhos (5 mol %) Pd(dba)_2_ (1 mol %) K_2_CO_3_ (1.2 equiv) *^t^*BuOH, reflux, 40 h	**3** (30%)
**20d**	H		**22**	*^t^*Bu-XPhos (5 mol %) Pd(dba)_2_ (1 mol %) K_2_CO_3_ (1.2 equiv) *^t^*BuOH, reflux, 40 h	**4** (67%)
**20a**	H		**23**	*^t^*Bu-XPhos (5 mol %) Pd(dba)_2_ (1 mol %) K_2_CO_3_ (1.2 equiv) *^t^*BuOH, reflux, 46 h	**5** (35%)
**20e**	H		**23**	DavePhos (3 mol %), Pd_2_(dba)_3_ (1 mol %) NaO*^t^*Bu (1.4 equiv) toluene, reflux, 24 h	**6** (30%)
**21a**		H	**22**	DavePhos (3 mol %), Pd_2_(dba)_3_ (1 mol %) NaO*^t^*Bu (1.4 equiv) toluene, reflux, 24 h	**7** (93%)
**21a**		H	**22**	DavePhos (3 mol %), Pd_2_(dba)_3_ (1 mol %) NaO*^t^*Bu (1.4 equiv) toluene, 160 °C (μW), 10 min	**7** (46%)
**24**		H	**22**	NaH (2 equiv), DMF, 100 °C, 20 h	**7** (72%)
**21b**		H	**22**	*^t^*Bu-XPhos (5 mol %) Pd(dba)_2_ (1 mol %) K_2_CO_3_ (1.2 equiv) *^t^*BuOH, reflux, 48 h	**8** (13%)
**21c**		H	**22**	*^t^*Bu-XPhos (5 mol %) Pd(dba)_2_ (1 mol %) K_2_CO_3_ (1.2 equiv) *^t^*BuOH, reflux, 48 h	**9** (2%)
**21d**		H	**22**	Pd(dppf)_2_Cl_2_ (2 mol %), K_2_CO_3_ (1.2 equiv), *^t^*BuOH, reflux, 21 h	**10** (29%)
**21a**		H	**23**	DavePhos (3 mol %), Pd_2_(dba)_3_ (1 mol %) NaO*^t^*Bu (1.4 equiv) toluene, reflux, 24 h	**11** (22%)
**21a**		H	**23**	DavePhos (3 mol %), Pd_2_(dba)_3_ (1 mol %) NaO*^t^*Bu (1.4 equiv) toluene, 160 °C (μW), 10 min	**11** (47%)
**21e**		H	**23**	*^t^*Bu-XPhos (5 mol %) Pd(dba)_2_ (1 mol %) K_2_CO_3_ (1.2 equiv) *^t^*BuOH, reflux, 48 h	**12** (8%)
**21b**		H	**23**	DavePhos (3 mol %), Pd_2_(dba)_3_ (1 mol %) NaO*^t^*Bu (1.4 equiv) toluene, reflux, 24 h	**13** (9%)

**Scheme 3:**
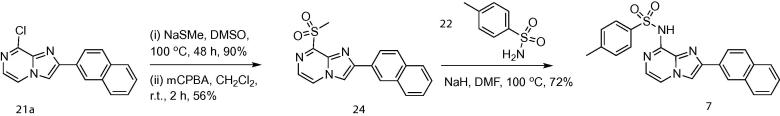
Alternative route to **7** via methylsulfone **24**.

Methyl sulfones have previously been used to attach a variety of nucleophiles onto imidazo[1,2-*a*]pyrazine rings.^24^, ^33^ As an alternative to the Buchwald–Hartwig coupling, **21a** was converted to methyl sulfone **24** (Scheme 3). Treatment with 4-toluene sulfonamide/NaH/DMF gave **7** in good yield, however for this substrate no improvement over the optimised Buchwald–Hartwig conditions could be effected via this route.

The regiochemistry of the 2- and 3-substituted imidazo[1,2-*a*]pyrazines was confirmed by 2D NMR. For the 2-substituted imidazo[1,2-*a*]pyrazine **21a**, HMBC (Fig. S1, Supporting information) showed correlation between the protons and carbons in the 3- and 5-positions of the imidazo[1,2-*a*]pyrazine rings. Conversely, with the 3-substituted imidazo[1,2-*a*]pyrazines no correlation was observed between the protons in the corresponding 2- and 5-positions.

### 2.3 Biochemical evaluation of first generation imidazo[1,2-*a*]pyrazine inhibitors

**Figure 2:**
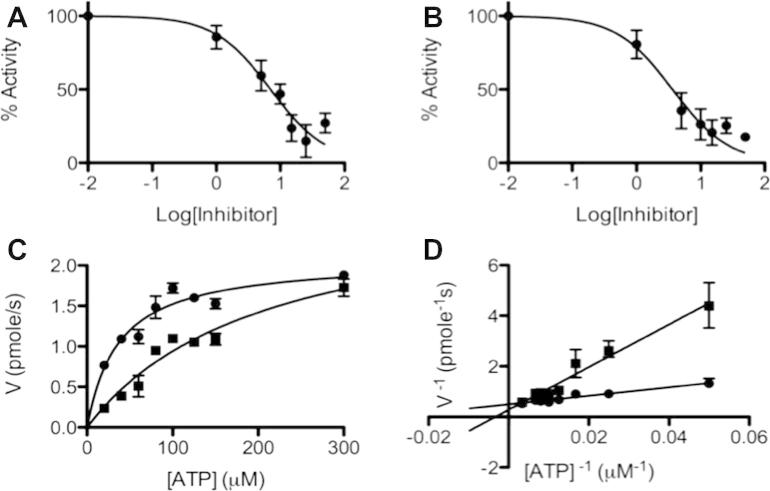
Dose-dependent and steady-state inhibition of HP0525. Dose–response curves used for IC_50_ estimations of compounds **11** (A) and **32** (B). Michaelis–Menten (C) and Lineweaver–Burk (D) plots, corresponding to ✠ without inhibitor, and ✠ with **11**. Error bars represent standard deviations using triplicate data.

The HP0525 protein was produced recombinantly in *Escherichia coli* and purified to high purity as described previously.^10^ The ATPase activity of HP0525 was measured by monitoring the release of inorganic phosphate (P_i_) using an in vitro ATPase assay (see Section 4 and SI). Initially, the inhibitory effects of the compounds were evaluated by performing the ATPase assay with and without compound present at concentrations of 500 μM (or 250 μM), 50 μM and 5 μM (data not shown). The compounds with inhibitory activities were further analyzed for dose-dependency by estimating their IC_50_s (Fig. 2). Those with the highest inhibitory effect, **11**, **5** and **6** with IC_50_s of 6, 20 and 48 μM, respectively, showed similarities in their chemical structures, see below.

To test our hypothesis that the inhibitors bind in the substrate pocket, as suggested by the molecular docking, we tested the mode of inhibition of **11**. Steady-state kinetic data displayed Michaelis–Menten behavior, and **11** unambiguously behaved as a competitive inhibitor (Fig. 2).

We verified that our analogues displayed suitable physicochemical profiles by calculating log *P* and log *S* using *A* log *P*2.1 applet.^34^ The results presented in Table 2 suggest that our first generation of compounds display limited solubility (0.1 to 10 mg/L) and log *P* values that are high but within limits described by the Lipinski’s ‘rule of five’ (i.e., ⩽5).^35^

**Table 2:** IC_50_ values for first generation compounds

**Figure:**
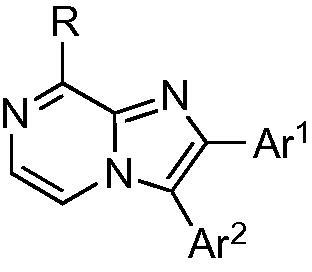

Compound	R	Ar^1^	Ar^2^	IC_50_/μM	*A* log *sP*	*A* log *pS*
**1**		H		77 ± 16	3.7	−5.7
**2**		H		96 ± 42	4.3	−5.8
**3**		H		144 ± 14	3.1	−4.7
**4**		H		154 ± 22	3.3	−5.4
**5**		H		20 ± 3	4.6	−6.0
**6**		H		48 ± 7	3.5	−5.1
**7**			H	88 ± 14	4.3	−5.7
**8**			H	82 ± 14	4.5	−5.8
**9**			H	167 ± 43	3.5	−4.7
**10**			H	133 ± 16	3.5	−5.4
**11**			H	6 ± 1	5.0	−6.0
**12**			H	99 ± 18	3.9	−5.1
**13**			H	18 ± 4	5.5	−6.2

### 2.4 Second generation imidazo[1,2-*a*]pyrazine inhibitors

The promising IC_50_ values observed for these compounds, and especially for **11**, prompted a further investigation of related structures. Table 2 shows that compounds with substituents in the 8-position where the sulfonamide moiety was remote from the core heterocycle showed the greatest potency. In particular, **11** showed the most promise as a lead for further investigation. However, the physicochemical properties (partition coefficient and solubility) of this series of compounds, were poor, and we therefore sought to explore structural modifications that would give candidates suitable for preclinical drug development with improved solubility as well as potency. As the sulfonamide could potentially act as a bioisostere of one of the phosphate groups of ATP,^36^ we aimed to evaluate analogues with different spacing and flexible or rigid linkers between the core heterocycle and the sulfonamide, and also analogues with the sulfonamide group absent. Analogues with heterocyclic groups at position 2, or lacking the aryl group altogether, were also evaluated, in a further effort to improve the solubility of this series. Finally, substitution at position 6 was also investigated.

A series of analogues of **11**, differing at the 8-position, were prepared (Table 3) via either Buchwald–Hartwig coupling from **21a** or nucleophilic substitution of the methyl sulfone **24**, in moderate to good yields. Deleting the arylsulfonamide group completely (**25**) resulted in complete loss of activity, further suggesting that this region of the 8-substituted imidazo[1,2-*a*]pyrazines is important for binding to the active site. The majority of variants of the *N*-(4-aminophenyl)-4-methylbenzenesulfonamido group (**26**–**30**) showed a decrease in potency compared to **11** when the sulfonamido group was placed at a greater distance from the imidazo[1,2-*a*]pyrazine ring, modified, or deleted completely. However, when the aniline group was replaced by an ethyl linker (**31**) comparable inhibition with better solubility and log *P* were obtained. Replacing the *p*-toluene sulfonamidyl group with quinoline-8-sulfonamide (**33**, **34**) also resulted in compounds of high potency. Surprisingly, when the *N*-(4-aminophenyl)-4-methylbenzenesulfonamido group was replaced by 3-(pyridin-3-yl)aniline (**32**) a further slight improvement in potency was seen (Table 3). Unfortunately, the most active compounds in this series (**32**, **33** and **34**) all exhibited comparable solubility and log *P* to **11**.

**Table 3:** Structures and IC_50_ values for 2nd generation compounds, varying at 8-position

**Figure:**
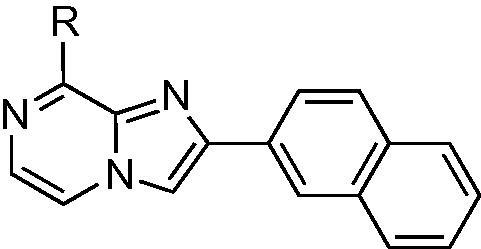

Compound	R	Synthesis method	IC_50_/μM	*A* log *sP*	*A* log *pS*
**25**	NH_2_	—	>1000	3.2	−4.3
**26**		A	18 ± 5	4.4	−5.3
**27**		A	61 ± 28	4.0	−5.4
**28**		A	29 ± 9	5.0	−6.0
**29**		A	75 ± 35	5.3	−6.0
**30**		B	58 ± 38	4.3	−5.7
**31**		B	7 ± 2	4.1	−5.6
**32**		A	4 ± 1	5.0	−5.8
**33**		A	7 ± 2	5.1	−6.0
**34**		A	7 ± 2	5.0	−6.0

Method A: Buchwald–Hartwig coupling from **21a**. Method B: NaH, **24**. Section 4 are given in the Supporting information.

The importance of the aryl substituent in position 2 was further reinforced by compound **35**; deleting the aryl substituent resulted in a complete loss of activity (Table 4). As the naphthalene substituent is highly lipophilic and is a major contributor to the insolubility of this series of inhibitors, we replaced this with a quinoxaline (**36**). However, this gave no appreciable improvement in either potency or solubility.

**Table 4:** Variants of the lead compound **11** at the 2- and 6-positions

Compound		IC_50_/μM	*A* log *sP*	*A* log *pS*
**35**		>1000	2.7	−4.8
**36**		28 ± 6	4.2	−5.3
**37**		6 ± 2	6.6	−6.3
**38**		77 ± 20	5.0	−5.8

**Scheme 4:**
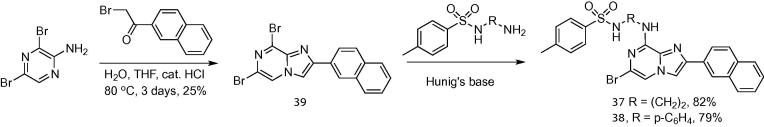
Synthesis of **37** and **38** via 6,8-dibromoimidazo[1,2-*a*]pyrazine **39**.

In order to explore the effects of substitution at the 6-position of the imidazo[1,2-*a*]pyrazine, **37** and **38** were synthesized. The key 6,8-dibromoimidazo[1,2-*a*]pyrazine **39** was prepared by condensation of 4,6-dibromo-2-aminopyrazine with 2-bromoacetyl naphthalene (Scheme 4). Nucleophilic reaction with the appropriate monotosylated diamine proceeded smoothly and exclusively at the 8-position to afford **37** or **38** in good yield. However, **38** showed poorer physicochemical properties and IC_50_ compared to the analogue **31**. Likewise, **37** showed poorer solubility and log *P* compared with **11**, which lacks the 6-substitutent, but showed comparable potency (Table 4). Overall, this suggests that the bromide substituent at the 5-position does not improve potency and leads to a poorer physicochemical profile.

### 2.5 Docking studies

**Figure 3:**
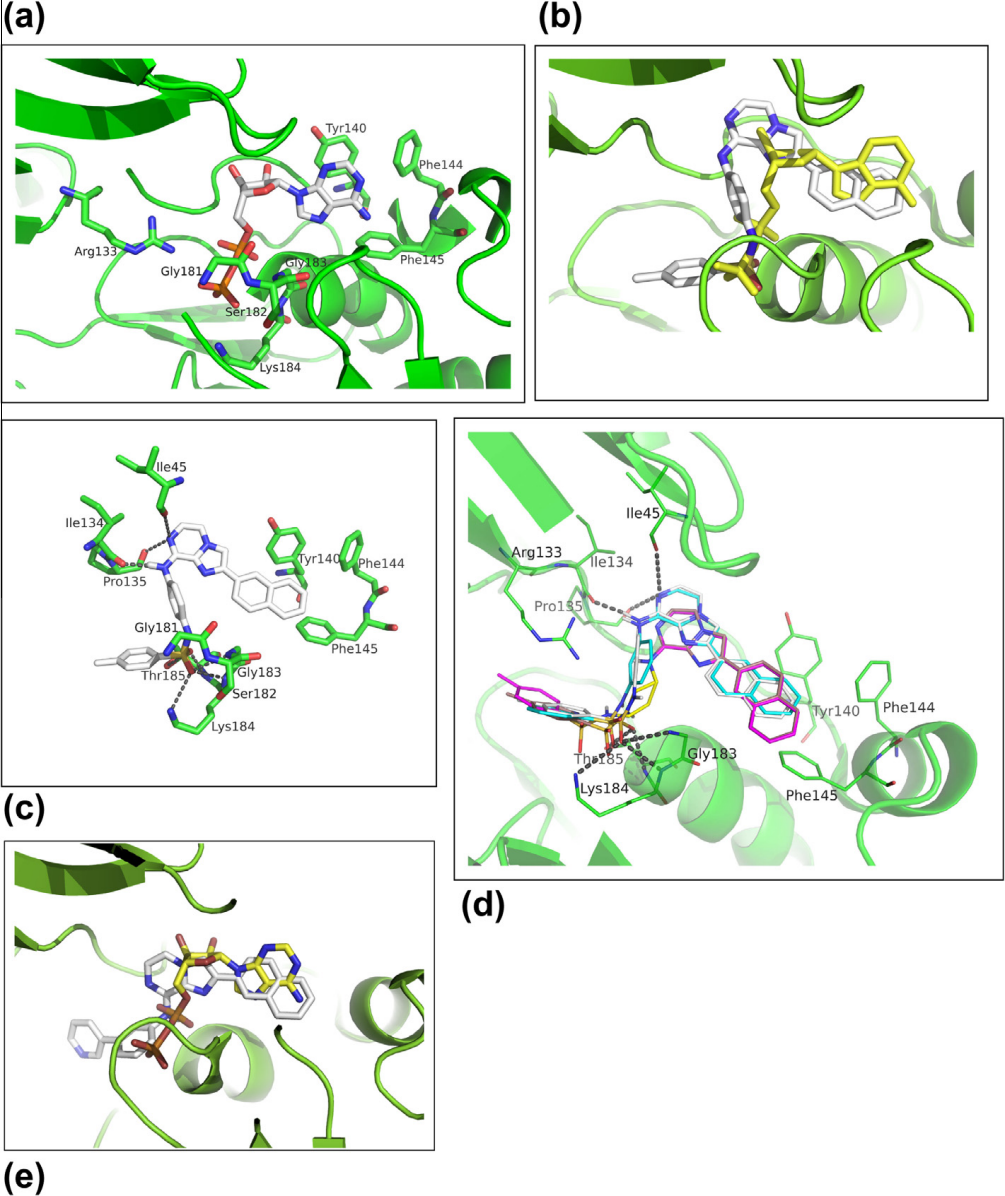
(a) Binding of ADP to HP0525 (PDB 1G6O). Key residues are labelled. (b) Comparison of the binding of **11** and ADP (Yellow). (c) Binding of **11** to HP0525, showing regions that could be explored to increase the potency of the series of inhibitors. (d) Compounds **11** (white), **30** (magenta), **31** (violet-brown) and **34** (turquoise) are overlaid, when docked in HP0525. Polar bonds are indicated corresponding to interactions involving compound **11**. (e) Comparison of the docking of **32** (white/blue) and ADP in HP0525.

In order to further understand the binding of these imidazo[1,2-*a*]pyrazines to HP0525, and to direct the design of more potent inhibitors, molecular docking studies were carried out using AutoDock Vina.^37^ Structural studies showed that the conformation of the apo form of each unit is variable, but the two structures 1G6O^9^ and 1NLY^8^ which possess ADP, or the ATP mimic ATP-γS, respectively, are structurally highly similar with an RMS of 0.50 Å for CA atoms and 0.78 Å for all atoms. Both of these structures contain two identical chains, A and B. For drug design purposes any of the four chains from the two crystal structures above are acceptable for use in modelling/screening. Investigation of the ligand–protein interactions of each, in particular the hydrogen bonding, reveals a more extensive network of interactions in 1NLY and the A chain also includes the active site metal (Mg). This might therefore be taken to be the more physiological representative structure. However, the resolution of 1G6O is 2.50 Å compared to 2.80 Å for 1NLY, and a Ramachandran^38^ analysis gives 4% of residues in more favourable regions for 1G6O. The crystal structure of the ADP-bound HP0525, 1G6O, with heteroatoms and ADP removed, was therefore used for the docking studies. Examination of the ADP/ATP binding pocket shows that it adopts a conical topology in which the entrance is wide open and the bottom of the cavity very narrow and very likely to tolerate small groups only. In accordance with kinases and phosphorylases topologies the entrance to the active side is highly lipophilic and the end of the cavity highly hydrophilic. In the entrance to the active site (adenosine binding region), lipophilicity is governed by three aromatic residues (Tyr140, Phe144 and Phe145). Hydrophilicity within the cavity (triphosphate binding region) is created by a tetrad Gly181/Ser182/Gly183/Lys184 along with Arg133. A third binding region, not exploited by ADP/ATP, is located in the direction of the 2′-ribose hydroxyl and is likely to tolerate small aliphatic moieties (Fig. 3a).

Docking of the lead compound **11** (Fig. 3b), and comparison with the binding of ADP, showed a binding mode in which the inhibitor is deeply buried within the enzymatic cavity. In this orientation, the naphthalene group occupies the purine-binding region of the active site, possibly making π-stacking interactions with Phe145. The core imidazo[1,2-*a*]pyrazine ring sits in place of the ribose moiety of ADP, with the sulfonamide occupying the phosphate binding region and making polar contacts with Gly181, Lys184 and Thr185. Examining the surface of the ADP binding site with the docked **11** (Fig. 3c) suggested that these two binding regions could be explored to increase the potency of this series of inhibitors. For example, in order to optimize the predicted interaction of the sulfonamide moiety with the phosphate binding site, a series of analogues (**30**, **31**, **33**, **34**) in which different spacer lengths and orientations between the sulfonamide and the 8-position were synthesized and tested. Docking studies on **30** (IC_50_ 58 μM), **31** (IC_50_ 7 μM), and **34** (IC_50_ 7 μM) suggest that the propyl chain of **30** may position the sulfonamide moiety too far from the phosphate binding region (Fig. 3d). Intriguingly, the most potent lead compound in this series, **32**, which is also the most ligand efficient, lacks the sulfonamide group completely. Here the docking (Fig. 3e) suggests a similar orientation, with the naphthalene group occupying the purine-binding region, and the imidazo[1,2-*a*]pyrazine ring occupying the ribose-binding area. However, the 3-(pyridin-3-yl)aniline moiety in this case occupies the phosphate binding region. This may indicate that the sulfonamide group is not always necessary for binding if other H-bond donor or acceptor groups are present in the correct orientation.

## 3 Conclusions

Following a virtual high throughput screen, a novel series of 8-amino imidazo[1,2-*a*]pyrazine derivatives have been developed, using a flexible synthetic route to deliver 2- and 3-aryl regioisomers. Biochemical evaluation showed moderate to good potency highlighting this class of compound as competitive inhibitors of the HP0525 ATPase from *H. pylori*, with potential as antibacterial agents. The structure–activity relationships of these 8-amino imidazo[1,2-*a*]pyrazines has been explored through docking studies, however co-crystallisation of these inhibitors with HP0525 is imperative to fully understand the interaction within the nucleotide binding site and aid in the development of more potent inhibitors. Furthermore, development of these compounds will require improvements in their aqueous solubility to enable a more suitable physicochemical profile.

## 4 Experimental section

### 4.1 Lead identification and molecular docking

The initial lead compounds were identified through screening of the SoftFocus kinase-targeted compound library (BioFocus). The pharmacophore alignment function of MOE^39^ was used to produce a rough initial alignment to ATP of the diverse input set based on common chemical features. The aligned structures were then energy-minimised in the context of the nucleotide binding site of HP0525 (PDB ID 1NLY^8^), with the atoms of the protein frozen, using NAMD.^40^ Ligand force field parameters were assigned according to the General Amber Force Field.^41^ The resulting binding poses were scored using the Hawkins GB/SA function of DOCK 6.^13^

Further molecular docking studies were carried out using AutoDock Vina.^37^ The crystal structure of ADP-HP0525 (PDB entry 1G60) was used to define a docking grid around the nucleotide binding site, with a size of 14 × 16 × 24 and a grid center of −12.034, 24.627 and 22.363 in the *x*, *y*, and *z* coordinates, respectively. An ‘exhaustiveness’ parameter of 8 was used. Ligand structures were generated using chem3D pro and further prepared using AutoDock Tools (ADT)^42^ as recommended in the documentation.

### 4.2 General chemistry

Melting points (Mp) were recorded on a Gallenkamp Melting Point Apparatus and are uncorrected. ^1^H and ^13^C NMR were recorded using Bruker AV400 (400 and 100 MHz, respectively), AV500 (500 and 125 MHz, respectively) and AV600 (600 and 150 MHz, respectively) spectrometers as indicated. Chemical shifts are quoted on the *δ* scale in units of ppm using TMS as an internal standard. Spectra were obtained using CDCl_3_, CD_3_OD, CD_2_Cl_2_ and DMSO-*d*_6_ as solvents and coupling constants (*J*) are reported in Hz with the following splitting abbreviations: s (singlet), d (doublet), t (triplet), dd (doublet of doublets), bs (broad singlet). Infra-Red (IR) spectroscopy was carried out using a PerkinElmer Spectrum 100 FT-IR Spectrometer using thin films. Absorption maxima (*ν*_max_) are reported in wavenumbers (cm^−1^).

Solvents and reagents were obtained from commercial sources and were used as received unless otherwise stated. Petroleum ether refers to the fraction of light petroleum ether boiling in the range 40–60 °C.

Representative examples of each of the synthetic routes shown in Scheme 1, Scheme 2, Scheme 3 are given. Full experimental for the preparation of the remaining compounds, and full compound characterisation, is given in the Supplementary data.

### 4.3 General method for synthesis of α-bromo aryl ketones, illustrated for the preparation of 2-bromo-1-(2-phenoxyphenyl)ethanone, **15b**

1-(2-Phenoxyphenyl)ethanone (2.00 g, 9.42 mmol) was dissolved in chloroform (60 mL) and ethanol (60 mL). Pyridinium tribromide (7.50 g, 23.6 mmol) was added and the reaction was stirred at 50 °C for 16 h. The reaction mixture was cooled to room temperature and the solvents removed in vacuo. The resulting orange slurry was suspended in H_2_O (30 mL) and extracted with EtOAc (4 × 30 mL). The combined organic extracts were washed with H_2_O (2 × 20 mL) and brine (1 × 20 mL), dried (Na_2_SO_4_), filtered and concentrated in vacuo to give a yellow oil. Flash chromatography was carried out (applied in petroleum ether; eluted 0% to 10% to 33% CH_2_Cl_2_) to afford the title compound as a pale yellow oil (2.30 g, 7.90 mmol, 84%). *R_f_ *= 0.68 (CH_2_Cl_2_); IR (*ν*_max_/cm^−1^, thin film): 1677, 1598, 1574; ^1^H NMR (500 MHz, CDCl_3_): *δ*_H_ = 4.65 (s, 2H), 6.86 (d, *J* = 8.4 Hz, 1H), 7.09 (d, *J* = 7.7 Hz, 2H), 7.17 (t, *J* = 7.6 Hz, 1H), 7.22 (t, *J* = 7.3 Hz, 1H), 7.40–7.47 (m, 3H), 7.92 (dd, *J* = 7.6, 1.5 Hz, 1H); ^13^C NMR (125 MHz, CDCl_3_): *δ*_C_ = 36.8, 117.6, 119.5, 123.0, 124.4, 126.2, 129.9, 131.3, 134.2, 155.0, 156.5, 191.6; LRMS *m*/*z* (EI^+^): 292 [M (^81^Br)]^+^, 290 [M (^79^Br)]^+^, 212 [M−Br]^+^, 197 [M−CH_2_Br]^+^; HRMS *m*/*z* (EI^+^): Found 289.99403 [M(^79^Br)]^+^; C_14_H_11_BrO_2_ requires 289.99369.

### 4.4 General method for synthesis of α-azido aryl ketones, illustrated for the preparation of 2-azido-1-(2-naphthyl)ethanone **16a**

2-(Bromoacetyl)naphthalene (2.00 g, 8.03 mmol) was dissolved in DMSO (10 mL) and the mixture was cooled on ice such that the temperature was kept below 10 °C. Sodium azide (0.630 g, 9.64 mmol) was added in one portion and the reaction was stirred under argon at room temperature for 90 min. The reaction was quenched with H_2_O (20 mL), and extracted with EtOAc (3 × 30 mL). The organic layers were combined, washed with H_2_O, dried (Na_2_SO_4_) and filtered. The solvent was removed in vacuo to give the title compound as a brown/orange oil (1.69 g, 8.01 mmol, 100%) with NMR consistent with literature values.^43^ *R_f_ *= 0.63 (5:1 petroleum ether/EtOAc); IR (*ν*_max_/cm^−1^, thin film): 2105, 1690; ^1^H NMR (600 MHz, CDCl_3_): *δ*_H_ = 4.73 (s, 2H), 7.59–7.62 (m, 1H), 7.65–7.68 (m, 1H), 7.91 (d, *J* = 8.1 Hz, 1H), 7.95 (d, *J* = 8.6 Hz, 1H), 7.99–8.01 (m 2H), 8.42 (s, 1H); ^13^C NMR (150 MHz, CDCl_3_): *δ*_C_ = 55.0, 123.3, 127.2, 127.9 129.0, 129.1, 129.6, 129.8, 131.7, 132.4, 136.0, 193.2; LRMS *m*/*z* (EI^+^): 211 [M]^+^, 155 [M−CH_2_N_3_]^+^, 127 [Naphthalene]^+^.

### 4.5 General method for synthesis of α-azido aryl alcohols, illustrated for the preparation of 2-azido-1-(2-naphthyl)ethanol **17a**

Azidoketone **16a** (2.11 g, 10.0 mmol) was dissolved in anhydrous MeOH (100 mL) and cooled on ice. Sodium borohydride (568 mg, 15.0 mmol) was added portion wise and the mixture was stirred on ice under argon for 1 h until the reaction had gone to completion by TLC. The solvent was removed and the resulting residue was taken up in CH_2_Cl_2_ (100 mL) and carefully washed with H_2_O (2 × 60 mL) followed by brine (60 mL). The organic extracts were dried over Na_2_SO_4_, filtered and concentrated in vacuo to give the title compound as a brown oil (2.14 g, 10.0 mmol, 100%). Spectroscopic data (for the racemic material) was consistent with that previously reported^44^ (for the (*S*)-enantiomer): *R_f_ *= 0.65 (3:1 petroleum ether/EtOAc); IR (*ν*_max_/cm^−1^, thin film): 3398, 2100; ^1^H NMR (500 MHz, CDCl_3_): *δ*_H_ = 2.70 (br s, 1H), 3.46–3.58 (m, 2H), 5.02 (dd, *J* = 8.1, 3.9 Hz, 1H), 7.44 (dd *J* = 8.4, 1.6 Hz, 1H), 7.49–7.52 (m, 2H), 7.83–7.86 (m, 4H); ^13^C NMR (125 MHz, CDCl_3_): *δ*_C_ = 58.1, 73.6, 123.7, 125.1, 126.4, 126.5, 127.8, 128.1, 128.6, 133.3 (2 signals), 138.0; LRMS *m*/*z* (EI^+^): 221, 157 [M−CH_2_N_3_]^+^, 147, 129.

### 4.6 General method for synthesis of α-amino aryl alcohols, illustrated for the preparation of 2-amino-1-(2-naphthyl)ethanol **14a**

Azidoalcohol **17a** (2.18 g, 10.2 mmol) was dissolved in anhydrous MeOH (50 mL) and 10% palladium on carbon (218 mg, 10% w/w) was added. The vessel was evacuated and purged with Ar (3×) and under static vacuum a balloon of hydrogen was added. The reaction mixture was stirred under hydrogen atmosphere until completion as determined by TLC and disappearance of N_3_ peak by IR. After 3½ h, the hydrogen was carefully released, the vessel evacuated and purged argon (3×), and the reaction mixture was filtered through Celite (pre-washed with MeOH). Solvent removal in vacuo gave the crude compound as a orange oil (1.91 g, 10.2 mmol, 100%). Spectroscopic data was consistent with that previously reported.^44^ *R_f_ *= 0.06 (5:1 EtOAc/MeOH); IR (*ν*_max_/cm^−1^, thin film): 3290, 3054, 2916, 1599; ^1^H NMR (500 MHz, CDCl_3_): *δ*_H_ = 2.82 (m, 2H), 4.59–4.75 (m, 1H), 7.36–7.40 (m, 3H), 7.74–7.76 (m, 4H); ^13^C NMR (125 MHz, CDCl_3_): *δ*_C_ = 49.8, 74.3, 123.8, 124.5, 125.7, 126.0, 127.5, 128.1, 132.8, 133.1 (2 signals), 139.7; LRMS *m*/*z* (ESI^+^): 229.2 [M+MeCN]^+^, 211.2 [M+Na]^+^, 188.1 [M+H]^+^, 170 [M−OH]^+^.

### 4.7 General method for synthesis of 2-[(3-chloropyrazin-2-yl)amino]-1-(2-aryl)ethanol, illustrated for the preparation of 2-[(3-chloropyrazin-2-yl)amino]-1-(2-naphthyl)ethanol **18a**

Amino alcohol **14a** (289 mg, 1.55 mmol), 2,3-dichloropyrazine (177 μL, 1.70 mmol) and Et_3_N (301 μL, 2.16 mmol) were dissolved in 1,4-dioxane (3 mL) and the reaction was stirred under reflux, under argon. After 19 h, the reaction was cooled to room temperature and the solvent removed in vacuo. The residue was taken up in CH_2_Cl_2_ and washed with H_2_O (3 × 20 mL) and brine (1 × 20 mL). The organic extracts were dried (Na_2_SO_4_), filtered and concentrated to give the crude product as an amber oil. Purification was carried out via flash chromatography (applied in CH_2_Cl_2_; eluted 0% to 33% EtOAc) to afford the title compound as a yellow oil (295 mg, 0.983 mmol, 63%). *R_f_ *= 0.64 (2:1 CH_2_Cl_2_/EtOAc); IR (*ν*_max_/cm^−1^, thin film): 3419, 3054, 2922, 1523; ^1^H NMR (500 MHz, CDCl_3_): *δ*_H_ = 3.65–3.70 (m, 1H), 3.88 (br s, 1H), 3.92–3.97 (m, 1H), 5.12 (dd, *J* = 7.5, 2.8 Hz), 5.67 (t, *J* = 5.3 Hz), 7.47–7.50 (m, 3H), 7.59 (d, *J* = 2.7 Hz, 1H), 7.81–7.84 (m, 3H), 7.86 (s, 1H), 7.91 (d, *J* = 2.7 Hz, 1H); ^13^C NMR (125 MHz, CDCl_3_): *δ*_C_ = 49.4, 73.9, 123.9, 124.8, 126.1, 126.4, 127.8, 128.0, 128.4, 131.3, 133.2, 133.3, 135.1, 139.4, 140.2, 151.5; LRMS *m*/*z* (ESI^+^): 300.1 [M(^35^Cl)+H]^+^, 284.2 [M(^37^Cl)−OH]^+^, 282.2 [M(^35^Cl)−OH]^+^; HRMS *m*/*z* (ESI^−^): Found 298.0731 [M(^35^Cl)−H]^−^; C_16_H_13_ClN_3_O requires 298.0747.

### 4.8 General method for synthesis of 2-[(3-chloropyrazin-2-yl)amino]-1-(2-aryl)ethanones, illustrated for the preparation of 2-[(3-chloropyrazin-2-yl)amino]-1-(2-naphthyl)ethanone **19a**

DMSO (982 μL, 13.9 mmol) was dissolved in anhydrous CH_2_Cl_2_ (60 mL) and the reaction mixture was cooled to and maintained at −78 °C. Oxalyl chloride (586 μL, 6.93 mmol) was added drop wise and the mixture was stirred for 20 min. **17a** (1.60 g, 5.33 mmol), dissolved in anhydrous CH_2_Cl_2_ (40 mL) was added dropwise, and stirred for 20 min. Et_3_N (3.54 mL, 26.6 mmol) was added dropwise and the reaction mixture was allowed to warm to room temperature over a period of 2.5 h. The reaction was then quenched with H_2_O (50 mL) and organics extracted, which were then washed with 2.0 M HCl (2 × 40 mL), NaHCO_3_ (satd aq 40 mL), H_2_O (40 mL) and brine (40 mL). The organic layer was dried (MgSO_4_), filtered and the solvent removed in vacuo to give a yellow/orange solid. Flash chromatography (applied in CH_2_Cl_2_; eluted 100:1 to 30:1 CH_2_Cl_2_/EtOAc) afforded the title compound as a yellow solid (903 mg, 3.03 mmol, 57%). Mp: 160 °C; *R_f_ *= 0.30 (30:1 CH_2_Cl_2_/EtOAc); IR (*ν*_max_/cm^−1^, thin film): 1680; ^1^H NMR (500 MHz, CDCl_3_): *δ*_H_ = 5.10 (d, *J* = 4.3, 2H), 6.54 (s, 1H), 7.58–7.62 (m, 1H), 7.64–7.67 (m, 1H), 7.68 (d, *J* = 6.1 Hz, 1H), 7.91 (d, *J* = 8.0 Hz, 1H), 7.96 (d, *J* = 8.6 Hz, 1H), 8.00–8.02 (m, 2H), 8.10 (dd, *J* = 8.6, 1.8 Hz, 1H), 8.62 (s, 1H); ^13^C NMR (125 MHz, CDCl_3_): *δ*_C_ = 48.4, 123.4, 127.3, 128.0, 129.0, 129.2, 129.8, 130.1, 131.3, 131.8, 132.6, 136.2, 139.7, 193.8; LRMS *m*/*z* (ESI^+^): 300 [M(^37^Cl)+H]^+^, 298 [M(^35^Cl)+H]^+^, 282 [M(^37^Cl)−OH]^+^, 280 [M(^35^Cl)−OH]^+^; HRMS *m*/*z* (ESI^−^): Found 296.0591 [M(^35^Cl)−H]^−^; C_16_H_11_ClN_3_O requires 296.0591.

### 4.9 General method for synthesis of 3-aryl-8-chloro-imidazo[1,2-*a*]pyrazines, illustrated for the preparation of 8-chloro-3-(2-naphthyl)imidazo[1,2-*a*]pyrazine **20a**

Compound **19a** (903 mg, 3.03 mmol) was dissolved in anhydrous toluene (40 mL) and the mixture was cooled on ice. Trifluoroacetic acid (1.64 mL, 21.2 mmol) was added and the reaction was allowed to stir on ice for 30 min, followed by the addition of trifluoroacetic anhydride (2.95 mL, 21.2 mmol). The reaction mixture was then stirred on ice for a further 30 min and then at room temperature for 65 h. The reaction was then diluted with toluene (20 mL) and washed with NaHCO_3_ solution (10% w/v, 3 × 20 mL) and brine (20 mL). The organics were dried (MgSO_4_), filtered and concentrated to give the crude product as an amber oil. Purification was carried out via flash chromatography (applied in CH_2_Cl_2_; eluted 80:1 to 10:1 CH_2_Cl_2_/EtOAc) to afford the title compound as an off white solid (386 mg, 1.38 mmol, 45%). Mp: 166 °C; *R_f_ *= 0.21 (10:1 CH_2_Cl_2_/EtOAc); IR (*ν*_max_/cm^−1^, thin film): 3102, 3052; ^1^H NMR (500 MHz, CDCl_3_): *δ*_H_ = 7.58–7.62 (m, 2H), 7.64 (dd, *J* = 8.6, 1.7 Hz, 1H), 7.73 (d, *J* = 4.6 Hz, 1H), 7.92–7.95 (m, 2H), 8.02 (s, 1H), 8.04–8.05 (m, 1H), 8.04 (s, 1H), 8.30 (d, *J* = 4.6 Hz, 1H); ^13^C NMR (125 MHz, CDCl_3_): *δ*_C_ = 116.4, 124.7, 125.2, 127.3, 127.4, 127.6, 128.0, 128.2, 128.6, 129.4, 129.7, 133.4, 133.5, 134.8, 138.4, 144.5; LRMS *m*/*z* (ESI^+^): 282 [M(^37^Cl)+H]^+^, 280 [M(^35^Cl)+H]^+^; HRMS *m*/*z* (ESI^+^): Found 280.0646 [M(^35^Cl)+H]^+^; C_16_H_11_ClN_3_ requires 280.0642.

### 4.10 General method for the synthesis of 4-methyl-*N*-[4-[3-arylimidazo[1,2-*a*]pyrazine-8-yl]]-sulfonamides, illustrated for the preparation of 4-methyl-*N*-[4-[3-(2-naphthyl)imidazo[1,2-*a*]pyrazine-8-yl]aminophenyl]benzenesulfonamide **5**

All glassware was evacuated and flushed with argon prior to use. Compound **20a** (283 mg, 1.01 mmol), *N*-(4-aminophenyl)-4-methylbenzenesulfonamide **23** (318 mg, 1.21 mmol), K_2_CO_3_ (167 mg, 1.21 mmol), Pd(dba)_2_ (5.80 mg, 1 mol %) and *tert-*butyl XPhos (21.5 mg, 5 mol %) were taken up in *^t^*BuOH (6 mL) and the reaction was stirred under reflux under Ar for 46 h. The reaction mixture was cooled to room temperature, diluted with MeOH (100 mL) and filtered through Celite (pre-washed with MeOH). Flash chromatography (applied in CH_2_Cl_2_; eluted 100:1 to 50:1 to 8:1 CH_2_Cl_2_/EtOAc) was carried out to give the title compound as a yellow solid (181 mg, 0.355 mmol, 35%). Mp: >200 °C; *R_f_ *= 0.12 (10:1 CH_2_Cl_2_/EtOAc); IR (*ν*_max_/cm^−1^, thin film): 3240, 3057, 1623, 1500, 1330, 1154; ^1^H NMR (600 MHz, CDCl_3_): *δ*_H_ = 2.37 (s, 3H), 7.08–7.10 (m, 2H), 7.17 (s, 1H), 7.21 (d, *J* = 8.3 Hz, 2H), 7.52 (d, *J* = 4.7 Hz, 1H), 7.57–7.58 (m, 2H), 7.64 (dd, *J* = 8.5, 1.6 Hz, 1H), 7.65 (d, *J* = 8.3 Hz, 2H), 7.73 (s, 1H), 7.79–7.80 (m, 3H), 7.91–7.92 (m, 2H), 8.00 (d, *J* = 8.5 Hz, 1H), 8.03 (s, 1H), 8.39 (br s, 1H); ^13^C NMR (150 MHz, CDCl_3_): *δ*_C_ = 21.6, 109.4, 120.3, 123.7, 125.2, 125.4, 127.1, 127.3, 127.9, 128.1, 128.9, 129.0, 129.3, 129.7, 130.4, 131.2, 133.1, 133.4, 136.1, 137.2, 143.7, 146.2; LRMS *m*/*z* (ESI^−^): 504 [M−H]^−^; HRMS *m*/*z* (ESI^−^): Found 504.1503 [M−H]^−^; C_29_H_22_N_5_O_2_S requires 504.1494.

### 4.11 General method for synthesis of 2-aryl-8-chloro-imidazo[1,2-*a*]pyrazines, illustrated for the preparation of 8-chloro-2-(2-naphthyl)imidazo[1,2-*a*]pyrazine **21a**

2-(Bromoacetyl)naphthalene **15a** (3.14 g, 12.6 mmol), 2-amino-3-chloropyrazine (1.63 g, 12.6 mmol), NaHCO_3_ (1.32 g, 16.7 mmol) and *^t^*BuOH (60 mL) were stirred under reflux for 40 h. The reaction mixture was cooled to room temperature and the solvent removed in vacuo. The resulting orange solid was taken up in H_2_O (100 mL) and extracted with CH_2_Cl_2_ (3 × 150 mL). The combined organic layers were washed with H_2_O (75 mL) and brine (75 mL), dried (MgSO_4_), filtered and concentrated in vacuo to give crude orange solid. On addition of CH_2_Cl_2_ and MeOH (∼1:1), insoluble material filtered off to give the title compound as a cream fluffy solid (1.05 g). Purification of the remaining filtrate via flash chromatography (applied in petroleum ether; eluted 10% to 20% to 33% EtOAc) afforded the title compound as a pale orange/brown solid (1.38 g, 4.95 mmol, 39%). Mp: Decomposed before melting; *R_f_ *= 0.34 (1:1 petroleum ether/EtOAc); IR (*ν*_max_/cm^−1^, thin film): 3026, 2921, 1495; ^1^H NMR (600 MHz, (CD_3_)_2_SO): *δ*_H_ = 7.55–7.58 (m, 2H), 7.76 (d, *J* = 4.4 Hz, 1H), 7.95–7.97 (m, 1H), 8.04 (d, *J* = 8.6 Hz, 1H) 8.07–8.09 (m, 1H), 8.16 (dd, *J* = 8.6, 1.7 Hz, 1H), 8.64 (s, 1H), 8.67 (d, *J* = 4.4 Hz, 1H), 8.87 (s, 1H,); ^13^C NMR (150 MHz, CDCl_3_): *δ*_C_ = 113.7, 120.6, 124.1, 124.9, 126.6, 126.7, 127.7 (2 signals), 128.4, 128.6, 129.9, 133.1, 133.2, 137.5, 141.2, 146.4; LRMS *m*/*z* (ESI^+^): 282 [M(^37^Cl)+H]^+^, 280 [M(^35^Cl)+H]^+^; HRMS *m*/*z* (EI^+^): Found 279.05574 [M(^35^Cl)]^+^; C_16_H_10_N_3_Cl requires 279.05578.

### 4.12 General method for the synthesis of 4-methyl-*N*-[4-[2-arylimidazo[1,2-*a*]pyrazine-8-yl]]-sulfonamides, illustrated for the preparation of 4-methyl-*N*-[4-[2-(2-naphthyl)imidazo[1,2-*a*]pyrazine-8-yl]aminophenyl]benzenesulfonamide **11**

All glassware was evacuated and flushed with argon prior to use. **21a** (50.0 mg, 0.178 mmol), *N*-(4-aminophenyl)-4-methylbenzenesulfonamide **23** (56.3 mg, 0.215 mmol), NaO*^t^*Bu (24.1 mg, 0.250 mmol, 1.4 equiv), 1 mol % Pd_2_(dba)_3_ (1.6 mg) and 3 mol % DavePhos (2.1 mg) were weighed into a 25 mL round bottom flask. Toluene (2 mL) was added and the reaction was stirred under reflux for 24 h. The reaction mixture was cooled to room temperature and the solvents removed in vacuo. The residue was taken up in CH_2_Cl_2_ (30 mL) and washed with water (3 × 30 mL). The combined aqueous extracts were washed with CH_2_Cl_2_ (30 mL). The CH_2_Cl_2_ layers were combined, washed with brine (30 mL), dried over MgSO_4_ and the solvents removed in vacuo to give an off-white solid. Flash chromatography (applied in toluene; eluted 3:1 toluene/EtOAc) gave the title compound as an off-white solid (20 mg, 0.039 mmol, 22%). Mp: decomposed before melting; *R_f_ *= 0.50 (1:1 petroleum ether/EtOAc); IR (*ν*_max_/cm^−1^, thin film): 3126, 2923, 2853, 1507, 1325, 1143; ^1^H NMR (600 MHz, CD_3_OD): *δ*_H_ = 2.42 (s, 3H), 7.20 (d, *J* = 5.2 Hz, 1H), 7.29 (d, *J* = 8.9 Hz, 2H), 7.36 (d, *J* = 8.2 Hz, 2H), 7.52–7.56 (m, 2H), 7.58 (d, *J* = 8.9 Hz, 2H), 7.75 (d, *J* = 8.2 Hz, 2H), 7.92 (d, *J* = 7.1, 1H), 7.96–7.99 (m, 3H), 8.13 (dd, *J* = 8.5, 1.7 Hz, 1H), 8.53 (s, 1H), 8.56 (s, 1H); ^13^C NMR (150 MHz, CD_3_OD): *δ*_C_ = 21.4, 113.9, 115.9 (2 signals), 122.9, 124.9, 125.8, 126.1, 127.6, 127.7, 128.4, 128.9, 129.3, 129.7, 130.7, 131.0, 133.5 (2 signals) 135.0 (2 signals), 137.7, 138.2, 145.2, 146.1, 148.1; LRMS *m*/*z* (ESI+): 506 [M+H]^+^, (ESI^−^): 504 [M−H]^−^; HRMS *m*/*z* (ESI^+^): Found 506.1651 [M+H]^+^; C_29_H_24_N_5_O_2_S requires 506.1651.

### 4.13 8-(Methylsulfonyl)-2-(naphthalen-2-yl)imidazo[1,2-*a*]pyrazine **24**

Compound **21a** (1.12 g, 4.01 mmol) was dissolved in anhydrous DMSO (16 mL). NaSMe (337 mg, 4.81 mmol) was added portionwise and the reaction was stirred at 100 °C for 16 h. The mixture was then cooled to room temperature, diluted with brine (50 mL) and extracted with CH_2_Cl_2_ (100 mL). The organic layer was washed with H_2_O (5 × 30 mL) and brine (30 mL), dried (MgSO_4_), filtered and solvent removed in vacuo. Flash chromatography (applied in CH_2_Cl_2_; eluted 0% to 1% to 2% EtOAc) afforded 8-(methylthio)-2-(naphthalen-2-yl)imidazo[1,2-*a*]pyrazine as an off white/yellow solid (989 mg, 3.40 mmol, 85%). Mp: 168 °C; *R_f_ *= 0.47 (5% EtOAc/CH_2_Cl_2_); IR (*ν*_max_/cm^−1^, thin film): 3055; ^1^H NMR (600 MHz, CDCl_3_): *δ*_H_ = 2.71 (s, 3H), 7.47–7.52 (m, 2H), 7.72 (d, *J* = 4.5 Hz, 1H), 7.82 (d, *J* = 4.5 Hz, 1H), 7.85 (d, *J* = 7.4 Hz, 1H), 7.90 (d, *J* = 8.5 Hz, 1H), 7.94 (d, *J* = 7.4 Hz, 1H), 7.98 (s, 1H), 8.04 (dd, *J* = 8.5, 1.6 Hz, 1H), 8.55 (s, 1H); ^13^C NMR (150 MHz, CDCl_3_): *δ*_C_ = 12.3, 110.3, 115.1, 124.3, 125.6, 126.4, 126.5, 127.9, 128.5, 128.6, 129.0, 130.2, 133.5 (2 signals), 138.9, 146.4, 154.4; LRMS *m*/*z* (ES^+^): 292 [M+H]^+^; HRMS *m*/*z* (ES^+^): Found 292.0909 [M+H]^+^; C_17_H_14_N_3_S requires 292.0908.

8-(Methylthio)-2-(naphthalen-2-yl)imidazo[1,2-*a*]pyrazine (1.77 g, 6.07 mmol) was dissolved in anhydrous CH_2_Cl_2_ (50 mL) and the mixture was cooled on ice. *m*CPBA (5.23 g, 30.3 mmol) was added in one portion and the reaction continued to stir at room temperature for 5 h. The reaction was partitioned with NaHCO_3_ (40 mL) and extracted with CH_2_Cl_2_ (3 × 30 mL); the combined organic extracts were then washed with brine (30 mL), dried (MgSO_4_), filtered and concentrated in vacuo. Flash chromatography (applied in CH_2_Cl_2_; eluted 2–5% EtOAc) afforded the title compound as a yellow solid (1.10 g, 3.40 mmol, 56.0%). Mp: >200 °C; *R_f_ *= 0.22 (10% EtOAc/CH_2_Cl_2_); IR (*ν*_max_/cm^−1^, thin film): 3121, 3010, 1312, 1138; ^1^H NMR (600 MHz, CDCl_3_): *δ*_H_ = 3.84 (s, 3H), 7.51–7.54 (m, 2H), 7.86–7.87 (m, 1H), 7.92 (d, *J* = 8.5 Hz, 1H), 7.95–7.96 (m, 1H), 8.02 (d, *J* = 4.3 Hz, 1H), 8.08 (dd, *J* = 8.5, 1.4 Hz, 1H), 8.26 (s, 1H), 8.32 (d, *J* = 4.3 Hz, 1H), 8.59 (s, 1H); ^13^C NMR (150 MHz, CDCl_3_): *δ*_C_ = 41.7, 110.9, 122.1, 124.3, 126.7, 126.8, 127.0, 127.9, 128.1, 128.7, 128.8, 129.1, 133.5, 134.0, 136.3, 148.6, 149.8; LRMS *m*/*z* (EI^+^): 323 [M]^+^; HRMS *m*/*z* (EI^+^): Found 323.07266 [M]^+^; C_17_H_13_N_3_O_2_S requires 323.07230.

### 4.14 Conversion of **24** to 4-methyl-*N*-[2-(2-naphthyl)imidazo[1,2-*a*]pyrazine-8-yl]benzenesulfonamide **7**

NaH (7.1 mg of a 60% suspension in mineral oil, 4.6 mg, 0.186 mmol, 2.0 equiv) was washed by stirring in anhydrous hexane (3 mL), syringing out the solvent and drying. Anhydrous DMF (0.5 mL) was added to the flask under Ar, followed by 4-toluene sulfonamide **22** (31.8 mg, 0.186 mmol, 2.0 equiv) in anhydrous DMF (0.5 mL). The contents were then stirred at rt for 20 min before **24** (30 mg, 0.093 mmol) in DMF (2 mL) was added dropwise, and the reaction was stirred at 100 °C for 20 h. The reaction was cooled to room temperature and quenched with satd aq NH_4_Cl (30 mL) and extracted with ethyl acetate (3 × 30 mL). The combined organic layers were washed with H_2_O (5 × 30 mL) and brine, dried (MgSO_4_), filtered and solvent removed. Flash chromatography (applied in petroleum ether; gradient 20% to 66% EtOAc) afforded **7** (27.8 mg, 0.669 mmol, 72%). Mp: >200 °C; *R_f_* = 0.32 (1:1 CH_2_Cl_2_/EtOAc); IR (*ν*_max_/cm^−1^, thin film): 3253; ^1^H NMR (600 MHz, DMSO-*d*_6_): *δ*_H_ = 2.37 (s, 3H), 7.16 (br d, *J* = 5.2 Hz, 1H), 7.39 (d, *J* = 8.2 Hz, 2H), 7.51–7.54 (m, 2H), 7.86 (br d, *J* = 5.2 Hz, 1H), 7.89 (d, *J* = 8.0 Hz, 2H), 7.92 (d, *J* = 7.7 Hz, 1H), 7.98 (d, *J* = 8.6 Hz, 1H), 8.01–8.05 (m, 2H), 8.52 (s, 1H), 8.59 (s, 1H), 11.69 (s, 1H, NH); ^13^C NMR (150 MHz, DMSO-*d*_6_): *δ*_C_ = 21.0, 111.0, 115.3, 116.8, 123.8, 124.2, 126.2, 126.3, 126.6, 127.7, 128.3, 128.4, 129.5, 130.0, 132.8, 133.2, 135.6, 140.0, 142.7, 144.5, 145.3; LRMS *m*/*z* (ESI^+^): 415 [M+H]^+^, (ESI^−^): 413 [M−H]^−^, HRMS *m*/*z* (ESI^+^): Found 415.1219 [M+H]^+^; C_23_H_19_N_4_O_2_S requires 415.1229.

### 4.15 Enzyme activity measurements

Assays for the activity of the HP0525 inhibitors were performed using a colorimetric ATPase assay (Innova Biosciences), see SI for details. The enzymatic reactions were performed in 96-well format for 30 min at 37 °C followed by measuring the absorbance at 620 nm, detecting the presence of inorganic phosphate product. For the IC_50_ measurements, each reaction contained 100 mM Tris–HCl (pH 7.5), 2.5 mM MgCl_2_, 125 μM ATP, 5% DMSO, 25 mM NaCl, 0.5 mM DTT 0.053 μM HP0525 and various concentrations of inhibitors (0–50 μM or 0–250 μM). Michaelis–Menten kinetics were performed under the same conditions as above but with various concentrations of ATP ranging from 0 to 500 μM, with and without 10 μM of compound **11**. Both IC_50_ and Michaelis–Menten kinetics measurements were made in triplicate.

## Supplementary data

Supplementary data Experimental details for the synthesis of compounds **1**–**4**, **6**–**10**, **12**, **13**, **25**–**38**, details of the optimization of the conversion of **21a** to **7**, NMR data confirming the regiochemistry of **21a**, ^1^H NMR spectra for all novel compounds, full description of the ATPase assay, IC_50_ curves for all compounds.
