# Supplementary material for: 2- and 3-substituted imidazo[1,2-a]pyrazines as inhibitors of bacterial type IV secretion
Source: Bioorg Med Chem. 2014 Nov 15;22(22):6459–70. doi: 10.1016/j.bmc.2014.09.036 (PMC4339681; doi:10.1016/j.bmc.2014.09.036)
Supplement: Supplementary data — Experimental details for the synthesis of compounds 1–4, 6–10, 12, 13, 25–38, details of the optimization of the conversion of 21a to 7, NMR data confirming the regiochemistry of 21a, 1H NMR spectra for all novel compounds, full description of the ATPase assay, IC50 curves for all compounds. [file mmc1.doc]

**2- And 3-substituted imidazo[1,2-*a*]pyrazines as inhibitors of bacterial type IV secretion**

James R. Sayer, Karin Walldén, Thomas Pesnot, Frederick Campbell, Paul J. Gane, Michela Simone, Hans Koss, Floris Buelens, Timothy P. Boyle, David L. Selwood, Gabriel Waksman, Alethea B. Tabor

***Supporting Information***

|  | Page |
| --- | --- |
| General Chemistry Methods | 2 |
| First generation imidazo[1,2-*a*]pyrazine inhibitors |  |
| Synthesis of α-azido aryl ketones **16b-e** | 3 |
| Synthesis of α-azido aryl alcohols **17b-e** | 6 |
| Synthesis of α-amino aryl alcohols **14b-e** | 9 |
| Synthesis of 2-[(3-chloropyrazin-2-yl)amino]-1-(2-aryl)ethanols **18b-e** | 12 |
| Synthesis of 2-[(3-chloropyrazin-2-yl)amino]-1-(2-aryl)ethanones **19b-e** | 15 |
| Synthesis of 3-aryl-8-chloro-imidazo[1,2-*a*]pyrazines **20b-e** | 18 |
| Synthesis of 2-aryl-8-chloro-imidazo[1,2-*a*]pyrazines **21b-e** | 22 |
| Synthesis of first generation 2- and 3-substituted 8-aminoimidazo[1,2-*a*]pyrazines  **1 – 4, 6 - 13** | 25 |
| Optimisation of reaction conditions for the synthesis of **7** | 37 |
| Second generation imidazo[1,2-*a*]pyrazine inhibitors, variants at 8-position, **25 – 38** | 41 |
| **Figure S1** HMBC spectrum of **21a** | 61 |
| Molecular docking – additional information | 61 |
| ^1^H NMR spectra of novel compounds | 62 |
| References | 121 |
| Details of ATPase assay | 123 |
| **Figure S1** IC50 curves | 125 |

**General Chemistry Methods**

Melting points (Mpt) were recorded on a Gallenkamp Melting Point Apparatus and are uncorrected. Proton nuclear magnetic resonance spectra (^1^H NMR) were recorded using Bruker AV400 (400 MHz), AV500 (500 MHz) and AV600 (600MHz) spectrometers as indicated. Carbon nuclear magnetic resonance spectra (^13^C NMR) were recorded using Bruker AV400 (100 MHz), AV500 (125 MHz) and AV600 (150MHz) spectrometers as indicated. Spectra were obtained using CDCl_3_, CD_3_OD, CD_2_Cl_2_ and DMSO-*d_6_* as solvents and chemical shifts are quoted on the δ scale in units of ppm using TMS as an internal standard. Coupling constants (*J*) are reported in Hz with the following splitting abbreviations: s (singlet), d (doublet), t (triplet), dd (doublet of doublets), bs (broad singlet). Infra-Red (IR) spectroscopy was carried out using a PerkinElmer Spectrum 100 FT-IR Spectrometer using thin films. Absorption maxima (ν_max_) are reported in wavenumbers (cm^-1^). High Performance Liquid Chromatography (HPLC) was performed using a Varian ProStar instrument; A Chiralpak AD column for normal phase analytical HPLC; DiscoveryBIO wide pore C18-10 (25 cm x 4.6 mm, 10 µm) for reverse phase analytical HPLC; and a DiscoveryBIO wide pore C18 (25 cm x 21.2 mm, 10 µm) column for reverse phase preparative HPLC. Each solvent used contained 0.1% TFA buffer. Liquid Chromatography Mass Spectrometry (LCMS) was carried out using SQD-Waters Acquity UPLC/SQD with C18 (50 mm x 2.1 mm, 1.7 µm) column. A total run time of 5 minutes and flow rate of 0.6 mL/min was used with gradient elution: 95% H_2_O/ 5% MeCN (0 min), 5% H_2_O/ 95% MeCN (3 min), 95% H_2_O/ 5% MeCN (4.5 min). Each solvent contained 0.1% formic acid buffer. LRMS refers to low resolution mass spectrometry and HRMS refers to high resolution mass spectrometry. Electron Impact/ Chemical Ionisation (EI/CI) MS was carried out using MAT900XP (Thermo Finnigan) instrument and electrospray ionization (ESI) accurate mass was determined using Waters LCT Premier XE instrumentation. Thin layer chromatography (TLC) was carried out using Fluka aluminium backed sheets coated with 60F_254_ silica gel. Visualisation of the silica plates was achieved using a UV lamp (λ_max_ = 245 nm) and/or potassium permanganate (KMnO_4_ in 1M NaOH with 5% K_2_CO_3_). Flash chromatography was carried out using either Geduran (Merck) or ZEOprep (Apollo) Si60 40-63 µm silica gel.HHjksdj

Solvents and reagents were obtained from commercial sources and were used as received unless otherwise stated. Dry solvents were dried over anhydrous columns,^1^ moisture levels were usually <15 ppm by Karl Fischer titration. Pet. ether refers to the fraction of light petroleum ether boiling in the range 40-60 ºC.

**First generation imidazo[1,2-*a*]pyrazine inhibitors**

**Synthesis of α-azido aryl ketones**

**2-azido-1-(2-phenoxyphenyl)ethanone, 16b**

2-bromo-1-(2-phenoxyphenyl)ethanone **15b** (3.07 g, 10.5 mmol) was dissolved in DMSO (15 mL) and the mixture was cooled on ice. Sodium azide (824 mg, 12.68 mmol) was added in one portion and the reaction was stirred under argon at RT for 5 h. An extra portion of sodium azide (200 mg) was added and left to stir overnight. After 15 h, the reaction was quenched with H_2_O (30 mL), and extracted with EtOAc (4 x 40 mL). The combined organic layers were washed with H_2_O (5 x 20 mL) and then dried (Na_2_SO_4_) and filtered. The solvent was removed *in vacuo* to give the product as a brown liquid (2.52 g, 9.96 mmol, 95%). R*_f_* = 0.4 (CH_2_Cl_2_); IR (ν_max_/cm^-1^, thin film): 3071, 2100, 1685, 1599; ^1^H NMR (500 MHz, CDCl_3_): δ_H_ = 4.60 (s, 2H, **1-H**), 6.87 (dd, *J* = 8.3, 0.9 Hz, 1H, **7-H**), 7.04-7.06 (m, 2H, **10,14-H**), 7.18-7.23 (m 2H, **5,12-H**), 7.40-7.43 (m, 2H, **11,13-H**), 7.45-7.49 (m, 1H, **6-H**), 7.92 (dd, *J* = 7.8, 1.5 Hz, 1H, **4-H**); ^13^C NMR (125 MHz, CDCl_3_): δ_C_ = 58.9 (**C-1**), 118.0 (**C-7**), 119.2 (**C-10**), 123.2 (**C-5**), 124.4 (**C-12**), 126.2 (**C-3**), 130.0 (**C-11**), 130.8 (**C-4**), 134.5 (**C-6**), 155.0 (**C-9**), 157.0 (**C-8**), 193.6 (**C-2**); LRMS m/z (CI^+^): 198 [M-CH_2_N­_3_]^+^, 85 [COCH_2_N_3_]^+^.

**2-azido-1-(3,4-dimethoxyphenyl)ethanone, 16c**

2-Bromo-1-(3,4-dimethoxyphenyl)ethanone^2^ (2.33 g, 8.61 mmol) was dissolved in anhydrous DMSO (10 mL) and the mixture was cooled on ice. Sodium azide (671 mg, 10.3 mmol) was added in one portion and the reaction was stirred under argon at RT for 2 h. An immediate colour change of yellow to orange was observed on addition of the sodium azide. The reaction was then quenched with H_2_O (30 mL), and extracted with EtOAc (4 x 40 mL). The combined organic layers were washed with H_2_O (5 x 20 mL) followed by brine (20 mL) and then dried (MgSO_4_) and filtered. The solvent was removed *in vacuo* to give an orange solid (1.87 g, 8.46 mmol, 98%) with NMR data comparable with literature values.^3^ R*_f_* = 0.22 (CH_2_Cl_2_); IR (ν_max_/cm^-1^, thin film): 2106, 1682, 1595, 1515; ^1^H NMR (600 MHz, CDCl_3_): δ_H_ = 3.94 (s, 3H, **9-H**), 3.96 (s, 3H, **10-H**), 4.52 (s, 2H, **1-H**), 6.90 (d, *J* = 8.4 Hz, 1H, **5-H**), 7.47 (dd, *J* = 8.5, 2.0 Hz, 1H, **4-H**), 7.52 (d, *J* = 2.1 Hz, 1H, **8-H**); ^13^C NMR (150 MHz, CDCl_3_): δ_C_ = 54.5 (**C-1**), 56.1 (**C-9**), 56.2 (**C-10**), 110.0 (**C-8**), 110.1 (**C-5**), 122.5 (**C-4**), 127.5 (**C-3**), 149.4 (**C-7**), 154.1 (**C-6**), 191.8 (**C-2**); LRMS m/z (CI^+^): 222 [M+H]^+^, 165 [M-CH_2_N_3_]^+^; HRMS m/z (CI^+^): Found 222.0878 [M+H]^+^; C_10_H_12_N_3_O_3_ requires 222.0879.

**2-azido-1-(3,5-dimethylphenyl)ethanone, 16d**

2-Bromo-1-(3,5-dimethylphenyl)ethanone^4^ (3.44 g, 15.1 mmol) was dissolved in anhydrous DMSO (15 mL) and the mixture was cooled on ice. Sodium azide (1.18 g, 18.2 mmol) was added in one portion and the reaction was stirred under argon at RT for 16 h. An extra portion of sodium azide (200 mg) was added and the reaction was left to stir for a further 1 h. A colour change from yellow to deep orange was observed. The reaction was then quenched with H_2_O (30 mL), and extracted with EtOAc (4 x 40 mL). The combined organic layers were washed with H_2_O (5 x 20 mL) followed by brine (20 mL) and then dried (MgSO_4_) and filtered. The solvent was removed *in vacuo* to give an orange solid (2.85 g, 15.1 mmol, 100%). R*_f_* = 0.59 (CH_2_Cl_2_); IR (ν_max_/cm^-1^, thin film): 2920, 2105, 1692, 1604; ^1^H NMR (500 MHz, CDCl_3_): δ_H_ = 2.35 (s, 6H, **7-H**), 4.52 (s, 2H, **1-H**), 7.20 (s, 1H, **6-H**), 7.40 (s, 2H, **4-H**); ^13^C NMR (125 MHz, CDCl_3_): δ_C_ = 21.3 (**C-7**), 55.0 (**C-1**), 125.7 (**C-4**), 134.5 (**C-3**), 135.9 (**C-6**), 138.8 (**C-5**), 193.6 (**C-2**); LRMS m/z (CI^+^): 132 [M-CH_2_N_3_]^+^

**2-azido-1-(3-thienyl)ethanone, 16e**

2-bromo-1-(3-thienyl)-1-ethanone^5^ **15e** (573 mg, 2.80 mmol) was dissolved in DMSO (3 mL) and the mixture was cooled on ice. Sodium azide (218 mg, 3.35 mmol) was added in one portion and the reaction was stirred under argon at RT for 5 h. The reaction was quenched with H_2_O (20 mL), and extracted with EtOAc (3 x 30 mL). The organic layers were combined, washed with H_2_O (5 x 20 mL) followed by brine (20 mL), dried (Na_2_SO_4_) and filtered. The solvent was removed *in vacuo* to give the title compound as a brown/orange oil (460 mg, 2.75 mmol, 98%). R*_f_* = 0.27 (CH_2_Cl_2_); IR (ν_max_/cm^-1^, thin film): 3105, 2097, 1679, 1508; ^1^H NMR (500 MHz, CDCl_3_): δ_H_ = 4.43 (s, 2H, **1-H**), 7.38 (dd, *J* = 5.1, 2.8 Hz, 1H, **5-H**), 7.55 (dd, *J* = 5.0, 1.2 Hz, 1H, **4-H**), 8.10 (dd, *J* = 2.9, 1.3 Hz, 1H, **7-H**); ^13^C NMR (125 MHz, CDCl_3_): δ_C_ = 55.5 (**C-1**), 126.6 (**C-5**), 127.2 (**C-4**), 132.7 (**C-7**), 139.1 (**C-3**), 187.6 (**C-2**); LRMS m/z (ES+): 185 [M+18]+, 168 [M+H]+; HRMS m/z (ESI^+^): Found 168.02354 [M+H]^+^; C_6_H_6_N_3_OS requires 168.02316.

**Synthesis of α-azido aryl alcohols**

**2-azido-1-(2-phenoxyphenyl)ethanol, 17b**

**16b** (2.52 g, 9.96 mmol) was dissolved in anhydrous MeOH (50 mL) and cooled on ice. Sodium borohydride (565 mg, 14.9 mmol) was added portion wise and the mixture was stirred on ice under argon for 1 h. The solvent was removed and the resulting residue was taken up in CH_2_Cl_2_ (50 mL) and carefully washed with H_2_O (2 x 40 mL). Re-extraction of the aqueous layers with CH_2_Cl_2_ (3 x 30 mL), followed by washing the combined organic extracts with brine, drying (Na_2_SO_4_), filtering and concentrating *in vacuo* gave the title compound as a brown oil (2.47 g, 9.69 mmol, 97%). R*_f_* = 0.19 (CH_2_Cl_2_); IR (ν_max_/cm^-1^, thin film): 3413, 3039, 2102, 1583; ^1^H NMR (500 MHz, CDCl_3_): δ_H_ = 2.54 (bs, 1H, 15-H), 3.50 (dd, *J* = 12.5, 8.0 Hz, 1H, **1-H**), 3.58 (dd, *J* = 12.5, 3.5 Hz, 1H, **1-H**), 5.23 (dd, *J* = 8.0, 3.4 Hz, 1H, **2-H**), 6.84 (dd, *J* = 8.2, 1.1 Hz, 1H, **7-H**), 6.97-7.00 (m, 2H, **10-H**), 7.13 (tt, *J* = 7.4, 1.1 Hz, 1H, **5-H***)*, 7.17 (td, *J* = 7.5, 1.0 Hz, 1H, **12-H**), 7.24-7.27 (m, 1H, **6-H**), 7.34-7.37 (m, 2H, **11-H**), 7.58 (dd, *J* = 7.7, 1.7 Hz, 1H, **4-H**); ^13^C NMR (125 MHz, CDCl_3_): δ_C_ = 56.5 (**C-1**), 68.7 (**C-2**), 118.0 (**C-7**), 118.3 (**C-10**), 123.4 (**C-5**), 123.5 (**C-12**), 127.1 (**C-4**), 129.6 (**C-6**), 129.6 (**C-11**), 130.8 (**C-3**), 153.5 (**C-9**), 156.3 (**C-8**); LRMS m/z (CI^+^): 200 [M-CH_2_N­_3_]^+^, 182 [M-OH,CH_2_N_3_]^+^.

**2-azido-1-(3,4-dimethoxyphenyl)ethanol, 17c**

**16c** (1.86 g, 8.40 mmol) was dissolved in anhydrous Et_2_O (80 mL). Activated neutral alumina (8 g) and sodium borohydride (635 mg, 16.8 mmol) were added and the suspension was stirred at RT under argon. After 16 h the reaction mixture was filtered and washed with ether. The resulting filtrate was washed with H_2_O (2 x 30 mL), followed by brine (2 x 30 mL). The combined organic extracts were dried (Na_2_SO_4_), filtered and concentrated *in vacuo* to give the title compound as a yellow oil (1.64 g, 7.37 mmol, 88%). R*_f_* = 0.65 (2:1 CH_2_Cl_2_/EtOAc); IR (ν_max_/cm^-1^, thin film): 3497, 2938, 2839, 2106, 1516; ^1^H NMR (500 MHz, CDCl_3_): δ_H_ = 2.67 (bs, 1H, **11-H**) 3.36 (dd, *J* = 12.6, 3.8 Hz, 1H, **1-H**), 3.49 (dd, *J* = 12.6, 8.3 Hz, 1H, **1-H**), 3.84 (s, 3H, **9-H**), 3.85 (s, 3H, **10-H**), 4.78 (dd, *J* = 8.3, 3.8 Hz, 1H, **2-H**), 6.82 (d, *J* = 8.2 Hz, 1H, **5-H**), 6.86 (dd, *J* = 8.2 ,1.9 Hz, 1H, **4-H**), 6.89 (d, *J* = 1.9 Hz, 1H, **8-H**); ^13^C NMR (125 MHz, CDCl_3_): δ_C_ = 55.6 (**C-9,10**), 57.7 (**C-1**), 72.9 (**C-2**), 108.6 (**C-8**), 110.8 (**C-5**), 117.9 (**C-4**), 132.0 (**C-3**), 148.6 (**C-7**), 148.8 (**C-6**); LRMS m/z (EI^+^): 223 [M]^+^,167 [M-CH_2_N_3_]^+^, 139 [M-CH(OH)CH_2_N_3_]^+^.

**2-azido-1-(3,5-dimethylphenyl)ethanol, 17d**

**16d** (2.85 g, 15.1 mmol) was dissolved in anhydrous MeOH (60 mL) and cooled on ice. Sodium borohydride (855 mg, 22.6 mmol) was added portion wise and the mixture was stirred on ice under argon for 1 h. The solvent was removed and the resulting orange oil was taken up in CH_2_Cl_2_ (60 mL) and washed with 2.0 M HCl (40 mL), H_2_O (30 mL) and brine (30 mL). The combined organic extracts were dried (MgSO_4_), filtered and concentrated *in vacuo* to give the title compound as an orange oil (2.85 g, 14.9 mmol, 99%). R*_f_* = 0.27 (CH_2_Cl_2_); IR (ν_max_/cm^-1^, thin film): 3395, 2918, 2099; ^1^H NMR (500 MHz, CDCl_3_): δ_H_ = 2.32 (s, 6H, **7-H**), 3.40 (dd, *J* = 12.6, 3.8 Hz, 1H, **1-H**), 3.47 (dd, *J =* 12.6, 8.4 Hz, 1H, **1-H**), 4.79 (dd, *J* = 8.4, 3.8 Hz, 1H, **2-H**), 6.96 (s, 1H, **6-H**), 6.97 (s, 2H, **4-H**); ^13^C NMR (125 MHz, CDCl_3_): δ_C_ = 21.2 (**C-7**), 58.2 (**C-1**), 73.5 (**C-2**), 123.8 (**C-4**), 130.0 (**C-6**), 138.4 (**C-5**), 140.7 (**C-3**); LRMS m/z (CI^+^): 192 [M+H]^+^, 132 [M-OH-N_3_]^+^.

**2-azido-1-(3-thienyl)ethanol, 17e**

**16e** (278 mg, 1.67 mmol) was dissolved in anhydrous MeOH (10 mL) and cooled on ice. Sodium borohydride (94.5 mg, 2.45 mmol) was added portion wise and the mixture was stirred on ice under argon for 1 h. The solvent was removed and the resulting residue was taken up in CH_2_Cl_2_ (30 mL) and carefully washed with H_2_O (2 x 20 mL) followed by brine (20 mL). The organic extracts were dried (Na_2_SO_4_), filtered and concentrated *in vacuo* to give the title compound as a yellow oil (257 mg, 1.52 mmol 91%). R*_f_* = 0.8 (1:1 pet. ether /EtOAc); IR (ν_max_/cm^-1^, thin film): 3372, 3105, 2096; ^1^H NMR (500 MHz, CDCl_3_): δ_H_ = 3.51-3.54 (m, 2H, **1-H**), 4.98 (dd, *J* = 4.7,1.9 Hz, 1H, **2-H**), 7.08 (dd, *J* = 5.0,1.3 Hz, 1H, **4-H**), 7.29-7.30 (m,, 1H, **7-H**), 7.34 (dd, *J* = 5.0, 3.0 Hz, 1H, **5-H**); ^13^C NMR (125 MHz, CDCl_3_): δ_C_ = 57.5 (**C-1**), 69.9 (**C-2**) 122.0 (**C-7**), 125.4 (**C-4**), 126.7 (**C-5**), 142.0 (**C-3**); LRMS m/z (CI^+^): 152 [M-OH]^+^, 127 [M-N_3_]^+^, 113 [M-CH_2_N_3_]^+^; HRMS m/z (CI^+^): Found 152.02859 [M-OH]^+^; C_6_H_6_N_3_S requires 152.02824.

**Synthesis of α-amino aryl alcohols**

**2-amino-1-(2-phenoxyphenyl)ethanol, 14b**

**17b** (2.46 g, 9.66 mmol) was dissolved in anhydrous MeOH (50 mL) and 10% Pd/C (246 mg,10% wt/wt) was added. The vessel was evacuated and purged with Ar (3x), and under static vacuum a balloon of H_2_ was added. The reaction was stirred at RT under H_2_ atmosphere until completion, as determined by TLC and disappearance of N_3_ peak by IR. After 22 h, the H_2_ was carefully released, the vessel evacuated and purged Ar (3x), and the reaction mixture was filtered through Celite (pre-washed with MeOH). Solvent removal *in vacuo* gave the title compound as a brown oil (2.21 g, 9.66 mmol, 100%). *Rf* = 0.0 (CH_2_Cl_2_); IR (ν_max_/cm-1, thin film): 3413, 3055, 2983; 1H NMR (600 MHz, CDCl_3_): δ_H_ = 2.17 (bs, 3H, **15,16-H**), 2.84 (dd, *J* = 13.2, 7.8 Hz, 1H, **1-H**), 3.10 (dd, *J* = 13.2, 4.2 Hz, 1H, **1-H**), 4.99 (dd, *J* = 7.8, 3.6 Hz, 1H, **2-H**), 6.82 (dd, *J* = 8.4, 1.2 Hz, 1H, **7-H**), 6.95 (d, *J* = 7.8 Hz, 2H, **10-H**), 7.08- 7.11 (m, 1H, **12-H***)*, 7.13-7.15 (m, 1H, **5-H**), 7.21 (td, *J* = 7.8, 1.8 Hz, 1H, **6-H**), 7.30-7.33 (m, 2H, **11-H**), 7.57 (dd, *J* = 7.8, 1.2 Hz, 1H, **4-H**); 13C NMR (150 MHz, CDCl_3_): δ_C_ = 47.7 (**C-1**), 69.4 (**C-2**), 118.2 (**C-7**), 118.5 (**C-10**), 123.4 (**C-12**), 123.9 (**C-5**), 127.5 (**C-4**), 128.6 (**C-6**), 130.0 (**C-11**), 133.3 (**C-3**), 153.8 (**C-9**), 157.2 (**C-8**); LRMS m/z (ES+): 230.1 [M+H]+ 212.1 [M-OH]+, 195.1 [M-OH, NH_2_]^+^.

**2-amino-1-(3,4-dimethoxyphenyl)ethanol, 14c**

**17c** (1.58 g, 7.09 mmol) was dissolved in anhydrous MeOH (30 mL) and 10% palladium on carbon (158 mg, 10% w/w) was added. The vessel was evacuated and purged with Ar (3x), and under static vacuum a balloon of H_2_ was added. The reaction mixture was stirred under hydrogen atmosphere until completion as determined by TLC and disappearance of N­_3_ peak by IR. After 2 h, the hydrogen was carefully released, and the reaction mixture was filtered through Celite (pre-washed with MeOH). Solvent removal *in vacuo* gave the crude compound as an orange oil. Flash chromatography (100% EtOAc followed by 100% MeOH), followed by re-dissolving in CH_2_Cl_2_ and filtering to remove silica yielded the title compound as a white solid (880 mg, 4.47 mmol, 63%). Spectroscopic data was consistent with that previously reported.^6^ R*_f_* = 0.3 (1:1 EtOAc/MeOH); IR (ν_max_/cm^-1^, thin film): 3362, 2938, 2838; ^1^H NMR (500 MHz, CDCl_3_): δ_H_ = 2.14 (bs, 3H, **11,12-H**) 2.80 (dd, *J* = 12.7, 7.9 Hz, 1H, **1-H**), 2.96 (dd, *J* = 12.6, 4.0 Hz, 1H, **1-H**), 3.86 (s, 3H, **9-H**), 3.88 (s, 3H, **10-H**), 4.56 (dd, *J* = 7.9, 4.0 Hz, 1H, **2-H**), 6.82 (d, *J* = 8.2 Hz, 1H, **5-H**), 6.86 (dd, *J* = 8.5, 1.8 Hz, 1H, **4-H**), 6.91 (d, *J* = 1.8 Hz, 1H, **8-H**); ^13^C NMR (125 MHz, CDCl_3_): δ_C_ = 48.9 (**C-1**), 55.5 (**C-9**),55.6 (**C-10**) 73.8 (**C-2**), 108.7 (**C-8**), 110.7 (**C-5**), 117.8 (**C-4**), 134.8 (**C-3**), 148.1 (**C-7**), 148.7 (**C-6**); LRMS m/z (EI^+^): 197 [M]^+^, 167 [M-CH_2_NH_2_]^+^; HRMS m/z (EI^+^): Found: 197.1049 [M]^+^; C_10_H_15_NO_3_ requires 197.1046.

**2-amino-1-(3,5-dimethylphenyl)ethanol, 14d**

**17d** (2.85 g, 14.9 mmol) was dissolved in anhydrous MeOH (60 mL) and 10% palladium on carbon (285 mg, 10% w/w) was added. The vessel was evacuated and purged with Ar (3x), and under static vacuum a balloon of H_2_ was added. The reaction mixture was stirred under hydrogen atmosphere until completion as determined by TLC and disappearance of N­_3_ peak by IR. After 22 h, the hydrogen was carefully released, and the reaction mixture was filtered through Celite (pre-washed with MeOH). Solvent removal *in vacuo* gave the title compound as a sticky green/brown solid (2.37 g, 14.4 mmol, 97%). R*_f_* = 0.0 (CH_2_Cl_2_); IR (ν_max_/cm^-1^, thin film): 3289, 3010, 2916, 2861; ^1^H NMR (600 MHz, CDCl_3_): δ_H_ = 2.31 (s, 6H, **7-H**), 2.81 (dd, *J* = 12.6, 7.8 Hz, 1H, **1-H**), 2.96 (dd, *J =* 13.2, 4.2 Hz, 1H, **1-H**), 4.57 (dd, *J* = 7.8, 4.2 Hz, 1H, **2-H**), 6.91 (s, 1H, **6-H**), 6.96 (s, 2H, **4-H**); ^13^C NMR (150 MHz, CDCl_3_): δ_C_ = 21.5 (**C-7**), 49.3 (**C-1**), 74.5 (**C-2**), 123.8 (**C-4**), 130.6 (**C-6**), 138.1 (**C-5**), 142.5 (**C-3**); LRMS m/z (EI^+^): 224, 135 [M-CH_2_NH_2_]^+^, 117 [M-CH_2_OHNH_2_]^+^.

**2-amino-1-(3-thienyl)ethanol, 14e**

**14e** (257 mg, 1.52 mmol) was dissolved in anhydrous MeOH (10 mL) and 10% palladium on carbon (25.7 mg, 10% w/w) was added. The vessel was evacuated and purged with Ar (3x), and under static vacuum the reaction vessel was then subjected to 3 bar hydrogen for 5 h. After this period the mixture was filtered through Celite and concentrated *in vacuo* to give a pale yellow solid (217 mg, 1.52 mmol, 100%). R*_f_* = 0.0 (1:1 CH_2_Cl_2_/EtOAc); ν_max_/cm^-1^ (thin film): 3194, 3091, 2921; ^1^H NMR (600 MHz, CDCl_3_): δ_H_ = 1.91 (bs, 3H, **8,9-H**), 2.88 (dd, *J =* 12.6, 7.8 Hz, 1H, **1-H**), 3.04 (d, *J =* 10.2 Hz, 1H, **1-H**), 4.75 (dd, *J* = 6.6, 3.6 Hz, 1H, **2-H**), 7.08 (d, *J* = 5.4 Hz, 1H, **4-H**), 7.23 (d, *J* = 2.4 Hz, 1H, **7-H**), 7.31 (dd, *J* = 4.8, 3.0 Hz, 1H, **5-H**); ^13^C NMR (150 MHz, CDCl_3_): δ_C_ = 48.4 (**C-1**), 70.9 (**C-2**) 121.1 (**C-7**), 125.6 (**C-4**), 126.3 (**C-5**), 144.0 (**C-3**); LRMS m/z (CI^+^): 177, 159, 127 [M-NH_2_]^+^; (EI^+^): 131, 127 [M-NH_2_]^+^, 119, 114.

**Synthesis of 2-[(3-chloropyrazin-2-yl)amino]-1-(2-aryl)ethanols**

**2-[(3-chloropyrazin-2-yl)amino]-1-(2-phenoxyphenyl)ethanol, 18b**

**14b** (2.21 g, 9.66 mmol), 2,3-dichloropyrazine (1.11 mL, 10.6 mmol) and Et_3_N (1.88 mL, 13.5 mmol) were dissolved in 1,4-dioxane (22 mL) and the reaction was stirred under reflux, under argon. After 17 h, the reaction was cooled to RT, and the solvent removed *in vacuo*. The residue was taken up in CH_2_Cl_2_ and washed with H_2_O (3 x 20 mL) and brine (1 x 20 mL). The organic extracts were dried (Na_2_SO_4_), filtered and concentrated to give the crude product as a brown oil. Purification was carried out *via* flash chromatography (applied in CH_2_Cl_2_; eluted 0% to 10% EtOAc) to afford the title compound as an orange oil (2.09 g, 6.15 mmol, 64%). R*_f_* = 0.42 (9:1 CH_2_Cl_2_/EtOAc); IR (ν_max_/cm^-1^, thin film): 3423, 3060, 2924; ^1^H NMR (400 MHz, CDCl_3_): δ_H_ = 3.76-3.83 (m, 1H, **1-H**), 3.92-3.98 (m, 1H, **1-H**), 5.31 (dd, *J* = 7.2, 3.2 Hz, 1H, **2-H**), 5.64 (1H, **16-H**), 6.87 (dd, *J* = 8.1,1.1 Hz, 1H, **7-H**), 6.99-7.02 (m, 2H, **10-H**), 7.10-7.18 (m, 2H, **5,12-H**), 7.25 (dd, *J* = 8.0, 1.7 Hz, 1H, **6-H**), 7.33-7.38 (m, 2H, **11-H**), 7.60 (d, *J* = 1.7 Hz, 1H, **4-H**), 7.61 (d, *J* = 2.8 Hz, 1H, **19-H**), 7.89 (d, *J* = 2.8 Hz, 1H, **18-H**); ^13^C NMR (100 MHz, CDCl_3_): δ_C_ = 47.9 (**C-1**), 69.3 (**C-2**), 118.2 (**C-7,10**), 123.2 (**C-5**), 123.5 (**C-12**), 127.2 (**C-4**), 128.6 (**C-6**), 129.6 (**C-11**), 130.8 (**C-19**), 132.2 (**C-3**), 134.8 (**C-20**), 139.2 (**C-18**), 151.0 (**C-17**), 153.4 (**C-9**), 156.6 (**C-8**); LRMS m/z (CI^+^): 325 [M(^37^Cl)-OH]^+^, 323 [M(^35^Cl)-OH]^+^; HRMS m/z (ESI^-^): Found 340.0867 [M(^35^Cl)-H]^-^; C_18_H_15_ClN_3_O_2_ requires 340.0853.

**2-[(3-chloropyrazin-2-yl)amino]-1-(3,4-dimethoxyphenyl)ethanol, 18c**

**14c** (827 g, 4.20 mmol), 2,3-dichloropyrazine (481 µL, 4.62 mmol) and Et_3_N (781 µL, 5.88 mmol) were dissolved in 1,4-dioxane (8 mL) and the reaction was stirred under reflux, under argon. After 16 h, the reaction was cooled to RT, and the solvent removed *in vacuo*. The residual brown oil was taken up in CH_2_Cl_2_ and washed with H_2_O (3 x 30 mL) and brine (1 x 20 mL). The organic extracts were dried (Na_2_SO_4_), filtered and concentrated to give the crude product as a brown oil. Purification was carried out *via* flash chromatography (applied in CH_2_Cl_2_; eluted 0% to 10% to 10% EtOAc) to afford the title compound as a light orange oil (748 mg, 2.42 mmol, 58%). R*_f_* = 0.27 (2:1 CH_2_Cl_2_/EtOAc); IR (ν_max_/cm^-1^, thin film): 3377, 2934; ^1^H NMR (500 MHz, CDCl_3_): δ_H_ = 3.60-3.66 (m, 1H, **1-H**), 3.83-3.89 (m, 1H, **1-H)**, 3.88 (s, 3H, **9-H**), 3.89 (s, 3H, **10-H**), 4.93 (dd, *J* = 7.6, 3.7 Hz, 1H, **2-H**), 5.61 (s, 1H, **12-H**), 6.87 (d, *J* = 8.2 Hz, 1H, **5-H**), 6.93 (d, *J* = 8.2 Hz, 1H, **4-H**), 6.97 (s, 1H, **8-H**), 7.62 (d, *J* = 1.0 Hz, 1H, **16-H**), 7.93 (d, *J* = 1.0 Hz, 1H, **15-H**); ^13^C NMR (125 MHz, CDCl_3_): δ_C_ = 49.0 (**C-1**), 55.6 (**C-9,10**), 73.2 (**C-2**), 108.6 (**C-8**), 110.8 (**C-5**), 117.8 (**C-4**), 130.9 (**C-16**) 134.1 (**C-3**),134.7 (**C-18**),139.6 (**C-15**) 148.4 (**C-7**), 148.8 (**C-6**), 150.9 (**C-13**); LRMS m/z (CI^+^): 312 [M(^37^Cl)+H]^+^, 310 [M(^35^Cl)+H]^+^, 294 [M(^37^Cl)-OH]^+^ 292 [M(^35^Cl)-OH]^+^; HRMS m/z (CI^+^): Found 310.0956 [M(^35^Cl)+H]^+^; C_14_H_17_ClN_3_O_3_ requires 310.0958.

**2-[(3-chloropyrazin-2-yl)amino]-1-(3,5-dimethylphenyl)ethanol, 18d**

**14d** (2.34 g, 14.2 mmol), 2,3-dichloropyrazine (1.62 mL, 15.6 mmol) and Et_3_N (2.76 mL, 19.8 mmol) were dissolved in 1,4-dioxane (24 mL) and the reaction was stirred under reflux, under argon. After 16 h, the reaction was cooled to RT, and the solvent removed *in vacuo*. The residue was taken up in CH_2_Cl_2_ and washed with H_2_O (3 x 30 mL) and brine (1 x 20 mL). The organic extracts were dried (MgSO_4_), filtered and concentrated to give the crude product as a brown oil. Purification was carried out *via* flash chromatography (applied in CH_2_Cl_2_; eluted CH_2_Cl_2_ to 30:1 – 10:1 CH_2_Cl_2_/EtOAc) to afford the title compound as a light orange oil (2.58 g, 9.37 mmol, 65%). R*_f_* = 0.37 (9:1 CH_2_Cl_2_/EtOAc); IR (ν_max_/cm^-1^, thin film): 3422, 2921; ^1^H NMR (600 MHz, CDCl_3_): δ_H_ = 2.33 (s, 6H, **7-H**), 3.27 (s, 1H, **8-H**) 3.61 (ddd, *J* = 13.8, 7.8, 4.8 Hz, 1H, **1-H**), 3.88 (ddd, *J* = 13.8, 6.6, 3.6 Hz, 1H, **1-H**), 4.91 (dd, *J* = 7.9, 2.8 Hz, 1H, **2-H**), 5.62 (bt, 1H, **9-H**), 6.95 (s, 1H, **6-H**), 7.02 (s, 2H, **4-H**), 7.62 (d, *J* = 3.0 Hz. 1H, **13-H**), 7.93 (d, *J* = 3.0 Hz, 1H, **12-H**); ^13^C NMR (150 MHz, CDCl_3_): δ_C_ = 21.5 (**C-7**), 49.53 (**C-1**), 73.8 (**C-2**), 123.7 (**C-4**), 129.7 (**C-6**), 131.2 (**C-13**), 135.3 (**C-15**) 138.4 (**C-5**), 139.7 (**C-12**), 142.5 (**C-3**), 151.3 (**C-10**); LRMS m/z (CI^+^): 280 [M(^37^Cl)+H]^+^, 278 [M(^35^Cl)+H]^+^, 262 [M(^37^Cl)-OH]^+^, 260 [M(^35^Cl)-OH]^+^; HRMS m/z (CI^+^): Found 278.1059 [M(^35^Cl)+H]^+^; C_14_H_17_N_3_OCl requires 278.1060.

**2-[(3-chloropyrazin-2-yl)amino]-1-(3-thienyl)ethanol, 18e**

**13e** (232 mg, 1.63 mmol), 2,3-dichloropyrazine (183 µL, 1.79 mmol), Et_3_N (316 µL, 2.27 mmol) and 1,4-dioxane (2.5 mL) were stirred under reflux, under argon for 18 h. The solvent was removed *in vacuo* and the residue was taken up in CH_2_Cl_2_ (30 mL), washed with H_2_O (5 x 10 mL) and brine (10 mL). The organics were dried (MgSO_4_), filtered and solvent removed to give a brown oil. Flash chromatography (applied in CH_2_Cl_2_; eluted CH_2_Cl_2_ to 2:1 CH_2_Cl_2_/EtOAc) afforded the title compound as an orange oil (193 mg, 0.753 mmol, 46%). R*_f_* = 0.46 (2:1 CH_2_Cl_2_/EtOAc) ; IR (ν_max_/cm^-1^, thin film): 3420, 3091, 2920, 1583, 1525; ^1^H NMR (600 MHz,CDCl_3_): δ_H_ = 3.69-3.74 (m, 1H, **1-H**), 3.96-4.00 (m, 1H, **1-H**), 5.10 (dd, *J* = 7.2, 3.0 Hz, 1H, **2-H**), 5.72 (bs, 1H, **9-H**), 7.13 (dd, *J* = 4.8, 1.2 Hz, 1H, **4-H**), 7.31-7.32 (m, 1H, **7-H**), 7.35 (dd, *J* = 5.4, 3.0 Hz, 1H, **5-H**), 7.65 (d, *J* = 3.0 Hz, 1H, **13-H**), 7.93 (d, *J* = 2.4 Hz, **12-H**); ^13^C NMR (150 MHz, CDCl_3_): δ_C_ = 49.0 (**C-1**), 70.4 (**C-2**), 121.6 (**C-7**), 125.5 (**C-4**), 126.7 (**C-5**), 131.3 (**C-13**), 135.6 (**C-15**), 138.8 (**C-12**), 143.1 (**C-3**), 150.8 (**C-10**); LRMS m/z (ESI^+^): 240 [M(^37^Cl)-OH]^+^, 238 [M(^35^Cl)-OH]^+^; HRMS m/z (ESI^-^): Found 254.0145 [M-H]^-^; C_10_H_9_N_3_OSCl requires 254.0155.

**Synthesis of 2-[(3-chloropyrazin-2-yl)amino]-1-(2-aryl)ethanones**

**2-[(3-chloropyrazin-2-yl)amino]-1-(2-phenoxyphenyl)ethanone, 19b**

DMSO (1.13 mL, 15.98 mmol) was dissolved in anhydrous CH_2_Cl_2_ (100 mL) and the mixture was cooled to and maintained at -78^o^C. Oxalyl chloride (677 µL, 7.99 mmol) was added drop wise and the reaction was stirred for 20 min. **18b** (2.10 g, 6.15 mmol), dissolved in CH_2_Cl_2_ (20 mL), was then added drop wise and after 20 min stirring, Et_3_N (4.08 mL, 30.7 mmol) was added drop wise. The reaction was then allowed to slowly warm to RT over a period of 2.5 h. The reaction was quenched with H_2_O (50 mL) and organics extracted followed by washing with 2.0 M HCl (2 x 40 mL), sat. NaHCO_3_ (1 x 40 mL), H_2_O (1 x 40 mL) and brine (1 x 40 mL). Drying (MgSO_4_), filtration and concentration gave a brown/orange oil. Flash chromatography was carried out (applied in CH_2_Cl_2_; eluted 50:1 to 10:1 CH_2_Cl_2_/EtOAc) to afford the title compound as a yellow solid (1.33 g, 3.92 mmol, 64%). Mpt: 76-78 ^o^C; R*_f_* = 0.74 (9:1 CH_2_Cl_2_/EtOAc); IR (ν_max_/cm^‑1^, thin film): 3423, 3060, 2924; ^1^H NMR (500 MHz, CDCl_3_): δ_H_ = 4.95 (d, *J* = 4.8 Hz, 1H, **1-H**), 6.30 (1H, **15-H**), 6.90 (dd, *J* = 8.4, 0.8 Hz, 1H, **7-H**), 7.11-7.13 (m, 2H, **10-H**), 7.18-7.23 (m, 2H, **5,12-H**), 7.40-7.44 (m, 2H, **11-H**), 7.46-7.49 (m, 1H, **6-H**), 7.59 (d, *J* = 2.7 Hz, 1H, **19-H**), 7.88 (d, *J* = 2.7 Hz, 1H, **18-H**), 8.01 (dd, *J* = 7.9, 1.8 Hz, 1H, **4-H**); ^13^C NMR (125 MHz, CDCl_3_): δ_C_ = 52.0 (**C-1**), 118.1 (**C-7**) 119.4 (**C-10**), 123.0 (**C-5**), 124.3 (**C-12**), 126.5 (**C-3**), 129.9 (**C-11**), 130.7 (**C-4**), 130.8 (**C-19**), 134.3 (**C-6**), 134.8 (**C-21**), 140.0 (**C-18**), 150.2 (**C-16**), 155.2 (**C-9**), 157.2 (**C-8**), 194.8 (**C-2**); LRMS m/z (ESI^+^): 342 [M(^37^Cl)+H]^+^, 340 [M(^35^Cl)+H]^+^, 322 [M(^35^Cl)-OH]^+^; HRMS m/z (ESI^+^): Found 340.0864 [M(^35^Cl)+H]^+^; C_18_H_15_ClN_3_O_2_ requires 340.0853.

**2-[(3-chloropyrazin-2-yl)amino]-1-(3,4-dimethoxyphenyl)ethanone, 19c**

DMSO (417 µL, 5.89 mmol) was dissolved in anhydrous CH_2_Cl_2_ (25 mL) and the mixture was cooled to and maintained at -78^o^C. Oxalyl chloride (249 µL, 2.94 mmol) was added drop wise and the reaction was stirred for 15 min. **18c** (700 mg, 2.26 mmol), dissolved in CH_2_Cl_2_ (20 mL), was then added drop wise and after 15 min stirring, Et_3_N (1.5 mL, 11.31 mmol) was added drop wise. The reaction was then allowed to slowly warm to RT over a period of 2 h. The reaction was quenched with H_2_O (20 mL) and organics extracted followed by washing with 2.0 M HCl (2 x 20 mL), sat. NaHCO_3_ (1 x 20 mL), H_2_O (1 x 20 mL) and brine (1 x 20 mL). Drying (MgSO_4_), filtration and concentration gave an off white solid. Flash chromatography (CH_2_Cl_2_ applied in CH_2_Cl_2_; eluted 50:1 to 10:1 CH_2_Cl_2_/EtOAc) gave the title compound as a white solid (543 mg, 1.76 mmol, 78%). Mpt: 128-130 ^o^C; R*_f_* = 0.31 (5:1 CH_2_Cl_2_/EtOAc); IR (ν_max_/cm^-1^, thin film): 3399, 2936, 1677; ^1^H NMR (500 MHz, CDCl_3_): δ_H_ = 3.96 (s, 3H, **9-H**), 3.97 (s, 3H, **10-H**), 4.88 (d, *J* = 4.2 Hz, 1H, **1-H)**, 6.43 (s, 1H, **12-H**), 6.94 (d, *J* = 8.2 Hz, 1H, **5-H**), 7.57 (d, *J* = 1.7 Hz, 1H, **8-H**), 7.64 (d, *J* = 2.9 Hz, 1H, **16-H**), 7.70 (dd, *J* = 8.2, 2.0 Hz, 1H, **4-H**), 7.96 (d, *J* = 2.5 Hz, 1H, **15-H**); ^13^C NMR (125 MHz, CDCl_3_): δ_C_ = 47.3 (**C-1**), 55.8 (**C-9**), 55.9 (**C-10**) 109.7 (**C-8**), 110.0 (**C-5**), 122.3 (**C-4**), 127.3 (**C-3**), 130.8 (**C-16**), 135.1 (**C-18**), 139.8 (**C-15**) 149.0 (**C-7**), 150.1 (**C-13**), 153.9 (**C-6**), 192.1 (**C-2**); LRMS m/z (CI^+^): 310 [M(^37^Cl)+H]^+^, 308 [M(^35^Cl)+H]^+^; HRMS m/z (CI^+^): Found 308.0814 [M(^35^Cl)+H]^+^; C_14_H_15_ClN_3_O_3_ requires 308.0811.

**2-[(3-chloropyrazin-2-yl)amino]-1-(3,5-dimethylphenyl)ethanone, 19d**

DMSO (1.71 mL, 24.2 mmol) was dissolved in anhydrous CH_2_Cl_2_ (170 mL) and the mixture was cooled to and maintained at -78^o^C. Oxalyl chloride (1.02 mL, 12.1 mmol) was added drop wise and the reaction was stirred for 20 min. **18d** (2.58 g, 9.30 mmol), dissolved in CH_2_Cl_2_ (30 mL), was then added drop wise and after 20 min stirring, Et_3_N (6.18 mL, 46.6 mmol) was added drop wise. The reaction was then allowed to slowly warm to RT over a period of 2.5 h. The reaction was quenched with H_2_O (50 mL) and organics extracted followed by washing with 2.0 M HCl (2 x 40 mL), sat. NaHCO_3_ (1 x 40 mL), H_2_O (1 x 40 mL) and brine (1 x 40 mL). Drying (MgSO_4_), filtration and concentration gave a yellow solid (2.31, 8.37 mmol, 90%) which was carried through to the next stage without further purification. Mpt: Decomposed before melting; R*_f_* = 0.76 (9:1 CH_2_Cl_2_/EtOAc); IR (ν_max_/cm^-1^, thin film): 3405, 2916, 1681, 1578, 1497; ^1^H NMR (500 MHz, CDCl_3_): δ_H_ = 2.40 (s, 6H, **7-H**), 4.89 (d, *J* = 4.0 Hz, 1H, **1-H**), 6.43 (s, 1H, **8-H**), 7.27 (s, 1H, **6-H**), 7.65 (d, *J* = 3.0 Hz. 1H, **12-H**), 7.67 (s, 2H, **4-H**), 7.97 (d, *J* = 3.0 Hz, 1H, **11-H**); ^13^C NMR (125 MHz, CDCl_3_): δ_C_ = 20.9 (**C-7**), 47.8 (**C-1**), 125.4 (**C-4**), 130.9 (**C-12**), 134.2 (**C-3**), 135.1 (**C-14**), 135.5 (**C-6**), 138.4 (**C-5**), 139.8 (**C-11**), 150.0 (**C-9**), 193.6 (**C-2**); LRMS m/z (ESI^-^): 276 [M(^37^Cl)-H]^-^, 274 [M(^35^Cl)-H]^-^; HRMS m/z (ESI^-^): Found 274.0762 [M(^35^Cl)-H]^-^; C_14_H_13_N_3_OCl requires 274.0747.

**2-[(3-chloropyrazin-2-yl)amino]-1-(3-thienyl)ethanone, 19e**

DMSO (128 µL, 1.81 mmol) was dissolved in anhydrous CH_2_Cl_2_ (9 mL) and the mixture was cooled to and maintained at -78^o^C. Oxalyl chloride (77.0 µL, 0.906 mmol) was added drop wise and the reaction was stirred for 20 min. **18e** (178 mg, 0.697 mmol), dissolved in CH_2_Cl_2_ (5 mL), was then added drop wise and after 20 min stirring, Et_3_N (463 µL, 3.48 mmol) was added drop wise. The reaction was then allowed to slowly warm to RT over a period of 2 1/2 h. The reaction was quenched with H_2_O (20 mL) and organics extracted followed by washing with 2.0 M HCl (2 x 10 mL), sat. NaHCO_3_ (1 x 10 mL), H_2_O (1 x 10 mL) and brine (1 x 10 mL). Drying (MgSO_4_), filtration and concentration gave an orange sticky solid. Flash chromatography (applied in CH_2_Cl_2_; eluted 50:1 to 20:1 CH_2_Cl_2_/EtOAc) gave the title compound as a yellow solid (135 mg, 0.531 mmol, 76%). Mpt: 130-134 ^o^C; R*_f_* = 0.85 (2:1 CH_2_Cl_2_/EtOAc); IR (ν_max_/cm^-1^, thin film): 3407, 3107, 2917, 1682, 1582; ^1^H NMR (600 MHz, CDCl_3_): δ_H_ = 4.84 (d, *J* = 4.4 Hz, 1H, **1-H**), 6.33(s, 1H, **9-H**), 7.41 (dd, *J* = 5.0, 2.8 Hz, 1H, **5-H**), 7.65 (dd, *J* = 5.0, 1.2 Hz, 1H, **4-H**), 7.66 (d, *J* = 2.8 Hz, 1H, **13-H**), 7.96 (d, *J* = 2.7 Hz, **12-H**), 8.26 (dd, *J* = 5.4, 3.0 Hz, 1H, **7-H**); ^13^C NMR (150 MHz, CDCl_3_): δ_C_ = 49.0 (**C-1**), 70.4 (**C-2**), 121.6 (**C-4**), 125.5 (**C-7**), 126.7 (**C-5**), 131.3 (**C-13**), 135.6 (**C-15**), 138.8 (**C-12**), 143.1 (**C-3**), 150.8 (**C-10**); LRMS m/z (CI^+^): 256 [M(^37^Cl)+H]^+^, 254 [M(^35^Cl)+H]^+^; HRMS m/z (CI^+^): Found 254.0147 [M(^35^Cl)+H]^+^; C_10_H_9_N_3_OSCl requires 254.0155.

**Synthesis of 3-aryl-8-chloro-imidazo[1,2-*a*]pyrazines**

**8-chloro-3-(2-phenoxyphenyl)imidazo[1,2-*a*]pyrazine, 20b**

**19b** (1.33 g, 3.92 mmol) was dissolved in anhydrous toluene (50 mL) and the mixture was cooled on ice. Trifluoroacetic acid (2.11 mL, 24.7 mmol) was added and the reaction was allowed to stir on ice for 30 min, followed by the addition of trifluoroacetic anhydride (3.81 mL, 24.7 mmol). The reaction mixture was then stirred on ice for a further 30 min and then at RT for 68 h. The reaction was then diluted with toluene (50 mL) and washed with NaHCO_3_ solution (10% w/v, 3 x 30 mL) and brine (1 x 40 mL). The organics were dried (MgSO_4_), filtered and concentrated to give crude brown oil. Purification was carried out *via* flash chromatography (applied in CH_2_Cl_2_; eluted 50:1 to 5:1 CH_2_Cl_2_/EtOAc to afford the title compound as a yellow sticky solid (1.26 g, 3.92 mmol, 100%). R*_f_* = 0.25 (9:1 CH_2_Cl_2_/EtOAc); IR (ν_max_/cm^-1^, thin film): 1460, 1231; ^1^H NMR (600 MHz, CDCl_3_): δ_H_ = 6.83–6.85 (m, 2H, **18-H**), 7.05-7.10 (m, 2H, **14,20-H**), 7.23-7.26 (m, 2H, **19-H**), 7.29 (td, *J* = 7.8, 1.2 Hz, 1H, **12-H**), 7.46-7.49 (m, 1H, **13-H**), 7.53 (dd, *J* = 7.2, 1.2 Hz, 1H, **11-H**), 7.70 (d, *J* = 4.2 Hz, 1H, **6-H**), 7.86 (s, 1H, **2-H**), 8.01 (d, *J* = 4.2 Hz, 1H, **5-H**); ^13^C NMR (150 MHz, CDCl_3_): δ_C_ = 118.3 (**C-5**), 118.5 (**C-18**), 118.8 (**C-22**), 124.2 (**C-10,12,20**), 126.1 (**C-3**), 130.0 (**C-19**), 131.5 (**C-13**), 132.0 (**C-11**), 135.6 (**C-2**), 138.4 (**C-9**), 143.9 (**C-8**), 155.0 (**C-15**), 156.0 (**C-17**); LRMS m/z (EI^+^): 323 [M(^37^Cl)]^+^, 321 [M(^35^Cl)]^+^; HRMS m/z (EI^+^): Found: 321.0659 [M(^35^Cl)]^+^; C_18_H_12_ClN_3_O requires 321.0663.

**8-chloro-3-(3,4-dimethoxyphenyl)imidazo[1,2-*a*]pyrazine, 20c**

**19c** (280 mg, 0.912 mmol) was dissolved in anhydrous toluene (20 mL) and the mixture was cooled on ice. Trifluoroacetic acid (490 µL, 6.39 mmol) was added and the reaction was allowed to stir on ice for 30 min, followed by the addition of trifluoroacetic anhydride (887 µL, 6.39 mmol). The reaction mixture was then stirred on ice for a further 30 min and then at RT for 68 h. The reaction was then diluted with toluene (50 mL) and washed with NaHCO_3_ solution (10% w/v, 3 x 30 mL) and brine (1 x 40 mL). The organics were dried (MgSO_4_), filtered and concentrated to give crude yellow solid. Purification was carried out *via* flash chromatography (applied in CH­_2_Cl_2_; eluted 9:1 to 2:1 CH_2_Cl_2_/EtOAc) to afford the title compound as a white solid (46.8 mg, 0.162 mmol, 18%). Mpt: > 200 ^o^C; R*_f_* = 0.32 (2:1 CH_2_Cl_2_/EtOAc); IR (ν_max_/cm^-1^, thin film): 2960, 2924, 1732; ^1^H NMR (400 MHz, CDCl_3_): δ_H_ = 3.93 (s, 3H, **16-H**), 3.96 (s, 3H, **17-H**), 6.99 (d, *J* = 2.0 Hz, 1H, **15-H**), 7.03 (d, *J* = 8.2 Hz, 1H, **12-H**), 7.10 (dd, *J* = 8.3, 2.0 Hz, 1H, **11-H**), 7.67 (d, *J* = 4.6 Hz, 1H, **6-H**), 7.84 (s, 1H, **2-H**), 8.15 (d, *J* = 4.6 Hz, 1H, **5-H**); ^13^C NMR (100 MHz, CDCl_3_): δ_C_ = 56.1 (**C-16**), 56.2 (**C-17**), 111.4 (**C-15**), 111.8 (**C-12**). 116.3 (**C-5**), 119.8 (**C-10**), 121.0 (**C-11**), 128.2 (**C-6**), 129.2 (**C-3**), 134.2 (**C-2**), 138.0 (**C-9**), 144.3 (**C-8**), 149.8 (**C-14**), 150.1 (**C-13**); 290 [M(^35^Cl)+H]^+^, 292 [M(^37^Cl)+H]^+^; HRMS m/z (ESI^+^): Found 290.0683 [M(^35^Cl)+H]^+^; C_14_H_13_N_3_O_2_Cl requires 290.0696.

**8-chloro-3-(3,5-dimethylphenyl)imidazo[1,2-*a*]pyrazine, 20d**

**19d** (2.29 g, 8.31 mmol) was dissolved in anhydrous toluene (90 mL) and the mixture was cooled on ice. Trifluoroacetic acid (4.48 mL, 58.1 mmol) was added and the reaction was allowed to stir on ice for 30 min, followed by the addition of trifluoroacetic anhydride (8.09 mL, 58.1 mmol). The reaction mixture was then stirred on ice for a further 30 min and then at RT for 68 h. The reaction was then diluted with toluene (50 mL) and washed with NaHCO_3_ solution (10% w/v, 3 x 40 mL) and brine (1 x 40 mL). The organics were dried (MgSO_4_), filtered and concentrated to give crude orange sticky solid. Purification was carried out *via* flash chromatography (applied in CH_2_Cl_2_; eluted 40:1 CH_2_Cl_2_/EtOAc) to afford the title compound as a yellow solid (531.7 g, 2.06 mmol, 25%). Mpt: 178-180 ^o^C; R*_f_* = 0.32 (9:1 CH_2_Cl_2_/EtOAc); IR (ν_max_/cm^-1^, thin film): 2918, 1603; ^1^H NMR (600 MHz, CDCl_3_): δ_H_ = 2.41 (s, 6H, **14-H**), 7.14 (s, 1H, **13-H**), 7.16 (s, 2H, **11-H**), 7.69 (d, *J* = 4.6 Hz, 1H, **6-H**), 7.87 (s, 1H, **2-H**), 8.21 (d, *J* = 4.6 Hz, 1H, **5-H**); ^13^C NMR (150 MHz, CDCl_3_): δ_C_ = 21.5 (**C-14**), 116.6 (**C-5**), 125.9 (**C-11**), 127.4 (**C-10**), 128.3 (**C-6**), 129.6 (**C-3**), 131.3 (**C-13**), 134.5 (**C-2**), 138.3 (**C-9**), 139.5 (**C-12**), 144.4 (**C-8**); LRMS m/z (EI^+^): 259 [M(^37^Cl)]^+^, 257 [M(^35^Cl)]^+^; HRMS m/z (CI^+^): Found: 257.0717 [M(^35^Cl)]^+^; C_14_H_12_ClN_3_ requires 257.0714.

**8-chloro-3-(3-thienyl)imidazo[1,2-*a*]pyrazine , 20e**

**19e** (345 mg, 1.36 mmol) was dissolved in anhydrous toluene (15 mL) and the mixture was cooled on ice. Trifluoroacetic acid (790 µL, 10.3 mmol) was added and the reaction was allowed to stir on ice for 30 minutes, followed by the addition of trifluoroacetic anhydride (1.43 L, 10.3 mmol). The reaction mixture was then stirred on ice for a further 30 minutes and then at RT for 68 h. The reaction was then diluted with toluene (20 mL) and washed with NaHCO_3_ solution (10% w/v, 3 x 10 mL) and brine (1 x 10 mL). The organics were dried (MgSO_4_), filtered and concentrated to give crude orange oil. Purification was carried out *via* flash chromatography (applied in CH_2_Cl_2_; eluted 20:1 to 5:1 CH_2_Cl_2_/EtOAc) to afford the title compound as an off white solid (105 mg, 0.477 mmol, 33%). Mpt: 178-180 ^o^C; R*_f_* = 0.34 (5:1 CH_2_Cl_2_/EtOAc); IR (ν_max_/cm^-1^, thin film): 3098, 1462; ^1^H NMR (600 MHz, CDCl_3_): δ_H_ = 7.34–7.36 (m, 1H, **12-H**), 7.58 (s, 1H, **14-H**), 7.59 (d, *J* = 0.7 Hz, 1H, **11-H**), 7.73 (d, *J* = 4.6 Hz, 1H, **6-H**), 7.91 (s, 1H, **2-H**), 8.19 (d, *J* = 4.6 Hz, 1H, **5-H**); ^13^C NMR (150 MHz, CDCl_3_): δ_C_ = 116.7 (**C-5**), 124.2 (**C-14**), 125.0 (**C-10**), 126.7 (**C-12**), 127.7 (**C-3**), 128.0 (**C-11**), 128.6 (**C-6**), 134.6 (**C-2**), 138.1 (**C-9**), 144.5 (**C-8**); LRMS m/z (CI^+^): 238 [M(^37^Cl)+H]^+^, 236 [M(^35^Cl)+H]^+^; HRMS m/z (CI^+^): Found 236.0057 [M(^35^Cl)+H]^+^; C_10_H_7_N_3_SCl requires 236.0049.

**Synthesis of 2-aryl-8-chloro-imidazo[1,2-*a*]pyrazines**

**8-chloro-2-(2-phenoxyphenyl)imidazo[1,2-*a*]pyrazine*,* 21b**

**15b** (438 mg, 1.50 mmol), 2-amino-3-chloropyrazine (195 mg, 1.50 mmol), NaHCO_3_ (158 mg, 1.88 mmol) and *^t^*BuOH (9 mL) were stirred under reflux for 48 h. The reaction was cooled to RT and solvent removed *in vacuo*. The sample was taken up in CH_2_Cl_2_ (25 mL) and washed with H_2_O (3 x 10 mL). The combined aqueous extracts were further washed with CH_2_Cl_2_ (4 x 10 mL), and the organic extracts were combined, dried (MgSO_4_), filtered and solvent removed. Flash chromatography (applied in pet. ether; eluted 25:1 to 10:1 to 5:1 to 3:1 pet. ether/EtOAc ) afforded a pale yellow solid. (156 mg, 0.485 mmol, 32%). Mpt: > 200 ^o^C; R*_f_* = 0.75 (1:1 pet. ether/EtOAc); IR (ν_max_/cm^-1^, thin film): 1071 – 1225; ^1^H NMR (600 MHz, CDCl_3_): δ_H_ = 6.97 (dd, *J* = 1.5, 8.2 Hz, 1H, **14-H**), 7.07 (d, *J* = 7.6 Hz, 2H, **18-H**), 7.16 (t, *J* = 7.4 Hz, 1H, **20-H**), 7.29 – 7.34 (m, 2H, **12,13-H**), 7.38 (dd, *J* = 8.6, 7.5 Hz, 2H, **19-H**), 7.65 (d, *J* = 4.5 Hz, 1H, **6-H**), 7.99 (d, *J* = 4.5 Hz, 1H, **5-H**), 8.31 (s, 1H, **3-H**), 8.59 (dd, *J* = 7.6, 1.9 Hz, 1H, **11-H**); ^13^C NMR (150 MHz, CDCl_3_): δ_C_ = 115.3 (**C-3**), 118.5 (**C-5**), 118.8 (**C-18**), 119.0 (**C-14**), 123.7 (**C-20**), 123.8 (**C-10**), 124.1 (**C-12**), 127.8 (**C-6**), 129.7 (**C-13**), 129.9 (**C-19**), 130.1 (**C-11**), 137.1 (**C-9**), 143.2 (**C-2**), 143.3 (**C-8**), 154.6 (**C-15**), 156.5 (**C-17**); LRMS m/z (ESI^+^): 324 [M(^37^Cl)+H]^+^, 322 [M(^35^Cl)+H]^+^; HRMS m/z (CI^+^): Found 322.07554 [M(^35^Cl)+H]^+^; C_18_H_13_ClN_3_O requires 322.0747.

**8-chloro-2-(3,4-dimethoxylphenyl)imidazo[1,2-*a*]pyrazine, 21c**

A mixture of **15c** (229 mg, 0.885 mmol), 2-amino-3-chloropyrazine (115 mg, 0.885 mmol) and NaHCO_3_ (93.0 mg, 1.11 mmol) in *^t^*BuOH (5 mL) were stirred under argon, under reflux for 40 h. After this point, the reaction mixture was cooled to RT and solvent removed *in vacuo*. The resulting orange solid was dissolved in CH_2_Cl_2_ (30 mL) and washed with H_2_O (3 x 30 mL). The aqueous layers were extracted further with CH_2_Cl_2_ (2 x 20 mL) before the combined organics were washed with brine (20 mL), dried (MgSO_4_), filtered and solvent removed to give a light brown solid. Flash chromatography (applied in pet. ether; eluted 3:1 pet. ether /EtOAc) was carried out to afford the title compound (155 mg, 0.536 mmol, 61%). Mpt: Decomposed before melting; R*_f_* = 0.11 (1:1 pet. ether/EtOAc); IR (ν_max_/cm^-1^, thin film): 3142, 2933, 2833, 1498; ^1^H NMR (600 MHz, CDCl_3_): δ_H_ = 3.93 (s, 3H, **16-H**), 4.00 (s, 3H, **17-H**), 6.93 (d, *J* = 8.3 Hz, 1H, **12-H**), 7.48 (dd, *J* = 8.2, 2.1 Hz, 1H, **11-H**), 7.59 (d, *J* = 2.1 Hz, 1H, **15-H**), 7.65 (d, *J* = 4.5, 1H, **6-H**), 7.96 (s, 1H, **3-H**), 8.02 (d, *J* = 4.5 Hz, 1H, **5-H**); ^13^C NMR (150 MHz, CDCl_3_): δ_C_ = 56.0 (**C-16**), 56.1 (**C-17**), 109.6 (**C-15**), 110.6 (**C-3**), 111.2 (**C-12**). 118.3 (**C-5**), 119.3 (**C-11**), 125.2 (**C-10**), 128.0 (**C-6**), 138.0 (**C-9**), 143.1 (**C-8**), 148.3 (**C-2**), 149.3 (**C-13**). 149.9 (**C-14**); LRMS m/z (ESI^+^): 292 [M^37^Cl+H]^+^, 290 [M(^35^Cl)+H]^+^; HRMS m/z (ESI^+^): Found 290.0687 [M(^35^Cl)+H]^+^; C_14_H_13_ClN_3_O_2_ requires 290.0696.

**8-chloro-2-(3,5-dimethylphenyl)imidazo[1,2-*a*]pyrazine, 21d**

A mixture of **15d** (530 mg, 2.33 mmol), 2-amino-3-chloropyrazine (302 mg, 2.33 mmol) and NaHCO_3_ (245 mg, 2.92 mmol) in *^t^*BuOH (11 mL) were stirred under argon, under reflux for 40 h. After this point, the reaction mixture was cooled to RT and solvent removed *in vacuo*. The resulting orange solid was dissolved in CH_2_Cl_2_ (100 mL) and washed with H_2_O (3 x 40 mL). The aqueous layers were extracted further with CH_2_Cl_2_ (2 x 50 mL) before the combined organics were washed with brine (75 mL), dried (MgSO_4_), filtered and solvent removed to give crude orange solid. Flash chromatography (1^st^: applied in pet. ether; eluted 9:1 to 5:1 to 2:1 pet. ether/EtOAc; 2^nd^: applied in CH_2_Cl_2_; eluted 9:1 CH_2_Cl_2_/EtOAc) was carried out to afford the title compound (245 mg, 0.951 mmol, 41%). Mpt: 158-162 ^o^C; R*_f_* = 0.68 (2:1 CH_2_Cl_2_/EtOAc); IR (ν_max_/cm^-1^, thin film): 2920, 1365; ^1^H NMR (500 MHz, CDCl_3_): δ_H_ = 2.38 (s, 6H, **14-H**), 7.05 (s, 1H, **13-H**), 7.62 (s, 2H, **11-H**), 7.66 (d, *J* = 4.5 Hz, 1H, **6-H**), 7.99 (s, 1H, **3-H**). 8.02 (d, *J* = 4.5 Hz, 1H, **5-H**); ^13^C NMR (125 MHz, CDCl_3_): δ_C_ = 21.3 (**C-14**), 111.3 (**C-3**), 118.4 (**C-5**), 124.5 (**C-11**), 128.0 (**C-6**), 131.1 (**C-13**), 132.1 (**C-10**), 138.2 (**C-9**), 138.6 (**C-12**), 143.4 (**C-8**). 148.6 (**C-2**); LRMS m/z (EI^+^): 257 [M(^35^Cl)]^+^, 259 [M(^37^Cl)]^+^; HRMS m/z (EI^+^): Found 257.0719 [M(^35^Cl)]^+^; C_14_H_12_ClN_3_ requires 257.0714.

**8-chloro-2-(3-thienyl)imidazo[1,2-*a*]pyrazine, 21e**

**15e** (293 mg, 1.43 mmol), 2-amino-3-chloropyrazine (185 mg, 1.43 mmol), NaHCO_3_ (150 mg, 1.79 mmol) and *^t^*BuOH (6 mL) were stirred under reflux for 41 h. The reaction was cooled to RT and solvent removed *in vacuo*. The sample was taken up in water (15 mL) and extracted with DCM (2 x 20 mL). The combined organics were washed with H_2_O (2 x 15 mL) and brine (15 mL) before they were dried (MgSO_4_), filtered and concentrated to give crude brown/orange solid. Flash chromatography (applied in pet. ether; eluted 2:1 pet. ether /EtOAc) afforded a pale yellow solid (109 mg, 0.464 mmol, 32.4%). Mpt: decomposed before melting; R*_f_* = 0.33 (1:1 pet. ether /EtOAc); IR (ν_max_/cm^-1^, thin film): 1240 – 1480; ^1^H NMR (600 MHz, CDCl_3_): δ_H_ = 7.44 (dd, *J* = 5.0, 3.0 Hz, 1H, **12-H**), 7.58 (dd, *J* = 5.0, 1.3 Hz, 1H, **11-H**), 7.69 (d, *J* = 4.4 Hz, 1H, **6-H**), 7.93 (s, 1H, **3-H**), 7.96 (dd, *J* = 3.0, 1.3 Hz, 1H **14-H**), 8.04 (d, *J* = 4.4 Hz, 1H, **5-H**); ^13^C NMR (150 MHz, CDCl_3_): δ_C_ = 111.1 (**C-3**), 118.4 (**C-5**), 123.6 (**C-14**), 126.0 (**C-12**), 126.6 (**C-11**), 128.1 (**C-6**), 134.1 (**C-10**), 138.0 (**C-9**), 143.3 (**C-8**), 144.3 (**C-2**); LRMS m/z (ESI^+^) 238 [M(^37^Cl)+H]^+^, 236 [M(^35^Cl)+H]^+^; HRMS m/z (ESI^+^): Found: 236.0056 [M(^35^Cl)+H]^+^; C_10_H_7_ClN_3_S requires 236.0049.

**Synthesis of first generation 2- and 3-substituted 8-aminoimidazo[1,2-*a*]pyrazines**

**4-methyl-*N*-[2-(2-naphthyl)imidazo[1,2-*a*]pyrazine-8-yl]benzenesulfonamide, 1**

All glassware was evacuated and flushed with argon prior to use. **20a** (12.3 mg, 0.044 mmol), 4-toluenesulfonamide **22** (9.10 mg, 0.053 mmol), K_2_CO_3_ (7.30 mg, 0.053 mmol), 1 mol% Pd(dba)_2_ (0.120 mg) and 5 mol% *tert-*butyl XPhos (0.600 mg) were weighed into a 5 mL round bottom flask. *^t^*BuOH (1 mL) was added and the reaction was stirred under reflux for 40 h. The reaction mixture was cooled to RT, diluted with MeOH and filtered through Celite (pre-washed with MeOH). Flash chromatography (applied in CH_2_Cl_2_; eluted 10:1 to 1:1 CH_2_Cl_2_/EtOAc) afforded the target compound as a white solid (7.50 mg, 0.018 mmol, 41%). Mpt: Decomposed before melting; R*_f_* = 0.62 (1:1 CH_2_Cl_2_/EtOAc); IR (ν_max_/cm^-1^, thin film): 3258, 3112, 2923, 2854, 1579; ^1^H NMR (600 MHz, DMSO-*d_6_*): δ_H_ = 2.36 (s, 3H, **26-H**), 7.16 (bs, 1H, **6-H**), 7.39 (d, *J* = 8.2 Hz, 2H, **24-H**), 7.60 - 7.62 (m, 2H, **15,16-H**), 7.74 (d, *J* = 8.9 Hz, 1H, **11-H**), 7.87 (bd, *J* = 6.8 Hz, 1H, **5, 23-H**) 7.91 (s, 1H, **2-H**), 8.00 - 8.04 (m, 2H, **14**,**17-H**), 8.11 (d, *J* = 8.2 H, **12-H**), 8.21 (s, 1H, **19-H**), 11.70 (s, 1H, **20-H**); ^13^C NMR (150 MHz, DMSO-*d_6_*): δ_C_ = 21.0 (**C-26**), 108.7 (**C-5**), 116.8 (**C-6**), 124.6 (**C-10**), 125.7 (**C-11**), 126.1 (**C-23**), 127.1 (**C-15,16**), 127.4 (**C-19**), 128.1 (**C-14,17**), 129.0 (**C-12**), 129.6 (**C-24**), 130.6 (**C-3**), 132.8 (**C-13,18**), 133.3 (**C-2**), 135.6 (**C-9**), 140.1 (**C-22**), 142.6 (**C-25**), 144.5 (**C-8**); LRMS m/z (ESI^+^): 415 [M+H]^+^, 260 [M-SO_2_C_6_H_4_CH_3_+H]^+^; HRMS m/z (ESI^+^): Found 415.1230 [M+H]^+^; C_23_H_19_N_4_O_2_S requires 415.1229.

**4-methyl-*N*-[2-phenoxyphenyl)imidazo[1,2-*a*]pyrazine-8-yl]benzenesulfonamide, 2**

All glassware was evacuated and flushed with argon prior to use. **20b** (516 mg, 1.60 mmol), 4-toluenesulfonamide **22** (330 mg, 1.93 mmol), K_2_CO_3_ (266 mg, 1.93 mmol), 1 mol% Pd(dba)_2_ (5.20 mg) and 5 mol% *tert-*butyl XPhos (26.0 mg) were weighed into a 50 mL round bottom flask. *^t^*BuOH (10 mL) was added and the reaction was stirred under reflux for 40 h. The reaction mixture was cooled to RT, diluted with MeOH and filtered through Celite (pre-washed with MeOH). Flash chromatography was carried out (applied in CH_2_Cl_2_; eluted 10:1 CH_2_Cl_2_/EtOAc to 1:1 gradient followed by 10% MeOH/CH_2_Cl_2_) to afford the title compound as yellow solid (188 mg, 0.412 mmol 26%). Mpt: > 200 ^o^C; R*_f_* = 0.18 (2:1 CH_2_Cl_2_/EtOAc); IR (ν_max_/cm^-1^, thin film): 3243, 2917, 1588; ^1^H NMR (600 MHz, CDCl_3_): δ_H_ = 2.37 (s, 3H, **27-H**), 6.85 (d, *J* = 7.8 Hz, 2H, **18-H**), 7.04 (d, *J* = 8.4 Hz, 2H, **6, 14H**), 7.07 (t, *J* = 7.2 Hz, 1H, **20H**), 7.23-7.27 (m, 5H, **12, 19, 25-H**), 7.43-7.46 (m, 3H, **5, 11, 13-H**), 7.65 (s, 1H, **2-H**), 7.94 (bs, 2H, **24-H**), 11.45 (bs, 1H, **21-H**); ^13^C NMR (150 MHz, CDCl_3_): δ_C_ = 21.7 (**C-27**), 109.5 (**C-5**), 115.1 (**C-6**), 118.6 (**C-10**), 118.8 (**C-18**), 118.9 (**C-14**), 124.0 (**C-12**), 124.2 (**C-20**), 126.8 (**C-24**), 128.1 (**C-3**), 129.4 (**C-25**), 130.0 (**C-19**), 131.5 (**C-13**), 132.1 (**C-11**), 134.6 (**C-2**), 136.0 (**C-9**), 139.5 (**C-23**), 143.2 (**C-26**), 146.0 (**C-8**), 155.0 (**C-15**), 155.9 (**C-17**); LRMS m/z (ESI^+^): 457 [M+H]^+^, 302 [M-C_6_H_5_-SO_2_]^+^. HRMS m/z (ESI^‑^): Found: 455.1183 [M-H]^-^; C_25_H_19_N_4_O_3_S requires 455.1178.

***N*-[3-(3,4-dimethoxyphenyl)imidazo[1,2-*a*]pyrazine-8-yl]-4-methyl-benzene­sulfonamide, 3**

All glassware was evacuated and flushed with argon prior to use. **20c** (95.0 mg, 0.033 mmol), 4-toluenesulfonamide **22** (67.5 mg, 0.039 mmol), K_2_CO_3_ (54.4 mg, 0.039 mmol), 1 mol% Pd(dba)_2_ (0.950 mg) and 5 mol% *tert-*butyl XPhos (4.75 mg) were weighed into a 10 mL round bottom flask. *^t^*BuOH (2 mL) was added and the reaction was stirred under reflux for 40 h. The reaction mixture was cooled to RT, diluted with MeOH and filtered through Celite (pre-washed with MeOH). Flash chromatography was carried out (applied in CH_2_Cl_2_; eluted 50:1 to 20:1 to 5:1 to 2:1 to 1:1 CH_2_Cl_2_/EtOAc) to afford the title compound as a yellow solid (42.3 mg, 0.010 mmol, 30.5%). Mpt: >200 ^o^C; R*_f_* = 0.08 (1:1 CH_2_Cl_2_/EtOAc); IR (ν_max_/cm^-1^, thin film): 3244, 3129, 2838, 1582; ^1^H NMR (600 MHz, CDCl_3_): δ_H_ = 2.34 (s, 3H, **24-H**), 3.89 (s, 3H, **16-H**), 3.93 (s, 3H, **17-H**), 6.93 (d, *J* = 1.8 Hz, 1H, **15-H**), 6.98 (d, *J* = 8.3 Hz, 1H, **12-H**), 7.02 (dd, *J* = 8.2, 1.8 Hz, **11-H**), 7.13 (bs, 1H, **6-H**), 7.21 (d, *J* = 8.1 Hz, 2H, **22-H**), 7.41 (bs, 1H, **5-H**), 7.64 (s, 1H, **2-H**), 7.92 (d, *J* = 8.1 Hz, 2H, **21-H**), 11.53 (s, 1H, **18-H**); ^13^C NMR (150 MHz, CDCl_3_): δ_C_ = 21.5 (**C-24**), 56.1 (**C-16**), 56.2 (**C-17**), 108.0 (**C-5**), 111.5 (**C-15**), 111.7 (**C-12**), 116.1 (**C-6**), 119.5 (**C-10**), 121.2 (**C-11**), 126.6 (**C-21**), 129.4 (**C-22**), 131.4 (**C-3**), 133.1 (**C-2**), 135.6 (**C-9**), 139.3 (**C-20**), 143.2 (**C-23**), 145.8 (**C-8**), 149.6 (**C-14**), 150.1 (**C-13**); LRMS m/z (ESI^+^): 425 [M+H]^+^, 447 [M+Na]^+^, HRMS m/z (ESI^-^): Found 423.0940 [M-H]^+^; C_21_H_21_N_4_0_4_S requires 423.0949.

***N*-[3,5-dimethylphenyl)imidazo[1,2-*a*]pyrazine-8-yl]-4-methyl-benzenesulfonamide, 4**

All glassware was evacuated and flushed with argon prior to use. **20d** (93.8 mg, 0.364 mmol), 4-toluenesulfonamide **22** (74.8 mg, 0.437 mmol), K_2_CO_3_ (60.4 mg, 0.437 mmol), 1 mol% Pd(dba)_2_ (2.10 mg) and 5 mol% *tert-*butyl XPhos (7.70 mg) were weighed into a 10 mL round bottom flask. *^t^*BuOH (2 mL) was added and the reaction was stirred under reflux for 40 h. The reaction mixture was cooled to RT, diluted with MeOH and filtered through Celite (pre-washed with MeOH). Flash chromatography was carried out (applied in CH_2_Cl_2_; eluted 9:1 to 1:1 CH_2_Cl_2_/EtOAc followed by 5% MeOH/CH_2_Cl_2_) to afford the title compound as a pale yellow solid (96.2 mg, 0.245 mmol, 67%). Mpt: >200 ^o^C; R*_f_* = 0.5 (CH_2_Cl_2_/10% MeOH); IR (ν_max_/cm^-1^, thin film): 3253, 1591; ^1^H NMR (600 MHz, CD_2_Cl_2_): δ_H_ = 2.38 (s, 6H, **14-H**), 2.39 (s, 3H, **21-H**), 6.98 (bs, 1H, **6-H**), 7.11 (s, 2H, **11-H**), 7.13 (s, 1H, **13-H**), 7.30 (d, *J* = 8.0 Hz, 2H, **19-H**), 7.50 (bs, 1H, **5-H**), 7.64 (s, 1H, **2-H**), 7.90 (bd, *J* = 6.1 Hz, 2H, **18-H**), 11.33 (bs, 1H, **15-H**); ^13^C NMR (150 MHz, CD_2_Cl_2_): δ_C_ = 21.4 (**C-14**), 21.7 (**C-21**), 108.9 (**C-5**), 115.8 (**C-6**), 125.9 (**C-3**), 126.4 (**C-11**), 126.8 (**C-18**), 129.8 (**C-19**), 131.4 (**C-13**), 132.2 (**C-10**), 133.6 (**C-2**), 136.1 (**C-9**), 139.4 (**C-12**), 139.7 (**C-17**), 143.9 (**C-20**), 146.2 (**C-8**); LRMS m/z (ESI^+^): 415 [M+Na]^+^, 393 [M+H]^+^; HRMS m/z (ESI^+^): Found: 393.1366 [M+H]^+^; C_21_H_21_N_4_O_2_S requires 393.1385.

**4-methyl-*N*-[4-[[3-(3-thienyl)imidazo[1,2-*a*]pyrazine-8-yl]amino]phenyl]benzene­sulfonamide, 6**

All glassware was evacuated and flushed with argon prior to use. **20e** (94.9 mg, 0.403 mmol), *N*-(4-aminophenyl)-4-methylbenzenesulfonamide **23** (126.8 mg, 0.484 mmol, 1.2 equiv.), NaO^t^Bu (54.2 mg, 0.564 mmol, 1.4 equiv.), 1 mol% Pd_2_(dba)_3_ (3.7 mg) and 3 mol% DavePhos (4.8 mg) were weighed into a 25 mL round bottom flask. Toluene (4 mL) was added and the reaction was stirred under reflux for 18 h. The reaction mixture was cooled to RT and the solvents removed *in vacuo*. Flash chromatography (applied in pet. ether; eluted 2:1 to 1:1 pet. ether/EtOAc) was carried out to give a mixture of the product and starting sulfonamide. Reverse phase preparative HPLC (35 to 55% MeCN/H_2_O over 10 min) was carried out to afford title compound (RT 15.5 min) as off white solid (56.7 mg, 0.123 mmol, 30%). Mpt: 108-110 °C; R*_f_* = 0.62 (1:1 CH_2_Cl_2_/EtOAc); IR (ν_max_/cm^-1^, thin film): 3112, 1624, 1542, 1506; ^1^H NMR (600 MHz, CD_3_OD): δ_H_ = 2.38 (s, 3H, **26-H**), 7.17 (d, *J* = 5.4 Hz, 1H, **6-H**), 7.28 (d, *J* = 9.0 Hz, 2H, **17-H**), 7.33 (d, *J* = 8.4 Hz, 2H, **24-H**), 7.48 (d, *J* = 9.0 Hz, 2H, **18-H**), 7.49, (dd, *J* = 5.4, 1.2 Hz, 1H, **11-H**), 7.72-7.73 (m, 3H, **12-H**, **23-H**), 7.92 (dd, *J* = 2.4, 1.2 Hz, 1H, **14-H**), 7.96 (bs, 1H, **2-H**), 8.02 (d, *J* = 5.4 Hz, 1H, **5-H**); ^1^H NMR (600 MHz, CDCl_3_): δ_H_ = 2.38 (s, 3H, **26-H**), 6.69 (s, 1H, **20-H**), 7.07 (d, *J* = 8.8 Hz, 2H, **18-H**), 7.22 (d, *J* = 8.2 Hz, 2H, **24-H**), 7.34 (dd, *J* = 4.0, 2.0 Hz, 1H, **11-H**), 7.52 (d, *J* = 4.7 Hz, 1H, **6-H**), 7.54-7.56 (m, 2H, **12,14-H**), 7.63 (d, *J* = 8.2 Hz, 2H, **23-H**), 7.64 (s, 1H, **2-H**), 7.71 (d, *J* = 4.7 Hz, 1H, **5-H**), 7.79 (d, *J* = 8.8 Hz, 2H, **17-H**), 8.34 (bs, 1H, **15-H**); ^13^C NMR (150 MHz, CD_3_OD): δ_C_ = 21.4 (**C-26**), 112.2 (**C-5**), 119.8 (**C-6**), 122.7 (**C-17**), 126.1 (**C-14**), 126.6 (**C-18**), 128.0 (**C-11**), 128.3 (**C-10**), 128.3 (**C-23**), 128.9 (**C-12**), 129.1 (**C-3**), 130.7 (**C-24**), 132.1 (**C-19**), 133.0 (**C-9**), 134.2 (**C-2**), 138.2 (**C-22**), 138.8 (**C-16**), 145.3 (**C-25**), 146.2 (**C-8**); ^13^C NMR (150 MHz, CDCl_3_): 21.7 (**C-26**), 109.7 (**C-5**), 120.3 (**C-17**), 123.6 (**C-12**), 123.9 (**C-18**), 124.5 (**C-10**), 126.9 (**C-11**), 127.4 (**C-14**), 127.5 (overlapping signals, **C-2, 23**), 128.1 (**C-3**), 129.1 (**C-6**), 129.8 (**C-24**), 131.2 (**C-19**), 132.9 (**C-9**), 136.2 (**C-22**), 137.4 (**C-16**), 143.9 (**C-25**), 146.1 (**C-8**); LRMS m/z (ES^+^): 484 [M+Na]^+^, 462 [M+H]^+^; HRMS m/z (ES^+^): Found 462.1065 [M+H]^+^; C_23_H_20_N_5_S_2_O_2_ requires 462.1058.

**4-methyl-*N*-[2-(2-naphthyl)imidazo[1,2-*a*]pyrazine-8-yl]benzenesulfonamide, 7**

##

All glassware was evacuated and flushed with argon prior to use. **21a** (50.0 mg, 0.178 mmol), 4-toluenesulfonamide **22** (36.9 mg, 0.215 mmol, 1.2 equiv.), NaO^t^Bu (24.1 mg, 0.250 mmol, 1.4 equiv.), 1 mol% Pd_2_(dba)_3_ (1.6 mg) and 3 mol% DavePhos (2.1 mg) were weighed into a 25 mL round bottom flask. Toluene (2 mL) was added and the reaction was stirred under reflux for 24 h. The reaction mixture was cooled to RT and the solvents removed in vacuo. The residue was taken up in CH_2_Cl_2_ (30 mL) and washed with water (3 x 30 mL). The combined aqueous extracts were washed with CH_2_Cl_2_ (30 mL). The CH_2_Cl_2_ layers were combined, washed with brine (30 mL), dried over MgSO_4_ and the solvents removed in vacuo to give an orange oil. The crude product was dry loaded onto a column and purified by flash chromatography (applied in pet. ether; eluted 4:1 to 1:1 pet. ether/EtOAc) to give the target compound as a white solid (68.7 mg, 0.166 mmol, 93%).

Alternative microwave procedure:

In a 2 mL microwave vial, Pd_2_(dba)_3_ (0.7 mg, 1 mol%), DavePhos (0.8 mg, 3 mol%) and NaO^t^Bu (9.6 mg, 0.100 mmol, 1.4 equiv.) were dissolved in anhydrous toluene (1 mL). **21a** (20 mg, 0.072 mmol) and **22** (14.7 mg, 0.086 mmol, 1.2 equiv.) were added and the reaction mixture was stirred at 160 °C for 10 min with a 20 bar pressure limit. The solvent was removed *in vacuo* and the crude mixture was purified *via* flash chromatography (applied pet. ether; eluted 4:1 to 1:2 pet. ether ) to give **7** as a white solid (13.6 mg, 46%).

Mpt: >200 ^o^C; R*_f_* = 0.32 (1:1 CH_2_Cl_2_/EtOAc); IR (ν_max_/cm^-1^, thin film): 3253; ^1^H NMR (600 MHz, DMSO-*d_6_*): δ_H_ = 2.37 (s, 3H, **26-H**), 7.16 (bd, *J* = 5.2 Hz, 1H, **6-H**), 7.39 (d, *J* = 8.2 Hz, 2H, **24-H**), 7.51 - 7.54 (m, 2H, **15,16-H**), 7.86 (bd, *J* = 5.2 Hz, 1H, **5-H**), 7.89 (d, *J* = 8.0 Hz, 2H, **23-H**), 7.92 (d, *J* = 7.7 Hz, 1H, **14-H**), 7.98 (d, *J* = 8.6 Hz, 1H, **12-H**), 8.01 - 8.05 (m, 2H, **11,17-H**), 8.52 (s, 1H, **19-H**), 8.59 (s, 1H, **3-H**), 11.69 (s, 1H, NH: **20-H**); ^13^C NMR (150 MHz, DMSO-*d_6_*): δ_C_ = 21.0 (**C-26**), 111.0 (**C-5**), 115.3 (**C-3**), 116.8 (**C-6**), 123.8 (**C-11**), 124.2 (**C-19**), 126.2 (**C-23**), 126.3 (**C15/16**), 126.6 (**C-15/16**), 127.7 (**C-14**), 128.3 (**C-17**), 128.4 (**C-12**), 129.5 (**C-24**), 130.0 (**C-10**), 132.8 (**C-13**), 133.2 (**C-18**), 135.6 (**C-9**), 140.0 (**C-22**), 142.7 (**C-25**), 144.5 (**C-8**), 145.3 (**C-2**); LRMS m/z (ESI^+^): 415 [M+H]^+^, (ESI^-^): 413 [M-H]^-^, HRMS m/z (ESI^+^): Found 415.1219 [M+H]^+^; C_23_H_19_N_4_O_2_S requires 415.1229.

**4-methyl-*N*-[2-(2-phenoxyphenyl)imidazo[1,2-*a*]pyrazine-8-yl]benzenesulfonamide, 8**

All glassware was evacuated and flushed with argon prior to use. **21b** (130 mg, 0.405 mmol), 4-toluenesulfonamide **22** (83.1 mg, 0.486 mmol), K_2_CO_3_ (67.1 mg, 0.127 mmol), Pd(dba)_2_ (1.30 mg) and 5 mol% *tert-*butyl XPhos (6.50 mg) were weighed into a 10 mL round bottom flask. *^t^*BuOH (3 mL) was added and the reaction was stirred under reflux for 48 h. The reaction mixture was cooled to RT, diluted with MeOH (100 mL) and filtered through Celite (pre-washed with MeOH). Flash chromatography was carried out (applied in CH_2_Cl_2_; eluted 100% CH_2_Cl_2_ to 40:1 to 30:1 to 10:1 to 1:1 CH_2_Cl_2_/EtOAc) to afford the title compound as a yellow solid (23.9 mg, 0.052 mmol 13%). Mpt: >200 ^o^C; R*_f_* = 0.18 (9:1 CH_2_Cl_2_/EtOAc); IR (ν_max_/cm^-1^, thin film): 3245, 1586; ^1^H NMR (600 MHz, CD_2_Cl_2_): δ_H_ = 2.43 (s, 3H, **27-H**), 6.91 (bs, 1H, **6-H**), 6.96 (d, *J* = 8.1 Hz, 1H, **14-H**), 7.08 (dd, *J* = 8.8, 0.7 Hz, 2H, **18-H**), 7.17 (t, *J* = 7.1 Hz, 1H, **20-H**), 7.27 (t, *J* = 7.3 Hz, 1H, **12-H**), 7.31 – 7.37 (m, 4H, **5,13,25-H**), 7.38 – 7.41 (m, 2H, **19-H**), 7.91 (bd, *J* = 5.7 Hz, 2H, **24-H**), 8.12 (s, 1H, **3-H**), 8.48 (dd, *J* = 7.8,1.7 Hz 1H, **11-H**), 11.41 (s, 1H, **21-H**); ^13^C NMR (150 MHz, CD_2_Cl_2_): δ_C_ = 21.3 (**C-27**), 110.2 (**C-5**), 115.0 (**C-6**), 117.7 (**C-3**), 118.8 (**C-18**), 118.9 (**C-14**), 123.7 (**C-20**), 123.8 (**C-10**), 123.9 (**C-12**), 126.2 (**C-24**), 129,1 (**C-11**), 129.5 (**C-15,25**), 130.0 (**C-19**), 134.7 (**C-9**), 139.4 (**C-23**), 142.1 (**C-2**), 143.5 (**C-26**), 145.5 (**C-8**), 154.3 (**C-15**), 156.5 (**C-17**); LRMS m/z (ESI^+^): 457 [M+H]^+^, HRMS m/z (ESI^-^): Found 455.1167 [M +H]^-^; C_25_H_19_N_4_O_3_S requires 455.1178.

***N*-[2-(3,4-dimethoxylphenyl)imidazo[1,2-*a*]pyrazine-8-yl]-4-methyl-benzene­sulfonamide, 9**

All glassware was evacuated and flushed with argon prior to use. **21c** (200 mg, 0.691 mmol), 4-toluenesulfonamide **22** (142 mg, 0.829 mmol), K_2_CO_3_ (115 mg, 0.829 mmol), 1 mol% Pd(dba)_2_ (2.00 mg) and 5 mol% *tert-*butyl XPhos (10.0 mg) were weighed into a 10 mL round bottom flask. *^t^*BuOH (4 mL) was added and the reaction was stirred under reflux for 48 h. The reaction mixture was cooled to RT, diluted with MeOH (100 mL) and filtered through Celite (pre-washed with MeOH). Flash chromatography was carried out (applied in CH_2_Cl_2_; eluted 100% CH_2_Cl_2_ to 50:1 to 1:9 CH_2_Cl_2_/EtOAc followed by CH_2_Cl_2_/10% MeOH) gave the title compound as a yellow sticky sold (5.2 mg, 0.012 mmol, 2%). Mpt: >200 ^o^C; R*_f_* = 0.26 (1:1 CH_2_Cl_2_/EtOAc); IR (ν_max_/cm^-1^, thin film): 3274, 3138, 1584; ^1^H NMR (600 MHz, DMSO-*d_6_*): δ_H_ = 2.36 (s, 3H, **24-H**), 3.78 (s, 3H, **16-H**), 3.83 (s, 3H, **17-H**), 7.02 (d, *J* = 8.2 Hz, 1H, **12-H**), 7.13 (bd, *J* = 4.9 Hz, 1H, **6-H**), 7.37 (d, *J* = 8.2 Hz, 1H, **22-H**), 7.45 (bs, 1H, **11-H**), 7.47 (bs, 1H, **15-H**), 7.81 (d, *J* = 5.5 Hz, 1H, **5-H**), 7.87 (d, *J* = 8.0 Hz, 2H, **21-H**), 8.41 (s, 1H, **3-H**), 11.63 (s, 1H, **18-H**); ^13^C NMR (150 MHz, DMSO-*d_6_*): δ_C_ = 21.0 (**C-24**), 55.5 (**C-16**), 55.6 (**C-17**), 108.9 (**C-15**), 111.0 (**C-5**), 111.9 (**C-12**), 114.3 (**C-3**), 116.6 (**C-6**) 118.2 (**C-11**), 125.3 (**C-10**), 126.1 (**C-21**), 129.5 (**C-22**), 135.0 (**C-9**), 139.8 (**C-20**), 142.7 (**C-23**), 144.4 (**C-8**), 145.6 (**C-2**), 149.0 (**C-14**), 149.1 (**C-13**); LRMS m/z (ES^+^): 423 [M+H]^+^; HRMS m/z (ES^+^): Found 423.1115 [M+H]^+^; C_21_H_21_N_4_O_4_S requires 423.1127.

***N*-[2-(3,5-dimethylphenyl)imidazo[1,2-*a*]pyrazine-8-yl]-4-methylbenzene­sulfonamide, 10**

A mixture of **21d** (100 mg, 0.388 mmol), 4-toluene sulfonamide **22** (79.8 mg, 0.466 mmol), Pd(dppf)Cl_2_ (6.30 mg, 2 mol%) and Cs_2_CO_3_ (152 mg, 0.466 mmol) in anhydrous toluene (3 mL) were stirred under reflux, under Ar. After 21 h, the reaction was cooled to RT, diluted with toluene (20 mL) and washed with H_2_O (10 mL) and brine (10 mL). Further extraction of the aqueous layers was carried out using CH_2_Cl_2_ (3 x 10 mL). The combined organics were dried (MgSO_4_), filtered and solvent removed *in vacuo*. Flash chromatography (applied in CH_2_Cl_2_; eluted 19:1 CH_2_Cl_2_/EtOAc) afforded the title compound as a yellow solid (43.4 mg, 0.111 mmol, 29%). Mpt: >200 °C; *R_f_* = 0.37 (2:1 CH_2_Cl_2_/EtOAc); IR (ν_max_/cm^-1^, thin film): 2928, 1595; ^1^H NMR (600 MHz, (CD_3_)_2_SO): δ_H_ = 2.31 (s, 6H, **14-H**), 2.36 (s, 3H, **21-H**), 6.97 (s, 1H, **13-H**), 7.13 (t, *J* = 5.4 Hz, 1H, **6-H**), 7.38 (d, *J* = 8.1 Hz, 2H, **19-H**), 7.54 (s, 2H, **11-H**), 7.81 (d, *J* = 5.4 Hz, 1H, **5-H**), 7.87 (d, *J* = 8.1 Hz, 2H, **18-H**), 8.42 (s, 1H, **3-H**), 11.63 (bd, *J* = 5.4 Hz, 1H, **7/15-H**); ^13^C NMR (150 MHz, (CD_3_)_2_SO): δ_C_ = 21.0 (overlapping signals, **C-14,21**), 111.0 (**C-5**), 114.8 (**C-3**), 116.7 (**C-6**), 123.3 (**C-11**), 126.1 (**C-18**), 129.5 (**C-19**), 129.8 (**C-13**), 132.4 (**C-10**), 135.2 (**C-9**), 137.9 (**C-12**), 139.9 (**C-17**), 142.6 (**C-20**), 144.5 (**C-8**), 145.5 (**C-2**); LRMS m/z (ES^+^): 393 [M+H]^+^, HRMS m/z (ES^-^): Found 391.1236 [M-H]^-^; C_21_H_19_N_4_O_2_ requires 391.1229.

**4-methyl-*N*-[4-[[2-(2-naphthyl)imidazo[1,2-*a*]pyrazine-8-yl]amino]phenyl]benzene­sulfonamide, 11**

In a 2 mL microwave vial, Pd_2_(dba)_3_ (0.7 mg, 1 mol%), DavePhos (0.8 mg, 3 mol%) and NaO^t^Bu (9.6 mg, 0.100 mmol, 1.4 equiv.) were dissolved in anhydrous toluene (1 mL). **21a** (20 mg, 0.072 mmol) and **23** (22.5 mg, 0.086 mmol, 1.2 equiv.) were added and the reaction mixture was stirred at 160 °C for 10 min with a 20 bar pressure limit. The solvent was removed *in vacuo* and the crude mixture was purified *via* flash chromatography (applied in toluene, eluted 3:1 toluene:EtOAc) to give **11** as a light brown solid (17 mg, 47%).

**4-methyl-*N*-[4-[[2-(3-thienyl)imidazo[1,2-*a*]pyrazine-8-yl]amino]phenyl]benzene­sulfonamide, 12**

All glassware was evacuated and flushed with argon prior to use. **21e** (128 mg, 0.540 mmol), *N*-(4-aminophenyl)-4-methylbenzenesulfonamide **23** (171 mg, 0.650 mmol), K_2_CO_3_ (90.1 mg, 0.652 mmol), 1 mol% Pd(dba)_2_ (3.12 mg) and 5 mol% *tert-*butyl XPhos (11.5 mg) were weighed into a 25 mL round bottom flask. *^t^*BuOH (5 mL) was added and the reaction was stirred under reflux for 40 h. The reaction mixture was cooled to RT, diluted with MeOH (100 mL) and filtered through Celite (pre-washed with MeOH). LCMS indicated the correct product mass was present, and flash chromatography (applied in pet. ether; eluted 1:1 pet. ether /EtOAc) was carried to give the title compound as a yellow solid (19.2 mg, 0.042 mmol, 8%). Mpt: 158-164 ^o^C; R*_f_* = 0.57 (1:1 CH_2_Cl_2_/Et_2_O); IR (ν_max_/cm^-1^, thin film): 1140; ^1^H NMR (600 MHz, CD_3_OD): δ_H_ = 2.41 (s, 3H, **26-H**), 7.17 (d, *J* = 4.5 Hz, 1H, **6-H**), 7.29 (d, *J* = 8.8 Hz, 2H, **17-H**), 7.35 (d, *J* = 8.0 Hz, 2H, **24-H**), 7.53 (d, *J* = 8.8, 2H, **18-H**), 7.57 (dd, *J* = 4.9, 2.9 Hz, 1H, **12-H**), 7.66 (dd, *J* = 4.9, 1.1 Hz, 1H, **11-H**), 7.75 (d, *J* = 8.4, 2 H, **23-H**), 7.96 (d, *J* = 5.4, 1H, **5-H**), 7.98 (dd, *J* = 1.1, 2.8 Hz, 1H, **14-H**), 8.30 (s, 1H, **3-H**); ^13^C NMR (150 MHz, CD_3_OD): δ_C_ = 20.0 (**C-26**), 112.6 (**C-5**), 114.3 (**C-3**), 118.8 (**C-6**), 121.4 (**C-17**), 122.3 (**C-14**), 122.6, 124.8 (**C-18**), 125.5 (**C-11**), 126.5 (**C-12**), 127.0 (**C-23**), 129.3, (**C-24**), 131.4 (**C-16**), 134.0 (**C-9 +C-10**), 136.8 (**C-19 + C-22**), 143.2 (**C-2**), 143.8 (**C-25**), 144.4 (**C-8**); LRMS m/z (ESI^+^) 462 [M+H]^+^; HRMS m/z (ESI^+^): Found 462.1047 [M+H]^+^; C_23_H_20_N_5_0_2_S_2_ requires 462.1058.

### **4-Methyl-N-(4-(2-(2-phenoxyphenyl)imidazo[1,2-a]pyrazin-8-ylamino)phenyl)­benzene­sulfonamide 13**

NaO^t^Bu (12.5 mg, 130 μmol), 1 mol% Pd_2_(dba)_3_ (1 mg, 1.1 μmol) and 3 mol% DavePhos (1 mg, 2.5 μmol) were weighed into a 25 mL round bottom flask and dissolved in anhydrous toluene (5 mL). **21b** (30 mg, 93.4 μmol) and *N*-(4-aminophenyl)-4-methylbenzenesulfonamide **23** (29 mg, 112 μmol) were added under argon atmosphere. The reaction was stirred under reflux for 24 h. The reaction was cooled to room temperature and the solvent removed *in vacuo*. The product was taken up in dichloromethane (2 mL) and filtered. The filtrate was washed with water (3 x 1 mL) and the combined aqueous extracts were further washed with dichloromethane (2 x 1 mL). The organic layers were combined, dried over MgSO_4_ and the solvent was removed *in vacuo*. flash chromatography (applied in pet. ether; eluted 3:1 pet. ether /EtOAc) was performed to give the product as a yellow, sticky solid (4.8 mg, 8.8 μmol, yield: 9%). Mpt: decomposed before melting; R_f_ = 0.14 (3:1 pet. ether/EtOAc); IR (v_max_/cm^-1^, thin film): 1489-1542; ^1^H NMR (600 MHz, CDCl_3_), δ (ppm): 2.38 (s, 3H, CH_3_: **32-H**), 6.36 (bs, 1H, NH: **21-H**), 6.96 (dd, *J* = 6.7, 2.3 Hz, 1H, CH: **14-H**), 7.06 (d, *J* = 7.5 Hz, 2H, CH: **18-H**), 7.07 (d, *J* = 8.6 Hz, 1H, CH: **24-H**), 7.14 (t, *J* = 7.2 Hz, 1H, CH: **20-H**), 7.22 (d, *J* = 8.3 Hz, 2H, CH: **30-H**), 7.21-7.31 (m, 2H, CH: **12-H**, CH: **13-H**), 7.35-7.38 (m, 3H, CH: **6-H**, CH: **19-H**), 7.48 (d, *J* = 4.5 Hz, 1H, CH: **5-H**), 7.62 (d, *J* = 8.2 Hz, 2H, CH: **29-H**), 7.79 (d, *J* = 8.3 Hz, 2H, CH: **23-H**), 8.05 (s, 1H, CH: **3-H**), 8.45 (dd, *J* = 6.2, 2.2 Hz, 1H, CH: **11-H**); ^13^C-NMR (125 MHz, CDCl_3_), δ (ppm): 21.7 **(C-32)**, 111.9 **(C-5)**, 115.0 **(C-3)**, 118.8 **(C-18)**, 119.4 **(C-14)**, 120.1 **(C-23)**, 123.7 **(C-20)**, 124.1 **(C-24)**, 124.2 **(C-12)**, 124.8 **(C-10)**, 127.4 **(C-29)**, 127.8 **(C-6)**, 129.1 **(C-11)**, 129.3 **(C-13)**, 129.7 **(C-30)**, 130.1 **(C-19)**, 130.8 **(C-25)**, 132.6 **(C-9)**, 136.1 **(C-28)**, 137.6 **(C-22)**, 140.0 **(C-2)**, 143.9 **(C-8)**, 145.9 **(C-31)**, 154.3 **(C-15)**; LRMS m/z (ES^+^): 550 [M(^37^Cl)+H]^+^, 549, 548 [M(^35^Cl)+H]^+^; HRMS m/z (ES^+^): Found 548.1733 [M+H]^+^; C_31_H_26_N_5_O_2_S requires 548.1756.

**Reaction conditions screen for the synthesis of 7**

*Method A*

All glassware was evacuated and flushed with Ar prior to use. **214a** (50.0 mg, 0.178 mmol), 4-toluene sulfonamide **22** (36.9 mg, 0.215 mmol), K_2_CO_3_ (29.7 mg, 0.215 mmol), Pd(dba)_2_ (1.03 mg, 1 mol%) and *^t^*Bu-XPhos (3.80 mg, 5 mol%) were taken up in *^t^*BuOH (3 mL) and the reaction was stirred under reflux for 24 h. The reaction mixture was cooled to RT, diluted with MeOH (100 mL) and filtered through Celite (pre-washed with MeOH). Flash chromatography (applied in CH_2_Cl_2_; eluted 5% to 10% to 20% EtOAc; or applied in pet. ether; eluted 20% to 33% to 50% to 66% EtOAc) afforded the target compound as a white solid (28.0 mg, 0.068 mmol, 38%).

*Method B (Microwave):*

In a 2 mL microwave vial, catalyst, ligand and base were dissolved in solvent. **21a** and 4-toluene sulfonamide **22** (1.2 eq) were added and the reaction mixture was stirred at the stated temperature and for the given time with a 20 bar pressure limit. The solvent was removed *in vacuo* and the crude mixture was purified *via* flash chromatography (applied in CH_2_Cl_2_; eluted 5% to 10% to 20% EtOAc; or applied in pet. ether; eluted 20% to 33% to 50% to 66% EtOAc) afforded the title compound.

*Method C:*

**21a**, 4-toluene sulfonamide **22** (2 eq) and base (in the case of NaH, the base was pre-activated by stirring in anhydrous hexanes and drying) were dissolved in solvent and stirred at the stated temperature for the given time. The reaction was cooled to RT and quenched with brine (in the case of NaH: with NH_4_Cl (Sat.Aq)) and extracted EtOAc (3x). The combined organic layers were washed with H_2_O (5x) and brine, dried (MgSO_4_), filtered and solvent removed. Flash chromatography (applied in CH_2_Cl_2_; eluted 5% to 10% to 20% EtOAc; or applied in pet. ether; eluted 20% to 33% to 50% to 66% EtOAc) afforded the title compound.

*Method D (Microwave):*

In a 2 mL microwave vial, **21a**, 4-toluene sulfonamide **22** (1.2 eq) and base were added and the reaction mixture was stirred at the stated temperature and for the given time, with a 20 bar pressure limit. The solvent was removed *in vacuo* and the crude mixture was purified *via* flash chromatography (applied in CH_2_Cl_2_; eluted 5% to 10% to 20% EtOAc; or applied in pet. ether; eluted 20% to 33% to 50% to 66% EtOAc) afforded the title compound.

*Method E:*

NaH was washed by stirring in anhydrous hexanes, syringing out the solvent and drying. Under Ar, the reaction solvent was added to the flask, followed by 4-toluene sulfonamide (2 eq) in solvent. The contents were then stirred at RT for 20 min before **24** in solvent was added dropwise, and the reaction was stirred at the stated temperature for the given time. The reaction was cooled to RT and quenched with NH_4_Cl (Sat. Aq) and extracted EtOAc (3x). The combined organic layers were washed with H_2_O (5x) and brine, dried (MgSO_4_), filtered and solvent removed. Flash chromatography (applied in pet. ether; eluted 20% to 33% to 50% to 66% EtOAc) afforded the title compound.

*Method F (Microwave):*

In a 2 mL microwave vial, **24**, 4-toluene sulfonamide (2 eq) and base were added and the reaction mixture was stirred at the stated temperature and for the given time with a 20 bar pressure limit. The solvent was removed *in vacuo* and the crude mixture was purified *via* flash chromatography (pet. ether; 20% to 33% to 50% to 66% EtOAc) to afford the title compound.

| **Entry** | **Method** | **Catalyst** | **Ligand** | **Base** | **Solvent** | **Temp/°C** | **Time/h** | **Yield/%** |
| --- | --- | --- | --- | --- | --- | --- | --- | --- |
| **1** | A | Pd(dba)_2_ (1 mol%) | Q-Phos  (2 mol%) | NaO*^t^*Bu  (1.5 eq) | toluene | 80 | 22 | 0 |
| **2** | A | Pd(dba)_2_ (1 mol%) | Q-Phos  (2 mol%) | NaO*^t^*Bu  (1.5 eq) | THF | 50 | 72 | 0 |
| **3** | A | Pd(dba)_2_ (1 mol%) | *^t^*Bu-XPhos  (5 mol%) | K_2_CO_3_  (1.2 eq) | THF | Reflux | 24 | 0 |
| **4** | A | Pd(dba)_2_ (1 mol%) | *^t^*Bu-XPhos  (5 mol%) | K_2_CO_3_  (1.2 eq) | *^t^*BuOH | Reflux | 24 | 38 |
| **5** | B | Pd(dba)_2_ (1 mol%) | *^t^*Bu-XPhos  (5 mol%) | K_2_CO_3_  (1.2 eq) | *^t^*BuOH | 120 | 30 min | 11 |
| **6** | A | Pd(dba)_2_ (1 mol%) | DavePhos  (3 mol%) | Cs_2_CO_3_  (1.4 eq) | *^t^*BuOH | Reflux | 24 | 5 |
| **7** | A | Pd(dba)_2_ (1 mol%) | DavePhos  (3 mol%) | Cs_2_CO_3_  (1.4 eq) | dioxane | Reflux | 24 | 66 |
| **8** | A | Pd(dba)_2_ (1 mol%) | DavePhos  (3 mol%) | Cs_2_CO_3_  (1.4 eq) | dioxane | 105^a^ | 24 | 17 |
| **9** | B | Pd(dba)_2_ (1 mol%) | DavePhos  (3 mol%) | Cs_2_CO_3_  (1.4 eq) | dioxane | 130 | 30 min | 1 |
| **10** | A | Pd(dba)_2_ (1 mol%) | DavePhos  (3 mol%) | Cs_2_CO_3_  (1.4 eq) | toluene | Reflux | 16 | 12 |
| **11** | A | Pd(dba)_2_ (1 mol%) | DavePhos  (3 mol%) | NaO*^t^*Bu  (1.4 eq) | toluene | Reflux | 24 | 67 |
| **12** | A | Pd_2_(dba)_3_ (1 mol%) | DavePhos  (3 mol%) | Cs_2_CO_3_  (1.4 eq) | dioxane | Reflux | 24 | 81 |
| **13** | A | Pd_2_(dba)_3_ (1 mol%) | DavePhos  (3 mol%) | NaO*^t^*Bu  (1.4 eq) | toluene | Reflux | 24 | 93 |
| **14** | B | Pd_2_(dba)_3_ (1 mol%) | DavePhos  (3 mol%) | NaO*^t^*Bu  (1.4 eq) | toluene | 160 | 10 min | 46 |
| **15** | A | Pd(OAc)_2_ (0.5 mol%) | DavePhos  (3 mol%) | NaO*^t^*Bu  (1.2 eq) | toluene | Reflux | 21 | 64 |
| **16** | A | Pd(OAc)_2_ (0.5 mol%) | DavePhos  (3 mol%) | NaO*^t^*Bu  (1.2 eq) | toluene | 120^a^ | 21 | 28 |
| **17** | B | Pd(OAc)_2_ (0.5 mol%) | DavePhos  (3 mol%) | NaO*^t^*Bu  (1.2 eq) | toluene | 95 | 30 | 0 |
| **18** | B | Pd(PPh_3_)_4_  (5 mol%) | - | NaO*^t^*Bu  (1.4 eq) | toluene | 160 | 10 min | 8 |
| **19** | B | Pd(dppf)Cl_2_ (5 mol%) |  | NaO*^t^*Bu  (1.4 eq) | toluene | 160 | 10 min | 38 |
| **20** | A | CuI  (15 mol%) | Diamine^b^  (30 mol%) | K_2_CO_3_  (2 eq) | DMF | 100 | 72 | 3 |
| **21** | C | - | - | DIPEA  (5 eq) | NMP | 140 | 24 | 0 |
| **22** | C^c^ | - | - | DIPEA  (3.8 eq) | dioxane | 100 | 21 | 0 |
| **23** | C | - | - | NaH  (2 eq) | DMSO | RT | 21 | 0 |
| **24** | C | - | - | NaH  (2 eq) | DMSO | 100 | 24 | 18 |
| **25** | C | - | - | NaH  (2 eq) | DMF | 100 | 24 | 22 |
| **26** | D | - | - | DIPEA  (1.5 eq) | MeCN | 160 | 10 | 0 |
| **27** | E | - | - | NaH  (2 eq) | DMSO | RT | 20 | 18 |
| **28** | E | - | - | NaH  (2 eq) | DMSO | 100 | 20 | 62 |
| **29** | E | - | - | NaH  (2 eq) | DMF | 100 | 20 | 72 |
| **30** | F | - | - | DIPEA  (2 eq) | MeCN | 160 | 10 | 0 |

Table S1: Reaction optimisation for the formation of 7. ^a^ Reaction carried out in sealed tube; ^b^ *N,N’*-dimethylcyclohexane-1,2-diamine; ^c^ 1.4 eq of 4-toluene sulfonamide used

**Second generation imidazo[1,2-*a*]pyrazine inhibitors, variants at 8-position.**

**2-(Naphthalen-2-yl)imidazo[1,2-*a*]pyrazin-8-amine (25)**

In a sealed tube, **24** (21.0 mg, 0.065 mmol) was dissolved in 2.0 M NH_3_/IPA (2 mL), and the mixture was heated at 100 °C for 16 h. Removal of solvent *in vacuo* followed by flash chromatography (applied in CH_2_Cl_2_; eluted 1% to 10% MeOH) afforded the title compound as a sticky yellow solid (3.40 mg, 0.013 mmol, 20%). Mpt: >200 °C; *R_f_* = 0.49 (20:1 CH_2_Cl_2_/MeOH); IR (ν_max_/cm^-1^, thin film): 3314, 2956-3055, 1620; ^1^H NMR (600 MHz, CDCl_3_): δ_H_ = 6.10 (bs, 2H, **20-H**), 7.29 (d, *J* = 4.7 Hz, 1H, **6-H**), 7.47-7.52 (m, 2H, **15,16-H**), 7.54 (d, *J* = 4.7 Hz, 1H, **5-H**), 7.85 (d, *J* = 8.4 Hz, 1H, **17-H**), 7.90 (d, *J* = 1.0 Hz, 2H, **12,14-H**), 7.92 (s, 1H, **3-H**), 8.00 (d, *J* = 8.5 Hz, 1H, **11-H**), 8.44 (s, 1H, **19-H**); ^13^C NMR (150 MHz, CDCl_3_): δ_C_ = 111.4 (**C-3**), 112.8 (**C-5**) 123.8 (**C-11**), 125.2 (**C-19**), 126.6 (**C-15**), 126.7 (**C-16**), 127.8 (signals overlapping, **C-6,17**), 128.4 (**C-12**), 128.8 (**C-14**), 129.4 (**C-10**), 133.5 (signals overlapping, **C-9,13,18**), 146.1(**C-8**), 148.4 (**C-2**); LRMS m/z (EI^+^): 260 [M]^+^; HRMS m/z (CI^+^): Found 261.1141 [M+H]^+^; C_16_H_13_N_4_ requires 261.1140.

***N*’-(2-(Naphthalen-2-yl)imidazo[1,2-*a*]pyrazin-8-yl)benzene-1,4-diamine (26)**

All glassware was dried and purged with Ar prior to use. Pd_2_(dba)_3_ (9.17 mg, 1 mol%), DavePhos (11.8 mg, 3 mol%) and NaO*^t^*Bu (135 mg, 1.402 mmol) were dissolved in anhydrous toluene (10 mL). **21a** (280 mg, 1.00 mmol) and 1,4-diaminobenzene (130 mg, 1.20 mmol) were added and the reaction was stirred under reflux under Ar for 16 h. The reaction was cooled to RT and solvent removed *in vacuo*, before the residue was taken up in CH_2_Cl­_2_ (100 mL) and washed with H_2_O (3 x 50 mL) and brine (30 mL), dried (MgSO_4_), filtered and concentrated *in vacuo*. Flash chromatography (applied in CH_2_Cl_2_; eluted 15:1 to 5:1 to 1:1) afforded the title compound as a sticky brown solid (95.0 mg, 0.271 mmol, 27%). *R_f_* = 0.22 (1:1 CH­_2_Cl_2_/EtOAc); IR (ν_max_/cm^-1^, thin film): 3335, 3051, 1622, 1541, 1507; ^1^H NMR (600 MHz, CDCl_3_): δ_H_ = 3.71 (bs, 2H, **25-H**), 6.76 (ap.d, *J* = 8.7 Hz, 2H, **23-H**), 7.42 (d, *J* = 4.6 Hz, 1H, **6-H**), 7.48-7.53 (m, 3H, **5,15,16-H**), 7.61 (d, *J* = 9.5 Hz, 2H, **22-H**), 7.86 (d, *J* = 7.5 Hz, 1H, **14-H**), 7.90 (s, 1H, **3-H**), 7.92-7.94 (m, 3H, **12,17,20-H**), 8.02 (dd, *J* = 8.5, 1.7 Hz, 1H, **11-H**), 8.48 (s, 1H, **19-H**); ^13^C NMR (150 MHz, CDCl_3_): δ_C_ = 110.8 (**C-5**), 111.0 (**C-3**), 115.8 (**C-23**), 122.4 (**C-22**), 124.1 (**C-11**), 124.9 (**C-19**), 126.3 (**C-15,16**), 127.9 (**C-14**), 128.4 (overlapping signals, **C-12,17**), 128.7 (**C-6**), 130.6 (overlapping signals, **C-10,21**), 133.4 (**C-13**), 133.7 (**C-9**), 133.8 (**C-18**), 142.7 (**C-24**), 144.7 (**C-2**), 146.8 (**C-8**); LRMS m/z (ES^+^): 352 [M+H]^+^, 338, 181; HRMS m/z (ES^+^): Found 352.1563 [M+H]^+^; C_22_H_18_N_5_ requires 352.1562.

***N*-(4-(2-(Naphthalen-2-yl)imidazo[1,2-*a*]pyrazin-8-ylamino)phenyl)methane­sulfon­amide (27)**

All glassware was dried and purged with Ar prior to use. Pd_2_(dba)_3_ (1.64 mg, 1 mol%), DavePhos (2.11 mg, 3 mol%) and NaO*^t^*Bu (24.1 mg, 0.250 mmol) were dissolved in anhydrous toluene. **21a** (50.0 mg, 0.179 mmol) and *N*-(4-aminophenyl)methanesulfonamide^7^ (39.9 mg, 0.215 mmol) were added and the reaction was stirred under reflux, under Ar for 20 h. The reaction was cooled to RT and the solvent removed *in vacuo*, before the residue was taken up in CH_2_Cl­_2_ (50 mL) and washed with NaHCO_3_ (30 mL), H_2_O (30 mL) and brine (30 mL), dried (MgSO_4_), filtered and concentrated *in vacuo*. Flash chromatography (applied in toluene; eluted 2:1 toluene/EtOAc) afforded the title compound as an off white solid (5.2 mg, 0.012 mmol, 7%). Mpt: Decomposed before melting; *R_f_* = 0.32 (1:1 toluene/EtOAc); IR (ν_max_/cm^-1^, thin film): 3248, 3056, 2926, 2854, 1624, 1543, 1508; ^1^H NMR (600 MHz, CDCl_3_): δ_H_ = 3.01 (s, 3H, **27-H**), 6.39 (s, 1H, **25-H**), 7.27-7.29 (m, 2H, **23-H**), 7.47 (d, *J* = 4.6 Hz, 1H, **6-H**), 7.49-7.54 (m, 2H, **15,16-H**), 7.60 (d, *J* = 4.6 Hz, 1H, **5-H**), 7.87 (d *J* = 7.8 Hz, 1H, **14-H**), 7.92-7.95 (m, 5H, **3,12,17,22-H**), 8.02 (dd, *J* = 8.5, 1.6 Hz, 1H, **11-H**), 8.18 (s, 1H, **20-H**), 8.48 (s, 1H, **19-H**); ^13^C NMR (150 MHz, CDCl_3_): δ_C_ = 39.3 (**C-27**), 111.2 (**C-3**), 111.9 (**C-5**), 128.2 (**C-6**), 120.8 (**C-22**), 123.3 (**C-23**), 124.1 (**C-11**), 125.0 (**C-19**), 126.4 (**C-15**), 126.6 (**C-16**), 127.9 (**C-14**), 128.2 (**C-17**), 128.8 (**C-12**), 130.4 (**C-10**), 131.1 (**C-24**), 133.4 (**C-13**), 133.7 (overlapping signals, **C-9,18**), 137.7 (**C-21**), 145.1 (**C-2**), 146.1 (**C-8**); LRMS m/z (ES^+^): 430 [M+H]^+^; HRMS m/z (ES^+^): Found 430.1324 [M+H]^+^; C_23_H_30_N_5_O_2_S requires 430.1338.

**4-Methyl-*N*-(4-(2-(naphthalen-2-yl)imidazo[1,2-*a*]pyrazin-8-yloxy)phenyl)benzene­sulfonamide (28)**

NaH (60% in mineral oil; 12.4 mg, 0.310 mmol) was washed by stirring in anhydrous hexanes (3 mL) for 20 min, removing the solvent using a syringe and drying the contents under high vacuum. DMF (0.5 mL) was added followed by *N*-(4-hydroxyphenyl)-4-methylbenzenesulfonamide^8^ (81.4 mg, 0.310 mmol) in DMF (1 mL) and the mixture was stirred at RT for 20 min. **24** (50.0 mg, 0.155 mmol) in DMF (1.5 mL) was added and the resulting deep red solution was heated at 100 °C under Ar for 16 h. The mixture was then diluted with EtOAc (50 mL) and washed with NH_4_Cl (sat. aq. 20 mL) and H_2_O (5 x 20 mL). The combined aqueous layers were then re-extracted with EtOAc (2 x 30 mL), followed by washing the combined organics with brine (30 mL), drying (MgSO_4_), filtering and concentrating *in vacuo*. The crude material was purified *via* flash chromatography (applied in toluene; eluted 25% EtOAc/toluene) to give the title compound as an off white-pink solid (36.4 mg, 0.072 mmol, 47%). Mpt: 128 °C; *R_f_* = 0.21 (2:1 toluene/EtOAc); IR (ν_max_/cm^-1^, thin film): 3568, 3049, 1488; ^1^H NMR (600 MHz, (CD_3_)_2_SO): δ_H_ = 2.39 (s, 3H, **31-H**), 7.15-7.17 (m, 2H, **23-H**), 7.21-7.13 (m, 2H, **22-H**), 7.33 (d, *J* = 4.6 Hz, 1H, **6-H**), 7.39 (d, *J* = 8.2 Hz, 2H, **29-H**), 7.52-7.56 (m, 2H, **15,16-H**), 7.70 (d, *J* = 8.2 Hz, 2H, **28-H**), 7.94 (d, *J* = 7.6 Hz, 1H, **17-H**), 8.01-8.04 (m, 2H, **12,14-H**), 8.15 (dd, *J* = 8.5, 1.6 Hz, 1H, **11-H**), 8.32 (d, *J* = 4.6 Hz, 1H, **5-H**), 8.61 (s, 1H, **19-H**), 8.74 (s, 1H, **3-H**), 10.34 (s, 1H, **25-H**); ^13^C NMR (150 MHz, (CD_3_)_2_SO): δ_C_ = 21.0 (**C-31**), 112.9 (**C-3**), 116.9 (**C-5**), 121.3 (**C-23**), 122.7 (**C-22**), 124.0 (**C-11**), 124.5 (**C-19**), 125.5 (**C-6**), 126.4 (**C-15**), 126.6 (**C-16**), 126.8 (**C-28**), 127.7 (**C-17**), 128.3 (**C-14**), 128.5 (**C-12**), 129.9 (**C-29**), 130.4 (**C-10**), 132.8 (**C-9**), 132.9 (**C-13**), 133.2 (**C-18**), 135.0 (**C-24**), 136.7 (**C-27**), 143.4 (**C-30**), 145.1 (**C-2**), 148.7 (**C-21**), 153.1 (**C-8**); LRMS m/z (ES^-^): 505 [M-H]^-^; HRMS m/z (ES^-^): Found 505.1323 [M+H]^+^; C_29_H_23_N_4_O_3_S requires 505.1334.

***N*-(4-Aminobenzyl)-2-(naphthalen-2-yl)imidazo[1,2-*a*]pyrazin-8-amine**

All glassware was dried and purged with Ar prior to use. Pd_2_(dba)_3_ (1.64 mg, 1 mol%), DavePhos (2.11 mg, 3 mol%) and NaO*^t^*Bu (24.1 mg, 0.250 mmol) were dissolved in anhydrous toluene (2 mL). **21a** (50.0 mg, 0.179 mmol) and 4-(aminomethyl)aniline (24.3 µL, 0.215 mmol) were added and the reaction was stirred under reflux, under Ar for 20 h. The reaction was cooled to RT and solvent removed *in vacuo*, before the residue was taken up in CH_2_Cl­_2_ (50 mL) and washed with NaHCO_3_ (sat. aq. 30 mL), H_2_O (30 mL) and brine (30 mL), dried (MgSO_4_), filtered and concentrated *in vacuo*. Flash chromatography (applied in pet. ether; eluted 4:1 to 3:1 to 2:1 to 1:1 to 1:3 pet. ether/EtOAc) afforded the title compound as a light yellow oil (20.1 mg, 0.055 mmol, 31%). *R_f_* = 0.52 (3:1 EtOAc/pet. ether); IR (ν_max_/cm^-1^, thin film): 3326, 1619, 1544, 1519; ^1^H NMR (600 MHz, CD_3_OD): δ_H_ = 4.58 (s, 2H, **21-H**), 6.71-6.74 (m, 2H, **24-H**), 7.19 (d, *J* = 8.4 Hz, 2H, **23-H**), 7.25 (d, *J* = 4.7 Hz, 1H, **6-H**), 7.44-7.49 (m, 2H, **15,16-H**), 7.65 (d, *J* = 4.7 Hz, 1H, **5-H**), 7.82-7.84 (m, 1H, **14-H**), 7.85-7.88 (m, 2H, **12,17-H**), 7.94 (dd, *J* = 8.5, 1.6 Hz, 1H, **11-H**), 8.16 (s, 1H, **3-H**), 8.34 (s, 1H, **19-H**); ^13^C NMR (150 MHz, CD_3_OD): δ_C_ = 45.5 (**C-21**), 111.5 (**C-5**), 113.1 (**C-3**), 116.7 (**C-24**), 124.9 (**C-11**), 125.5 (**C-19**), 127.2 (**C-15**), 127.5 (**C-16**), 128.7 (**C-14**), 129.1 (**C-6**), 129.2 (**C-17**), 129.2 (**C-22**), 129.5 (**C-12**), 129.9 (**C-23**), 131.7 (**C-10**), 134.6 (overlapping signals, **C-9,13**), 135.0 (**C-18**), 145.4 (**C-2**), 148.1 (**C-25**), 149.8 (**C-8**); LRMS m/z (ES^+^): 366 [M+H]^+^, 273 [M-aniline]^+^, 261 [M-CH_2_-aniline]^+^; HRMS m/z (ES^+^): Found 366.1716 [M+H]^+^; C_23_H_20_N_5_ requires 366.1719.

**4-Methyl-*N*-(4-((2-(naphthalen-2-yl)imidazo[1,2-*a*]pyrazin-8-ylamino)methyl)­phenyl)­­benzene­­sulfonamide (29)**

*N*-(4-Aminobenzyl)-2-(naphthalen-2-yl)imidazo[1,2-a]pyrazin-8-amine (18.0 mg, 0.049 mmol) was dissolved in anhydrous pyridine (1 mL) and the mixture was cooled on ice. 4-Methylbenzene-1-sulfonyl chloride (11.3 mg, 0.059 mmol) was added and the deep yellow/orange solution was stirred under Ar at RT for 16 h. The solvent was then removed *in vacuo* and the crude material was purified *via* flash chromatography (applied in CH_2_Cl_2_; eluted 10% to 20% EtOAc) to afford the title compound as pale green sticky solid (8.20 mg, 0.016 mmol, 32%). *R_f_* = 0.44 (20% EtOAc/CH_2_Cl_2_); IR (ν_max_/cm^-1^, thin film): 3240, 3050, 2923, 2823, 1544, 1509; ^1^H NMR (600 MHz, CDCl_3_): δ_H_ = 2.47 (s, 3H, **32-H**), 4.76 (bs, 2H, **21-H**), 6.49 (bs, 1H, **20-H**), 6.73 (bs, 1H, **26-H**), 7.03 (d, *J* = 8.5 Hz, 2H, **24-H**), 7.22 (d, *J* = 8.2 Hz, 2H, **30-H**), 7.29 (d, *J* = 8.5 Hz, 2H, **23-H**), 7.35 (d, *J* = 4.5 Hz, 1H, **6-H**), 7.46-7.51 (m, 3H, **5,15,16-H**), 7.64 (d, *J* = 8.2 Hz, 2H, **29-H**), 7.83-7.85 (m, 1H, **14-H**), 7.87-7.90 (m, 3H, **3,12,17-H**), 7.95 (dd, *J* = 8.6, 1.2 Hz, 1H, **11-H**), 8.40 (s, 1H, **19-H**); ^13^C NMR (150 MHz, CDCl_3_): δ_C_ = 21.7 (**C-32**), 44.2 (**C-21**), 110.3 (**C-5**), 111.0 (**C-3**), 122.0 (**C-24**), 124.0 (**C-11**), 124.8 (**C-19**), 126.3 (**C-15**), 126.6 (**C-16**), 127.4 (**C-29**), 127.9 (Overlapping signals, **C-12,17**), 128.7 (**C-6**), 129.0 (**C-23**), 129.8 (**C-30**), 130.5 (**C-10**), 133.3 (**C-13**), 133.6 (**C-9**), 133.7 (**C-18**), 135.6 (**C-22**), 135.8 (**C-25**), 136.2 (**C-28**), 144.0 (**C-31**), 144.6 (**C-2**), 148.8 (**C-8**); LRMS m/z (ES^‑^): 518 [M-H]^-^; HRMS m/z (ES^-^): Found 518.1658 [M-H]^-^; C_30_H_24_N_5_O_2_S requires 518.1651.

***N*-(3-aminopropyl)-4-methylbenzenesulfonamide**

1,3-Diaminopropane (2.0 g, 27 mmol) and triethylamine (5.5 g, 54 mmol) were dissolved in a 2/1 solvent mixture of CH2Cl2/THF (50 mL). The resulting solution was stirred at room temperature and 4-methylbenzenesulfonyl chloride (2.5 g, 13 mmol) was added portion-wise over 2 h. The reaction was quenched by addition of 1 M HCl (50 mL). The biphasic mixture was extracted with 1 M HCl (20 mL x 2). The combined aqueous layers were basified with 2 M NaOH (50 mL) and extracted with CH2Cl2 (3 x 50 mL). The organic layers were combined, dried (MgSO4), filtered and concentrated under reduced pressure to give a colourless solid (1.45 g, 49% yield) suitably pure for subsequent synthetic steps. Mpt: Decomposed before melting; R*f* = 0.25 (15% MeOH in CH2Cl2); IR (νmax/cm^-1^, thin film): 3359, 2673, 1598, 1492, 1318; 1H NMR (600 MHz, CDCl3): δH 1.58 (*app*. qt, *J* = 6.9 Hz, 2H, **9-H**), 2.43 (s, 3H, **CH3**), 2.62 (t, *J* = 6.2 Hz, 2H, **10-H**), 2.87 (t, *J* = 6.2 Hz, 2H, **8-H**), 7.38 (d, *J* = 8.2 Hz, 2H, **3-H and 5-H**), 7.72 (d, *J* = 8.2 Hz, 2H, **2-H and 6-H**); 13C NMR (150 MHz, CDCl3): δC = 21.6 (**CH3**), 30.8 (**C-9**), 41.1 (**C- 10**), 43.3 (**C-8**), 127.2 (**C-2 and C-6**), 129.8 (**C-3 and C-5**), 137.2 (**C-1**), 143.2 (**C-4**); HRMS m/z (CI+): Found 229.10124 [M+H]+; C10H17N2O2S requires 229.10107.

**4-Methyl-*N*-(3-(2-(naphthalen-2-yl)imidazo[1,2-*a*]pyrazin-8-ylamino)propyl)­benzene­sulfonamide (30)**

To a solution of *N*-(3-aminopropyl)-4-methylbenzenesulfonamide (73.1 mg, 0.322 mmol) in anhydrous DMF (2 mL) was added DIPEA (56.1 µL, 0.322 mmol) followed by **24** (52.0 mg, 0.161 mmol) and the reaction mixture was stirred at 100 °C for 16 h. Cooling to RT and removal of the solvent was followed by flash chromatography (applied in toluene; eluted 2:1 toluene/EtOAc), which yielded the title compound as a white solid (24.9 mg, 0.053 mmol, 33%). Mpt: Decomposed before melting; *R_f_* = 0.26 (3:1 EtOAc/pet. ether); IR (ν_max_/cm^-1^, thin film): 3023, 2971, 1740, 1548, 1370, 1217; ^1^H NMR (600 MHz, CDCl_3_): δ_H_ = 1.79-1.81 (m, 2H, **22-H**), 2.35 (s, 3H, **30-H**), 3.00 (bd, *J* = 5.4 Hz, 2H, **23-H**), 3.68 (q, *J* = 5.8 Hz, 2H, **21-H**), 6.27 (bs, 1H, **20-H**), 7.02 (bs, 1H, **24-H**), 7.21 (d, *J* = 8.2 Hz, 2H, **28-H**), 7.33 (d, *J* = 4.7 Hz, 1H, **6-H**), 7.43 (d, *J* = 4.6 Hz, 1H, **5-H**), 7.46-7.51 (m, 2H, **15,16-H**), 7.72 (d, *J* = 8.2 Hz, 2H, **27-H**), 7.84-7.85 (m, 1H, **14-H**), 7.86 (s, 1H, **3-H**), 7.88-7.90 (m, 2H, **12,17-H**), 7.95 (dd, *J* = 8.5, 1.6 Hz, 1H, **11-H**), 8.38 (s, 1H, **19-H**); ^13^C NMR (150 MHz, CDCl_3_): δ_C_ = 21.6 (**C-30**), 30.3 (**C-22**), 38.3 (**C-21**), 39.7 (**C-23**), 110.2 (**C-5**), 111.1 (**C-3**), 124.0 (**C-11**), 124.7 (**C-19**), 126.3 (**C-15**), 126.6 (**C-16**), 127.1 (**C-27**), 127.9 (**C-14**), 128.1 (**C-6**), 128.4 (**C-17**), 128.7 (**C-12**), 129.7 (**C-28**), 130.5 (**C-10**), 133.3 (**C-9,13**), 133.7 (**C-18**), 137.6 (**C-26**), 143.1 (**C-29**), 144.8 (**C-2**), 149.7 (**C-8**); LRMS m/z (ES^+^): 472 [M+H]^+^; HRMS m/z (EI^+^): Found 471.1726 [M]^+^; C_26_H_25_N_5_O_2_S requires 471.1724.

***N*-(2-Aminoethyl)-4-methylbenzenesulfonamide**

1,2-Diaminoethane (2.0 g, 33 mmol) and triethylamine (6.7 g, 75 mmol) were dissolved in a 2/1 solvent mixture of CH_2_Cl_2_/THF (50 mL). The resulting solution was stirred at room temperature and 4-methylbenzenesulfonyl chloride (3.2 g, 17 mmol) was added portion-wise over 2 h. The reaction was quenched by addition of 1 M HCl (50 mL). The biphasic mixture was extracted with 1 M HCl (20 mL x 2). The combined aqueous layers were basified with 2 M NaOH (50 mL) and extracted with CH_2_Cl_2_ (50 mL x 3). The organic layers were combined, dried (MgSO4), filtered and concentrated under reduced pressure to give a colourless solid (2.1 g, 59% yield) suitably pure for subsequent synthetic steps. Mpt: Decomposed before melting; R*f* = 0.22 (15% MeOH in CH_2_Cl_2_); IR (ν_max_/cm-1, thin film): 3360, 2644, 1598, 1493, 1317; 1H NMR (600 MHz, CD_3_OD): δH= 2.42 (s, 3H, **CH3**), 2.65 (t, *J* = 6.2 Hz, 2H, **8-H**), 2.89 (t, *J* = 6.2 Hz, 2H, **7-H**), 7.38 (d, *J* = 8.2 Hz, 2H, **3-H and 5-H**), 7.73 (d, *J* = 8.2 Hz, 2H, **2-H and 6-H**); 13C NMR (150 MHz, CD3OD): δC = 21.4 (**CH3**), 42.2 (**C-8**), 46.4 (**C-7**), 128.0 (**C-2 and C-6**), 130.8 (**C-3 andC-5**), 138.8 (**C-1**), 144.7 (**C-4**); HRMS m/z (EI+): Found 215.08564 [M+ H]+; C_9_H_15_O_2_N_3_S requires 215.08542.

**4-Methyl-*N*-(2-(2-(naphthalen-2-yl)imidazo[1,2-*a*]pyrazin-8-ylamino)ethyl­)­benzene­sulfonamide­ (31)**

To a solution of *N*-(2-aminoethyl)-4-methylbenzenesulfonamide (65.9 mg, 0.310 mmol) in anhydrous DMF (2 mL) was added DIPEA (53.9 µL, 0.310 mmol) followed by **24** (50.0 mg, 0.155 mmol) and the reaction mixture was stirred at 100 °C for 16 h. Cooling to RT and removal of the solvent was followed by flash chromatography (applied in toluene; eluted 2:1 toluene/EtOAc), which yielded the title compound as a white solid (15 mg, 0.033 mmol, 21%). Mpt: 158-160 °C; *R_f_* = 0.26 (3:1 EtOAc/pet. ether); IR (ν_max_/cm^‑1^, thin film): 3412, 3029, 2924, 2853, 1621, 1538; ^1^H NMR (600 MHz, CDCl_3_): δ_H_ = 2.24 (s, 3H, **29-H**), 3.32 (bd, *J* = 4.7 Hz, 2H, **22-H**), 3.70-3.73 (m, 2H, **21-H**), 6.32 (bs, 1H, **20-H**), 6.55 (bs, 1H, **23-H**), 7.10 (d, *J* = 8.1 Hz, 2H, **27-H**), 7.29 (d, *J* = 4.6 Hz, 1H, **6-H**), 7.45 (d, *J* = 4.6 Hz, 1H, **5-H**), 7.48-7.52 (m, 2H, **15,16-H**), 7.63 (d, *J* = 8.1 Hz, 2H, **26-H**), 7.85-7.86 (m, 2H, **3,14-H**), 7.88-7.91 (m, 2H, **12,17-H**), 7.94 (d, *J* = 8.4 Hz, 1H, **11-H**), 8.37 (s, 1H, **19-H**); ^1^H NMR (600 MHz, (CD_3_)_2_SO): δ_H_ = 2.30 (s, 3H, **29-H**), 3.03 (q, *J* = 6.3 Hz, 2H, **22-H**), 3.53 (q, *J* = 6.3 Hz, 2H, **21-H**), 7.23-7.24 (m, 3H, **6,27-H**), 7.45 (t, *J* = 5.9 Hz, 1H, **20-H**), 7.50-7.56 (m, 2H, **15,16-H**), 7.63 (d, *J* = 8.2 Hz, 2H, **26-H**), 7.67 (t, *J* = 5.5 Hz, 1H, **23-H**), 7.77 (d, *J* = 4.6 Hz, 1H, **5-H**), 7.94 (d, *J* = 7.9 Hz, 1H, **14-H**), 7.96 (d, *J* = 7.9 Hz, 1H, **17-H**), 8.00 (d, *J* = 8.6 Hz, 1H, **12-H**), 8.11 (dd, *J* = 8.6, 1.6 Hz, 1H, **11-H**), 8.46 (s, 1H, **3-H**), 8.52 (s, 1H, **19-H**); ^13^C NMR (150 MHz, CDCl_3_): δ_C_ = 21.6 (**C-29**), 40.7 (**C-21**), 45.0 (**C-22**), 110.6 (**C-5**), 111.0 (**C-3**), 124.0 (**C-11**), 124.8 (**C-19**), 126.4 (**C-15**), 126.6 (**C-16**), 127.0 (**C-26**), 127.9 (**C-6,14**), 128.4 (**C-17**), 128.7 (**C-12**), 129.5 (**C-27**), 130.4 (**C-10**), 133.2 (**C-9**), 133.4 (**C-13**), 133.7 (**C-18**), 137.0 (**C-25**), 143.3 (**C-28**), 144.9 (**C-2**), 149.5 (**C-8**); ^13^C NMR (150 MHz, (CD_3_)_2_SO): δ_C_ = 21.0 (**C-29**), 40.1 (**C-21**), 41.6 (**C-22**), 110.3 (**C-5**), 112.0 (**C-**3), 123.8 (**C-19**), 124.1 (**C-11**), 126.1 (**C-15**), 126.6 (**C-16**), 126.6 (**C-26**), 127.7 (**C-14**), 128.0 (**C-17**), 128.1 (**C-6**), 128.3 (**C-12**), 129.4 (**C-27**), 131.0 (**C-10**), 132.6 (**C-13**), 132.9 (**C-9**), 133.2 (**C-18**), 137.3 (**C-25**), 142.4 (**C-28**), 143.0 (**C-2**), 148.4 (**C-8**); LRMS m/z (ES^+^): 458 [M+H]^+^; HRMS m/z (EI^+^): Found 458.1656 [M+H]^+^; C_25_H_24_N_5_O_2_S requires 458.1651.

**2-(Naphthalen-2-yl)-*N*-(3-(pyridin-3-yl)phenyl)imidazo[1,2-*a*]pyrazin-8-amine (32)**

All glassware was dried and purged with Ar prior to use. Pd_2_(dba)_3_ (1.28 mg, 1 mol%), DavePhos (1.65 mg, 3 mol%) and NaO*^t^*Bu (18.8 mg, 0.195 mmol) were dissolved in anhydrous toluene (2 mL). **21a** (39.0 mg, 0.140 mmol) and 3-(pyridin-3-yl)aniline^9^ (28.5 mg, 0.168 mmol) were added and the reaction was stirred under reflux, under Ar for 16 h. The reaction was cooled to RT, and solvent removed *in vacuo*. The crude material was taken up in CH_2_Cl_2_ (50 mL) and washed H_2_O (30 mL) and NaHCO_3_ (sat. aq. 30 mL). The combined aqueous layers were then re-extracted with CH_2_Cl_2_ (2 x 30 mL) followed by washing the combined organics with brine (30 mL), drying (MgSO_4_), filtering and concentrating *in vacuo*. Purification by flash chromatography (applied in toluene; eluted 20% to 33% EtOAc) afforded the title compound as a sticky brown solid (18.9 mg, 0.046 mmol, 33%). *R_f_* = 0.26 (1:1 toluene/EtOAc); IR (ν_max_/cm^-1^, thin film): 2922, 1514; ^1^H NMR (600 MHz, CDCl_3_): δ_H_ = 7.32 (d, *J* = 7.8 Hz, 1H, **24-H**), 7.42 (dd, *J* = 7.8, 4.8 Hz, 1H, **31-H**), 7.49-7.53 (m, 4H, **6,15,16,25-H**), 7.61 (d, *J* = 4.6 Hz, 1H, **5-H**), 7.87 (d, *J* = 7.8 Hz, 1H, **14-H**), 7.92 (dd, *J* = 8.2, 1.6 Hz, 1H, **26-H**), 7.93-7.95 (m, 3H, **3,12,17-H**), 7.98 (dt, *J* = 7.8, 1.9 Hz, 1H, **32-H**), 8.03 (dd, *J* = 8.5, 1.6 Hz, 1H, **11-H**), 8.28 (bt, *J* = 1.6 Hz, 1H, **22-H**), 8.32 (bs, 1H, **20-H**), 8.49 (s, 1H, **19-H**), 8.62 (dd, *J* = 4.8, 1.4 Hz, 1H, **30-H**), 8.94 (d, *J* = 1.9 Hz, 1H, **28-H**); ^13^C NMR (150 MHz, CDCl_3_): δ_C_ =111.2 (**C-3**), 111.8 (**C-5**), 118.3 (**C-22**), 119.3 (**C-26**), 121.8 (**C-24**), 123.8 (**C-31**), 124.1 (**C-11**), 125.0 (**C-19**), 126.4 (**C-15**), 126.6 (**C-16**), 127.9 (**C-14**), 128.4 (**C-17**), 128.5 (**C-6**), 128.7 (**C-12**), 129.9 (**C-25**), 130.4 (**C-10**), 133.4 (**C-13**), 133.7 (**C-18**), 133.8 (**C-9**), 134.9 (**C-32**), 136.8 (**C-27**), 138.7 (**C-23**), 140.2 (**C-21**), 145.0 (**C-2**), 146.2 (**C-8**), 148.4 (**C-28**), 148.4 (**C-30**); LRMS m/z (ES^+^): 414 [M+H]^+^; HRMS m/z (ES^+^): Found 414.1706 [M+H]^+^; C_27_H_20_N_5_ requires 414.1719.

***N*-(4-((2-(Naphthalen-2-yl)imidazo[1,2-*a*]pyrazin-8-yl)amino)phenyl)quinoline-8-sulfonamide (33)**

To a stirred solution of **26** (10.0 mg, 0.029 mmol), quinoline-8-sulfonyl chloride (14.3 mg, 0.062 mmol, 2.2eq.) and DMAP (cat.) in anhydrous CH_2_Cl_2_ (2 mL), under N_2_, was added Et_3_N (23 μL, 0.165 mmol, 5.7 eq.) and the reaction stirred at room temperature for 16h. The crude reaction mixture was washed with H_2_O (5 mL) and sat. NaHCO_3_ (5 mL). The organic fractions were dried (MgSO_4_), concentrated and purified by column chromatography (applied in CH_2_Cl_2_; eluted 100% to 20% EtOAc/CH_2_Cl_2_) to give the product as a pale green oil (2.5 mg, 0.005 mmol, 16%). *R_f_* = 0.58 (1:1 EtOAc/CH_2_Cl_2_); ^1^H NMR (600 MHz, CDCl_3_): δ_H_ = 7.01 (d, *J* = 8.8 Hz, 2H, **23-H**), 7.35 (d, *J* = 4.6 Hz, 1H, **6-H**), 7.47-7.51 (m, 2H, **15,16-H**), 7.52 (d, *J* = 4.6 Hz, 1H, **5-H**), 7.58 (t, *J* = 7.6 Hz, 1H, **31-H**), 7.63-7.66 (m, 3H, **22,35-H**), 7.85 (d, *J* = 7.7 Hz, 1H, **14-H**), 7.89 (s, 1H, **3-H**), 7.90-7.92 (m, 2H, **12,17-H**), 7.95 (bs, 1H, **19-H**), 7.98 (dd, *J* = 1.6 and 8.5 Hz, 1H, **32-H**), 8.03 (dd, *J* = 1.3 and 8.2 Hz, 1H, **11-H**), 8.31 (dd, *J* = 1.7 and 8.4 Hz, 1H, **34-H**), 8.34 (dd, *J* = 1.3 and 7.4 Hz, 1H, **30-H**), 8.40 (bs, 1H, **H-27**), 8.43 (bs, 1H, **H-20**), 9.18 (dd, *J* = 1.7 and 4.3 Hz, 1H, **36-H**); ^13^C NMR (150 MHz, CDCl_3_): δ_C_ = 111.1, 111.7, 120.0, 122.5, 123.7, 124.0, 124.9, 126.0, 126.3, 126.6, 127.9-128.9 (overlap), 131.8, 132.1, 133.4, 133.7, 135.5, 137.2, 137.4, 143.3, 151.4; LRMS m/z (CI^+^): 542 [M]^+^; HRMS m/z (CI^+^): Found 542.15172 [M]^+^; C_31_H_22_N_6_O_2_S requires 542.15195.

***N*-(2-(Naphthalen-2-yl)imidazo[1,2-*a*]pyrazin-8-yl)benzene-1,3-diamine**

All glassware was dried and purged with Ar prior to use. Pd_2_(dba)_3_ (16.4 mg, 2 mol%), DavePhos (17.6 mg, 5 mol%) and NaO*^t^*Bu (172 mg, 1.78 mmol) were dissolved in anhydrous toluene (5 mL). **21a** (250 mg, 0.89 mmol) and 1,3-diaminobenzene (97 mg, 0.89 mmol) were added and the reaction was stirred at 120 ^o^C in a sealed tube for 16 h. The reaction was cooled to RT and solvent removed *in vacuo*, before the residue was taken up in CH_2_Cl­_2_ (25 mL) and washed with H_2_O (2 x 50 mL) and brine (50 mL), dried (MgSO_4_), filtered and concentrated *in vacuo*. Flash chromatography (applied in CH_2_Cl_2_; eluted 15:1 to 5:1 to 1:1 CH­_2_Cl_2_/EtOAc) afforded the title compound as a sticky brown oil (90 mg, 0.26 mmol, 29%). *R_f_* = 0.59 (1:1 CH­_2_Cl_2_/EtOAc); ^1^H NMR (600 MHz, CDCl_3_): δ_H_ = 3.76 (bs, 2H, **27-H**), 6.44 (app d, *J* = 7.6 Hz, 1H, **24-H**), 7.13-7.19 (m, 2H, **22, 24-H**), 7.46 (d, *J* = 4.6 Hz, 1H, **6-H**), 7.48-7.55 (m, 3H, **5, 15, 16-H**), 7.56 (d, *J* = 4.5 Hz, 1H, **26-H**), 7.86 (d, *J* = 7.7 Hz, 1H, **17-H**), 7.92-7.95 (m, 3H, **3, 12, 14-H**), 8.02 (dd, *J* = 1.6 Hz and 8.5 Hz, 1H, **11-H**), 8.06 (s, 1H, **20-H**), 8.48 (s, 1H, **19-H**); ^13^C NMR (150 MHz, CDCl_3_): δ_C_ = 106.3, 110.1, 110.1, 111.1, 111.4, 124.1, 124.9, 126.3, 126.6, 127.9, 128.4, 128.4, 128.7, 129.9, 130.6, 133.4, 133.8, 140.4, 144.9, 146.3, 147.3; LRMS m/z (CI^+^): 351 [M]^+^; HRMS m/z (CI^+^): Found 351.14749 [M]^+^; C_22_H_17_N_5_ requires 351.14785.

***N*-(3-((2-(Naphthalen-2-yl)imidazo[1,2-*a*]pyrazin-8-yl)amino)phenyl)quinoline-8-sulfonamide (34)**

To a stirred solution of *N*-(2-(naphthalen-2-yl)imidazo[1,2-*a*]pyrazin-8-yl)benzene-1,3-diamine (10.0 mg, 0.029 mmol), quinoline-8-sulfonyl chloride (14.3 mg, 0.062 mmol, 2.2 eq.) and DMAP (cat.) in anhydrous CH_2_Cl_2_ (2 mL), under N_2_, was added Et_3_N (23 μL, 0.165 mmol, 5.7 eq.) and the reaction stirred at room temperature for 16 h. The crude reaction mixture was washed with H_2_O (5 mL) and sat. NaHCO_3_ (5 mL), organic fractions dried (MgSO_4_), concentrated and purified by column chromatography (applied in CH_2_Cl_2_; eluted 100% to 20% EtOAc/CH_2_Cl_2_) to give the product as a pale green oil (3.4 mg, 0.006 mmol, 21%). *R_f_* = 0.68 (1:1 EtOAc/CH_2_Cl_2_); ^1^H NMR (600 MHz, CDCl_3_): 6.75 (d, *J* = 7.9 Hz, 2H, **24-H**), 7.12 (t, *J* = 8.5 Hz, 2H, **23-H**), 7.34 (d, *J* = 4.4 Hz, 1H, **6-H**), 7.49-7.54 (m, 2H, **15,16-H**), 7.55 (d, *J* = 4.5 Hz, 1H, **5-H**), 7.58 (t, *J* = 7.8 Hz, 1H, **31-H**), 7.63 (m, 3H, **22,26,35-H**), 7.87 (d, *J* = 7.9 Hz, 1H, **14-H**), 7.91 (s, 1H, **3-H**), 7.92-7.96 (m, 3H, **12,17,19-H**), 7.99-8.02 (m, 2H, **11,32-H**), 8.29 (d, *J* = 8.3 Hz, 1H, **34-H**), 8.40 (d, *J* = 7.3 Hz, 1H, **30-H**), 8.47 (s, 1H, **H-27**), 8.48 (s, 1H, **H-20**), 9.21 (dd, *J* = 1.4 and 4.2 Hz, 1H, **36-H**); ^13^C NMR (150 MHz, CDCl_3_): 111.1, 116.5, 122.4, 124.0, 125.9, 126.6, 127.9, 128.1, 128.5, 128.7, 128.9, 129.6, 132.1, 133.8, 135.4, 137.3, 137.8, 140.0, 143.3, 151.4; LRMS m/z (CI^+^): 542 [M]^+^; HRMS m/z (CI^+^): Found 542.15113 [M]^+^; C_31_H_22_N_6_O_2_S requires 542.15195.

**Second generation imidazo[1,2-*a*]pyrazine inhibitors, variants at 2- and 6-positions.**

**8-Chloroimidazo[1,2-*a*]pyrazine**

2-Amino-3-chloropyrazine (200 mg, 1.54 mmol) and NaHCO_3_ (162 mg, 1.93 mmol) were suspended in *^t^*BuOH. Chloroacetaldehyde (50% w/v in H_2_O; 200 µL, 1.54 mmol) was added and the reaction mixture was stirred under reflux for 40 h. The reaction was then cooled to RT and solvent removed *in vacuo*. The residual material was taken up in CH_2_Cl_2_ (100 mL) washed H_2_O (40 mL) and brine (40 mL), dried (MgSO_4_), filtered and concentrated *in vacuo*. Flash chromatography (applied in pet. ether; eluted 3:1 to 2:1 to 1:3 pet. ether/EtOAc) afforded the title compound as an off white solid (118 mg, 0.773 mmol, 50%). Mpt: Decomposed before melting; *R_f_* = 0.17 (2:1 EtOAc/pet. ether); IR (ν_max_/cm^-1^, thin film): 3144, 3105, 1432; ^1^H NMR (600 MHz, (CD_3_)_2_SO): δ_H_ = 7.73 (d, *J* = 4.5 Hz, 1H, **6-H**), 7.87 (d, *J* = 0.9 Hz, 1H, **2-H**), 8.28 (d, *J* = 0.9 Hz, 1H, **3-H**), 8.66 (d, *J* = 4.5 Hz, 1H, **5-H**); ^13^C NMR (150 MHz, (CD_3_)_2_SO): δ_C_ = 117.3 (**C-3**), 120.8 (**C-5**), 127.3 (**C-6**), 135.5 (**C-2**), 137.1 (**C-9**), 141.7 (**C-8**); LRMS m/z (CI^+^): 154 [M(^35^Cl)+H]^+^, 156 [M(^37^Cl)+H]^+^; HRMS m/z (CI^+^): Found 154.0166 [M(^35^Cl)+H]^+^; C_6_H_5_ClN_3_requires 154.0172.

***N*-(4-(Imidazo[1,2-*a*]pyrazin-8-ylamino)phenyl)-4-methylbenzenesulfonamide (35)**

All glassware was dried and purged with Ar prior to use. Pd_2_(dba)_3_ (2.97 mg, 1 mol%), DavePhos (3.83 mg, 3 mol%) and NaO*^t^*Bu (43.8 mg, 0.456 mmol) were dissolved in anhydrous toluene (3 mL). 8-Chloroimidazo[1,2-*a*]pyrazine (49.8 mg, 0.324 mmol) and *N*-(4-aminophenyl)-4-methylbenzenesulfonamide **23** (106 mg, 0.389 mmol) were added and the reaction was stirred under reflux, under Ar for 16 h. The reaction was cooled to RT and solvent removed *in vacuo*, before the residue was taken up in CH_2_Cl­_2_ (50 mL) and washed with H_2_O (3 x 30 mL) and brine (30 mL), dried (MgSO_4_), filtered and concentrated *in vacuo*. Flash chromatography (applied in pet. ether; eluted 2:1 pet. ether/EtOAc) afforded the title compound as a brown solid (69.7 mg, 0.184 mmol, 56%). Mpt: Decomposed before melting; *R_f_* = 0.30 (3:1 EtOAc/pet. ether); IR (ν_max_/cm^-1^, thin film): 3338, 3045, 1537, 1505; ^1^H NMR (600 MHz, CDCl_3_): δ_H_ = 2.37 (s, 3H, **21-H**), 6.88 (s, 1H, **15-H**), 7.06 (d, *J* = 8.8 Hz, 2H, **13-H**), 7.21 (d, *J* = 8.3 Hz, 2H, **19-H**), 7.45 (d, *J* = 4.6 Hz, 1H, **6-H**), 7.56-7.57 (m, 3H, **2,3,5-H**), 7.63 (d, *J* = 8.3 Hz, 2H, **18-H**), 7.76 (d, *J* = 8.8 Hz, 2H, **12-H**), 8.19 (s, 1H, **10-H**); ^13^C NMR (150 MHz, CDCl_3_): δ_C_ = 21.7 (**C-21**), 111.9 (**C-5**), 115.2 (**C-3**), 120.3 (**C-12**), 123.9 (**C-13**), 127.4 (**C-18**), 128.4 (**C-6**), 129.7 (**C-19**), 131.1 (**C-14**), 131.9 (**C-2**), 133.2 (**C-9**), 136.2 (**C-17**), 137.4 (**C-11**), 143.9 (**C-20**), 146.0 (**C-8**); LRMS m/z (ES^+^): 380 [M+H]^+^, 402 [M+Na]^+^; HRMS m/z (ES^+^): Found 380.1168 [M+H]^+^; C_19_H_18_N_5_O_2_S requires 380.1181.

**2-(8-Chloroimidazo[1,2-*a*]pyrazin-2-yl)quinoxaline**

2-Bromo-1-(quinoxalin-2-yl)ethanone^10^ (648 mg, 2.58 mmol), 2-amino-3-chloropyrazine (334 mg, 2.58 mmol) and NaHCO_3_ (271 mg, 3.23 mmol) in *^t^*BuOH (15 mL) were stirred under reflux for 40 h. The solvent was removed *in vacuo* and the resulting residual was taken up in CH_2_Cl_2_ (100 mL) and washed H_2_O (2 x 40 mL) and brine (40 mL), dried (MgSO_4_), filtered and solvent removed *in vacuo*. Flash chromatography (applied in pet. ether; eluted 3:1 to 1:1 to 1:3 pet. ether/EtOAc) afforded the title compound as an orange solid (342 mg, 1.21 mmol, 47%). Mpt: >200 °C; *R_f_* = 0.11 (1:1 pet. ether/EtOAc); IR (ν_max_/cm^-1^, thin film): 2924, 1675, 1495; ^1^H NMR (600 MHz, (CD_3_)_2_SO): δ_H_ = 7.84 (d, *J* = 4.5 Hz, 1H, **6-H**), 7.89-7.95 (m, 2H, **14,15-H**), 8.15-8.17 (m, 2H, **13,16-H**), 8.73 (d, *J* = 4.5 Hz, 1H, **5-H**), 9.08 (s, 1H, **3-H**), 9.71 (s, 1H, **19-H**); ^13^C NMR (150 MHz, (CD_3_)_2_SO): δ_C_ = 116.9 (**C-3**), 121.0 (**C-5**), 128.3 (**C-6**). 129.0 (**C-13**), 129.1 (**C-16**), 130.5 (**C-15**), 131.1 (**C-14**), 137.7 (**C-9**), 141.4 (**C-12**), 141.8 (**C-17**), 142.2 (**C-8**), 143.4 (**C-19**), 143.9 (**C-10**), 146.4 (**C-2**); LRMS m/z (CI): 282 [M(^35^Cl)+H]^+^, 284 [M(^37^Cl)+H]^+^; HRMS m/z (CI): Found 282.0552 [M(^35^Cl)+H]^+^; C_14_H_9_ClN_5_ requires 282.0547.

**4-Methyl-*N*-(4-(2-(quinoxalin-2-yl)imidazo[1,2-*a*]pyrazin-8-ylamino)phenyl)­benzene­sulfonamide (36)**

All glassware was dried and purged with Ar prior to use. Pd(dba)_2_ (2.04 mg, 1 mol%), DavePhos (4.19 mg, 3 mol%) and Cs_2_CO_3_ (162 mg, 0.497 mmol) were stirred in dissolved in 1,4-dioxane (5 mL). 2-(8-Chloroimidazo[1,2-*a*]pyrazin-2-yl)quinoxaline (100 mg, 0.355 mmol) and *N*-(4-aminophenyl)-4-methylbenzenesulfonamide **23** (112 mg, 0.426 mmol) were added and the reaction was stirred under reflux for 40 h. The reaction was cooled to RT and solvent removed *in vacuo*. The resulting residue was taken up in CH_2_Cl_2_ (60 mL) and washed with NaHCO_3_ (sat. aq. 40 mL), H_2_O (30 mL) and brine (30 mL), dried (MgSO_4_), filtered and solvent removed *in vacuo*. Flash chromatography (applied in toluene; eluted 0% to 50% EtOAc) afforded the title compound as a light yellow solid (12.3 mg, 0.024 mmol, 7%). Mpt: Decomposed before melting; *R_f_* = 0.49 (1:1 CH_2_Cl_2_/EtOAc); IR (ν_max_/cm^-1^, thin film): 3135, 3061, 1495; ^1^H NMR (600 MHz, (CD_3_)_2_SO): δ_H_ = 2.34 (s, 3H, **31-H**), 7.05 (d, *J* = 8.9 Hz, 2H, **23-H**), 7.35 (d, *J* = 8.3 Hz, 2H, **29-H**), 7.44 (d, *J* = 4.6 Hz, 1H, **6-H**), 7.64 (d, *J* = 8.3 Hz, 2H, **28-H**), 7.84-7.86 (m, 1H, **15-H**), 7.88-7.90 (m, 3H, **14,22-H**), 8.03 (d, *J* = 4.6 Hz, 1H, **5-H**), 8.10-8.14 (m, 2H, **13,16-H**), 8.80 (s, 1H, **3-H**), 9.61 (s, 1H, **20-H**), 9.78 (s, 1H, **19-H**), 10.06 (bs, 1H, **25-H**); ^13^C NMR (150 MHz, (CD_3_)_2_SO): δ_C_ = 21.0 (**C-31**), 112.5 (**C-5**), 115.9 (**C-3**), 121.1 (**C-23**), 121.2 (**C-22**), 126.8 (**C-28**), 128.2 (**C-6**), 128.8 (**C-13**), 129.1 (**C-16**), 129.7 (**C-29**), 129.9 (**C-15**), 130.9 (**C-14**), 132.1 (**C-24**), 133.4 (**C-9**), 136.6 (**C-21**), 136.7 (**C-27**), 141.2 (**C-10**), 141.4 (**C-17**), 141.5 (**C-12**), 143.1 (**C-30**), 143.7 (**C-19**), 146.1 (**C-8**), 147.0 (**C-2**) ; LRMS m/z (ES^+^): 530 [M+Na]^+^, 508 [M+H]^+^; HRMS m/z (ES^+^): Found 508.1565 [M+H]^+^; C_27_H_22_N_7_O_2_S requires 508.1556.

**3,5-Dibromopyrazin-2-amine**

Pyrazin-2-amine (2.0g, 21 mmol) was dissolved in dichloromethane (50 mL) and the resulting solution was stirred at room temperature. *N*-bromosuccinimide (9.4g, 53mmol) was added. After completion, the mixture was concentrated under reduced pressure to give brown solid crude which was subsequently purified by chromatography (applied in hexane; eluted 10% EtOAc/hexane) to give the title compound as a pale yellow solid (3.6g 14 mmol, 68%). Mpt: 106-108 ^o^C; R*f* = 0.80 (1:1 EtOAc/hexane); IR (ν_max_/cm^-1^, thin film): 3447, 3280, 3154, 1621, 1549, 1506, 1450; ^1^H NMR (600 MHz, CDCl_3_): δ_H_ = 5.04 (brs, 2H, NH_2_), 8.04 (s, 1H, 6-H); ^13^C NMR (150 MHz, CDCl_3_): δ_C_ = 123.8 (C-5), 124.0 (C-3), 143.3 (C-6), 152.0 (C-2); HRMS m/z (CI^+^): found 251.87731 [M+H]^+^, C_4_H_4_Br_2_N_3_ requires 251.87720.

**6,8-Dibromo-2-(naphthalene-2-yl)imidazo[1,2-*a*]pyrazine (39)**

3,5-Dibromopyrazin-2-amine (200 mg, 0.79 mmol) and 2-bromo-1-(naphthalene-2‑yl)ethanone (490 mg, 2.0 mmol) were dissolved in ethanol (50 mL). A drop of concentrated HCl was added and the mixture was refluxed for 3 days at 80 ^o^C. After completion, the mixture was allowed to reach room temperature and concentrated under reduced pressure. The resulting crude was subsequently purified by chromatography (applied in hexane; eluted 15% EtOAc/hexane) to give the title compound (80 mg, 0.19 mmol, 25%). R*_f_* = 0.25 (1/3 EtOAc/hexane); IR (ν_max_/cm^-1^, thin film): 3135, 1622, 1592, 1505, 1481, 1447; ^1^H NMR (600 MHz, CDCl_3_): δ_H_= 7.52-7.56 (m, 2H, 15-H and 16-H), 7.85-7.88 (m, 1H, 14-H), 7.91-7.96 (m, 2H, 12-H and 17-H), 8.02-8.05 (m, 1H, 11-H), 8.13 (s, 1H, 3-H), 8.26 (s, 1H, 5-H), 8.54 (s, 1H, 19-H); ^13^C NMR (150 MHz, CDCl_3_): δ_C_ = 111.9, 118.9, 120.3, 123.2, 124.1, 126.3, 126.8, 126.9, 127.9, 128.7, 128.8, 133.5, 133.9, 138.8, 149.6, 152.0; HRMS m/z (EI+): Found 400.91624 [M]+; C_16_H_9_Br_2_N_3_ requires 400.91577;

***N*-(4-((6-Bromo-2-(naphthalen-2-yl)imidazo[1,2-*a*]pyrazin-8-yl)amino)phenyl)-4- methylbenzenesulfonamide (37)**

**23** (30 mg, 114 μmol) and *N*,*N*-diisopropylethylamine (31 μL, 178 μmol) were dissolved in n-butanol (10 mL) and the reaction was stirred at 120 ^o^C for 14 h. After completion, the reaction mixture was concentrated under reduced pressure and purified by normal phase chromatography with a 25% EtOAc in hexane eluent. 34 mg (58 μmol, 51% yield) of the product was isolated. R*f* = 0.45 (2:1 EtOAc/hexane); 1H NMR (600 MHz, CDCl_3_): δ_H_ = 2.39 (s, 3H, 24-H), 7.10 (d, *J* = 8.4 Hz, 2H, 13-H and 15-H), 7.24 (d, *J* = 8.2 Hz, 2H, 20-H and 22-H), 7.48‒7.56 (m, 2H, 30-H and 31-H), 7.63 (d, *J* = 8.2 Hz, 2H, 19-H and 23-H), 7.74 (s, 1H, 3-H), 7.81 (d, *J* = 8.4 Hz, 2H, 12-H and 16-H), 7.87 (d, *J* = 8.4 Hz, 1H, 29-H), 7.88 (s, 1H, 5-H), 7.81 (d, *J* = 8.4 Hz, 2H, 27-H and 32-H), 7.98 (dd, *J* = 1.5 and 8.5 Hz, 1H, 26-H), 8.44 (s, 1H, 34-H); 13C NMR (150 MHz, CDCl_3_): δ_C_ = 21.6, 111.1, 111.3, 120.0, 120.1, 121.8, 123.9, 124.0, 125.1, 126.5, 126.7, 127.4, 127.9, 128.4, 128.8, 129.8,130.0, 131.5, 132.4, 133.5, 133.7, 134.8, 136.1, 143.9, 144.0; HRMS m/z (CI+): 584.07632 [M+H]+; C_29_H_23_N_5_BrO_2_N_5_S requires 584.07558.

***N*-(2-((6-Bromo-2-(naphthalen-2-yl)imidazo[1,2-*a*]pyrazin-8-yl)amino)ethyl)-4- methylbenzenesulfonamide (38)**

6,8-Dibromo-2-(naphthalene-2-yl)imidazo[1,2-*a*]pyrazine (30 mg, 74 μmol), *N*-(2-aminoethyl)-4-methylbenzenesulfonamide (23 mg, 108 μmol) and *N*,*N*-diisopropylethylamine (31 μL, 178 μmol) were dissolved in n-butanol (10 mL) and the reaction was stirred at 80 ^o^C for 14 h. After completion, the reaction mixture was concentrated under reduced pressure and purified by chromatography (applied in hexane; eluted 25% EtOAc/ hexane) to give the title compound (33 mg, 62 μmol, 83%). R*f* = 0.50 (2:1 EtOAc/hexane); IR (ν_max_/cm-1, thin film): 3287, 3107, 2868, 1600, 1545; ^1^H NMR (600 MHz, CDCl_3_): δ_H_= 2.30 (s, 3H, **20-H**), 3.34 (q, *J* = 5.5 Hz, 2H, **12-H**), 3.71 (q, *J* = 5.5 Hz, 2H, **11-H**), 5.71 (t, *J* = 5.3 Hz, 1H, **10-H**), 6.39 (m, 1H, **13-H**), 7.18 (d, *J* = 8.0 Hz, 2H, **16-H** and **18-H**), 7.47-7.53 (m, 2H, **25-H** and **26-H**), 7.60 (s, 1H, **3-H**), 7.73 (d, *J* = 8.2 Hz, 2H, **15-H** and **19-H**), 7.81 (s, 1H, **5-H**), 7.85-7.95 (m, 4H, **24-H, 27-H, 29- H** and **30-H),** 8.37 (s, 1H, **22-H**); ^13^C NMR (150 MHz, CDCl_3_): δ_C_ = 21.6, 29.8, 44.1, 109.8, 110.9, 122.2, 123.9, 124.9, 126.5, 126.6, 126.7, 127.1, 128.4, 128.8, 129.6, 129.9, 132.0, 133.5, 133.7, 137.0, 143.4, 145.0, 147.8; HRMS m/z (EI+): Found 536.0757 [M+H]+; C_25_H_23_BrN_5_O_2_S requires 536.0756.

5-H

3-H


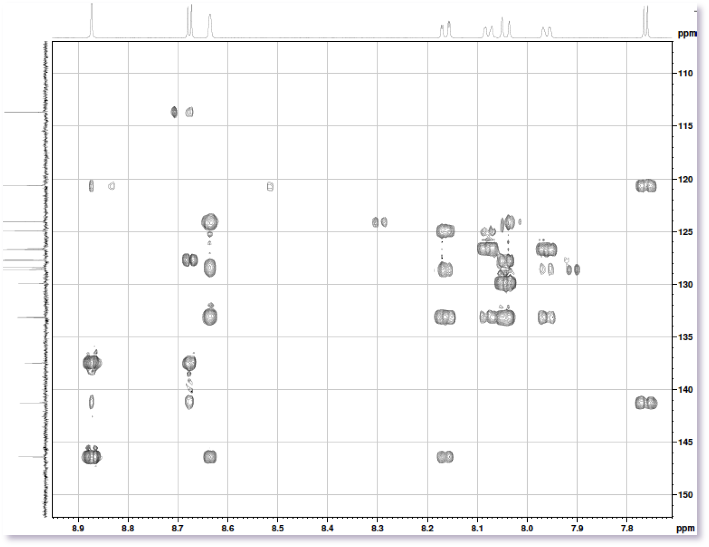


C-3

C-5

**Figure S1** HMBC spectrum of **21a**, indicating coupling of H_3_ with C_5_ and H_5_ with C_3_

Molecular docking studies were carried out using AutoDock Vina.^11^ The crystal structure of ADP-HP0525 was retrieved from RSCB Protein Data Bank (PDB entry: IG60). All heteroatoms (H_2_O, PEG, ADP) were removed from the .pdb file, which was then converted to pdbqt format “*enzyme*.pdbqt” using AutoDock Tools (ADT)^12^ with the addition of polar hydrogens and Kollman charges. The docking grid box (located at the nucleotide binding site) had a size of 14 x 16 x 24 with a grid center of -12.034, 24.627 and 22.363 in the *x*, *y*, and *z* coordinates respectively. All ligand structures were generated in mol2 format (“*ligand*.mol2”) using chem3D professional Cambridge software (no energy minimization was carried out) and were converted to “*ligand*.pdbqt” format using ADT. The docking file, “*ligand*.txt” was prepared containing receptor and ligand information (in .pdbqt format), output file “*out_ligand*.pdbqt” and docking grid box coordinates and size. An “exhaustiveness” parameter was set to a value of 8. The window command prompt was used to script the docking.

**
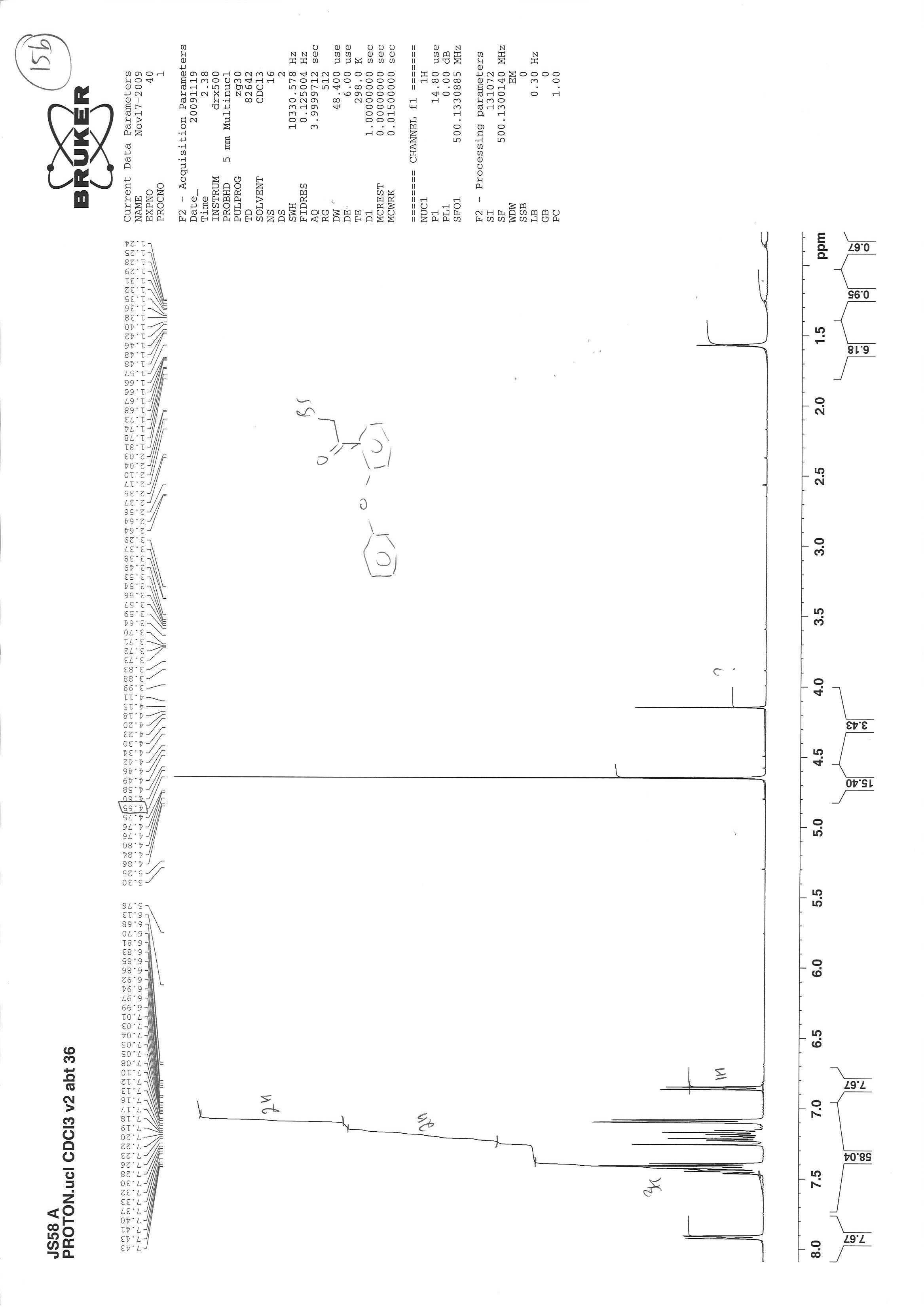
2-Bromo-1-(2-phenoxy­phenyl)ethanone 15b**

**
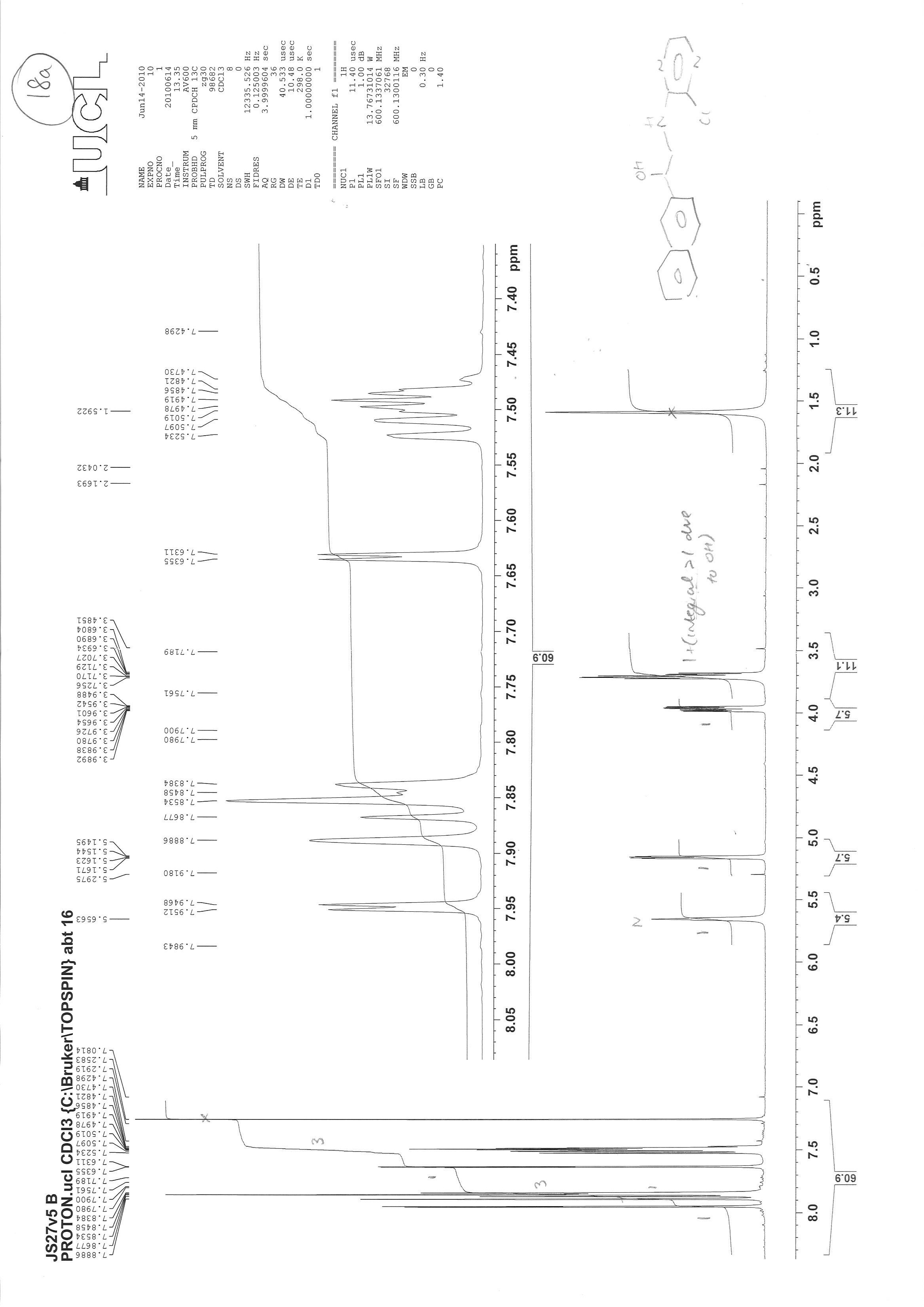
**

**2-[(3-Chloropyrazin-2-yl)amino]-1-(2-naphthyl)ethanol 18a.**

**
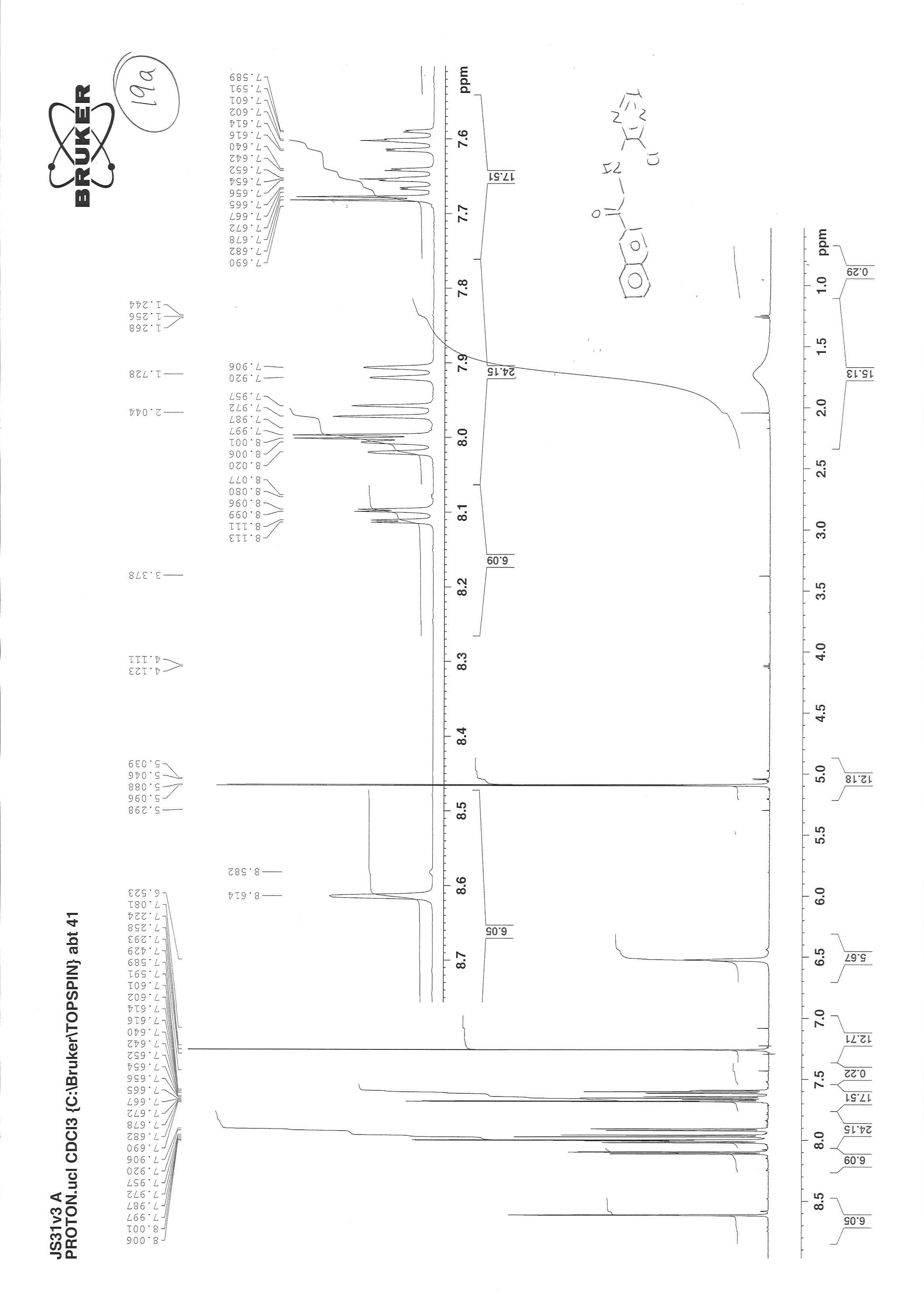
**

**2-[(3-Chloropyrazin-2-yl)amino]-1-(2-naphthyl)ethanone 19a.**

**
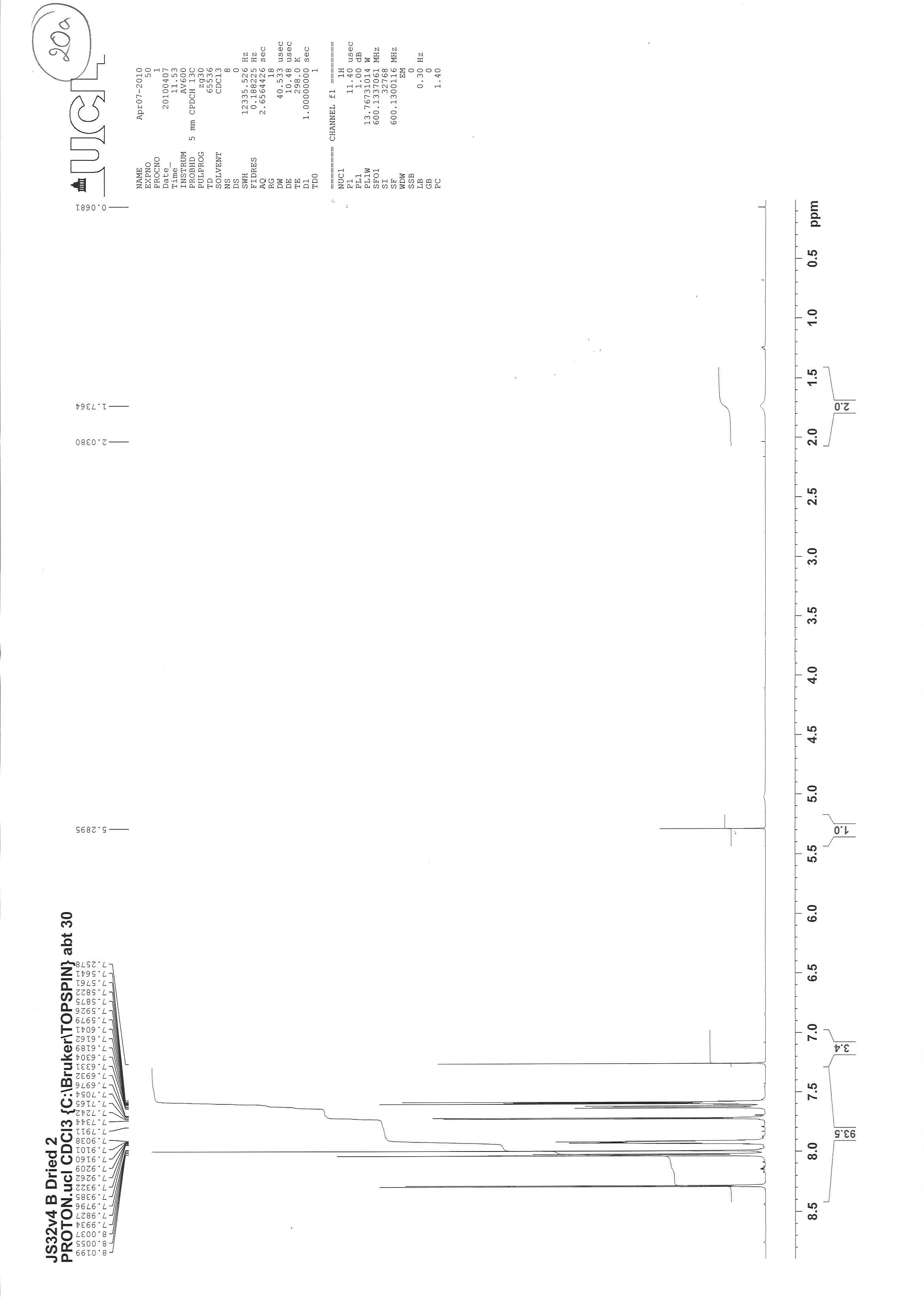
**

**8-Chloro-3-(2-naphthyl)imidazo[1,2-*a*]pyrazine 20a.**

**
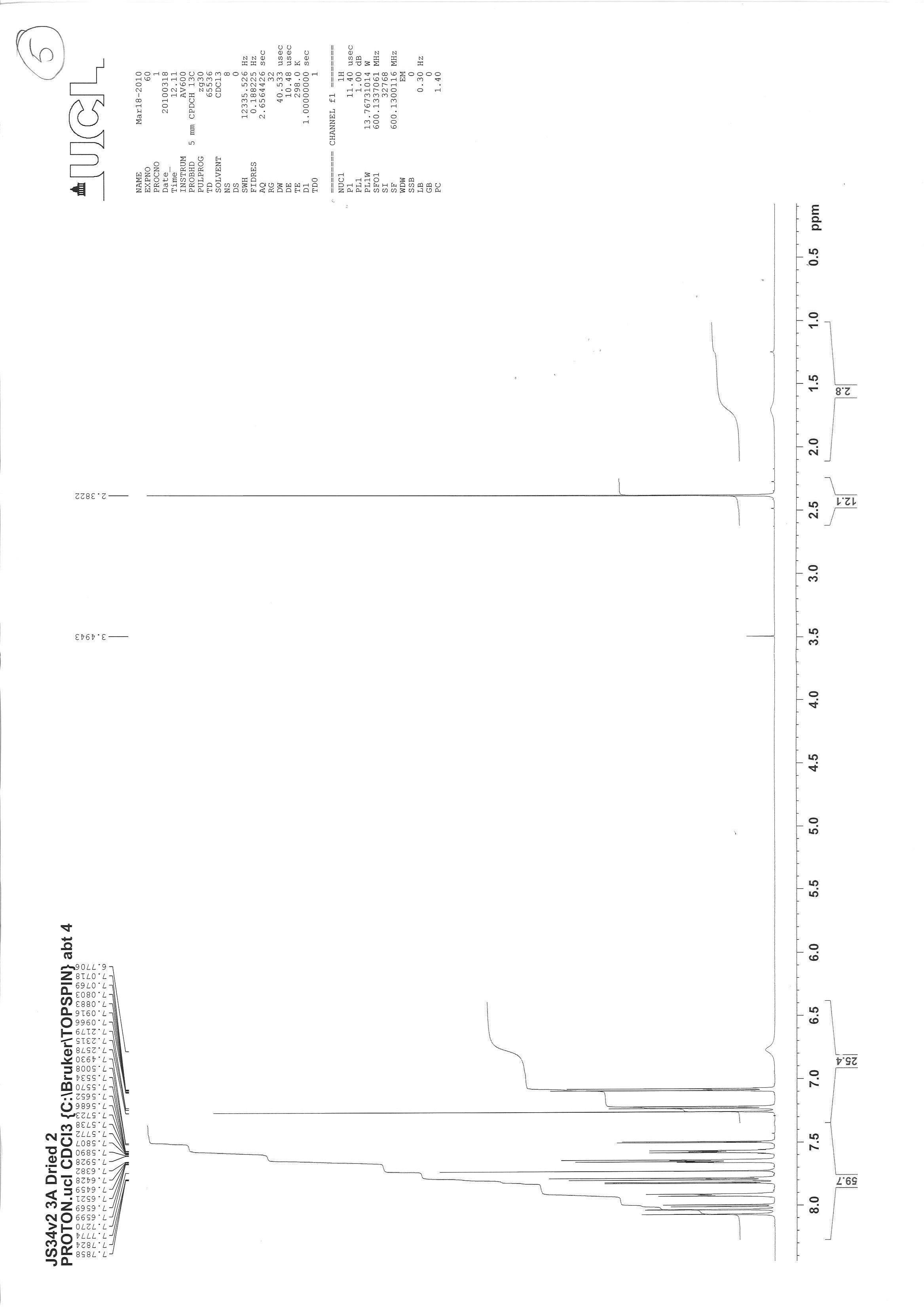
4-Methyl-*N*-[4-[3-(2-naphthyl)imidazo[1,2-*a*]pyrazine-8-yl]aminophenyl]­benzene­sulfonamide 5.**

**
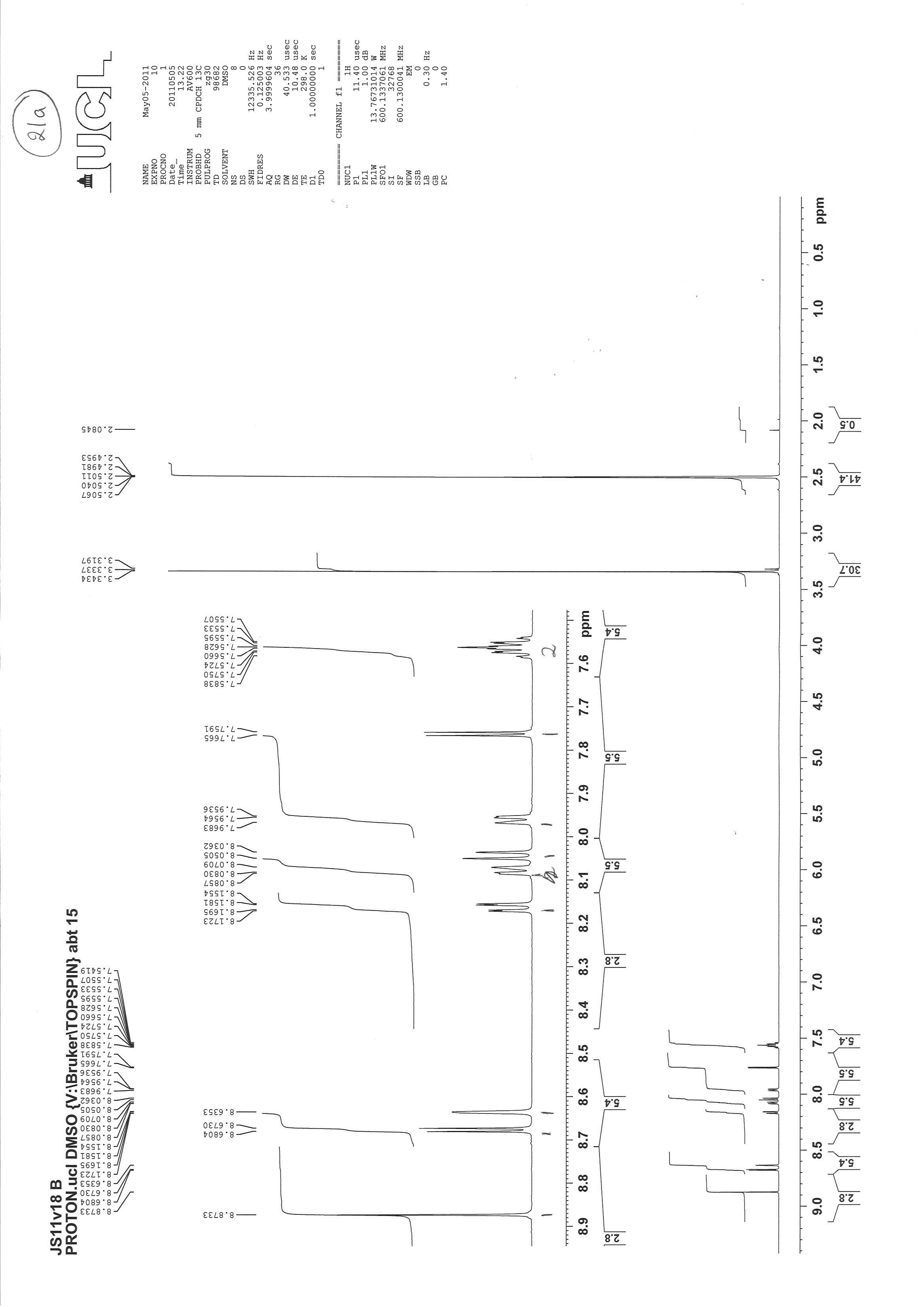
**

**8-Chloro-2-(2-naphthyl)imidazo[1,2-a]pyrazine 21a.**

**
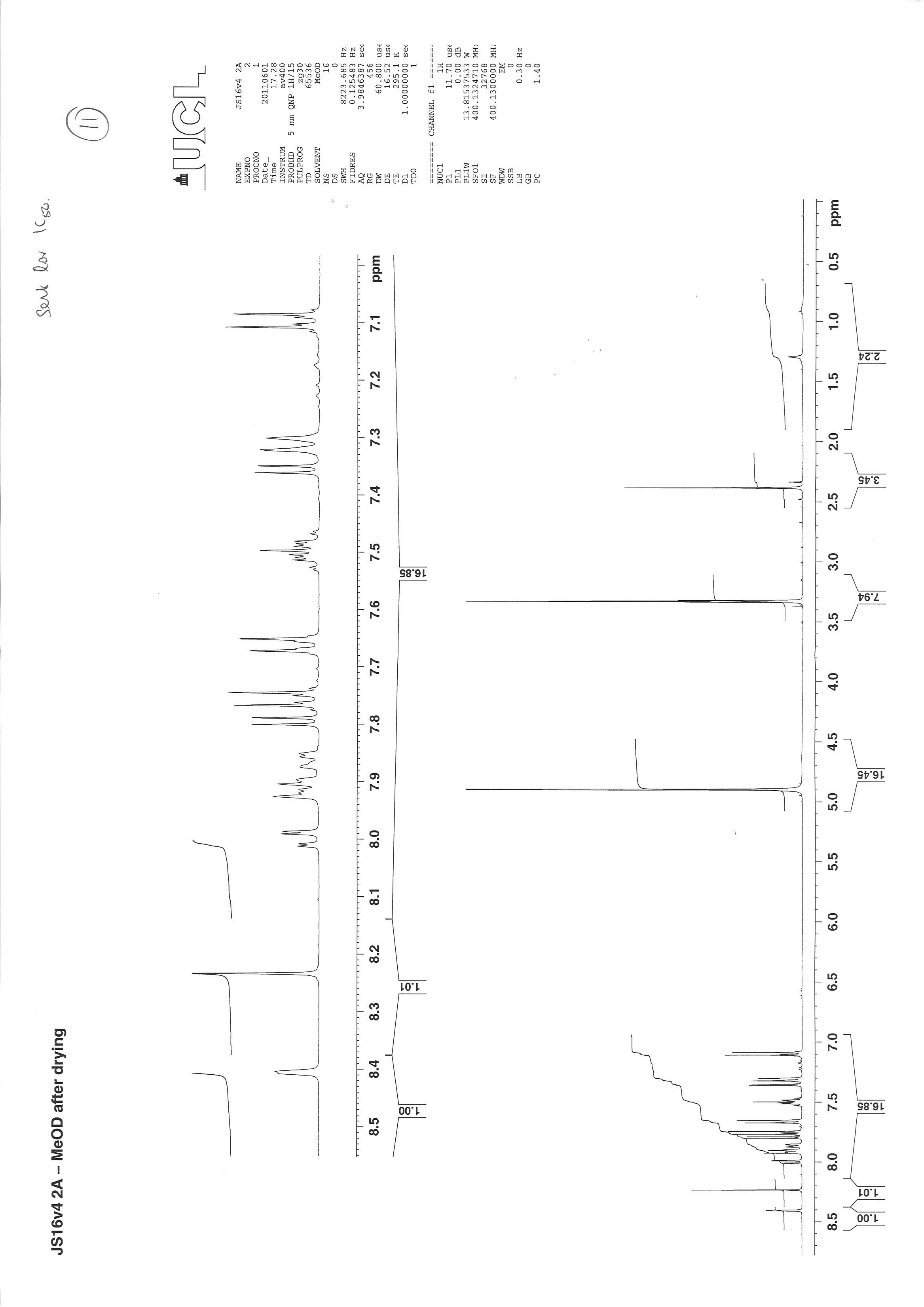
4-Methyl-*N*-[4-[2-(2-naphthyl)imidazo[1,2-*a*]pyrazine-8-yl]aminophenyl]­benzene­sulfonamide 11.**

**
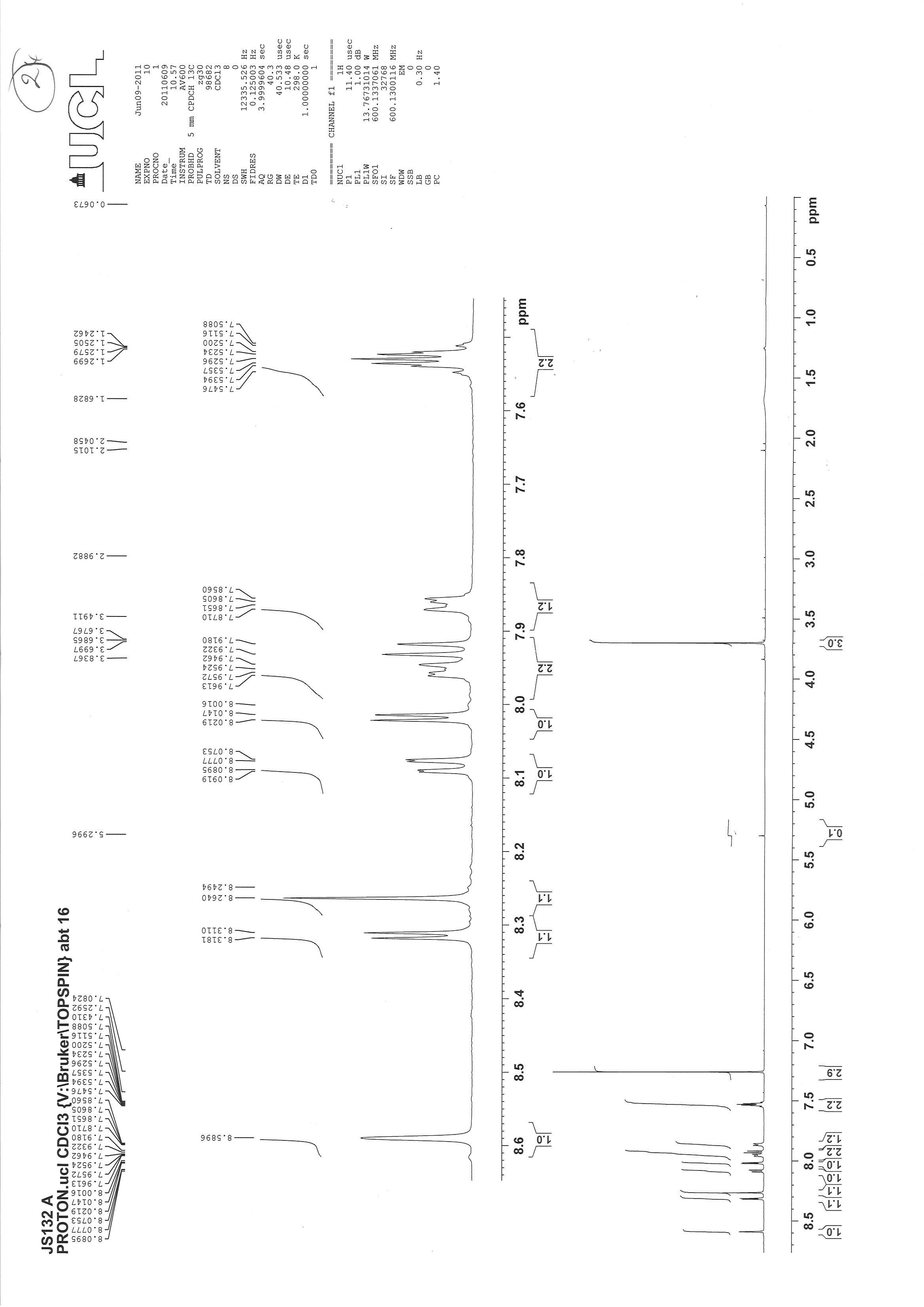
**

**8-(Methylsulfonyl)-2-(naphthalen-2-yl)imidazo[1,2-*a*]-pyrazine 24.**

**
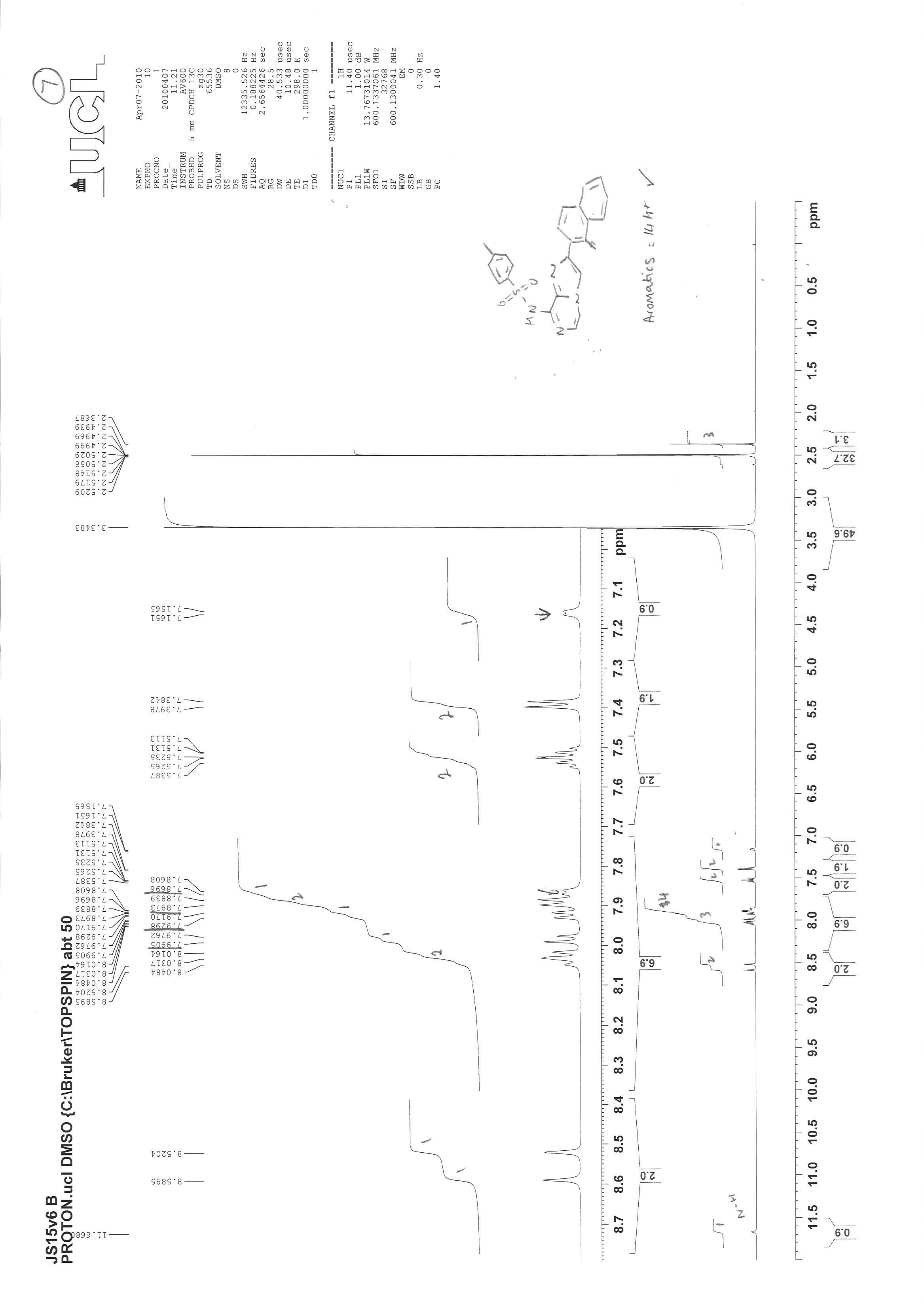
**

**4-Methyl-*N*-[2-(2-naphthyl)imidazo[1,2-*a*]pyrazine-8-yl]benzenesulfonamide 7**

**
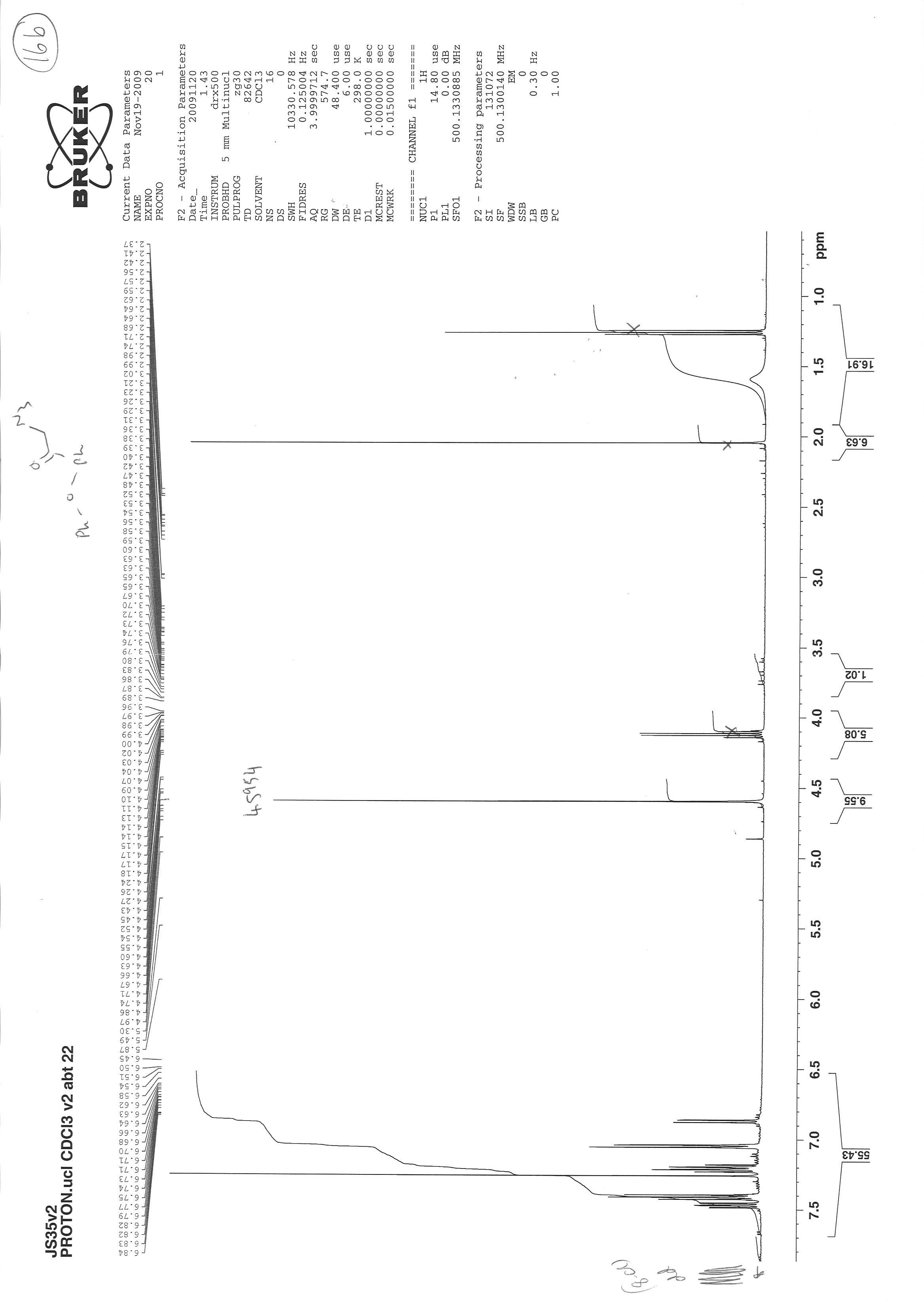
**

**2-Azido-1-(2-phenoxyphenyl)ethanone 16b**

**
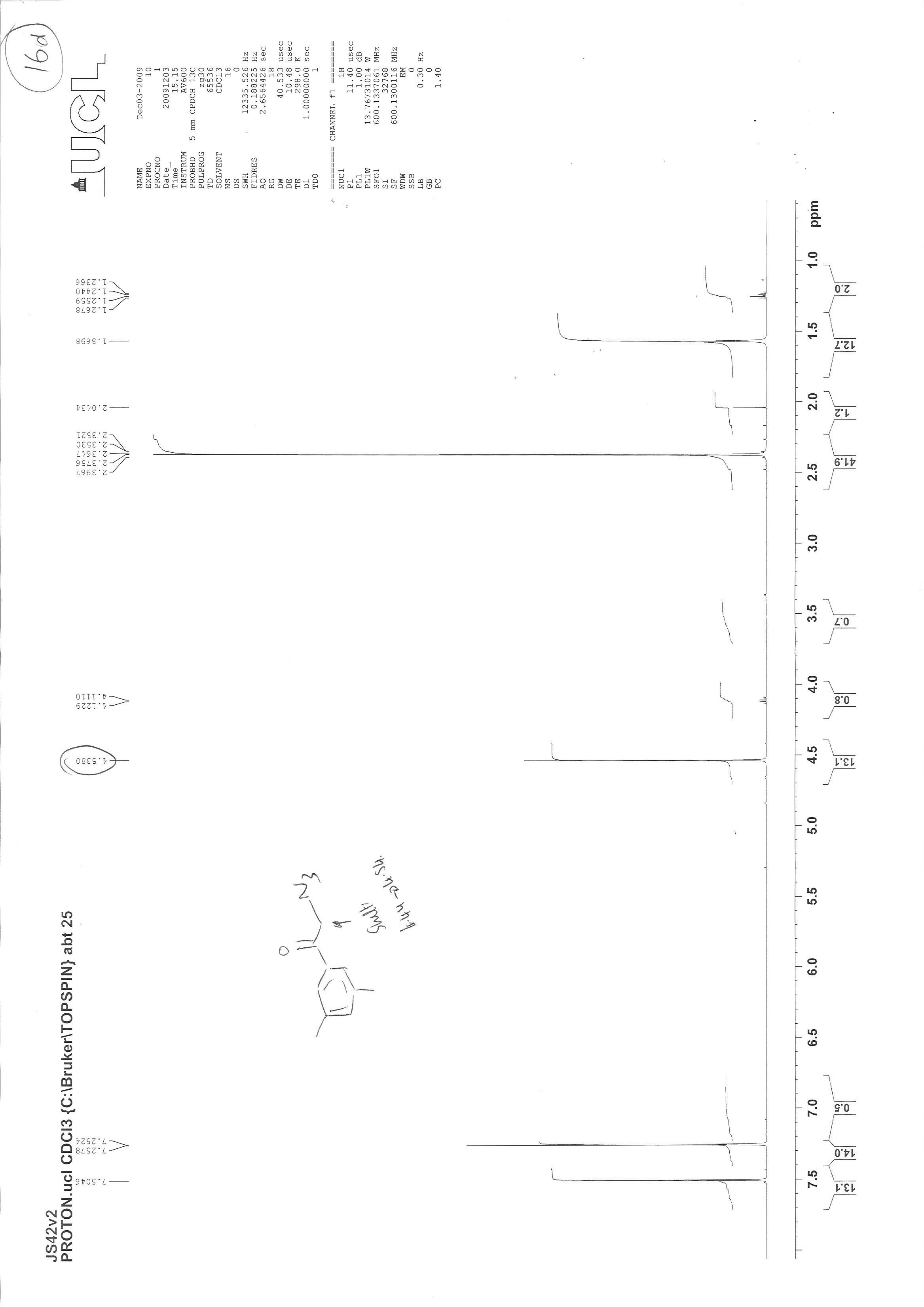
**

**2-Azido-1-(3,5-dimethylphenyl)ethanone 16d**

**
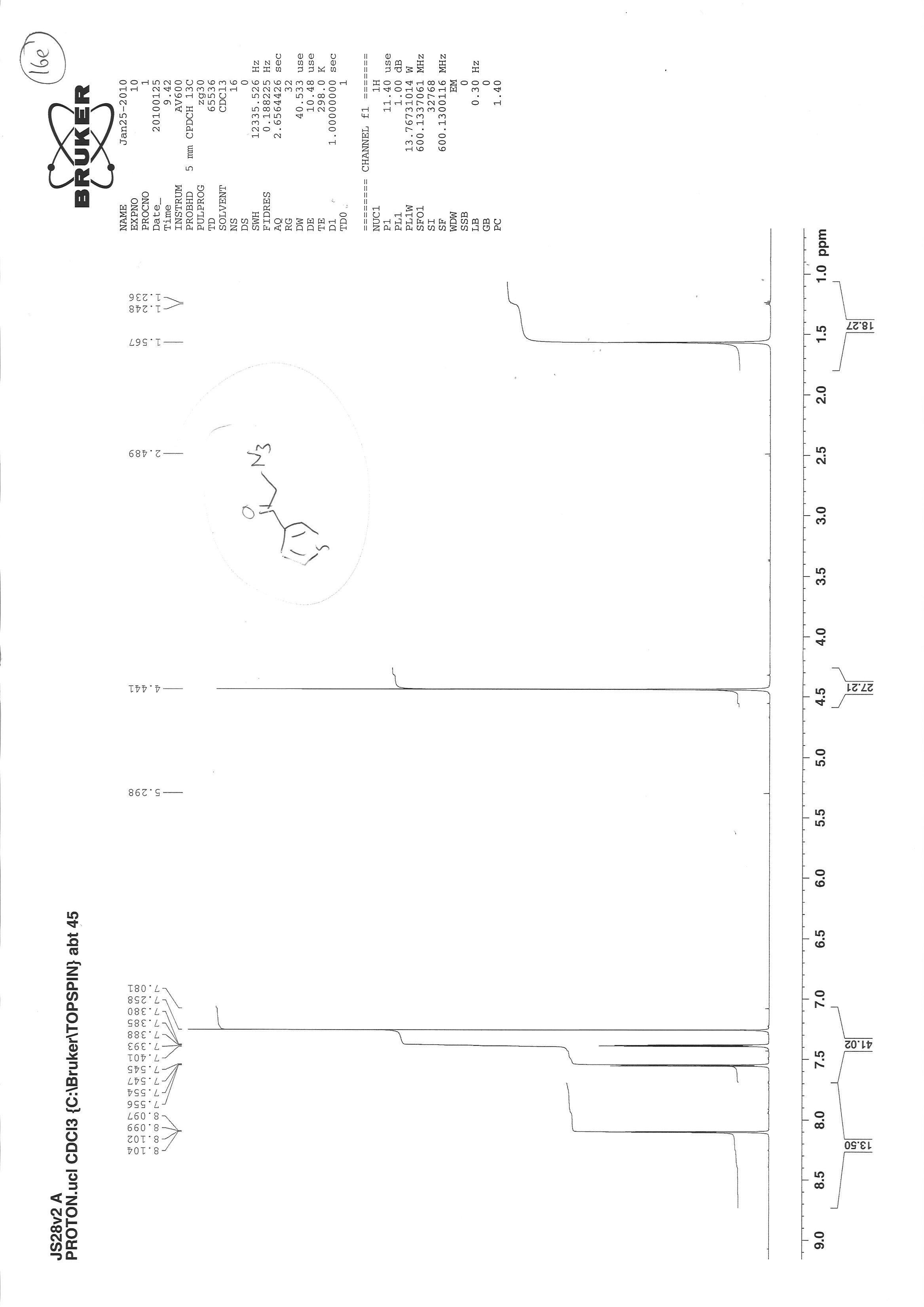
**

**2-Azido-1-(3-thienyl)ethanone 16e**

**
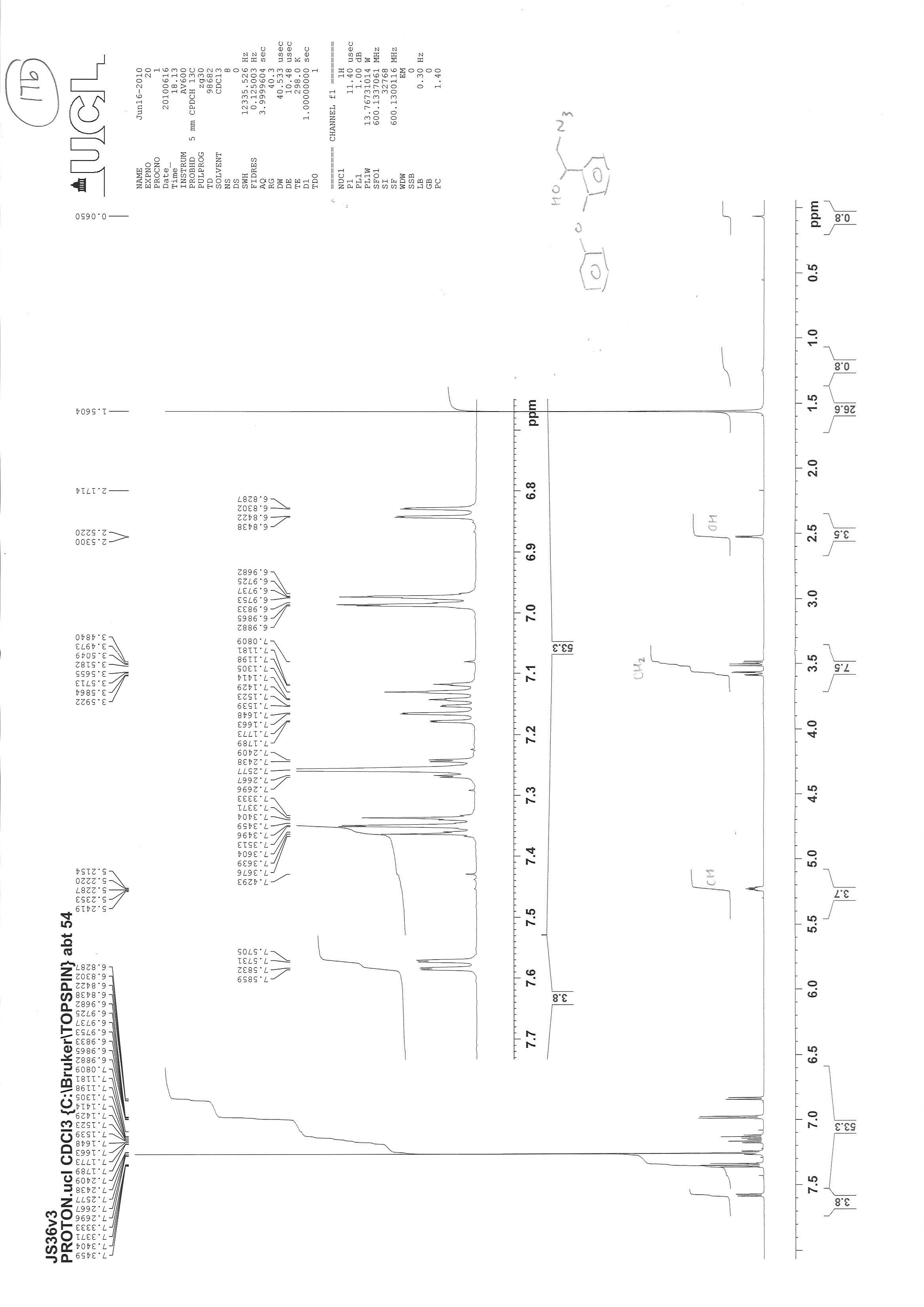
**

**2-Azido-1-(2-phenoxyphenyl)ethanol, 17b**

**
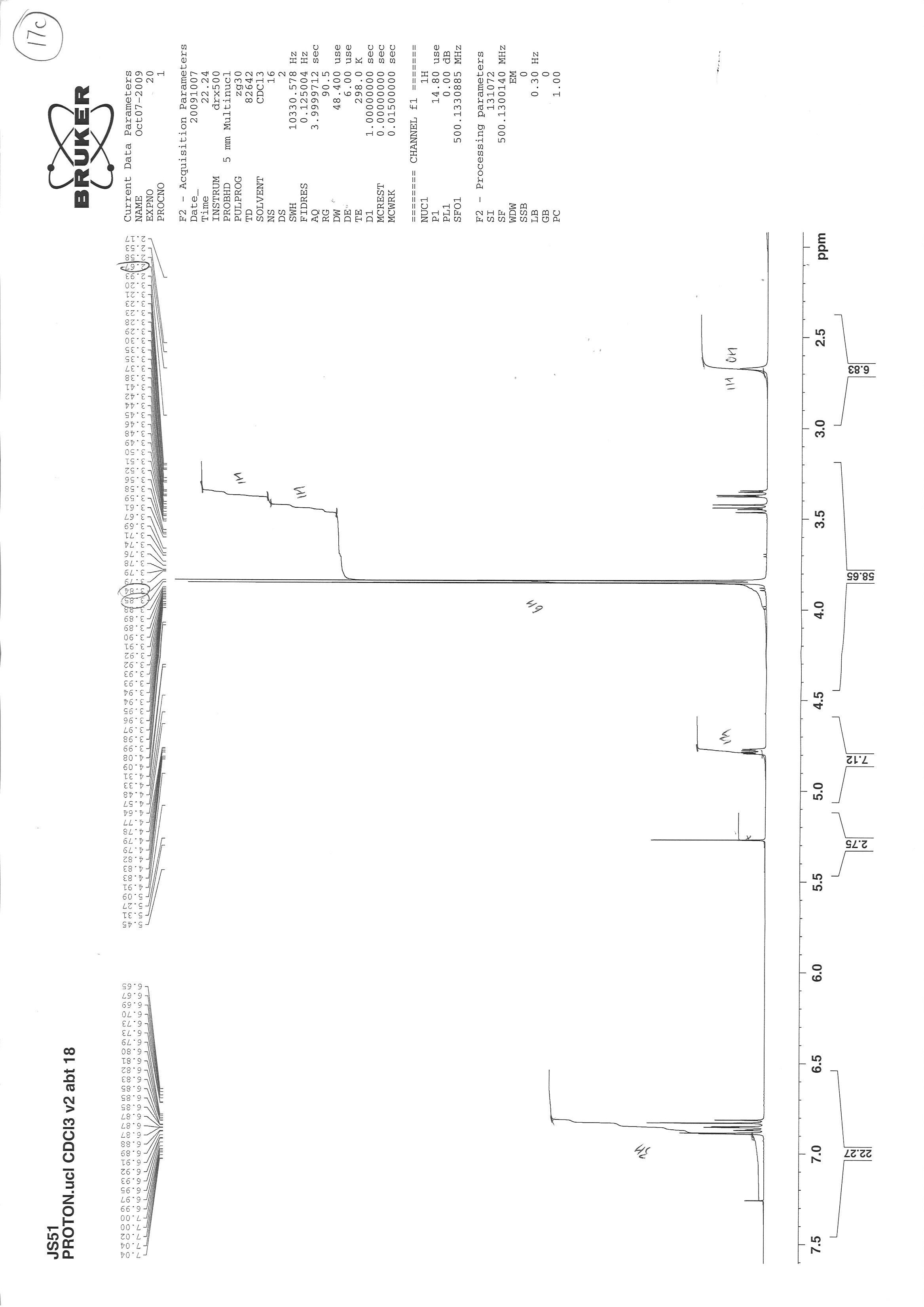
**

**2-Azido-1-(3,4-dimethoxyphenyl)ethanol, 17c**

**
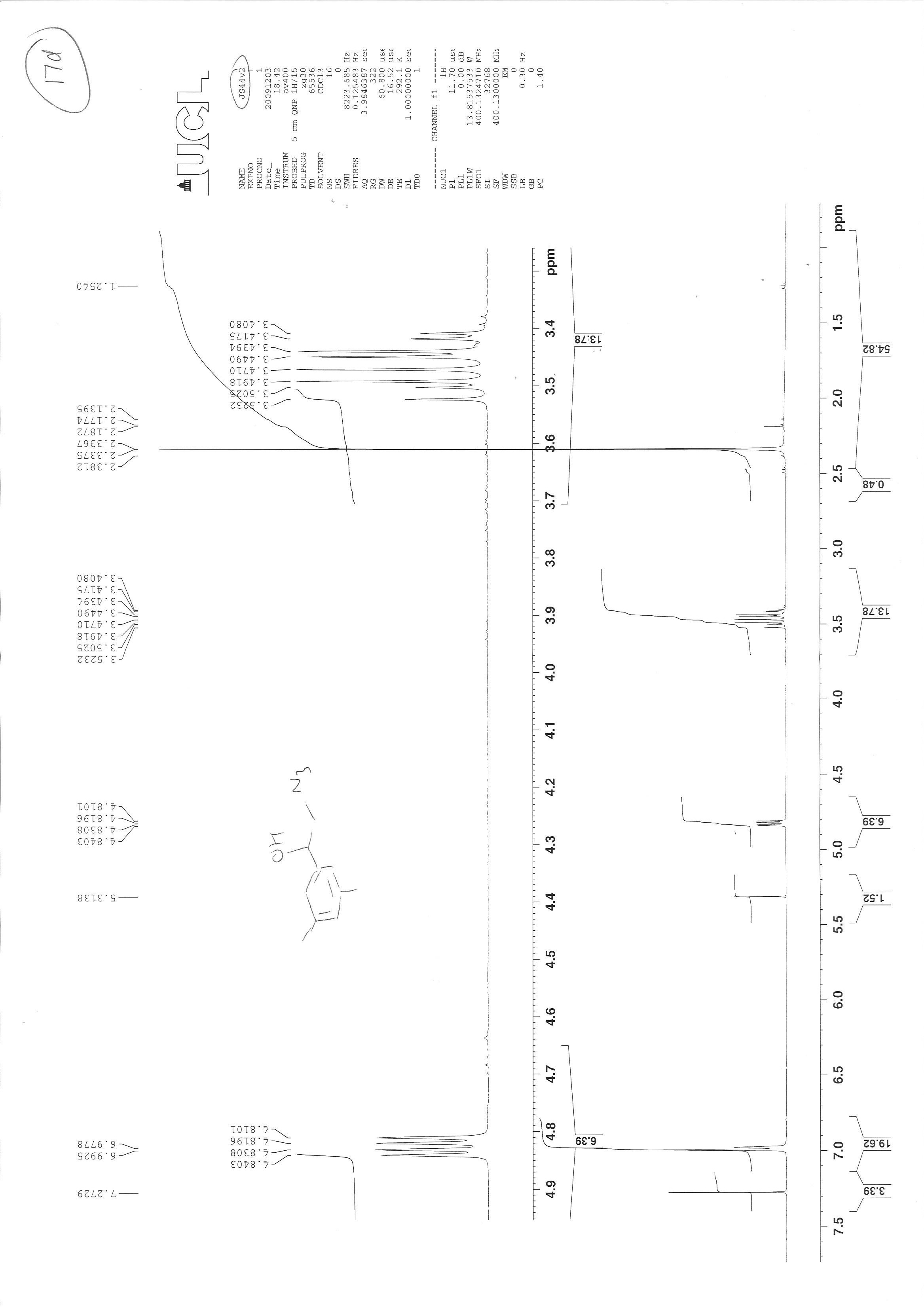
**

**2-Azido-1-(3,5-dimethylphenyl)ethanol, 17d**

**
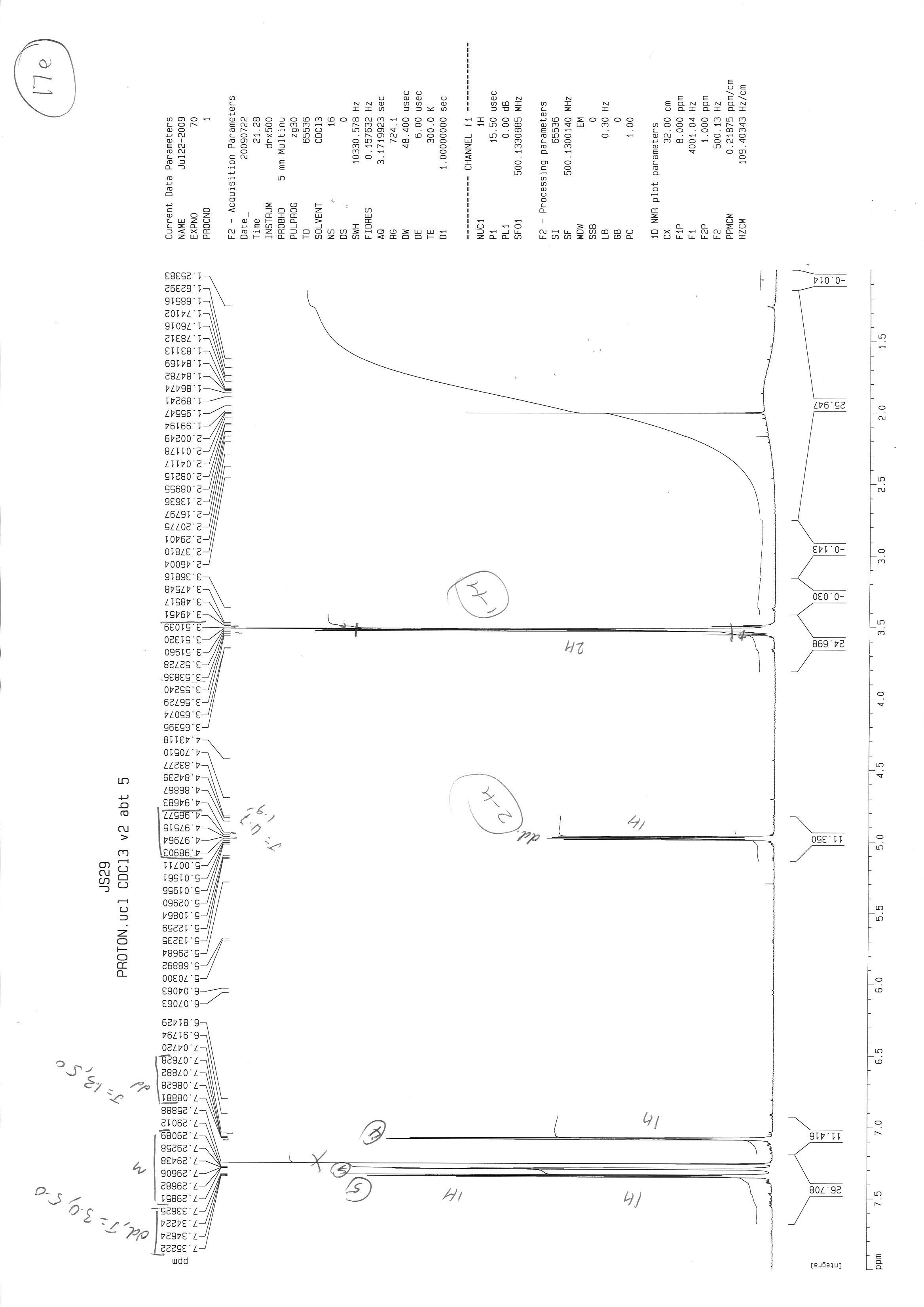
**

**2-Azido-1-(3-thienyl)ethanol, 17e**

**
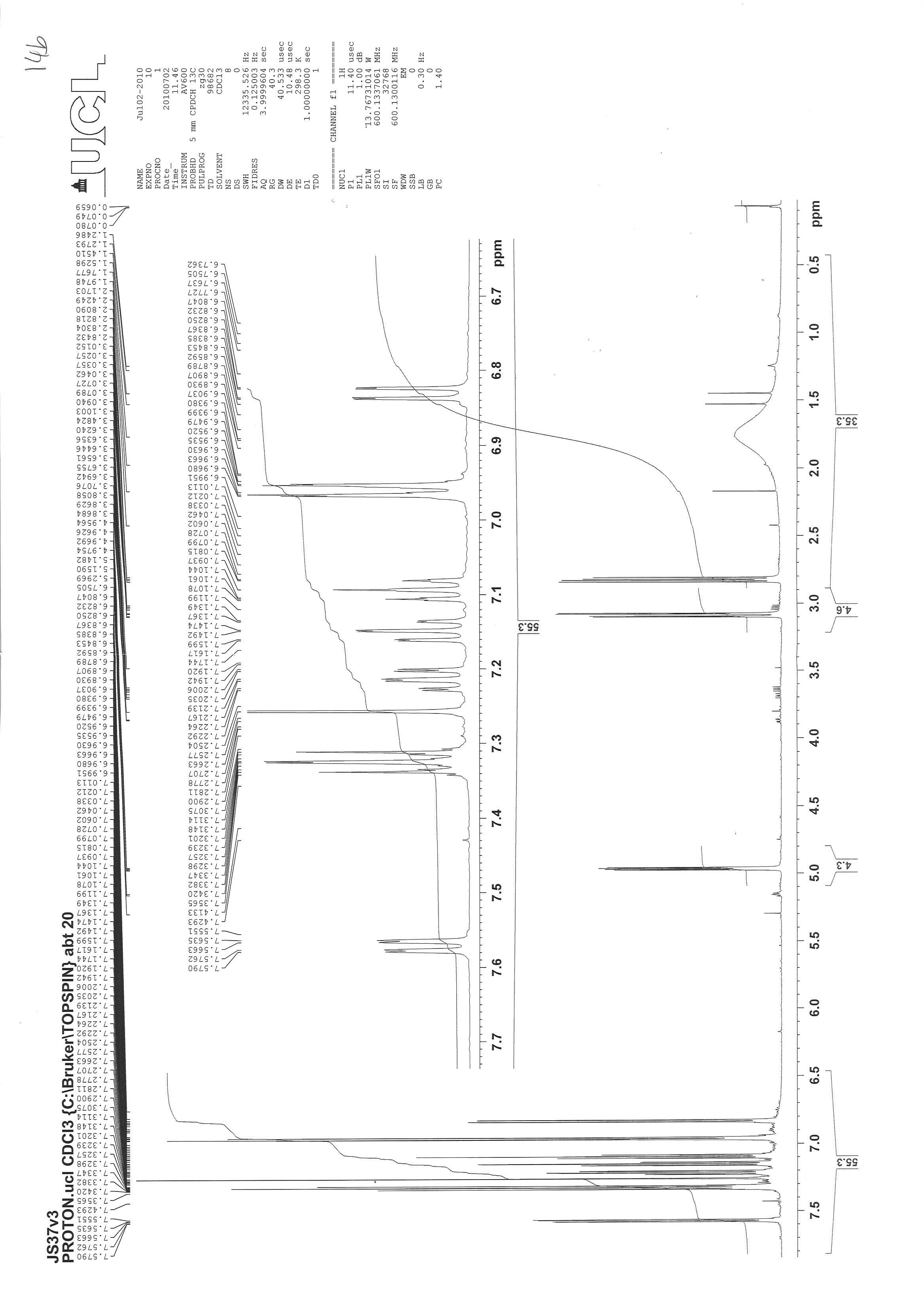
**

**2-Amino-1-(2-phenoxyphenyl)ethanol, 14b**

**
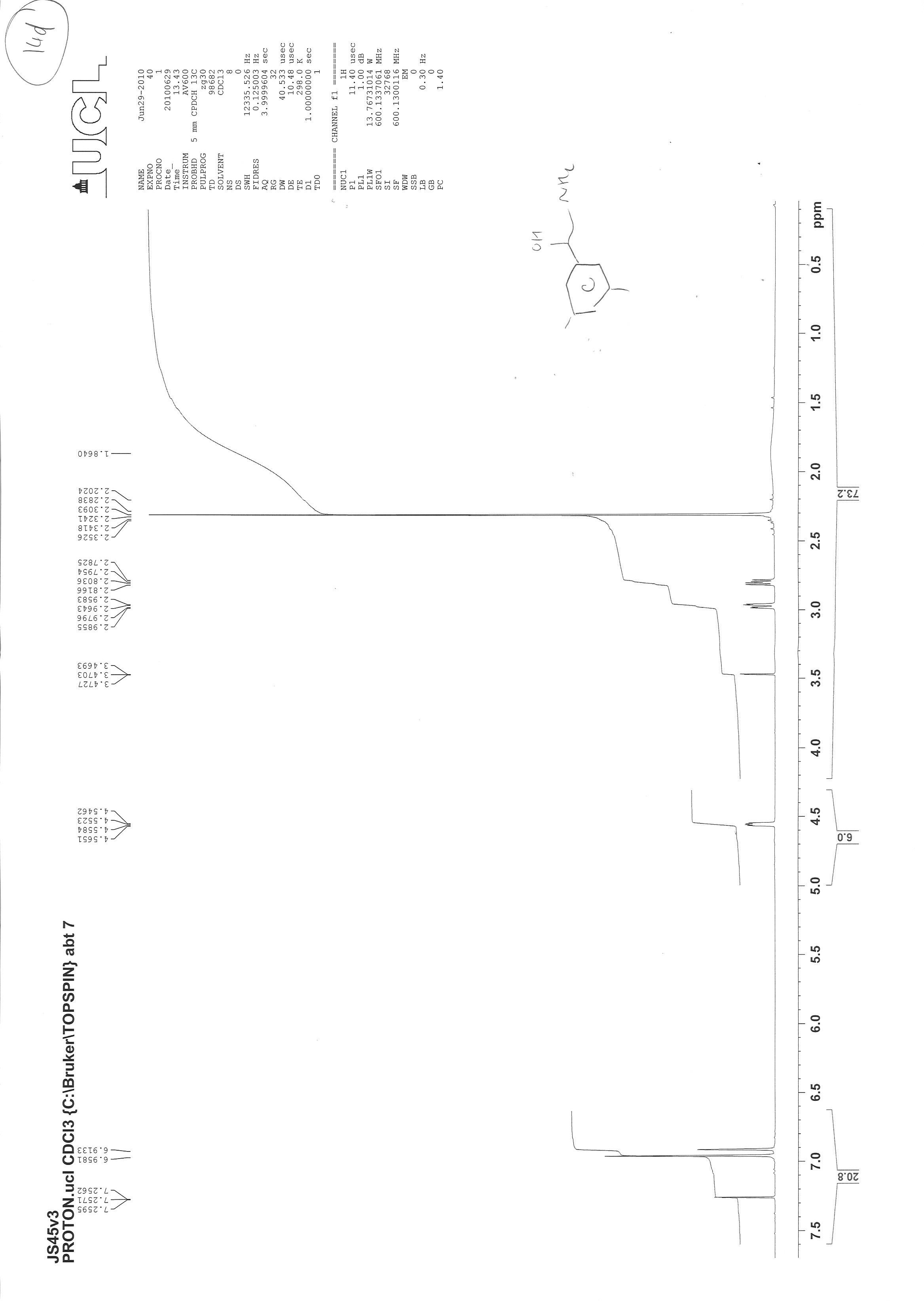
2-Amino-1-(3,5-dimethylphenyl)ethanol, 14d**

**
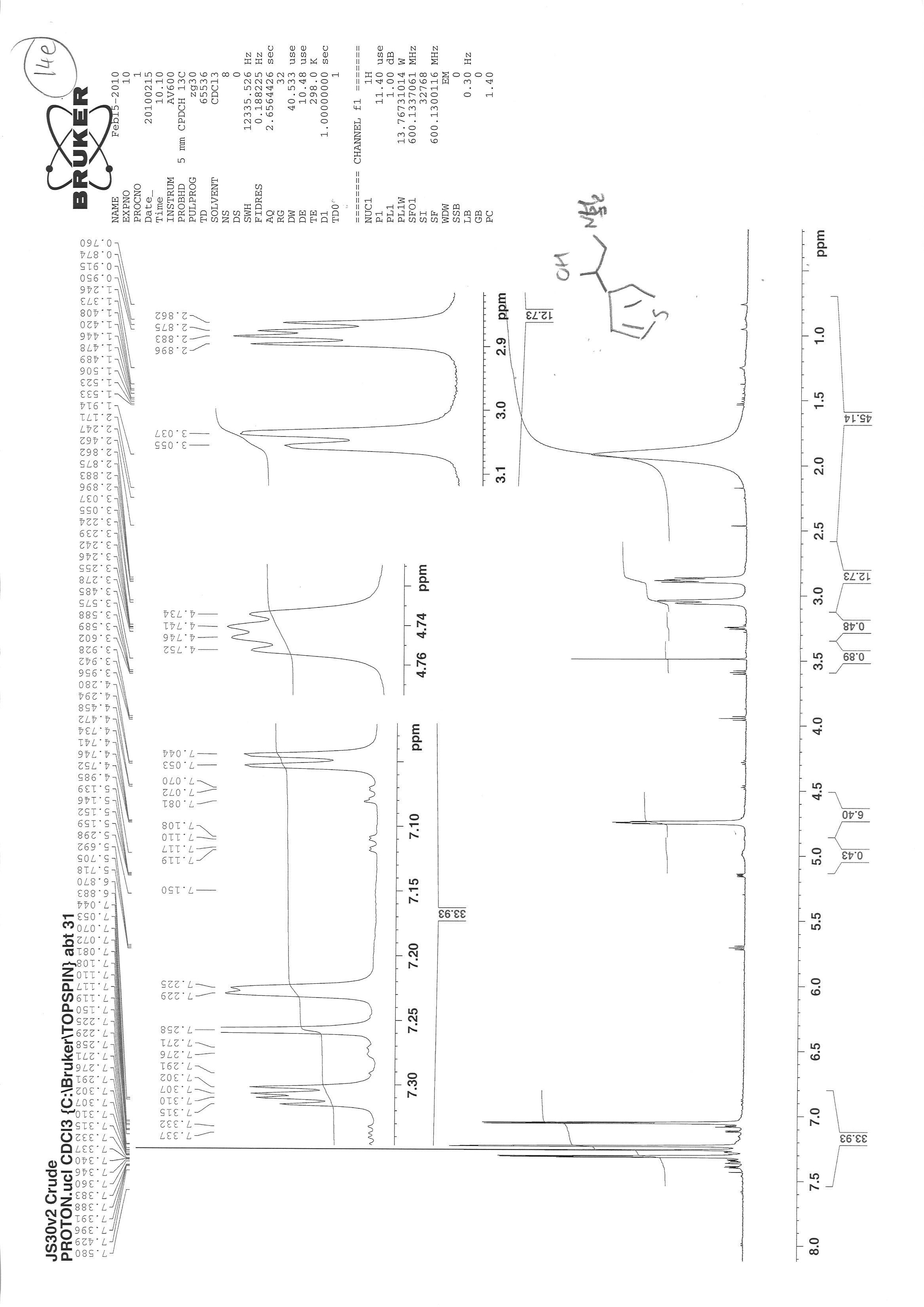
**

**2-Amino-1-(3-thienyl)ethanol, 14e**

**
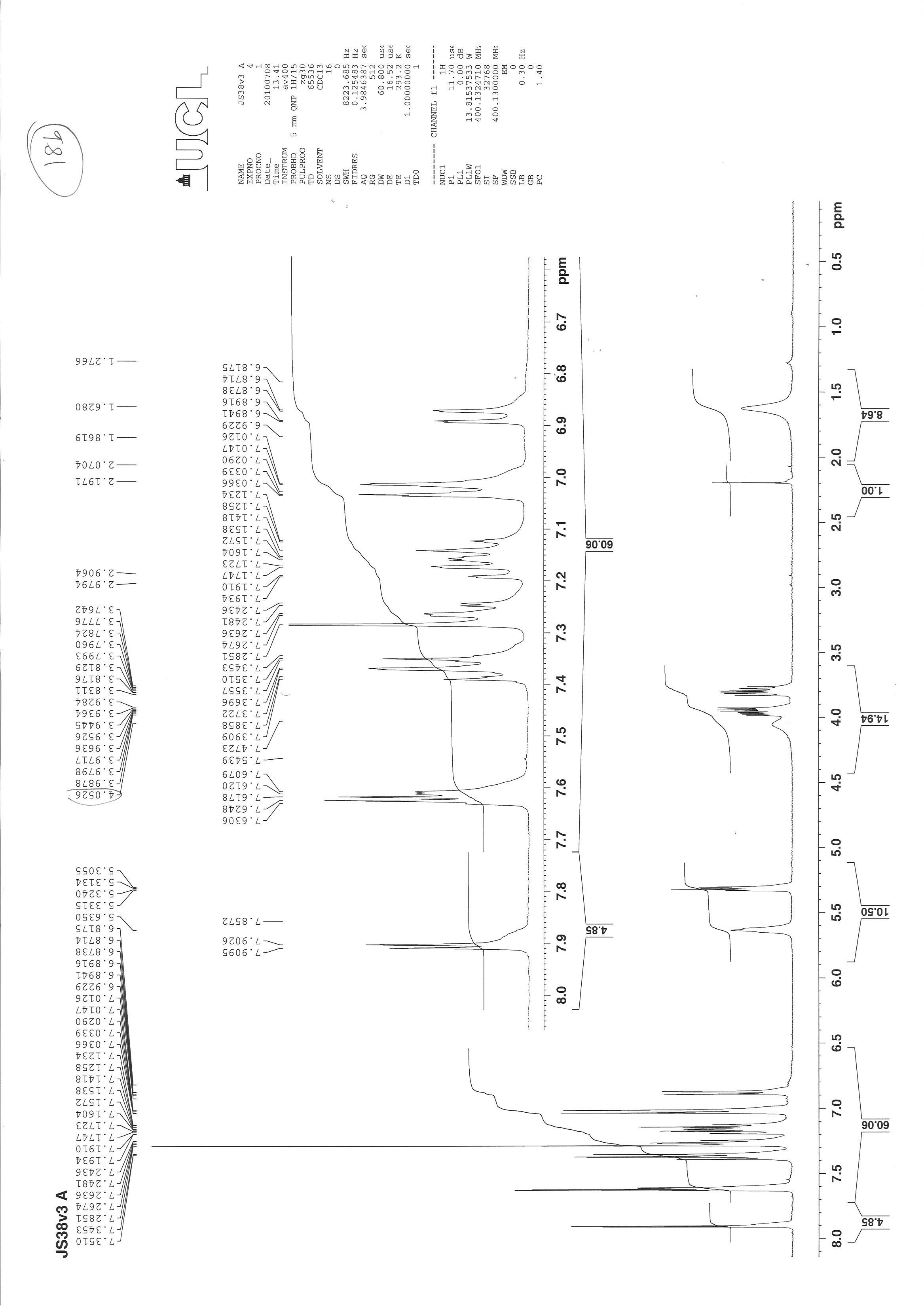
**

**2-[(3-Chloropyrazin-2-yl)amino]-1-(2-phenoxyphenyl)ethanol, 18b**

**
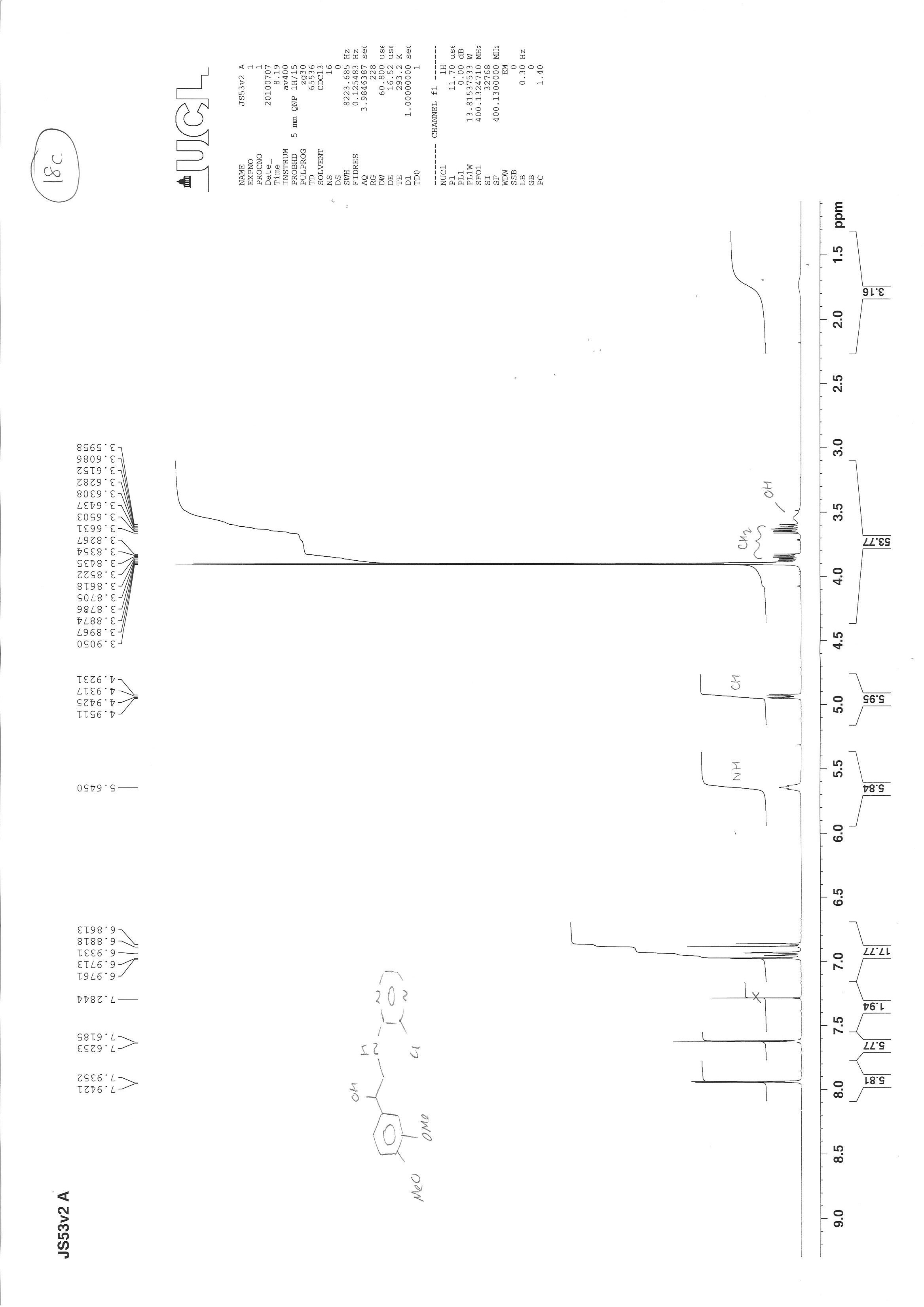
**

**2-[(3-Chloropyrazin-2-yl)amino]-1-(3,4-dimethoxyphenyl)ethanol, 18c**

**
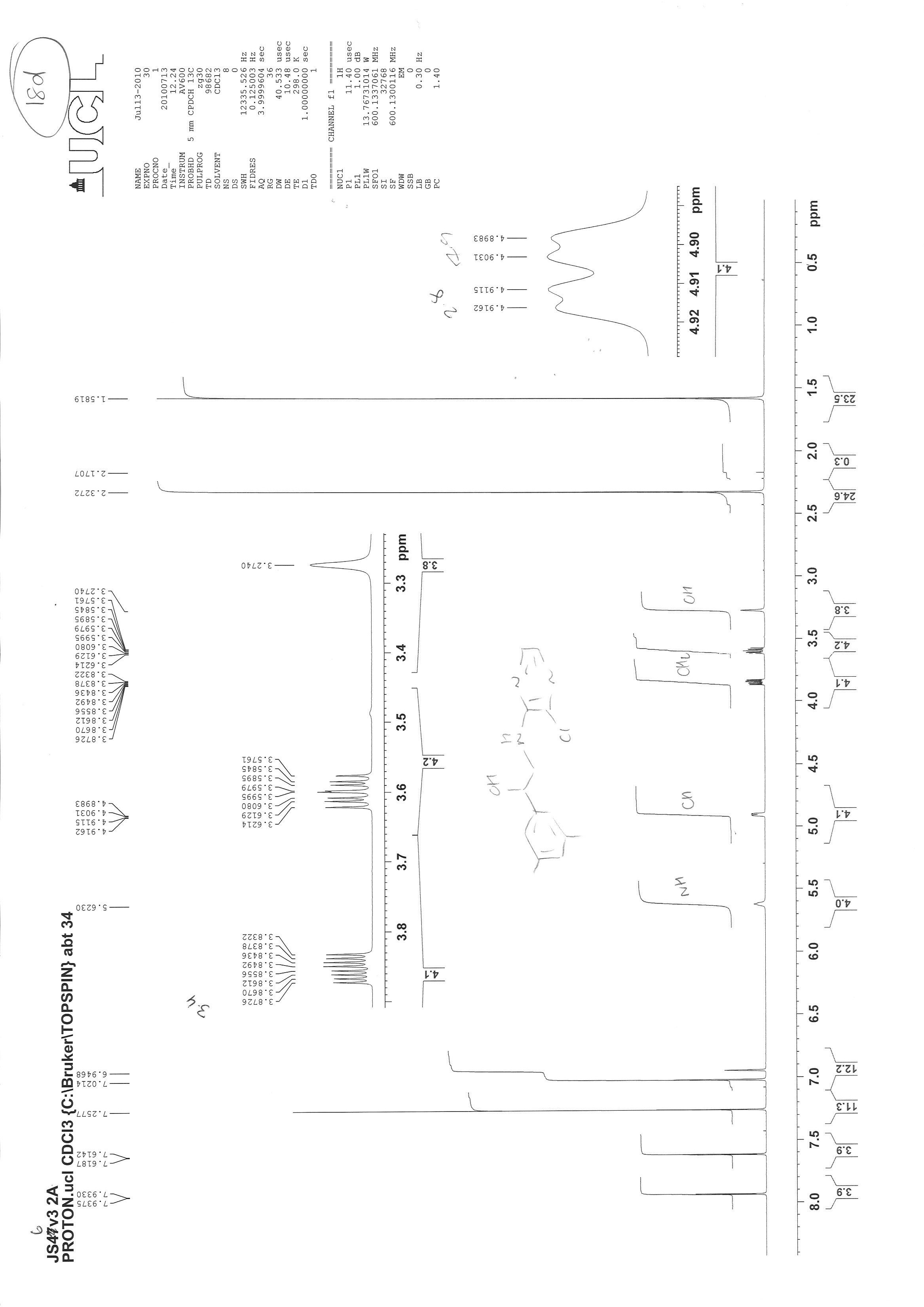
**

**2-[(3-Chloropyrazin-2-yl)amino]-1-(3,5-dimethylphenyl)ethanol, 18d**

**
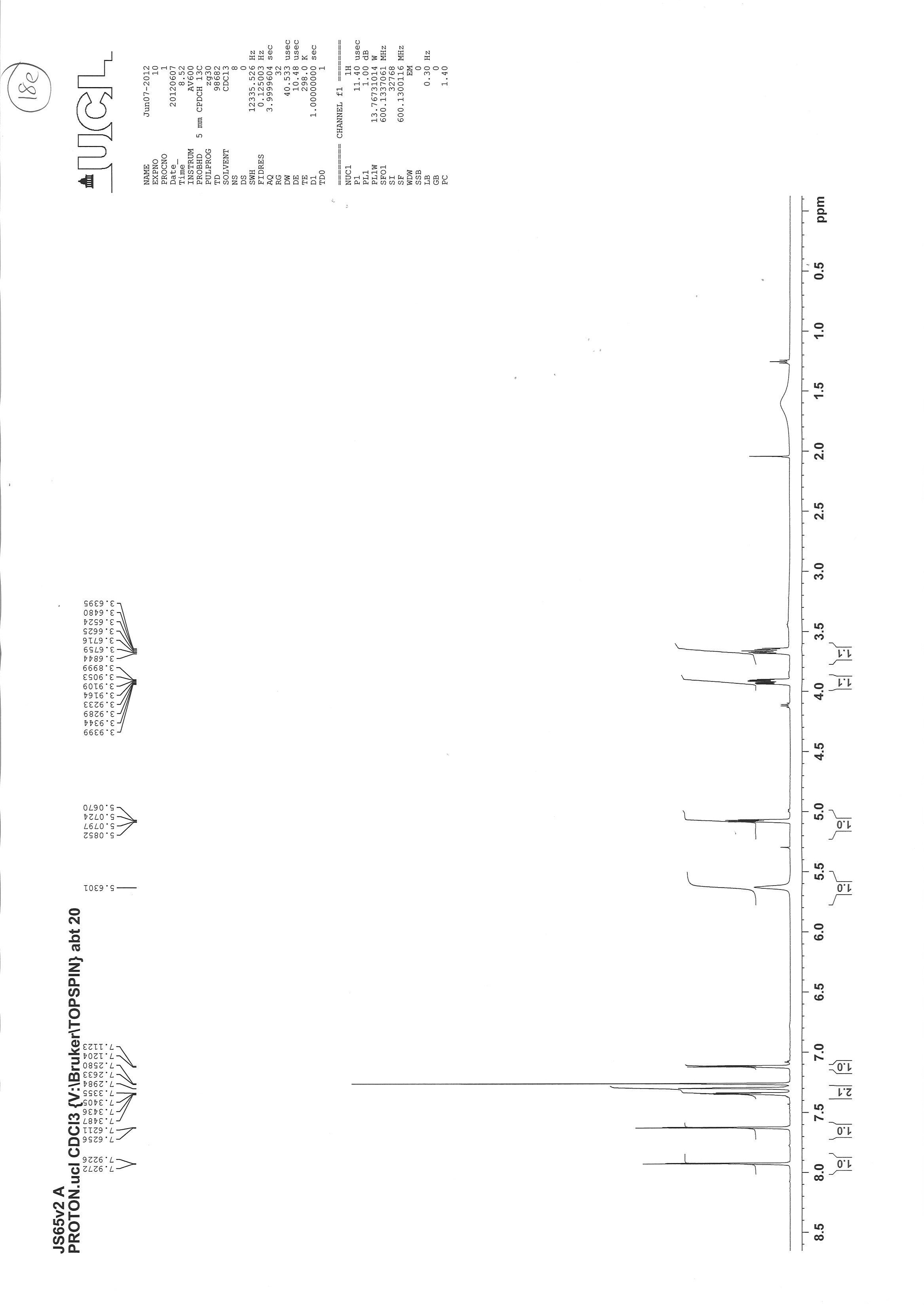
**

**2-[(3-Chloropyrazin-2-yl)amino]-1-(3-thienyl)ethanol, 18e**

**
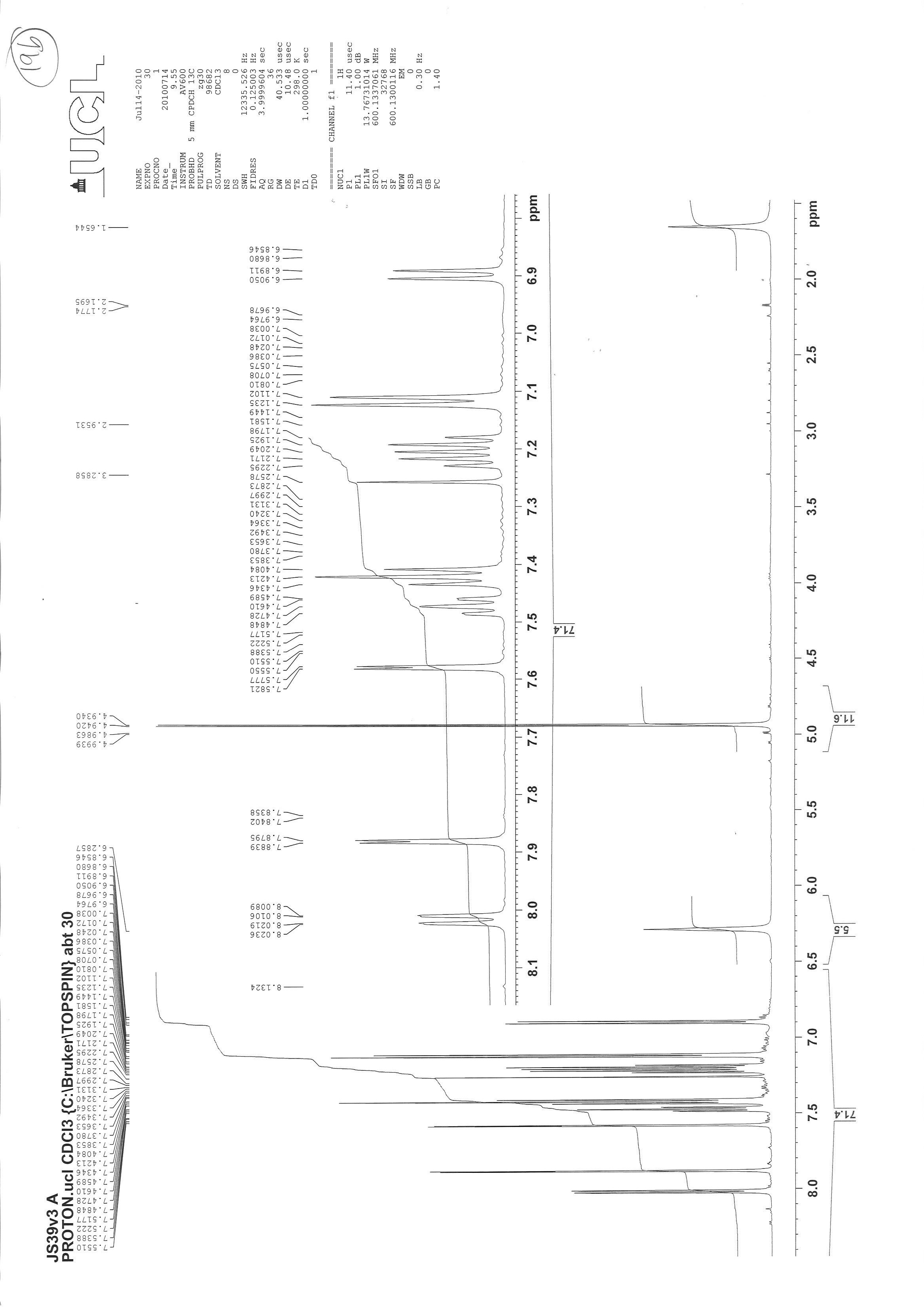
**

**2-[(3-Chloropyrazin-2-yl)amino]-1-(2-phenoxyphenyl)ethanone, 19b**

**
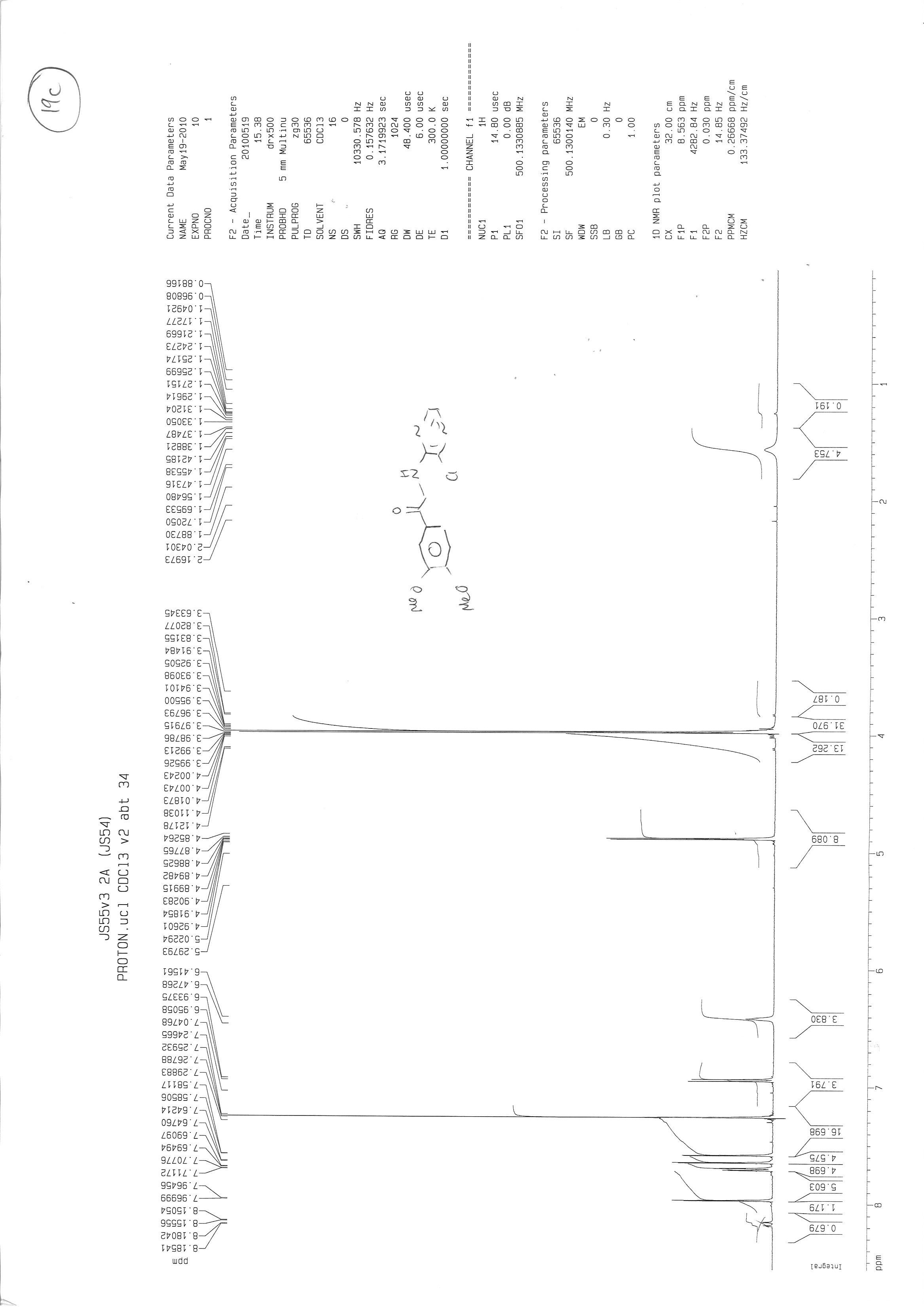
**

**2-[(3-Chloropyrazin-2-yl)amino]-1-(3,4-dimethoxyphenyl)ethanone, 19c**

**
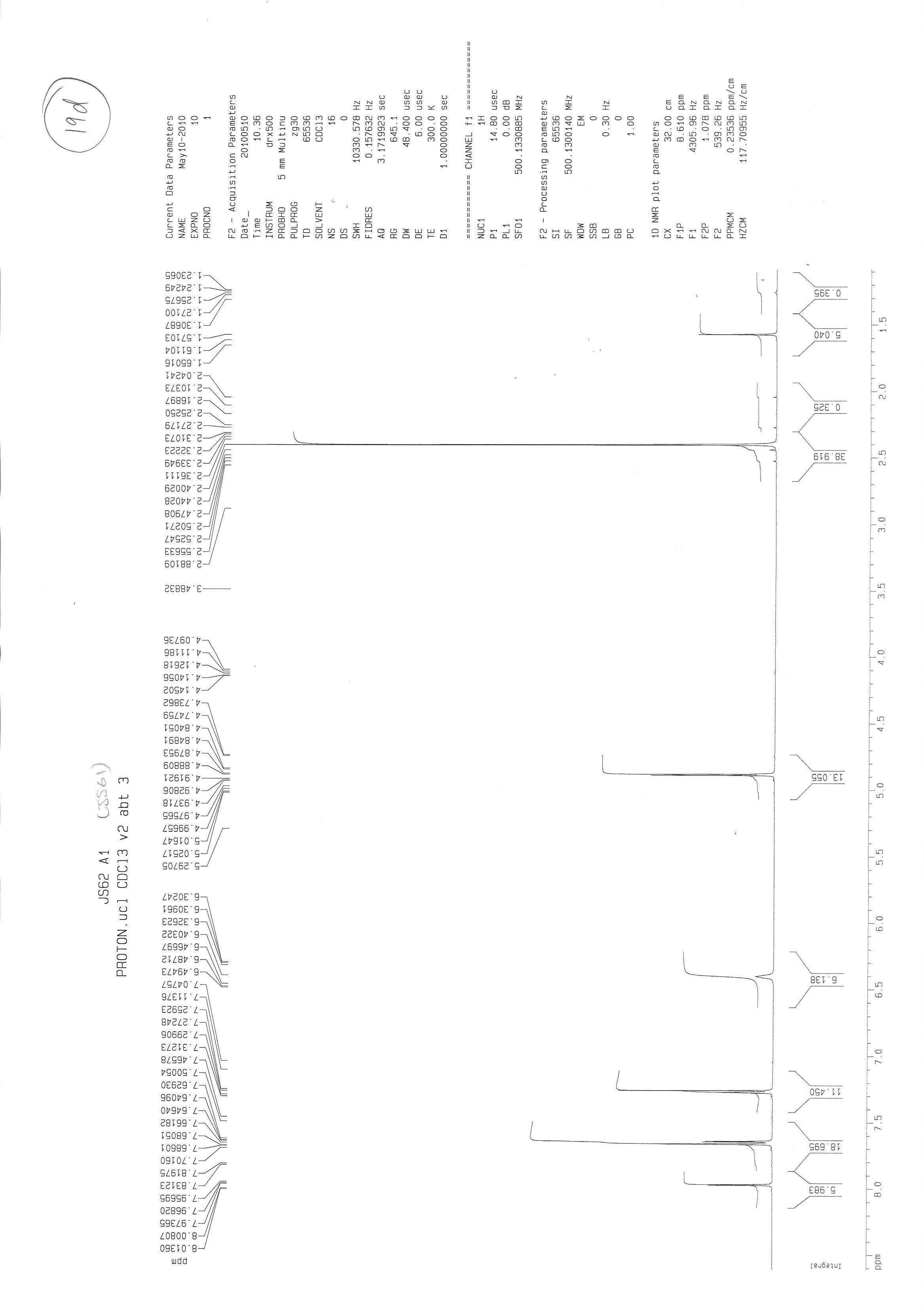
**

**2-[(3-Chloropyrazin-2-yl)amino]-1-(3,5-dimethylphenyl)ethanone, 19d**

**
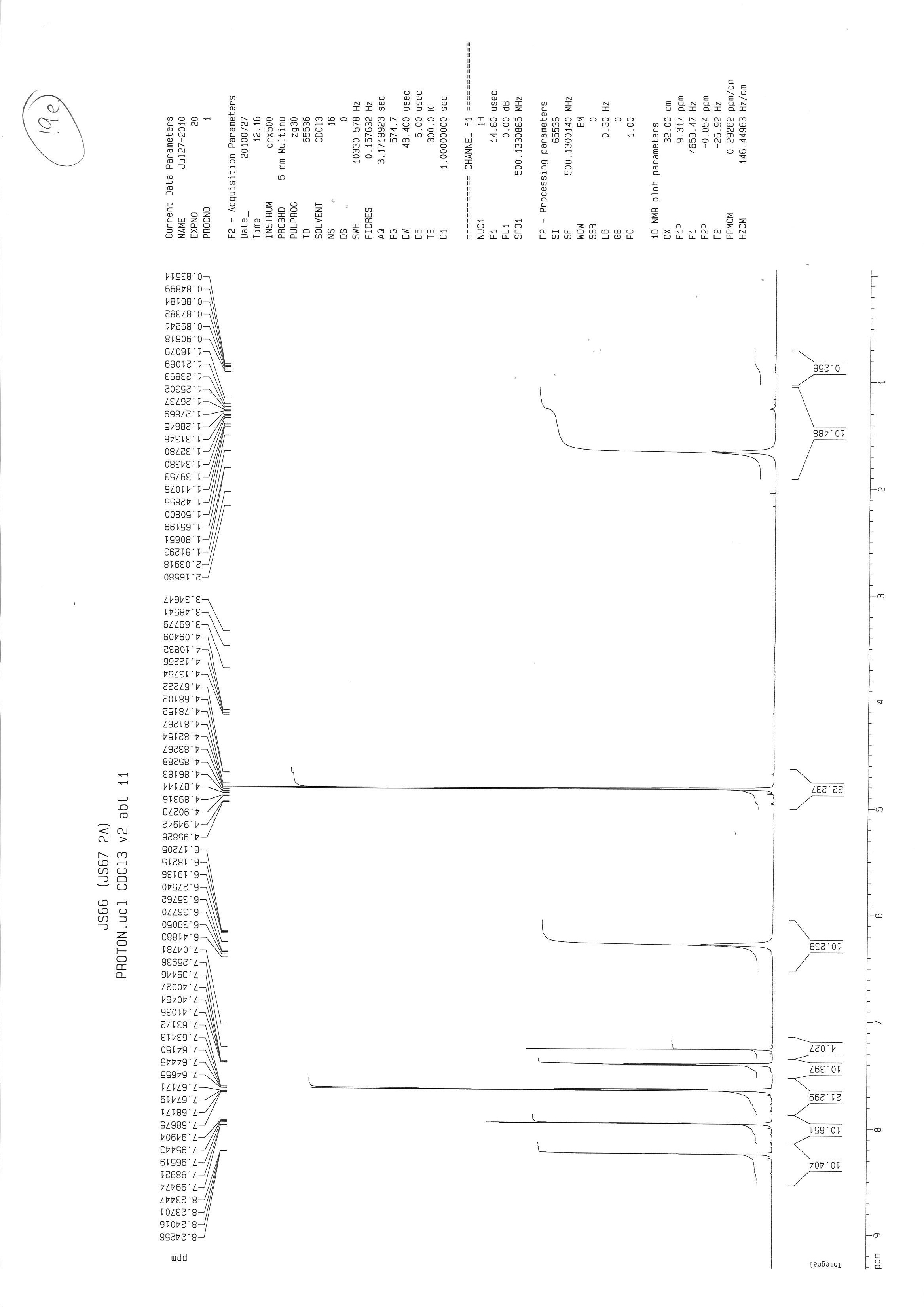
**

**2-[(3-Chloropyrazin-2-yl)amino]-1-(3-thienyl)ethanone, 19e**

**
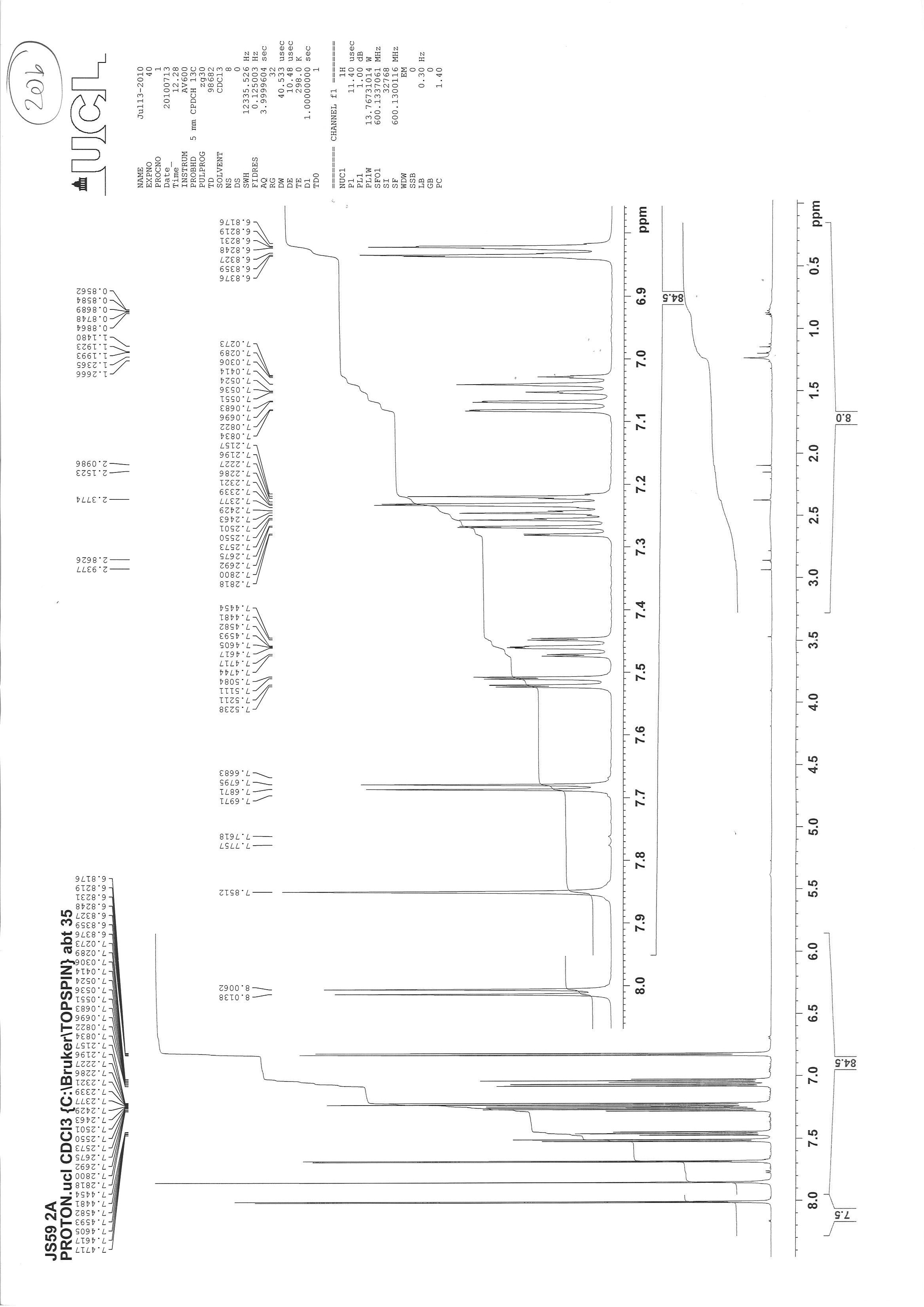
**

**8-Chloro-3-(2-phenoxyphenyl)imidazo[1,2-*a*]pyrazine, 20b**

**
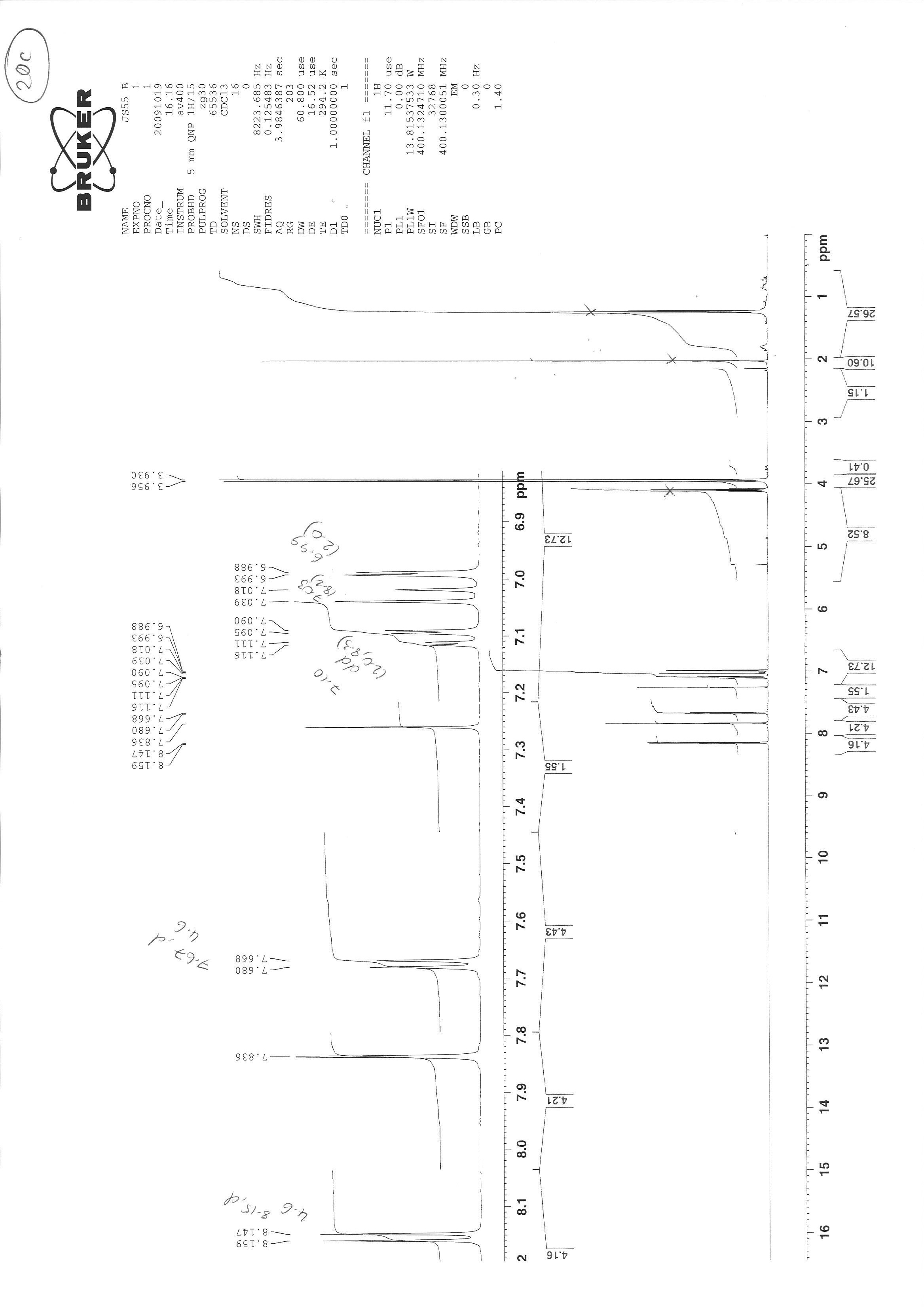
**

**8-Chloro-3-(3,4-dimethoxyphenyl)imidazo[1,2-*a*]pyrazine, 20c**

**
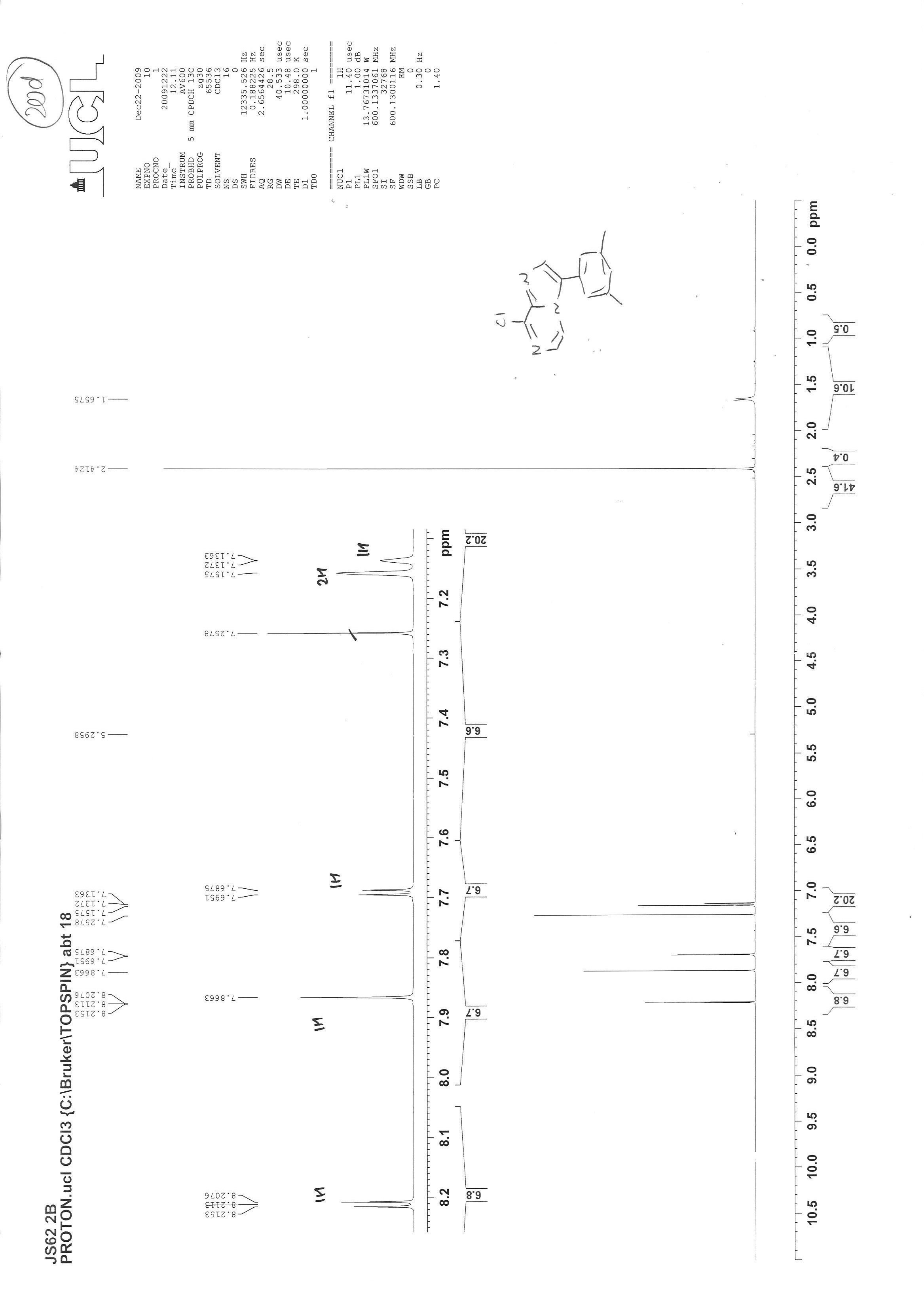
**

**8-Chloro-3-(3,5-dimethylphenyl)imidazo[1,2-*a*]pyrazine, 20d**


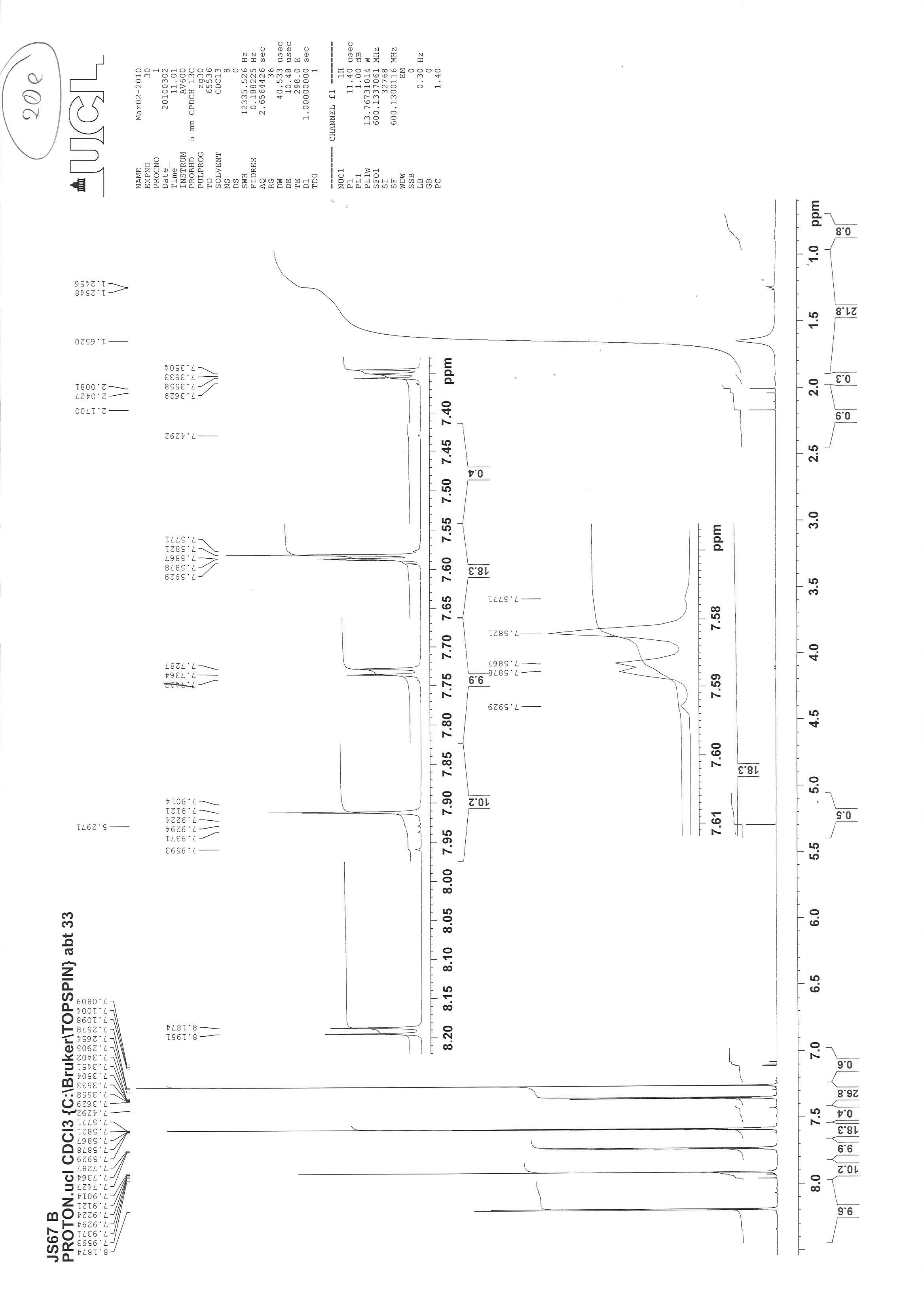


**8-Chloro-3-(3-thienyl)imidazo[1,2-*a*]pyrazine, 20e**

**
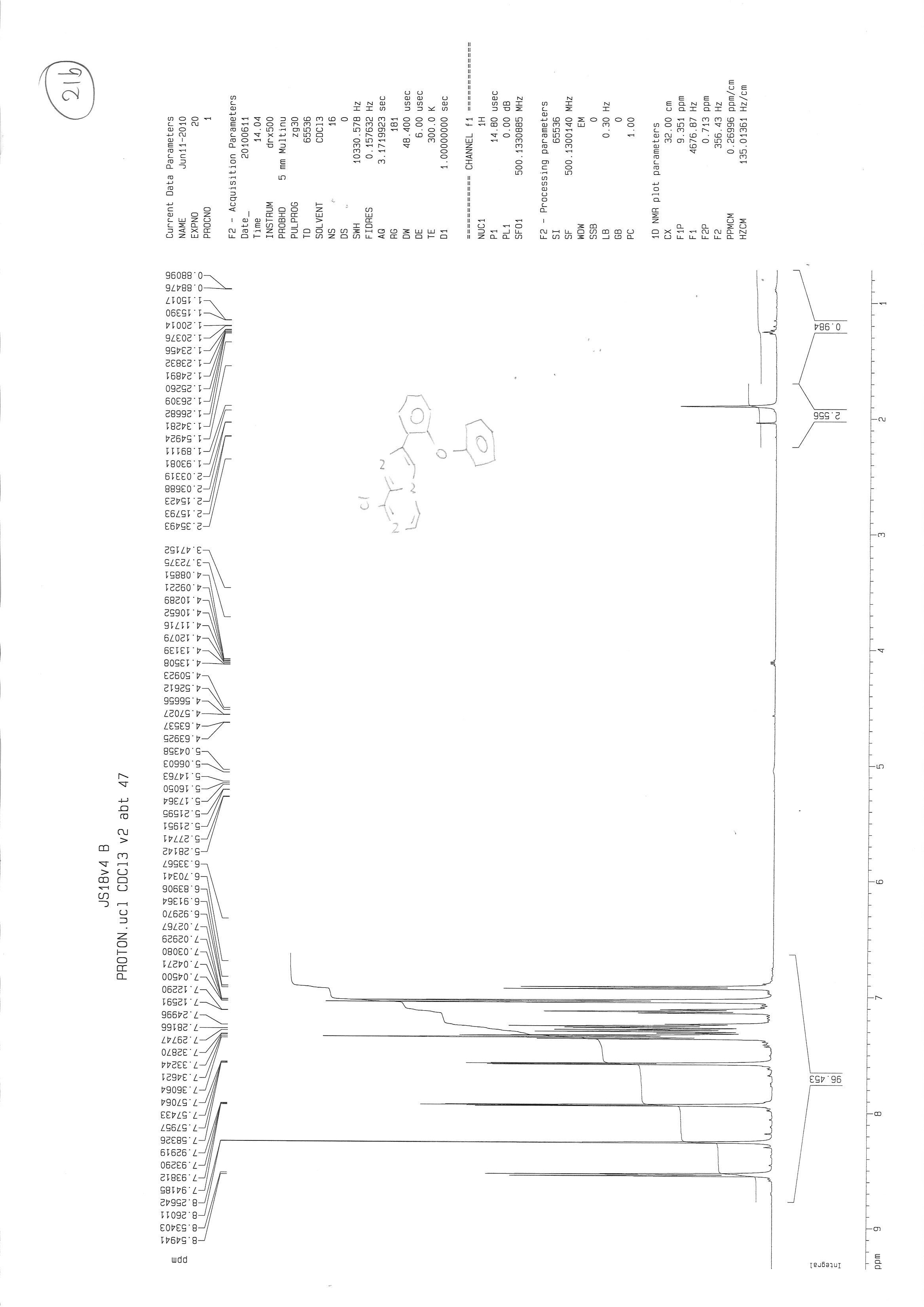
**

**8-Chloro-2-(2-phenoxyphenyl)imidazo[1,2-*a*]pyrazine*,* 21b**

**
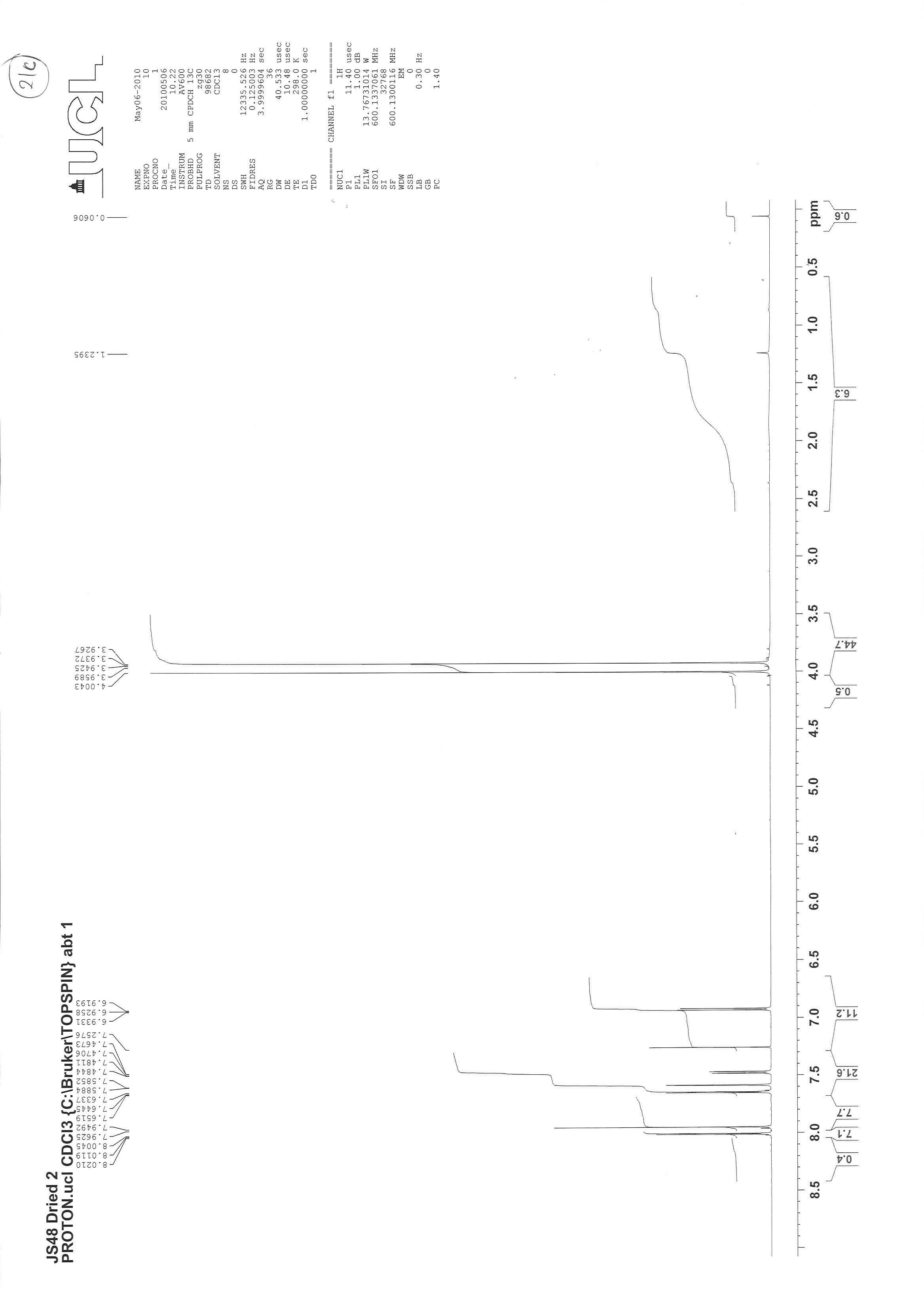
**

**8-Chloro-2-(3,4-dimethoxylphenyl)imidazo[1,2-*a*]pyrazine, 21c**

**
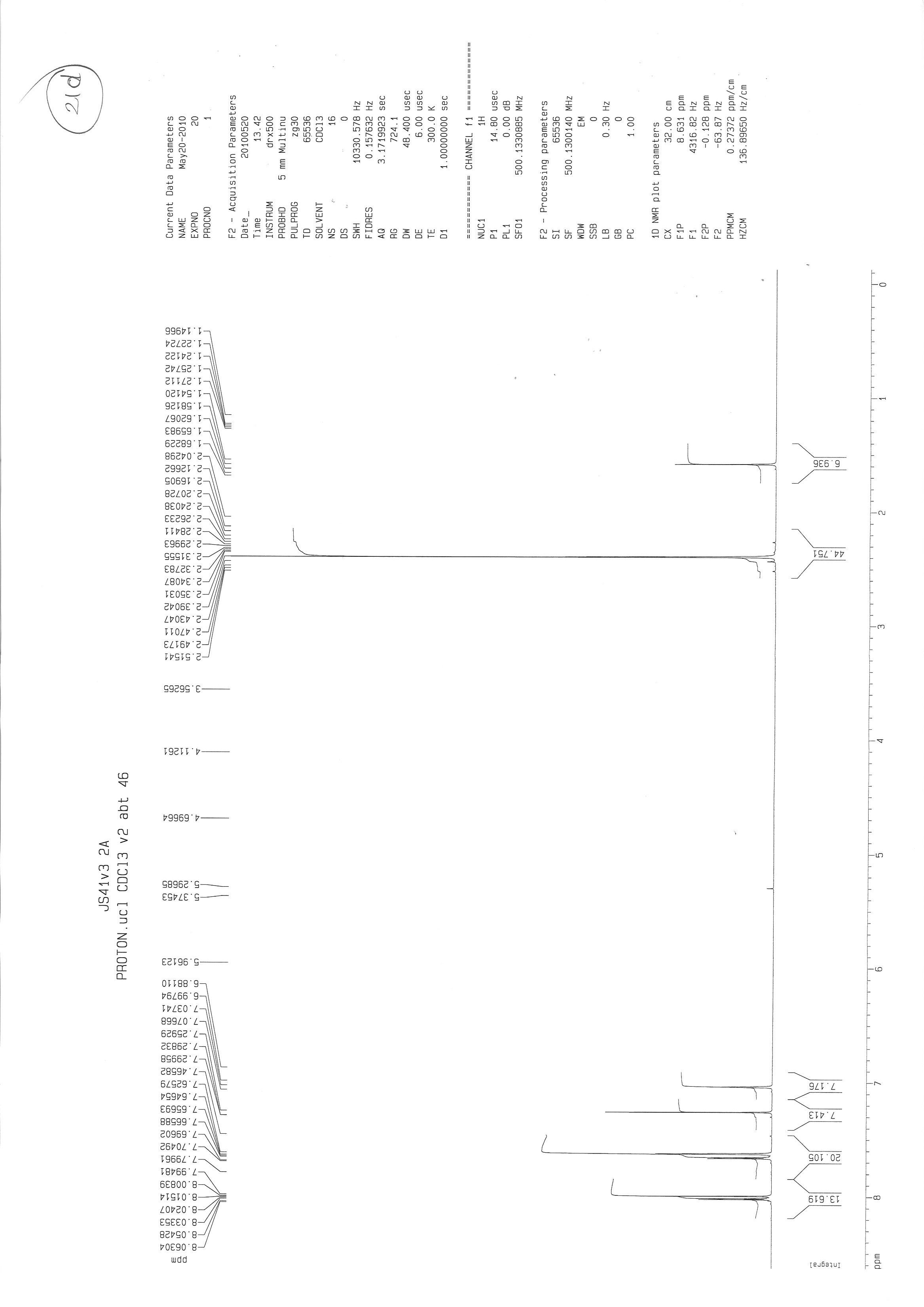
**

**8-Chloro-2-(3,5-dimethylphenyl)imidazo[1,2-*a*]pyrazine, 21d**

**
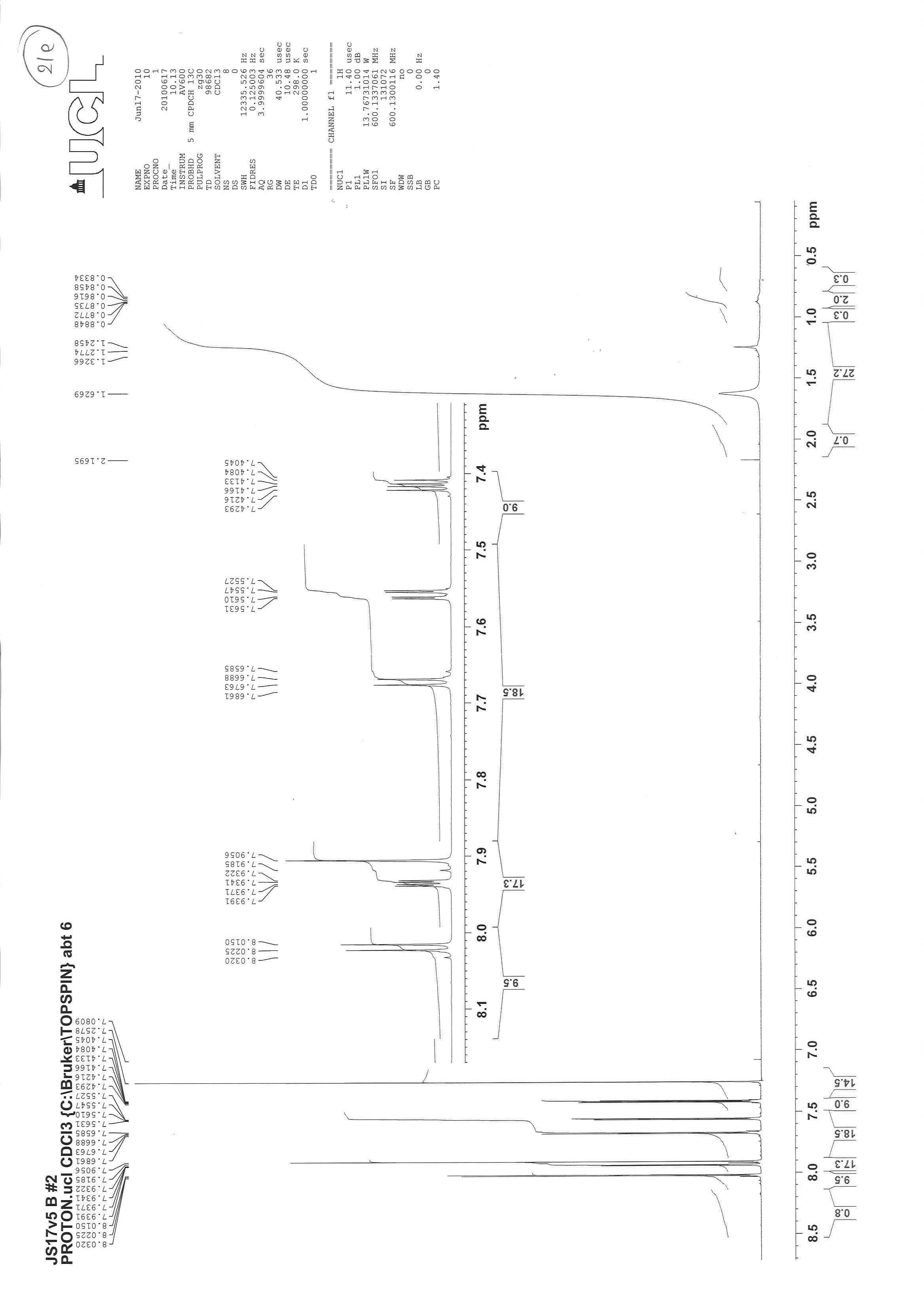
**

**8-Chloro-2-(3-thienyl)imidazo[1,2-*a*]pyrazine, 21e**

**
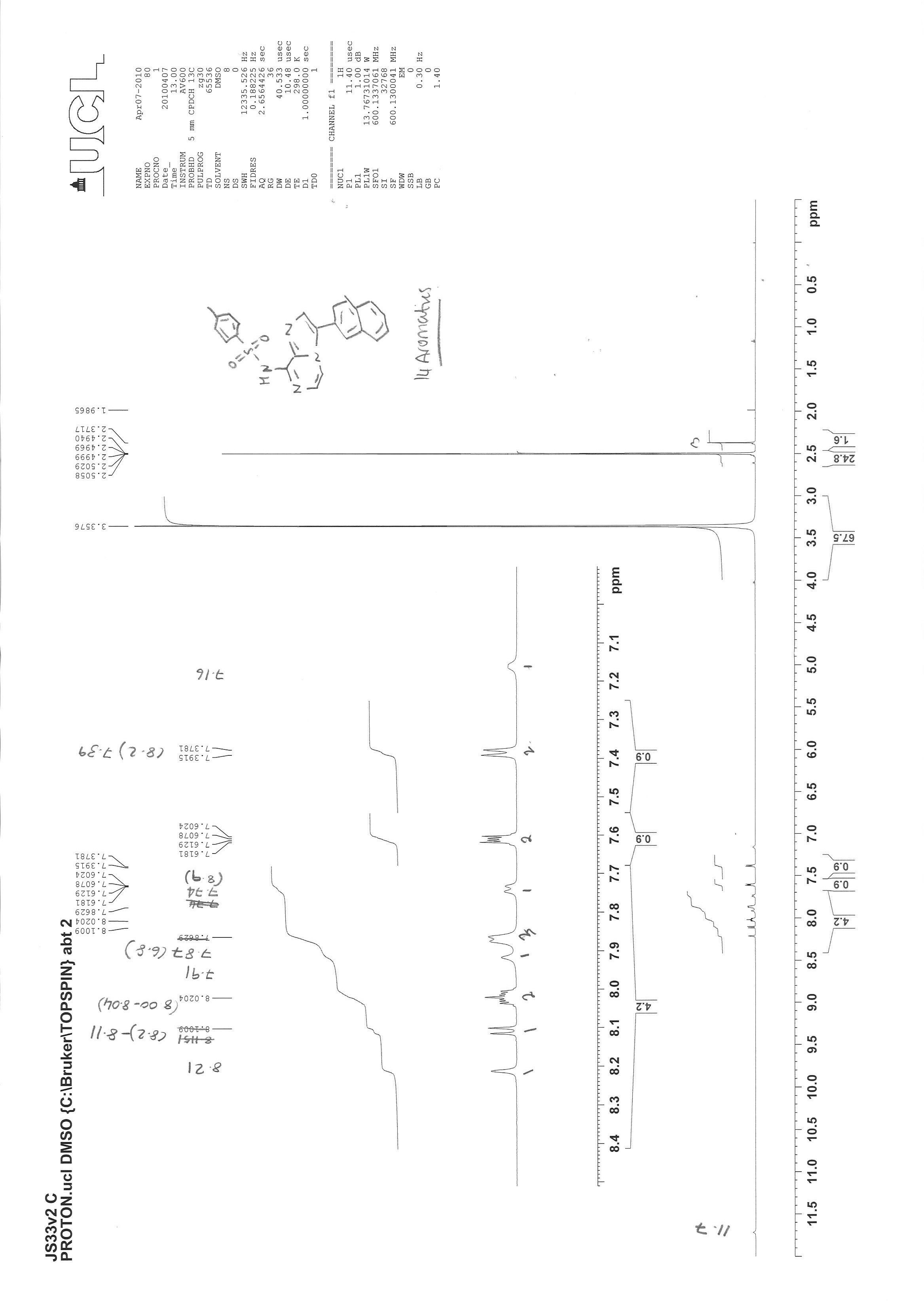
**

**4-Methyl-*N*-[2-(2-naphthyl)imidazo[1,2-*a*]pyrazine-8-yl]benzenesulfonamide, 1**

**
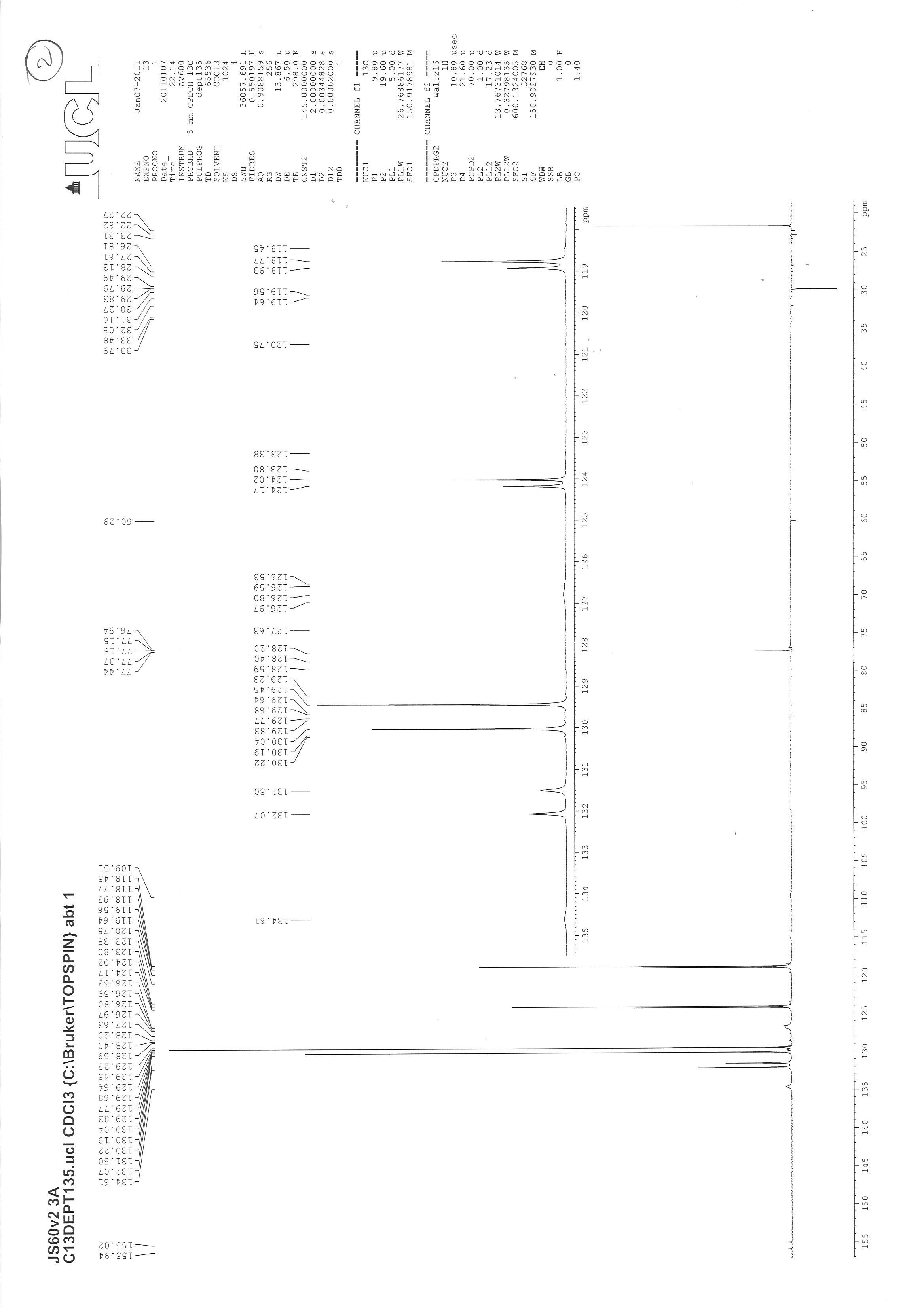
**

**4-Methyl-*N*-[2-phenoxyphenyl)imidazo[1,2-*a*]pyrazine-8-yl]benzenesulfonamide, 2**

**
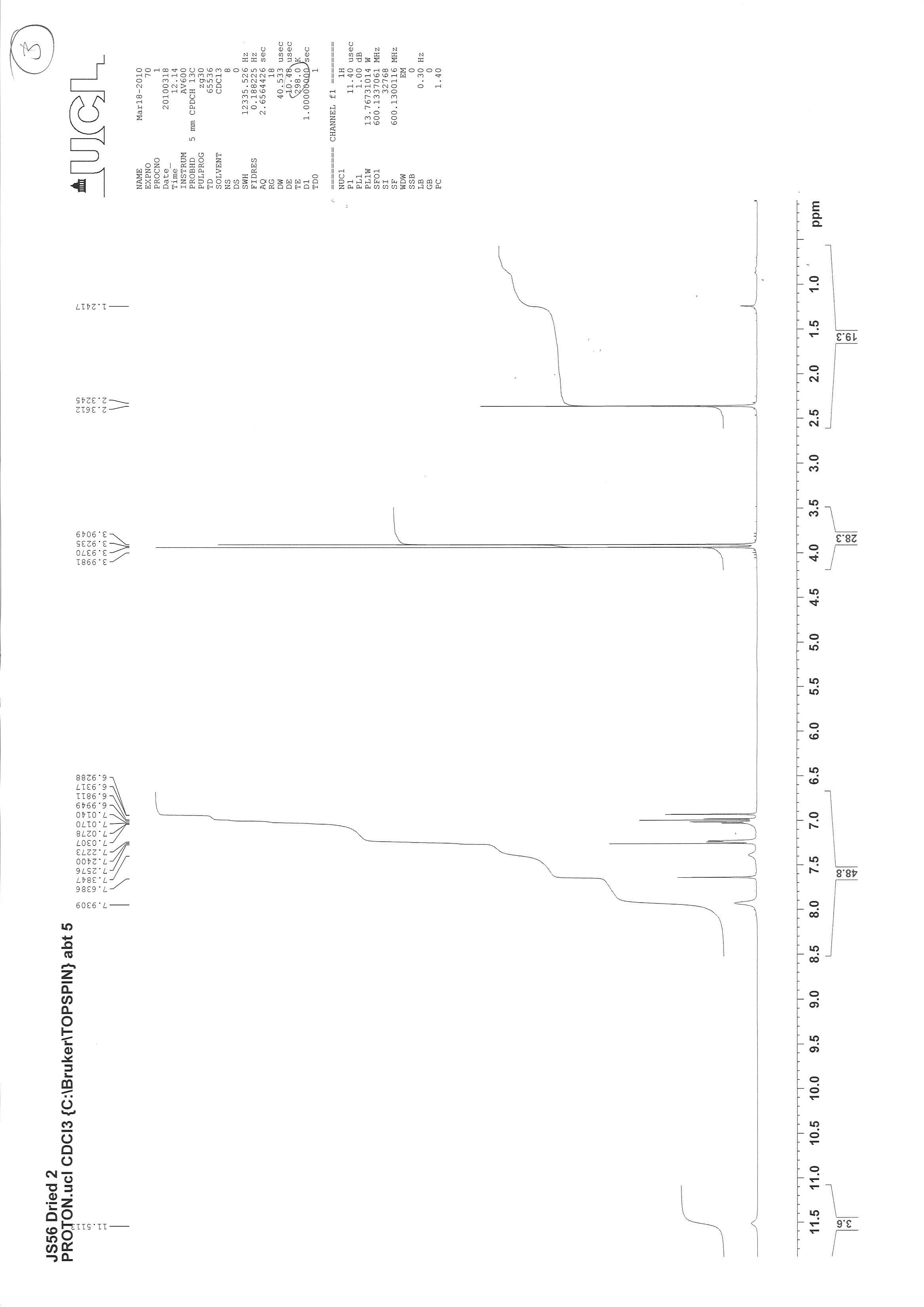
*N*-[3-(3,4-Dimethoxyphenyl)imidazo[1,2-*a*]pyrazine-8-yl]-4-methyl-benzene­sulfonamide, 3**

**
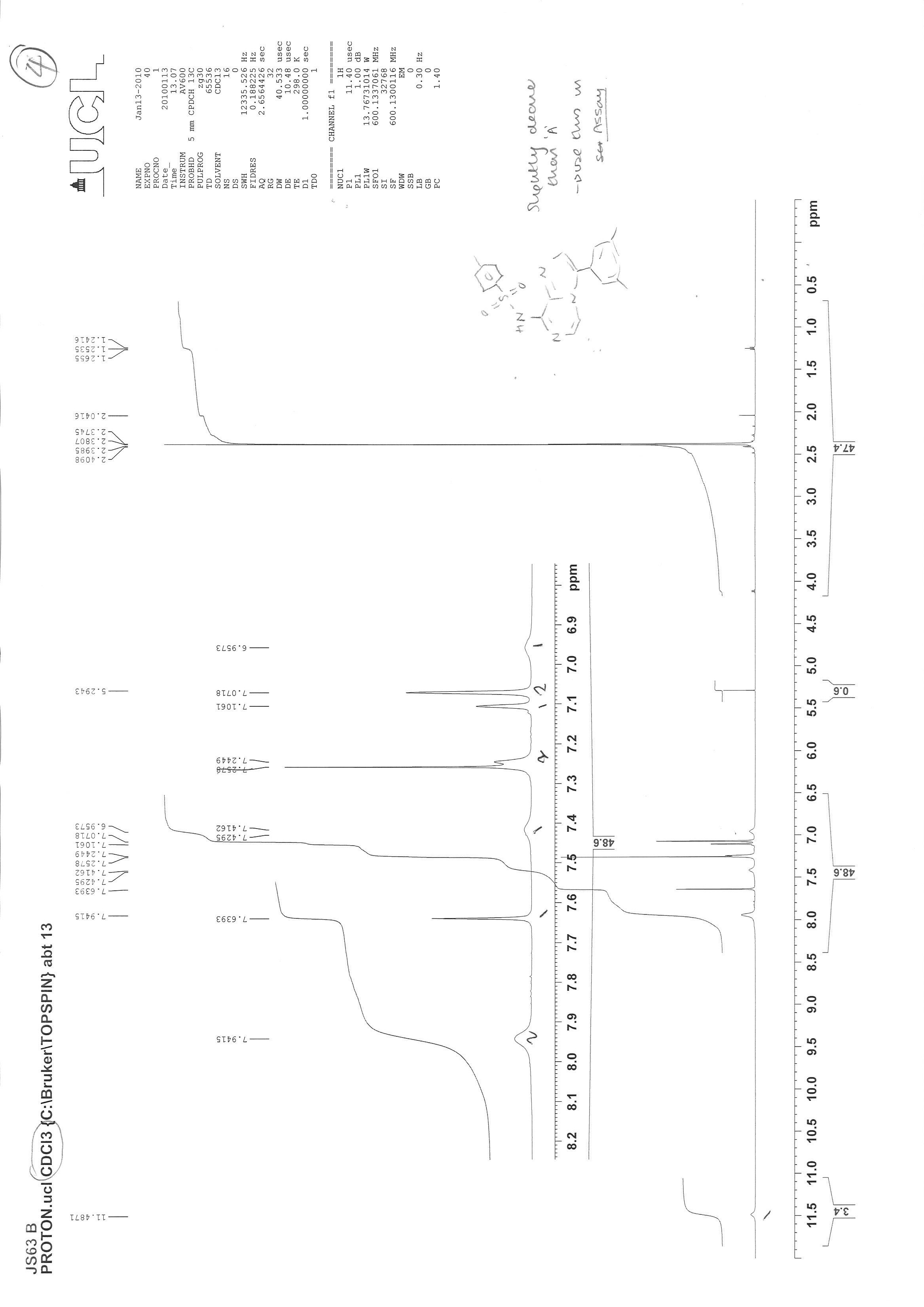
*N*-[3,5-Dimethylphenyl)imidazo[1,2-*a*]pyrazine-8-yl]-4-methyl-benzenesulfonamide, 4**

**
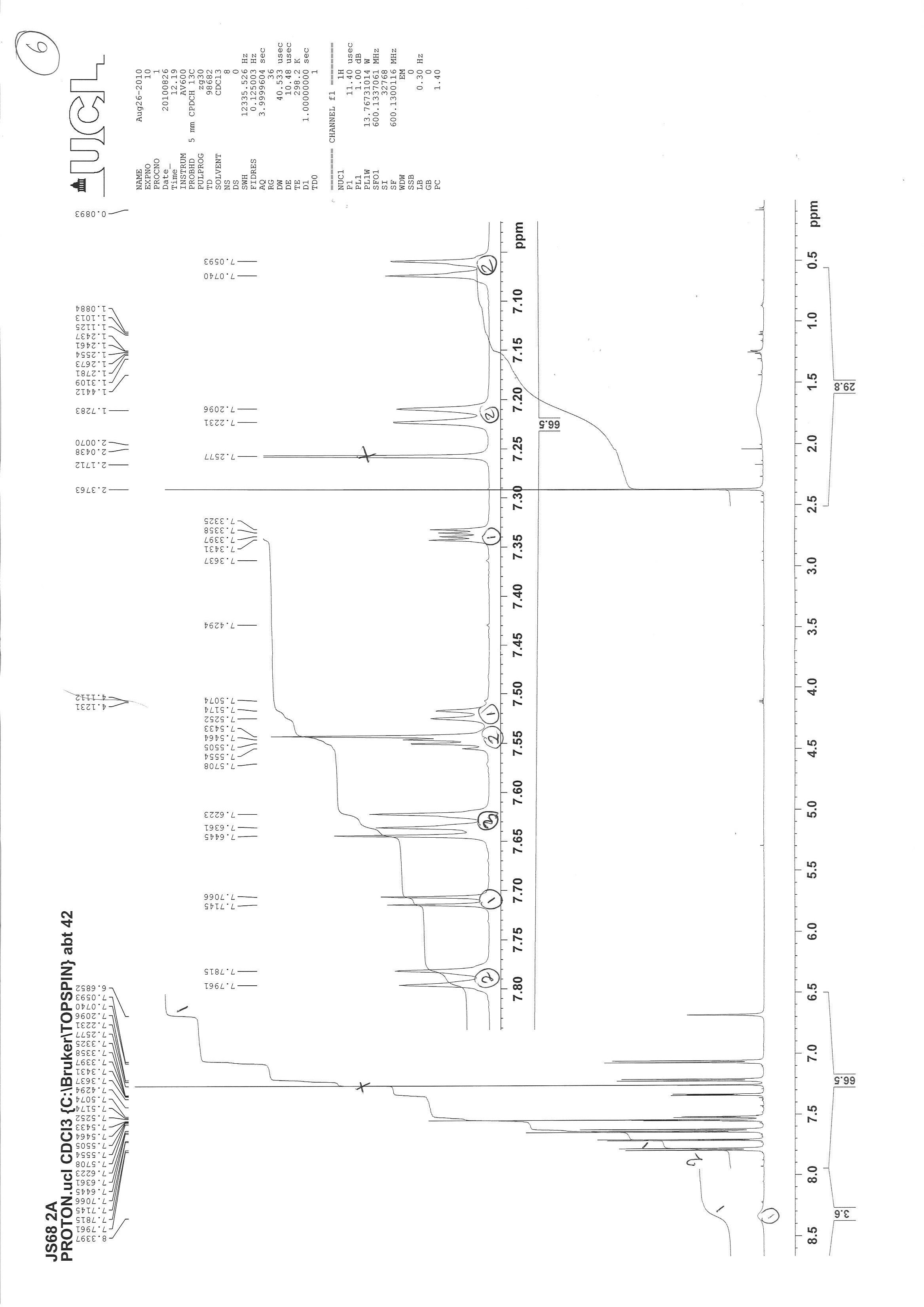
4-Methyl-*N*-[4-[[3-(3-thienyl)imidazo[1,2-*a*]pyrazine-8-yl]amino]phenyl]benzene­sulfonamide, 6**

**
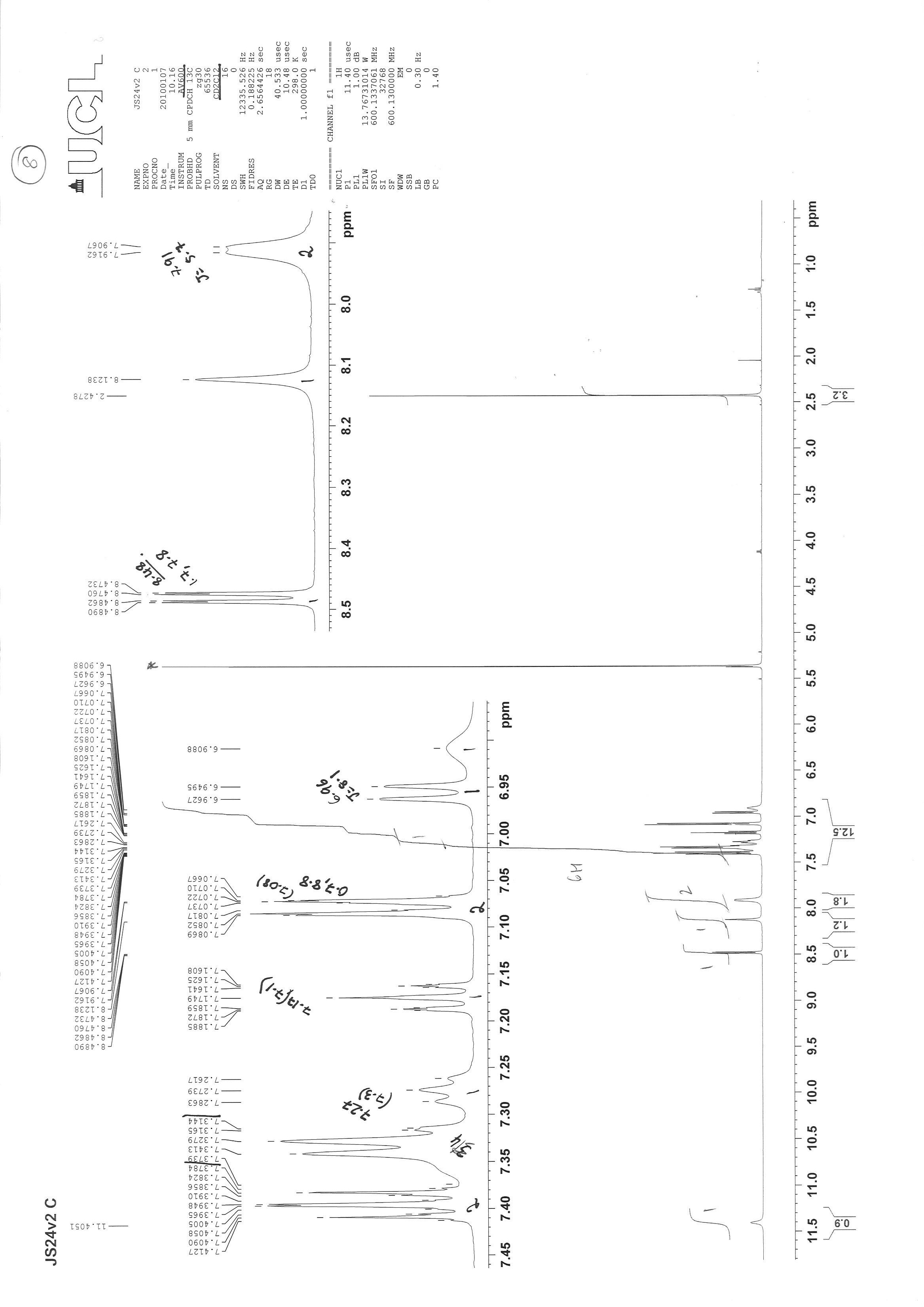
4-Methyl-*N*-[2-(2-phenoxyphenyl)imidazo[1,2-*a*]pyrazine-8-yl]benzenesulfonamide, 8**

**
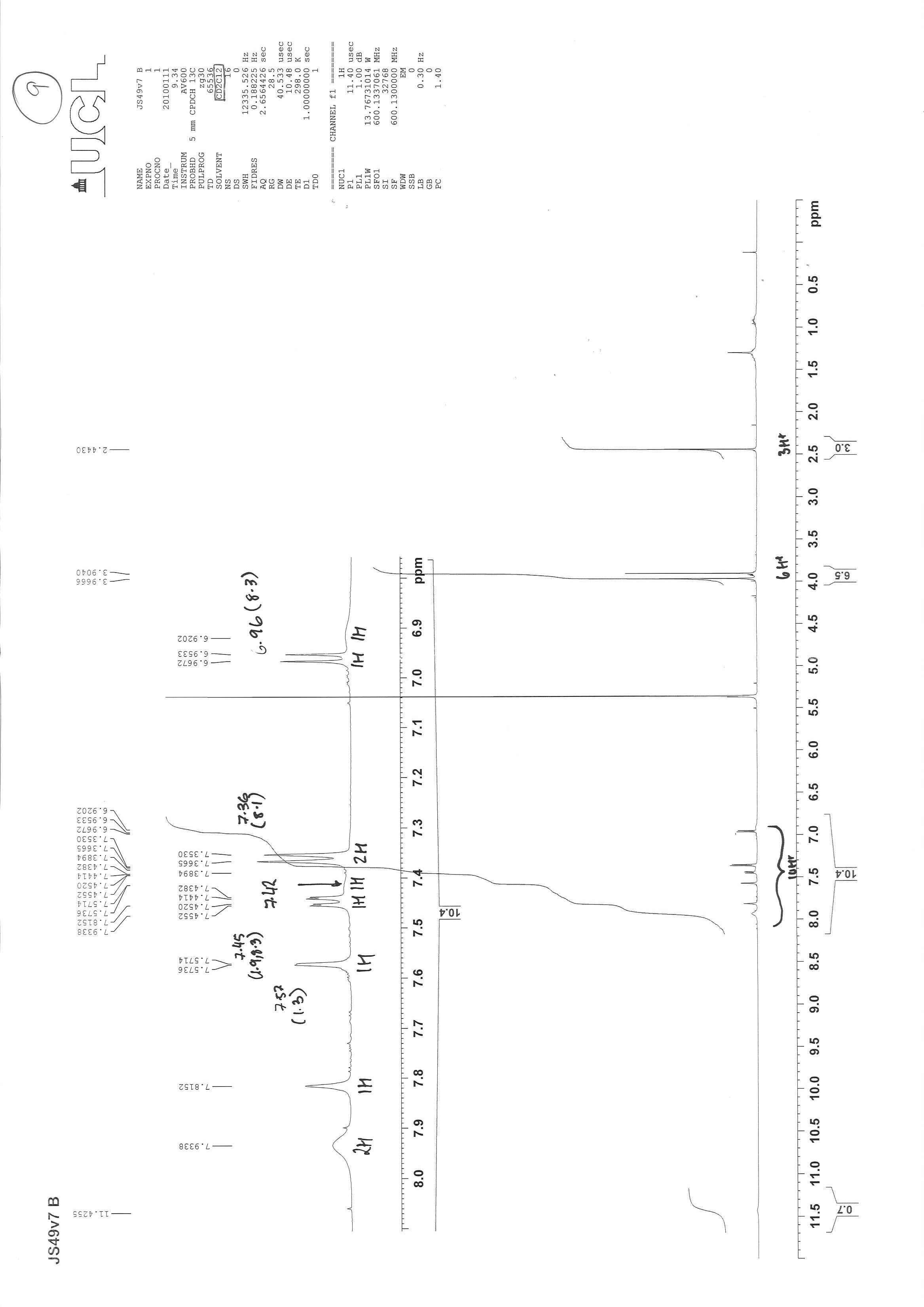
*N*-[2-(3,4-Dimethoxylphenyl)imidazo[1,2-*a*]pyrazine-8-yl]-4-methyl-benzene­sulfonamide, 9**

**
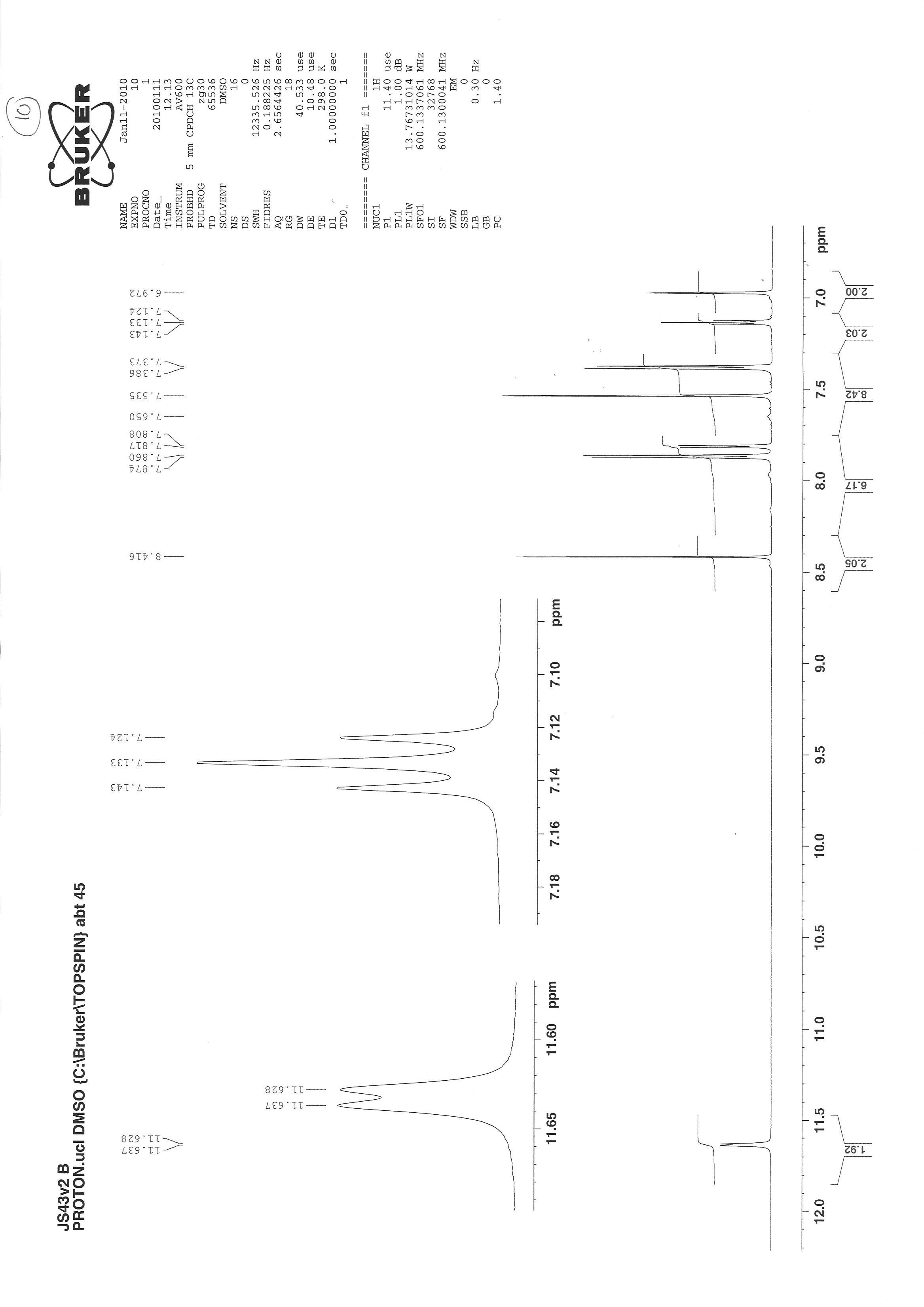
*N*-[2-(3,5-Dimethylphenyl)imidazo[1,2-*a*]pyrazine-8-yl]-4-methylbenzene­sulfonamide, 10**

**
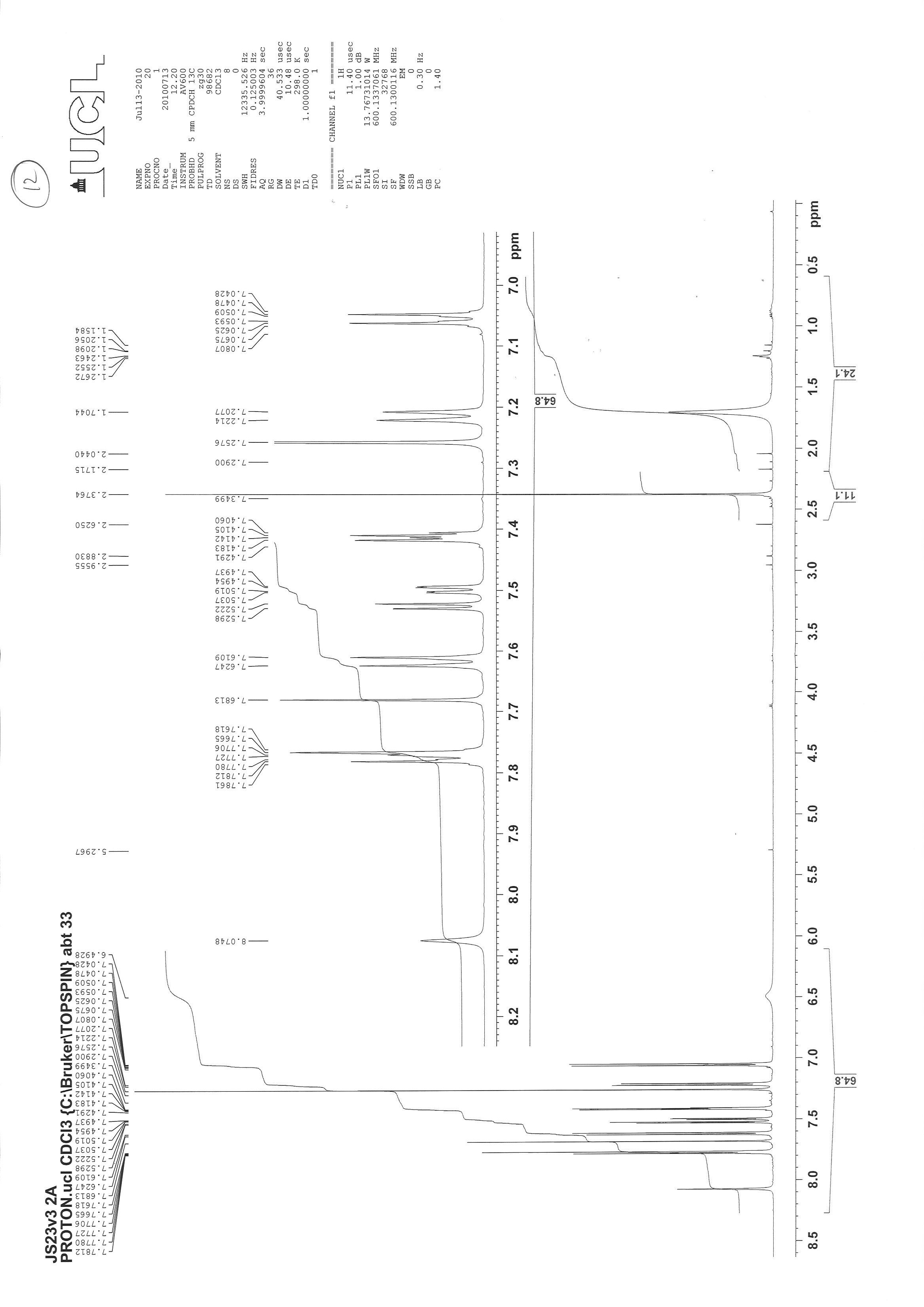
4-Methyl-*N*-[4-[[2-(3-thienyl)imidazo[1,2-*a*]pyrazine-8-yl]amino]phenyl]benzene­sulfonamide, 12**

**
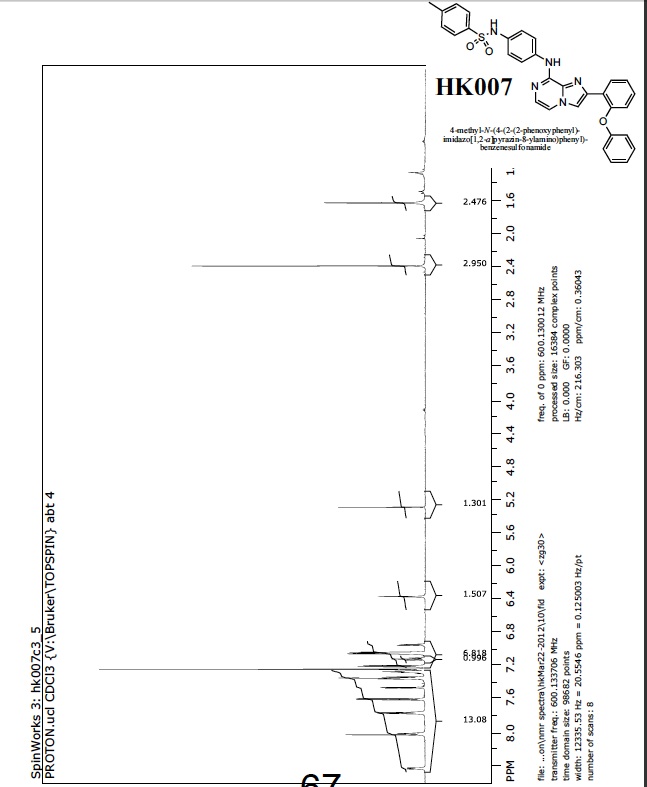
**

### **4-Methyl-N-(4-(2-(2-phenoxyphenyl)imidazo[1,2-a]pyrazin-8-ylamino)phenyl)­benzene­sulfonamide 13**


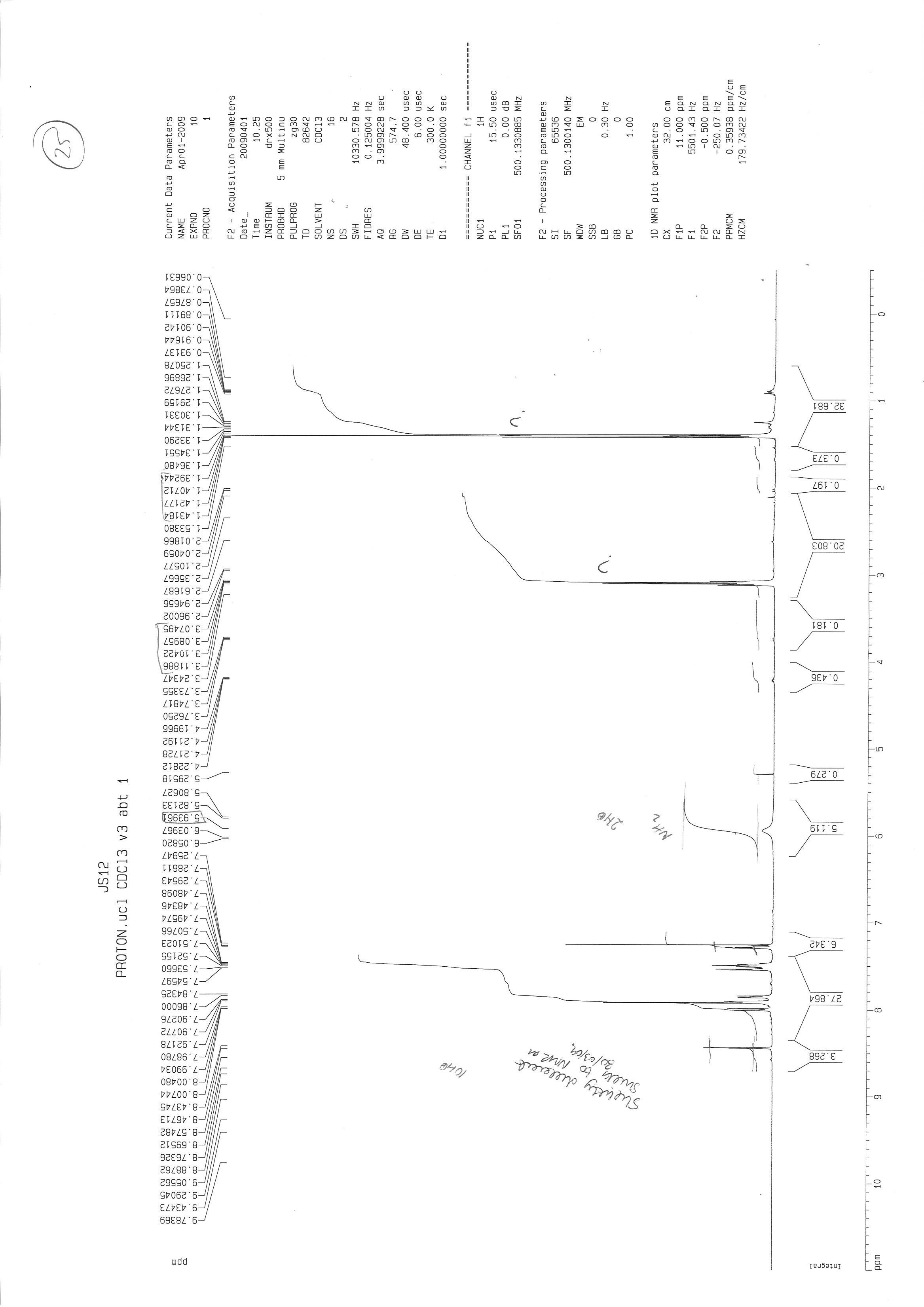


**2-(Naphthalen-2-yl)imidazo[1,2-*a*]pyrazin-8-amine (25)**


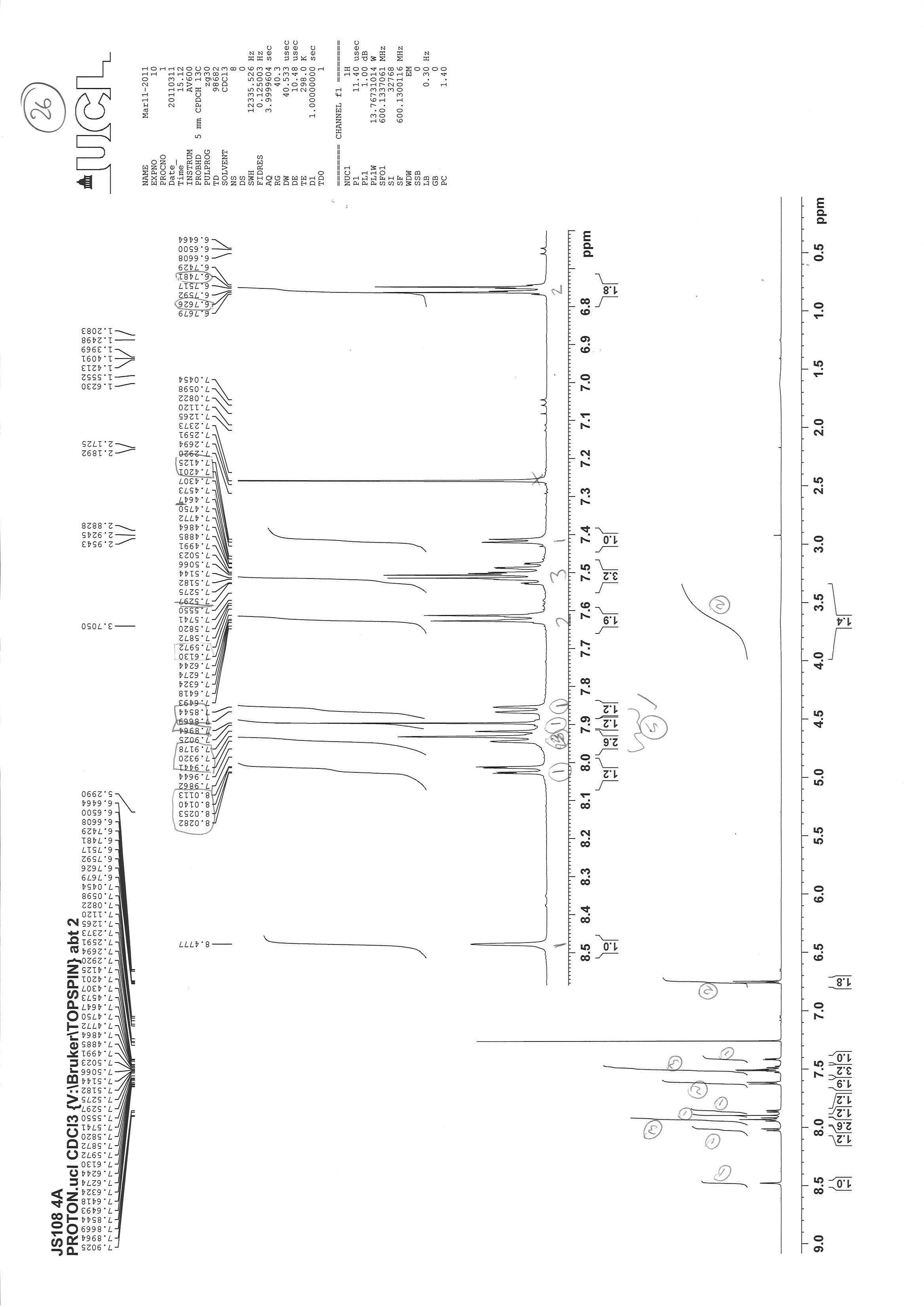


***N*’-(2-(Naphthalen-2-yl)imidazo[1,2-*a*]pyrazin-8-yl)benzene-1,4-diamine (26)**


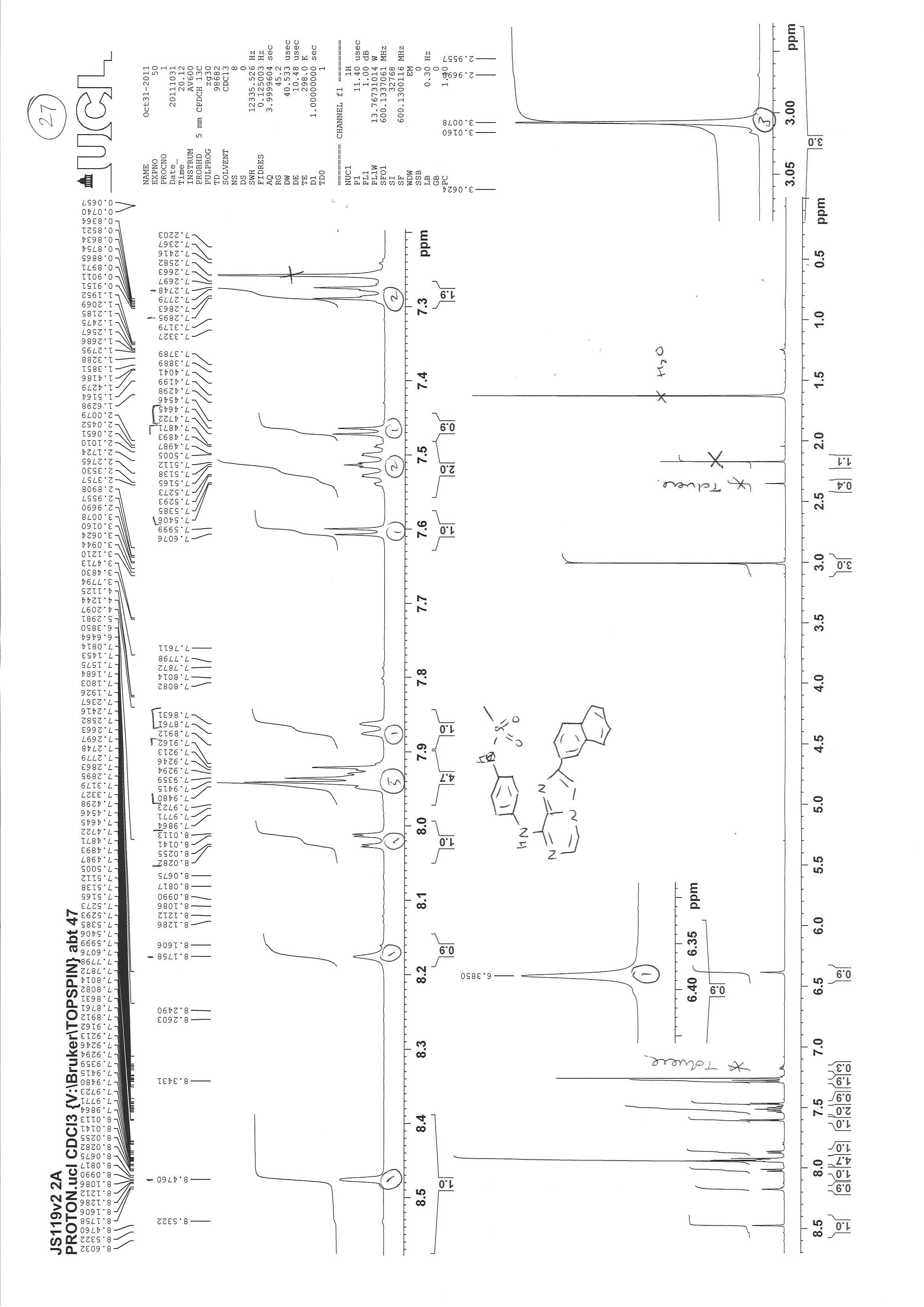
***N*-(4-(2-(Naphthalen-2-yl)imidazo[1,2-*a*]pyrazin-8-ylamino)phenyl)methane­sulfon­amide (27)**

**
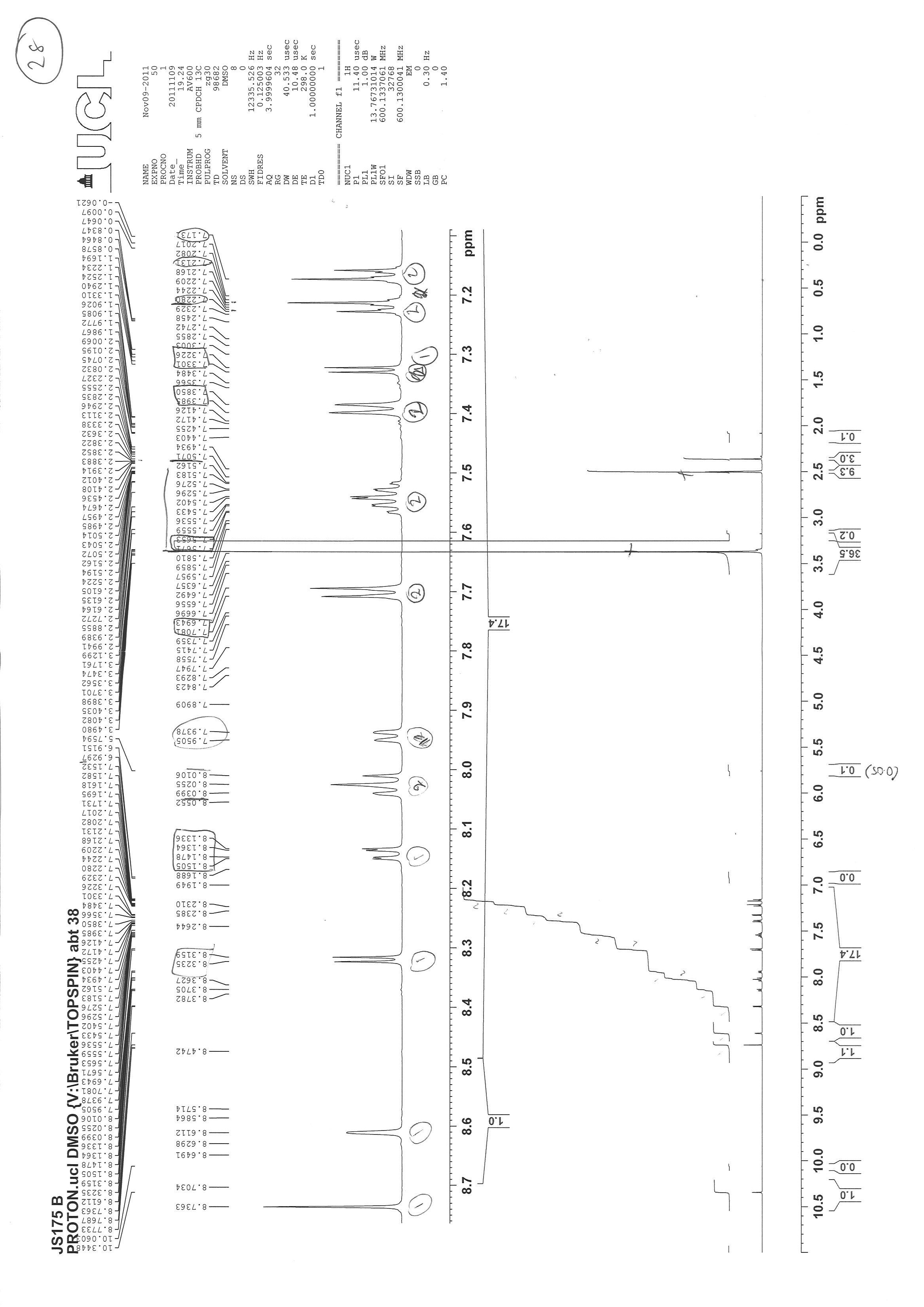
4-Methyl-*N*-(4-(2-(naphthalen-2-yl)imidazo[1,2-*a*]pyrazin-8-yloxy)phenyl)benzene­sulfonamide (28)**

**
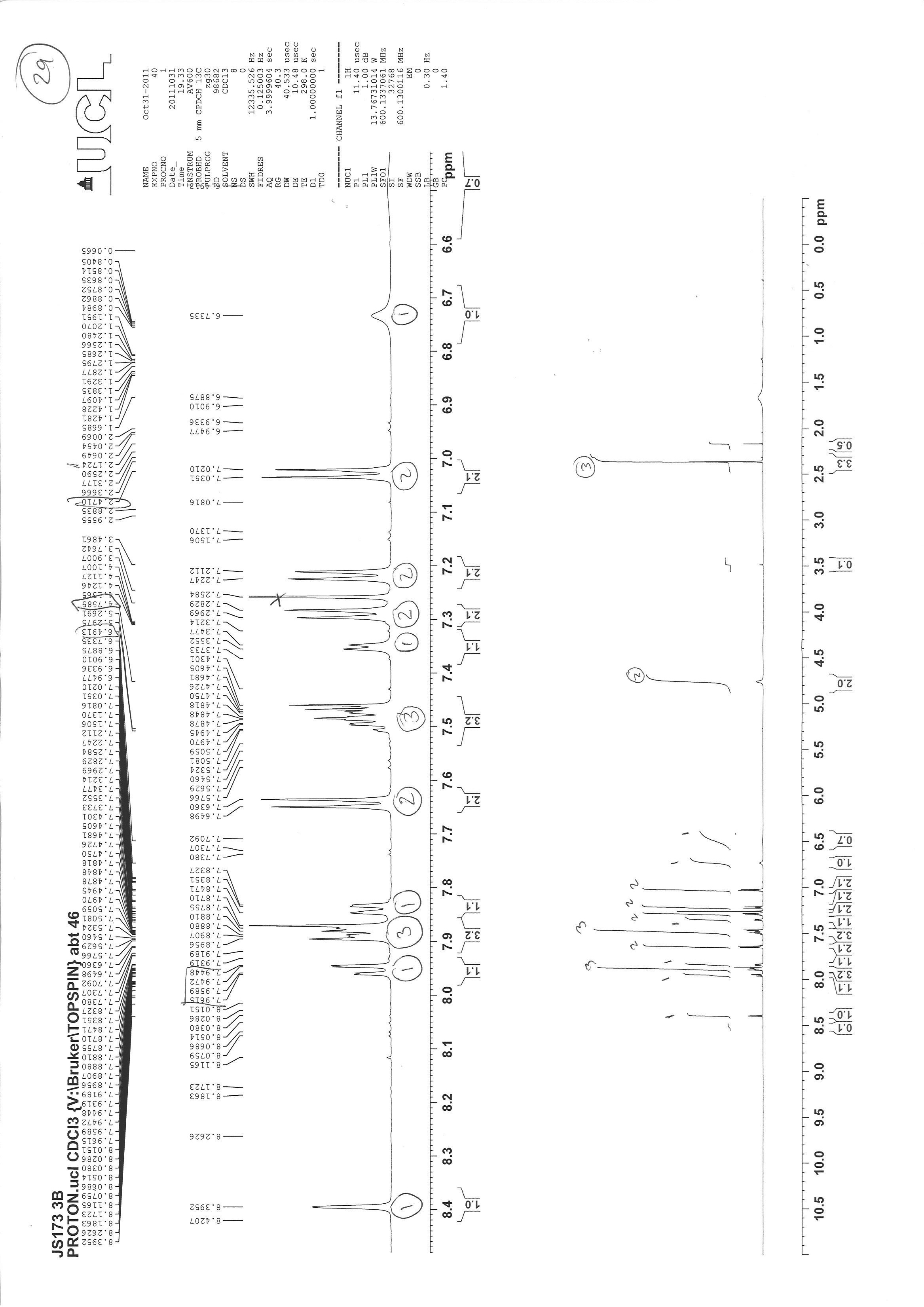
4-Methyl-*N*-(4-((2-(naphthalen-2-yl)imidazo[1,2-*a*]pyrazin-8-ylamino)methyl)­phenyl)­­benzene­­sulfonamide (29)**

**
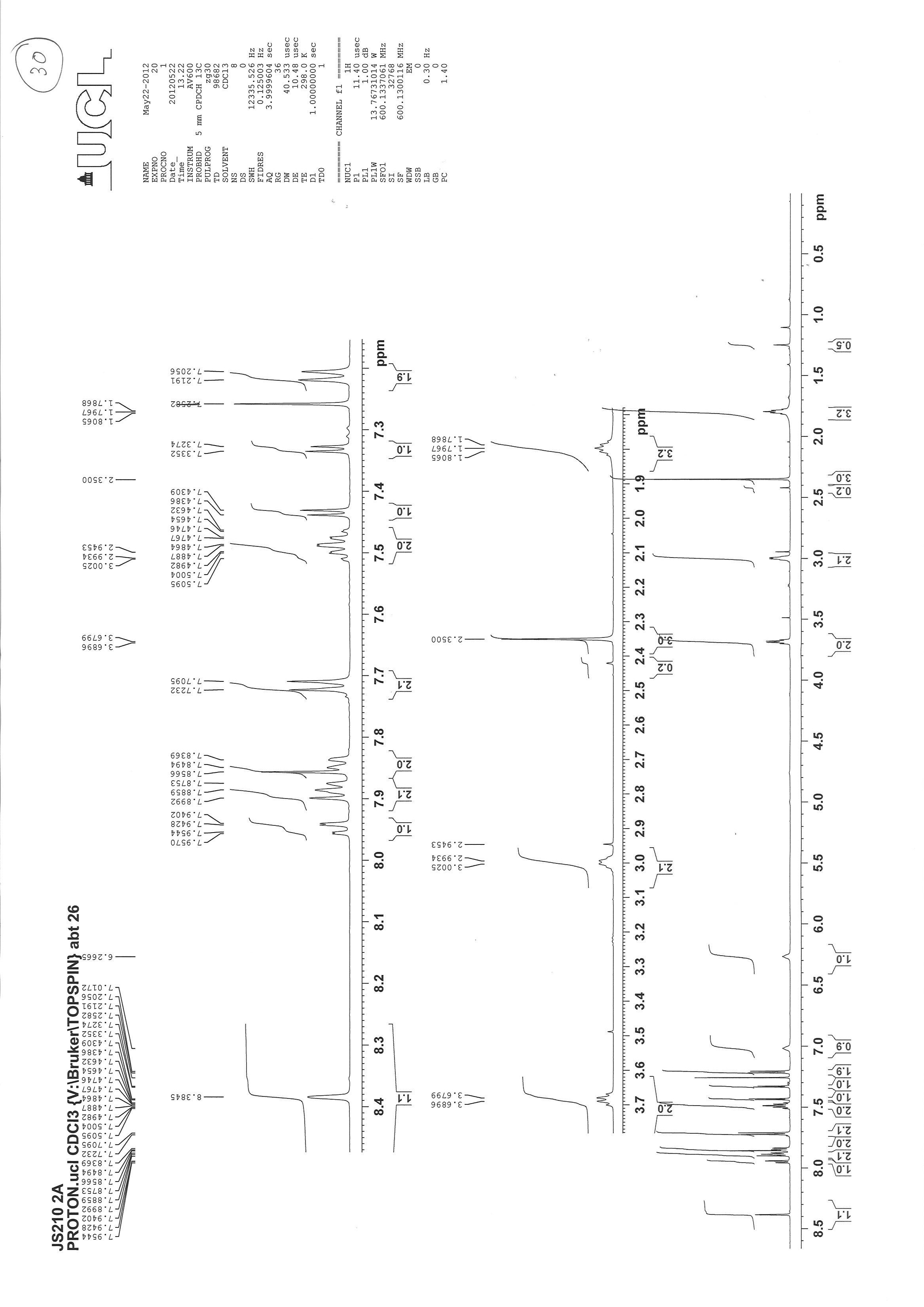
4-Methyl-*N*-(3-(2-(naphthalen-2-yl)imidazo[1,2-*a*]pyrazin-8-ylamino)propyl)­benzene­sulfonamide (30)**

**
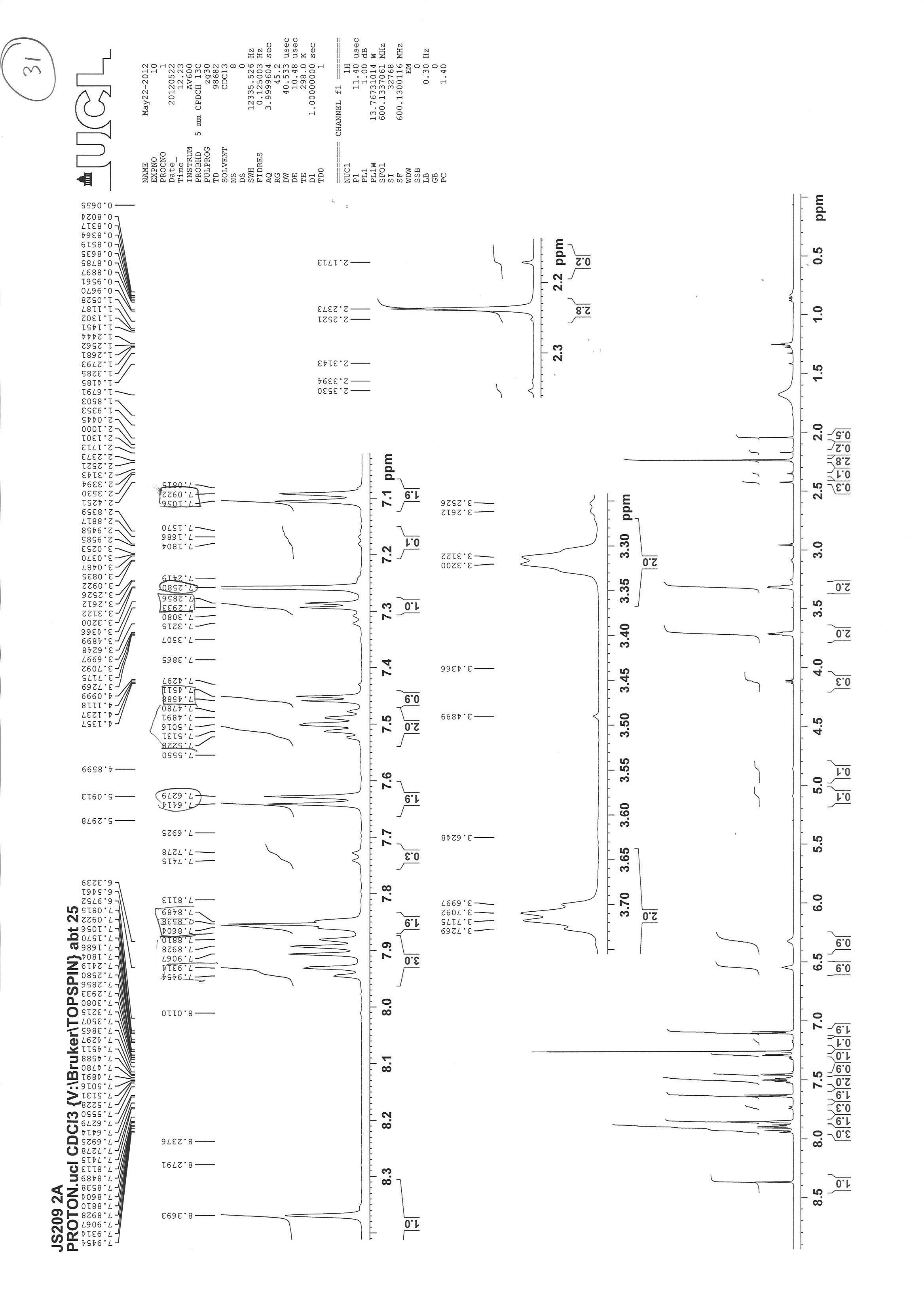
4-Methyl-*N*-(2-(2-(naphthalen-2-yl)imidazo[1,2-*a*]pyrazin-8-ylamino)ethyl­)­benzene­sulfonamide­ (31)**

**
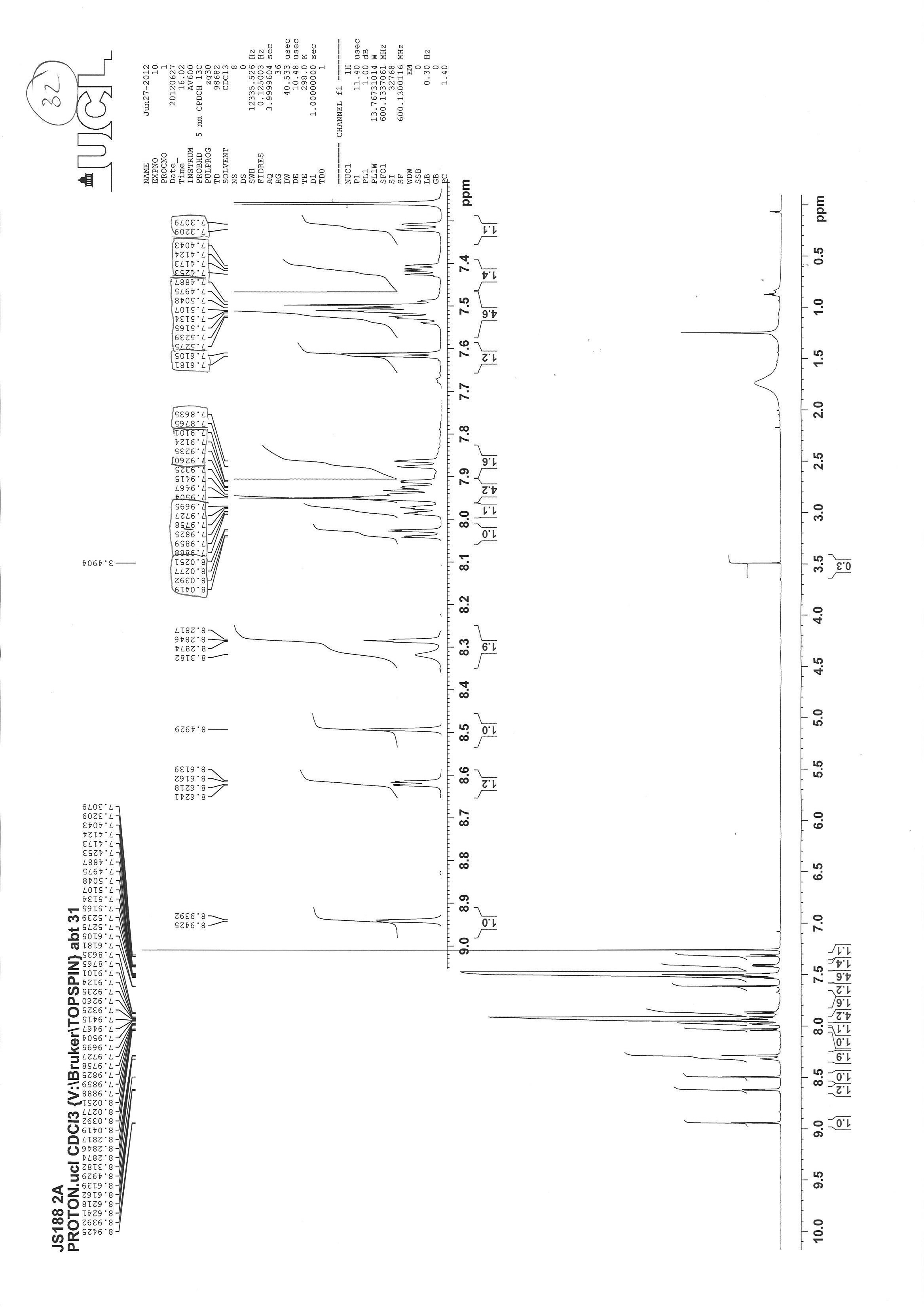
**

**2-(Naphthalen-2-yl)-*N*-(3-(pyridin-3-yl)phenyl)imidazo[1,2-*a*]pyrazin-8-amine (32)**

**
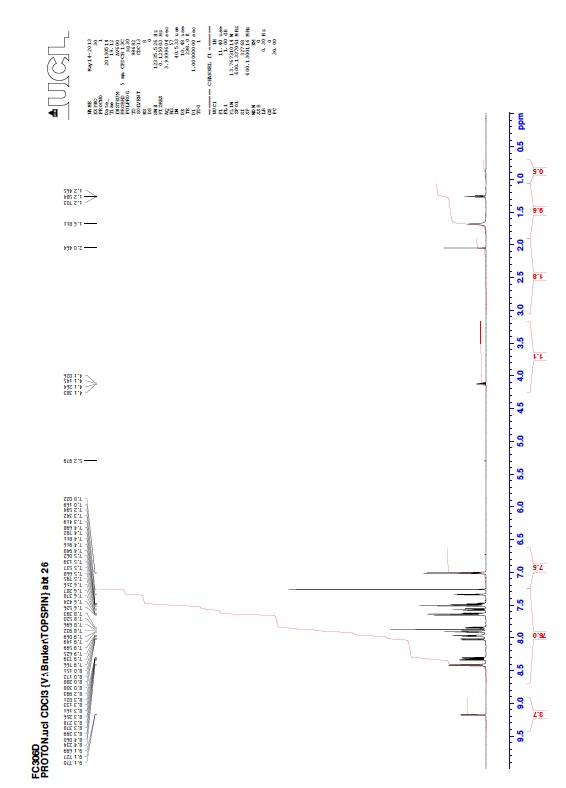
**

***N*-(4-((2-(Naphthalen-2-yl)imidazo[1,2-*a*]pyrazin-8-yl)amino)phenyl)quinoline-8-sulfonamide (33)**

**
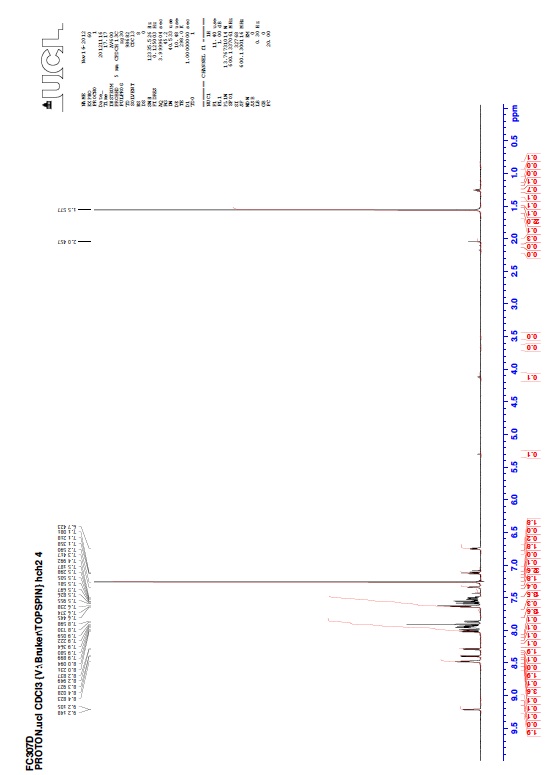
*N*-(3-((2-(Naphthalen-2-yl)imidazo[1,2-*a*]pyrazin-8-yl)amino)phenyl)quinoline-8-sulfonamide (34)**

**
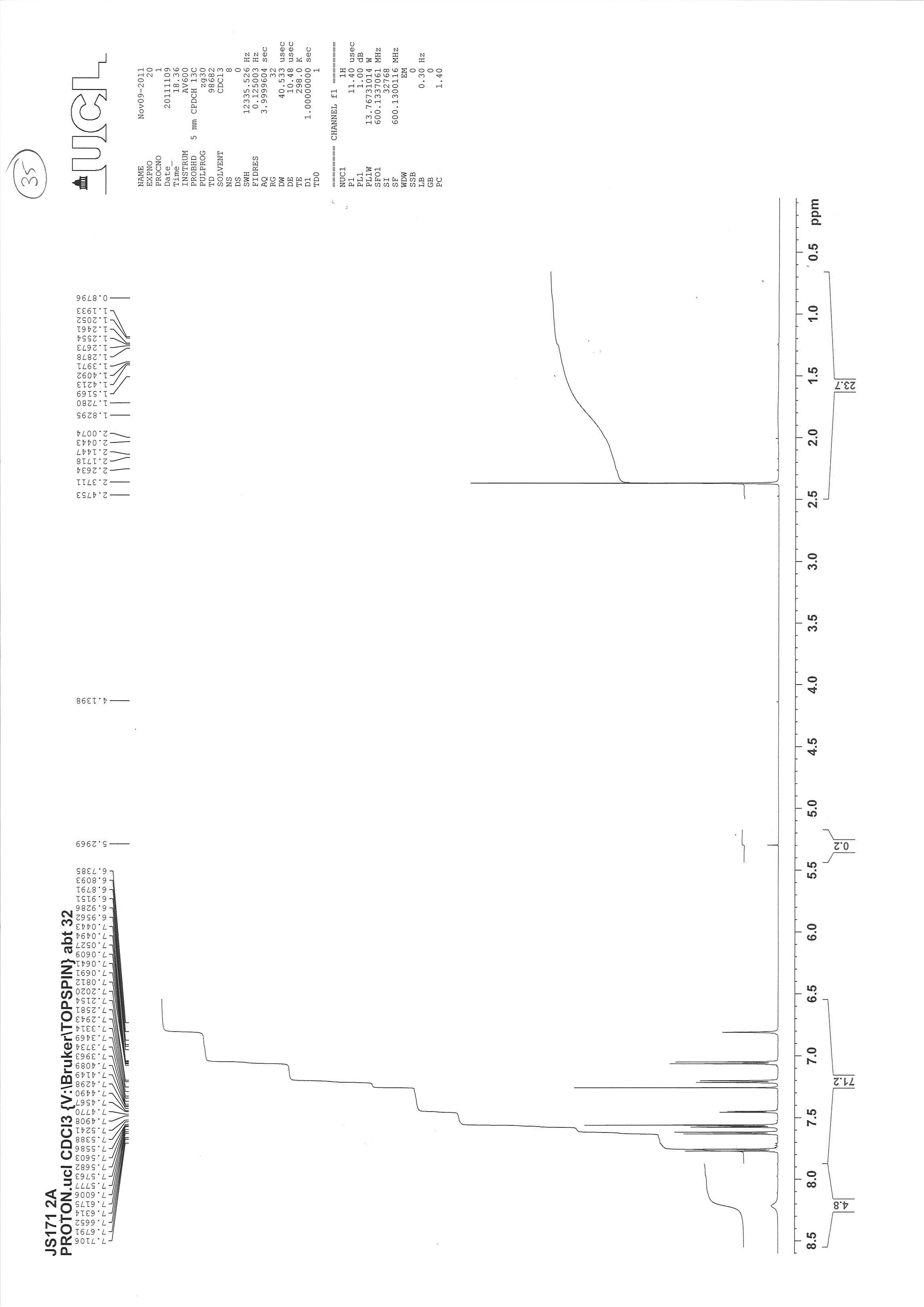
**

***N*-(4-(Imidazo[1,2-*a*]pyrazin-8-ylamino)phenyl)-4-methylbenzenesulfonamide (35)**

**
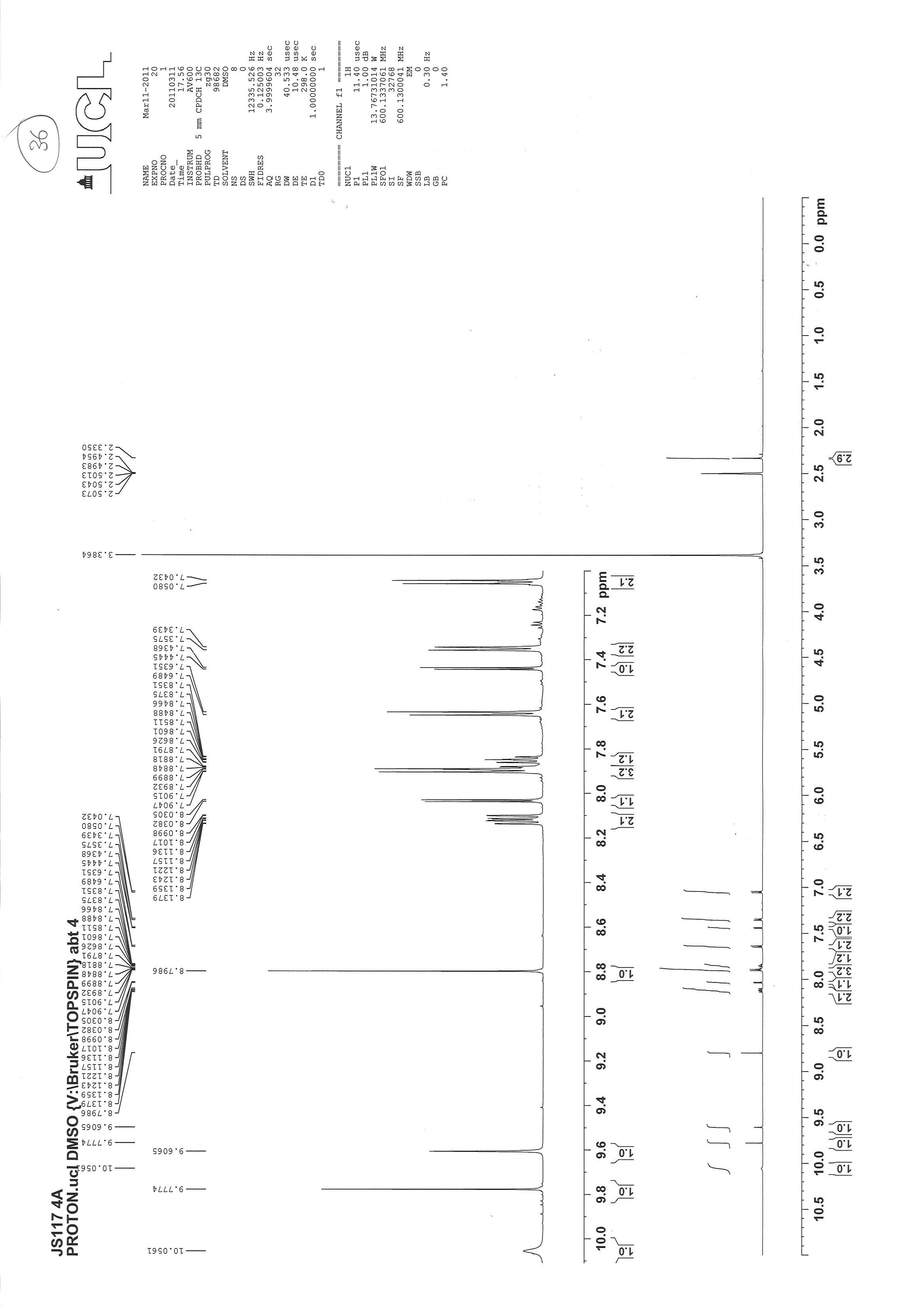
4-Methyl-*N*-(4-(2-(quinoxalin-2-yl)imidazo[1,2-*a*]pyrazin-8-ylamino)phenyl)­benzene­sulfonamide (36)**

**
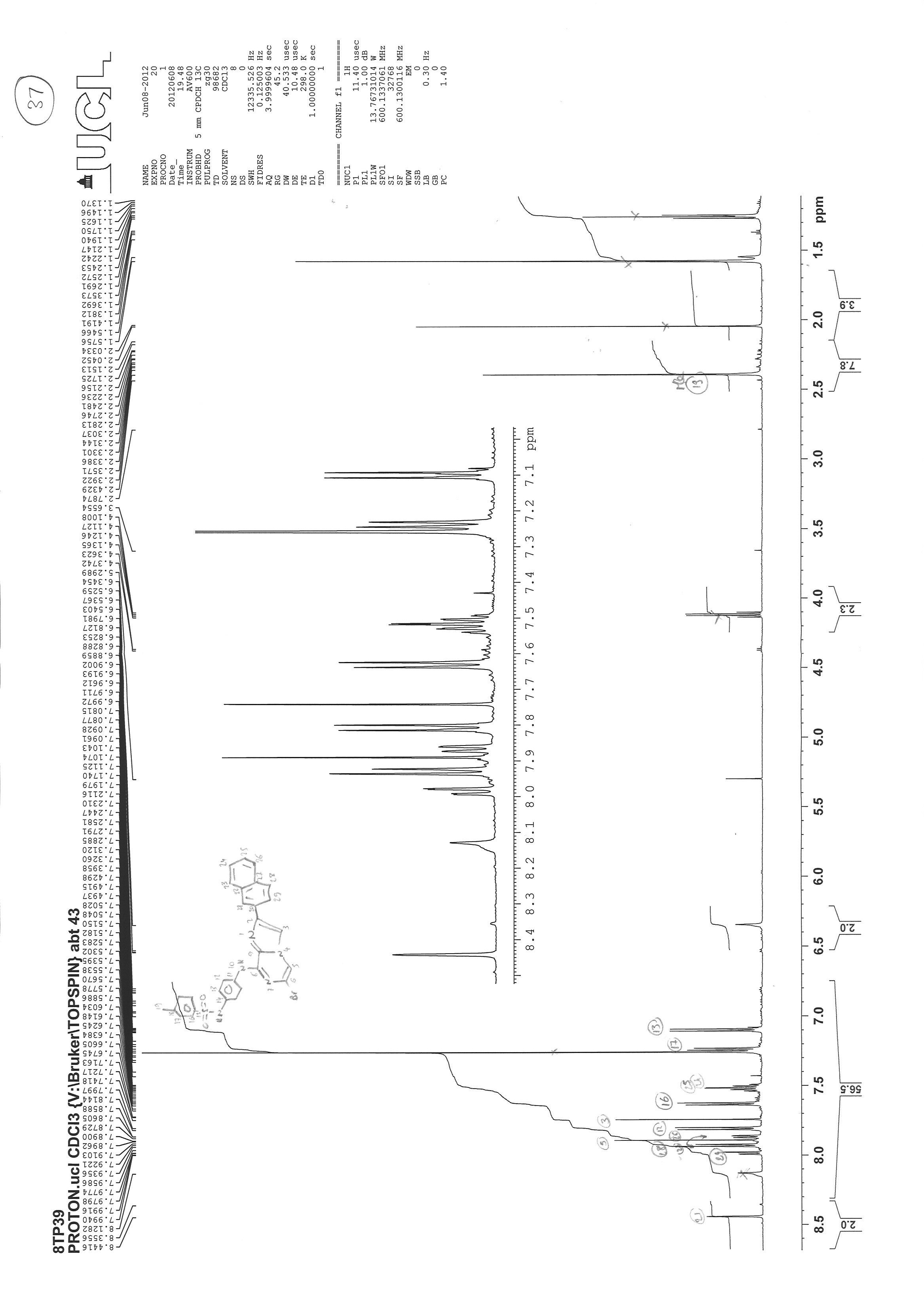
*N*-(4-((6-Bromo-2-(naphthalen-2-yl)imidazo[1,2-*a*]pyrazin-8-yl)amino)phenyl)-4- methylbenzenesulfonamide (37)**

**
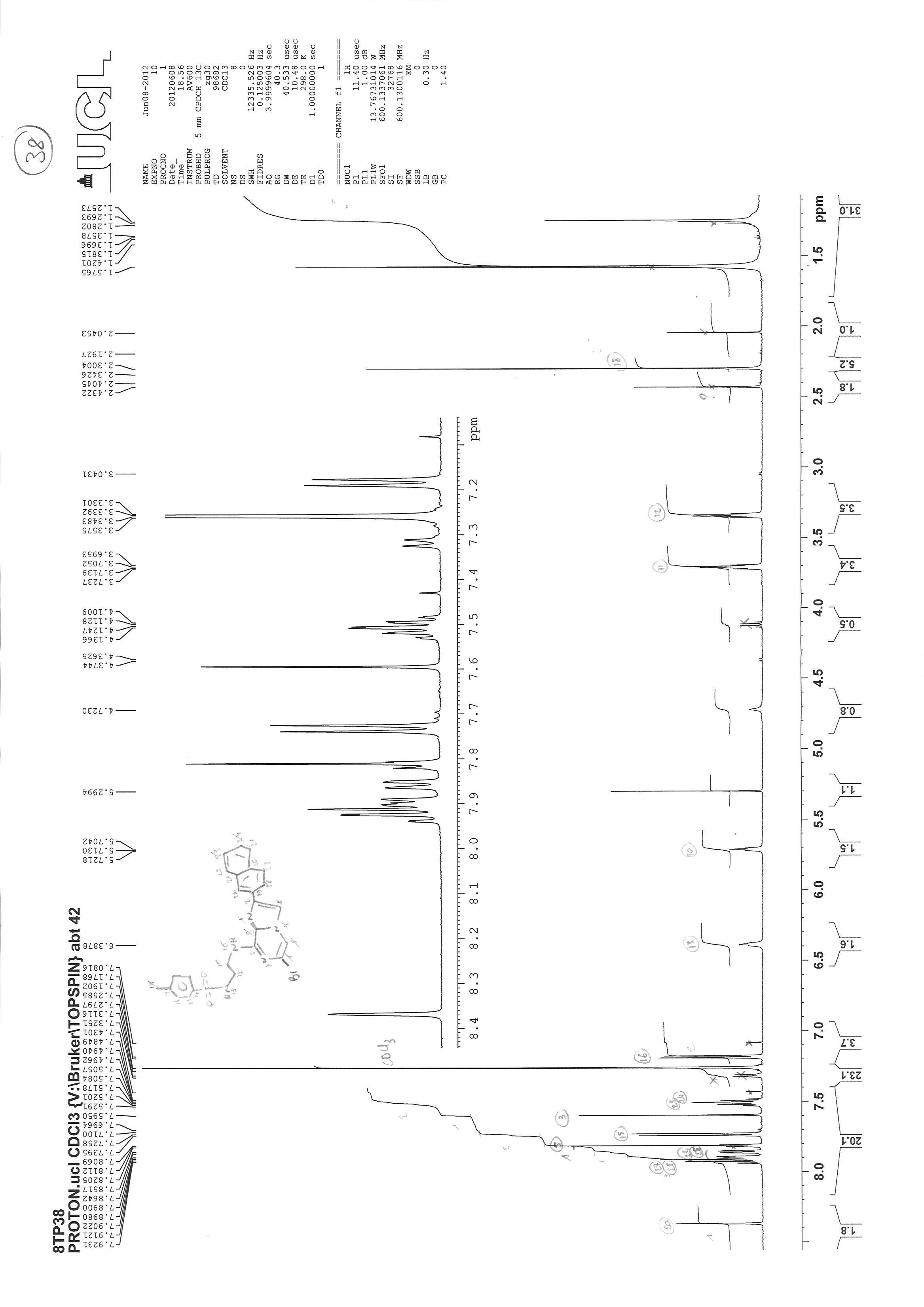
*N*-(2-((6-Bromo-2-(naphthalen-2-yl)imidazo[1,2-*a*]pyrazin-8-yl)amino)ethyl)-4- methylbenzenesulfonamide (38)**

**References**

1. Pangborn, A. B.; Ciardello, M. A.; Grubbs, R. H.; Rosen, R. K. and Timmers, F. J., Safe and Convenient Procedure for Solvent Purification, *Organometallics,* **1996**, *15,* 1518-1520.
2. Koufaki, M.; Kiziridi, C.; Nikoloudaki, F. and Alexis, M. N., Design and Synthesis of 1,2-dithiolane Derivatives and Evaluation of their Neuroprotective Activity, *Bioorg. Med. Chem. Lett.,* **2007**, *17*, 4223 - 4227.
3. Gong, P.K.; Blough, B.E.; Brieaddy, L.E.; Huang, X.; Kuhar, M.J.; Navarro, H.A. and Carroll, I., Synthesis, Monoamine Transporter Binding, Properties, and Functional Monoamine Uptake Activity of 3*â*-[4-Methylphenyl and 4-Chlorophenyl]-2*â*-[5-(Substituted phenyl)thiazol-2-yl]tropanes, *J. Med. Chem*., **2007**, *50*, 3686-3695.
4. Von Roman, U., Ruhdorfer, J., Knorr, R., Alkenyl Bromides by Brominative Deoxygenation of Ketones in One or Two Steps, *Synthesis*, **1993**, *10*, 985-992.
5. La Regina, G.; Bai, R.; Rensen, W.; Coluccia, A.; Piscitelli, F.; Gatti, V.; Bolognesi, A.; Lavecchia, A.; Granata, I.; Porta, A.; Maresca, B.; Soriani, A.; Iannitto, M. L.; Mariani, M.; Santoni, A.; Brancale, A.; Ferlini, C.; Dondio, G.; Varasi, M.; Mercurio, C.; Hamel, E.; Lavia, P., Novellino, E. and Silvestri, R., Design and Synthesis of 2-Heterocyclyl-3-arylthio-1*H*-indoles as Potent Tubulin Polymerisation and Cell Growth Inhibitors with Improved Metabolic Stability, *J. Med. Chem.,* **2011**, *54*, 8394-8406.
6. Ina, S.; Yamana, K and Noda, K., Use of 2-Phenylmorpholin-5-one Derivatives, US Patent Appl. US6265402, Jul 24, 2001.
7. Dragovich, P. S.; Murphy, D. E., Tran, C. V. and Ruebsam, F., Efficient Large-Scale Synthesis of 2-Amino-5-Methanesulfonylaminobenzenesulfonamide, *Synth. Commun*., **2008**, *38*, 1909-1916.
8. Jackson, S. K.; Banfield, S. C. and Kerr, M. A., Total Synthesis of (±)-Herbindole B and (±)-*cis*-Trikentrin B, *Org Lett*, **2005**, *7*, 1215-1218.
9. Manolikakes, G.; Hernandez, C. M.; Schade, M. A.; Metzger, A.; Knochel, P., Palladium- and Nickel- Catalysed Cross-Couplings of Unsaturated Halides Bearing Relatively Acidic Protons with Organozinc Compounds, *J. Org. Chem.,* **2008**, *73*, 8422-8436.
10. Rowe, D. J.; Garner, C. D. and Joule, J. A., The Preparation of 1-Aryl- and 1-Heteroaryl-alkene-1,2-dithiolates, *J. Chem. Soc. Perkin Trans. 1,* **1985**, 1907-1910.
11. Trott, O.; Olson, A. J. *J. Comput. Chem.,* **2010**, *31*, 455.
12. Morris, G. M.; Huey, R.; Lindstrom, W.; Sanner, M. F.; Goodsell, D. S. and Olson, A. J., *J. Comput. Chem.,* **2009**, *30*, 2785.

**ATPase assay.**

ATPase activity measurements were carried out using a colorimetric *in vitro* ATPase assay kit (Innova Biosciences), using 96-well ELISA microplates (Greiner Bio-One) with reaction volumes of 100 μL in each well. Multi-pipettes with either 8 tips or 96 tips were used. Enzymatic reactions were performed for 30 min at 37 ºC followed by measuring the absorbance at 620 nm, detecting the presence of inorganic phosphate product. Each reaction contained 100 mM Tris–HCl, pH 7.5; 2.5 mM MgCl_2_; 125 μM ATP; 5% DMSO; 25 mM NaCl; 0.5 mM DTT and 0.053 μM HP0525, with or without inhibitor. In detail, the assay can be described in steps. Step 1: 48 μL of substrate buffer mix (providing the components Tris–HCl, pH 7.5; MgCl_2_; ATP and DMSO) was dispensed to each well. Step 2: 2 μL of compound (solublized in DMSO) was added to corresponding well. Step 3: Enzymatic reaction was started by the addition of 50 μL of protein (in 50 mM Tris-HCl, pH 7.5; 50 mM NaCl and 1 mM DTT) to the wells. Step 4: Reaction plates were incubated at 37 degrees for 30 min. Step 5: The reaction was stopped by the addition of 25 uL of Gold mix reagent, followed by vigorous mixing. After 2-5 min, 10 uL of stabilizer solution was added to the reaction. Step 6: After an additional 30 min incubation at room temperature, the absorbance at 620 nm was measured using a microplate reader.

Data was normalized using non-inhibited HP0525 as positive control (100% enzyme activity) and fully inhibited enzyme as negative control (0% enzyme activity). In the negative control, the protein was added after the Gold mix, as described in the standard protocol of the Innova Biociences ATPase assay kit. As an alternative negative control, providing equivalent readouts, 10 mM EDTA could also be added at Step 2, then removing the catalytically essential Mg ions from the active site of HP0525.

Michaelis-Menten kinetics were performed under the same conditions as above but with various concentrations of ATP ranging from 0 to 500 μM, with and without 10 μM of compound 11. Negative controls for each ATP concentration were used. GraphPad Prism version 5.0d for Mac OS X (GraphPad Software, San Diego California USA, www.graphpad.com) was used for the IC_50_ calculations using the Prism equation for non-linear normalized dose-response ‘Log(Inhibitor) vs. normalized response’ with Hill slope set to 1; and for Michaelis-Menten and Lineweaver-Burk analysis.


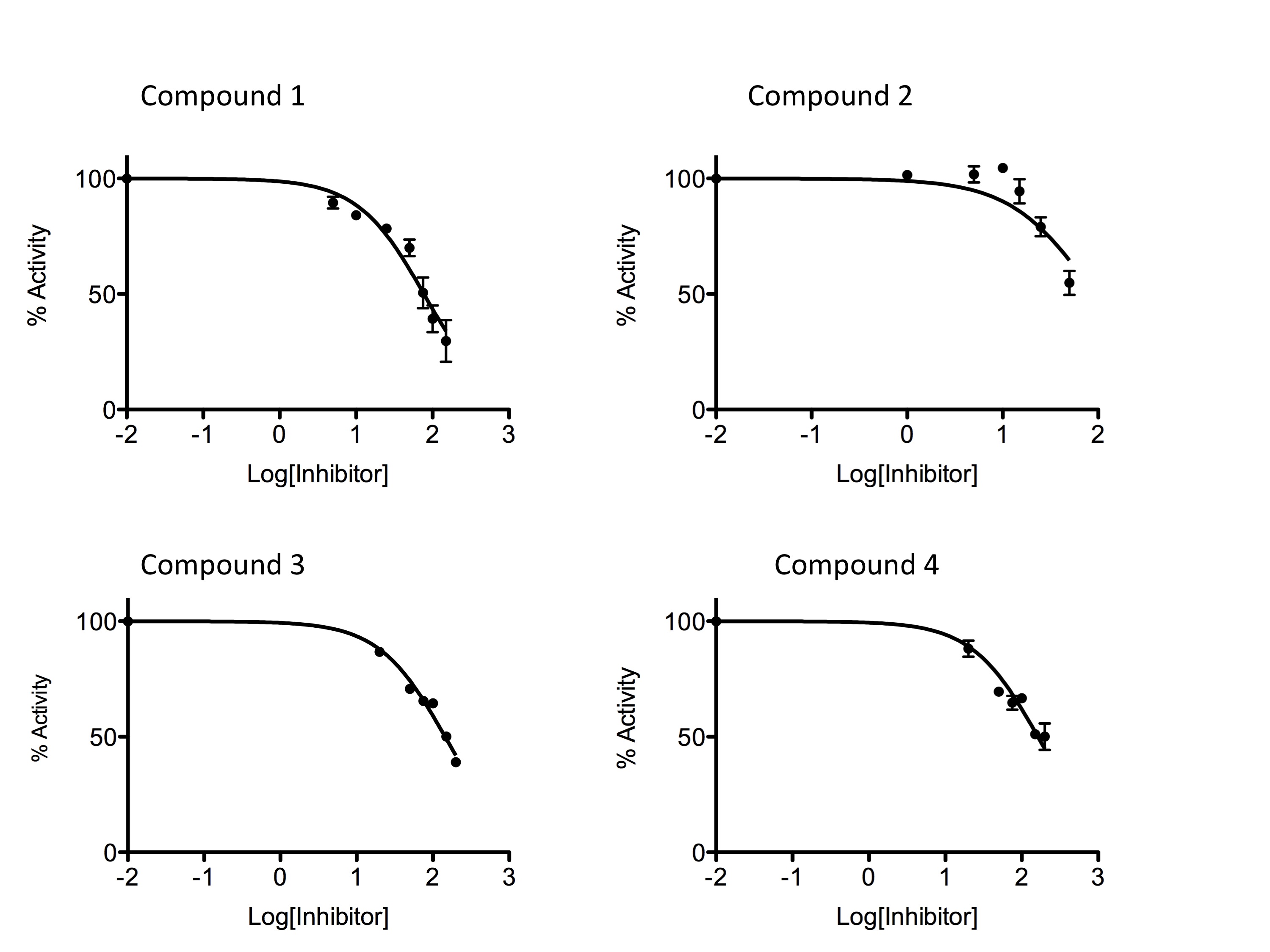


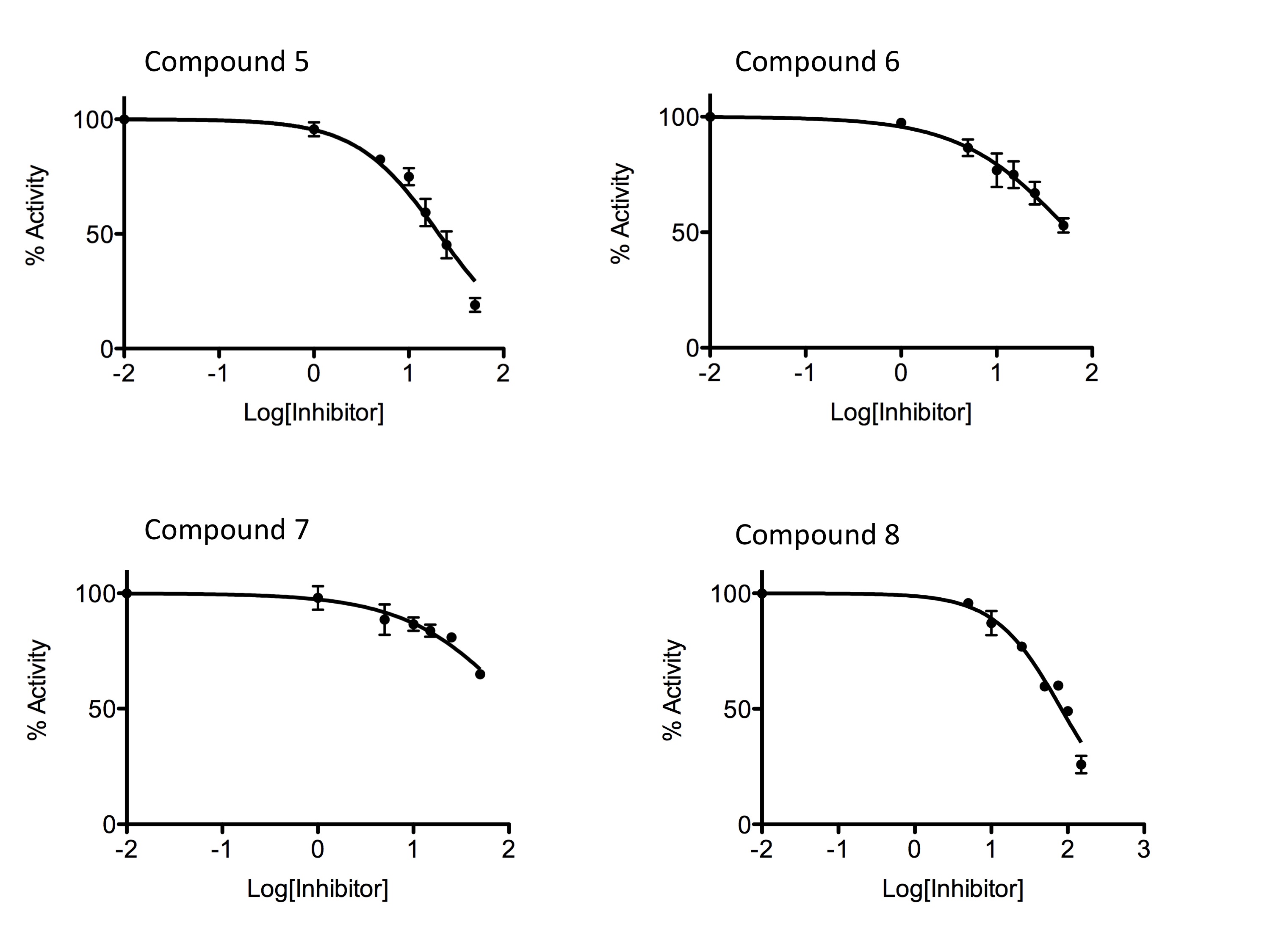


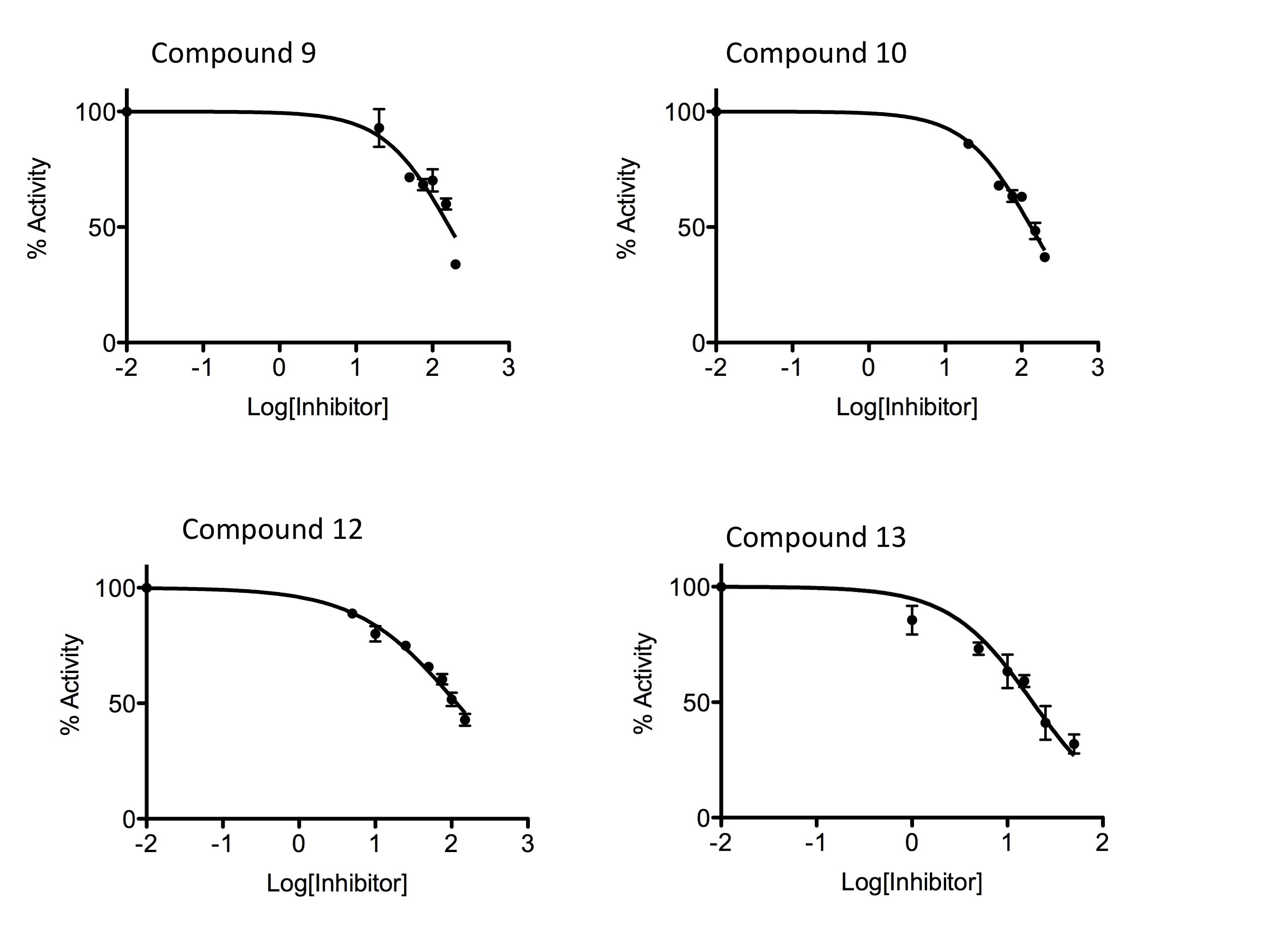


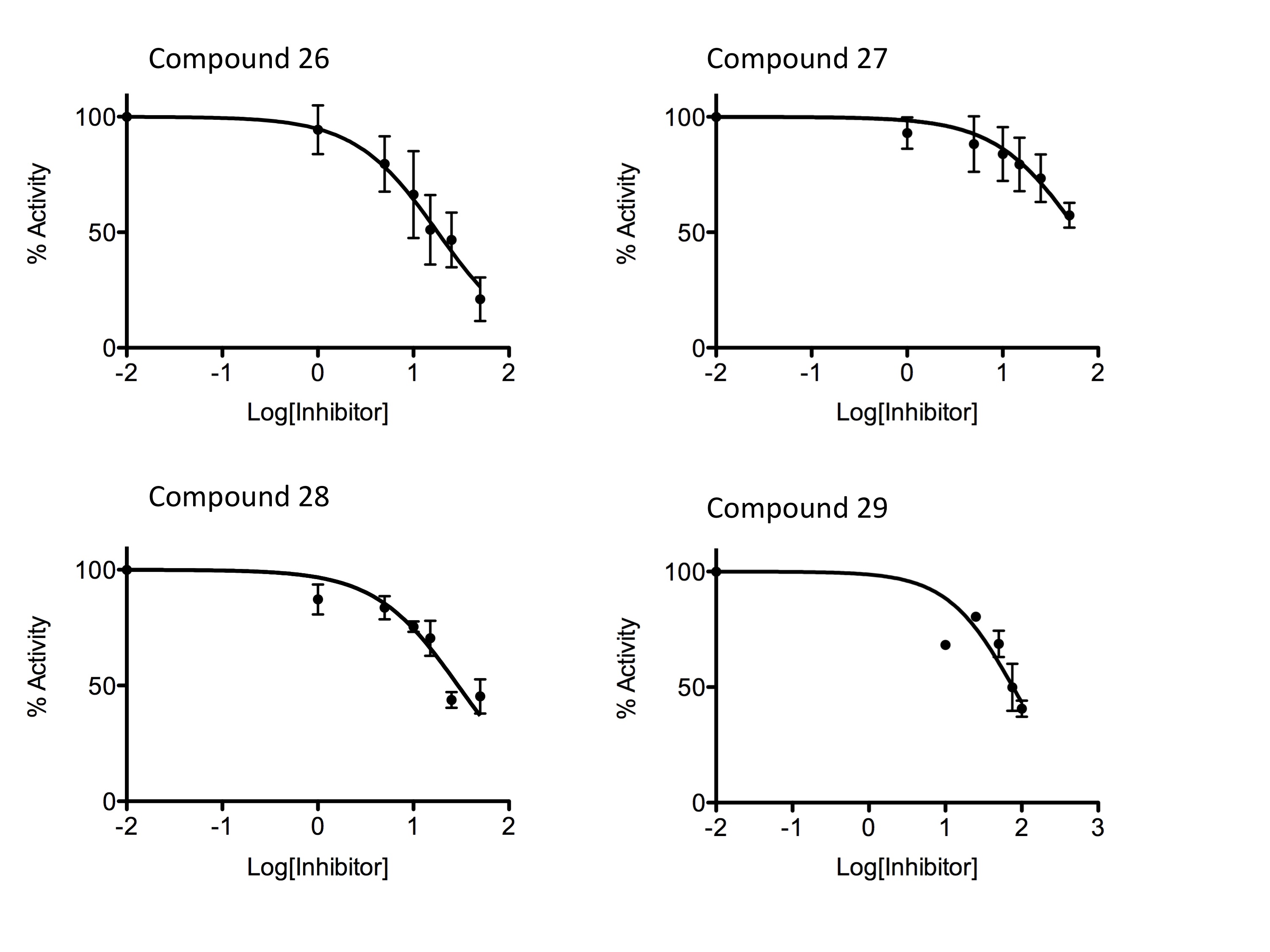


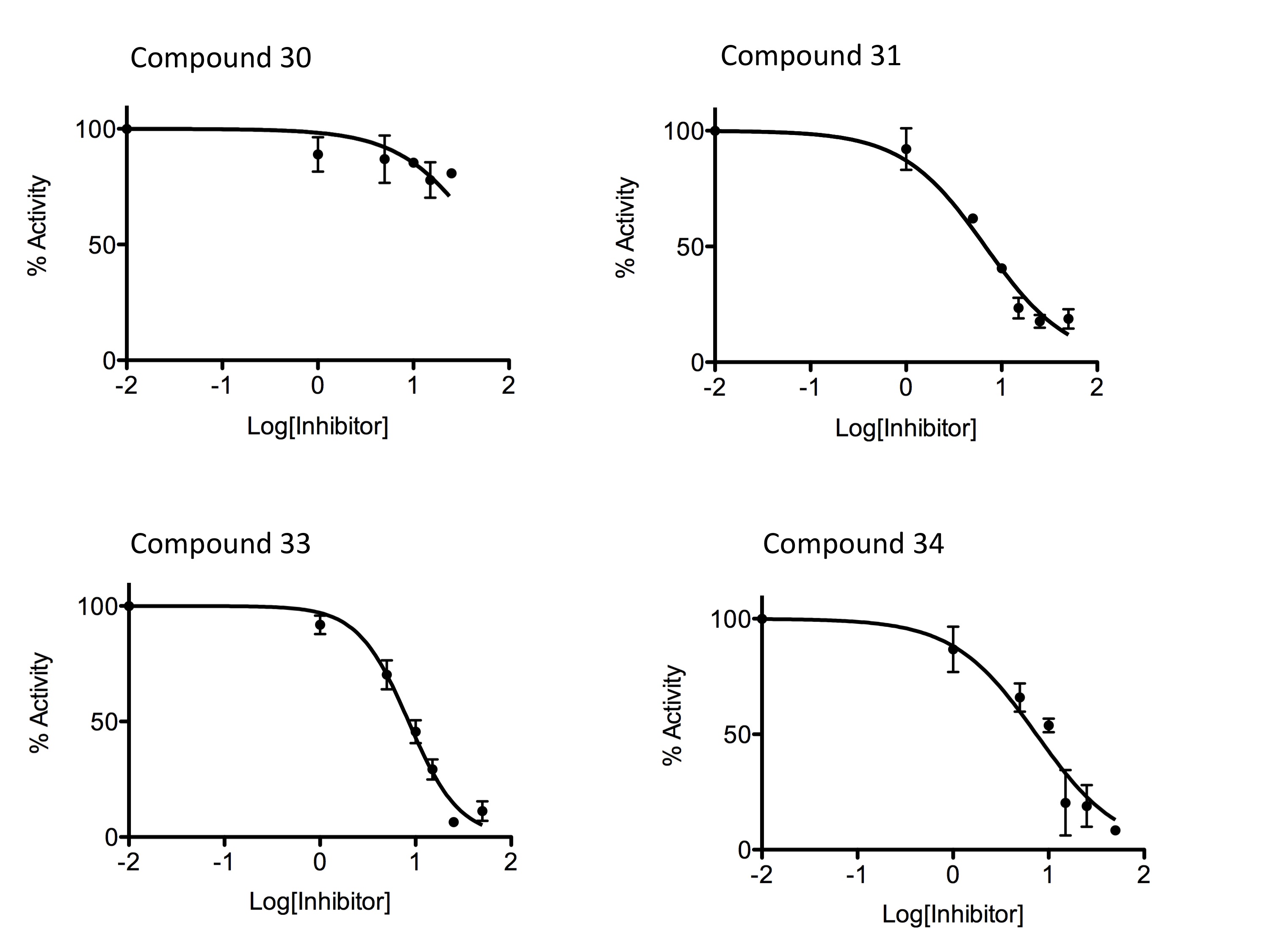


**Figure S2.** IC50 curves corresponding to compounds presented in Table 3 and 4. Curves corresponding to compounds 11 and 32 are presented in Figure 2. 100% activity corresponds to 100% protein activity.
